# Cystobactamid 507: Concise Synthesis, Mode of Action, and Optimization toward More Potent Antibiotics

**DOI:** 10.1002/chem.202000117

**Published:** 2020-04-28

**Authors:** Walid A. M. Elgaher, Mostafa M. Hamed, Sascha Baumann, Jennifer Herrmann, Lorenz Siebenbürger, Jana Krull, Katarina Cirnski, Andreas Kirschning, Mark Brönstrup, Rolf Müller, Rolf W. Hartmann

**Affiliations:** ^1^ Department of Drug Design and Optimization Helmholtz Institute for Pharmaceutical Research Saarland Saarland University Campus E8.1 66123 Saarbrücken Germany; ^2^ Department of Microbial Natural Products Helmholtz Institute for Pharmaceutical Research Saarland Saarland University Campus E8.1 66123 Saarbrücken Germany; ^3^ PharmBioTec GmbH 66123 Saarbrücken Germany; ^4^ Department of Chemical Biology Helmholtz Centre for Infection Research Inhoffenstrasse 7 38124 Braunschweig Germany; ^5^ Institute of Organic Chemistry Leibniz University of Hannover Schneiderberg 1B 30167 Hannover Germany

**Keywords:** antibiotics, conformation analysis, drug design, hydrogen bonds, total synthesis

## Abstract

Lack of new antibiotics and increasing antimicrobial resistance are among the main concerns of healthcare communities nowadays, and these concerns necessitate the search for novel antibacterial agents. Recently, we discovered the cystobactamids—a novel natural class of antibiotics with broad‐spectrum antibacterial activity. In this work, we describe 1) a concise total synthesis of cystobactamid 507, 2) the identification of the bioactive conformation using noncovalently bonded rigid analogues, and 3) the first structure–activity relationship (SAR) study for cystobactamid 507 leading to new analogues with high metabolic stability, superior topoisomerase IIA inhibition, antibacterial activity and, importantly, stability toward the resistant factor AlbD. Deeper insight into the mode of action revealed that the cystobactamids employ DNA minor‐groove binding as part of the drug–target interaction without showing significant intercalation. By designing a new analogue of cystobactamid 919‐2, we finally demonstrated that these findings could be further exploited to obtain more potent hexapeptides against Gram‐negative bacteria.

## Graphical abstract

**Optimized cystobactamid antibiotics**: MedChem optimization of the antibiotic cystobactamid 507 targeting DNA gyrase resulted in the construction of analogues with improved activity. Bioactive conformation was disclosed and the shortest synthetic route established. Mode of action studies revealed specific binding at the DNA minor groove. Incorporation of a tripeptidic cystobactamid 507 analogue into a hexapeptide resulted in superior activity.

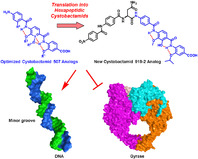

## Introduction

The ongoing prevalence of antibiotic‐resistant bacteria poses an imminent threat to humanity.^1^, ^2^ Therefore, the need to discover novel antibiotics with new chemical scaffolds has been established. Nature represents a rich repository of antibiotics; however, the major part of these natural products (NPs) have to be modified to optimize pharmacokinetic and pharmacodynamic properties.^3^, ^4^

**Figure 1:**
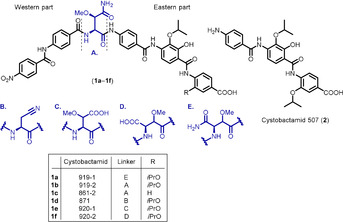
Examples of the natural cystobactamids **1 a**–**f**, **2**.^5^, ^6^

Recently, we reported the discovery of the cystobactamids—a new family of antibiotics—isolated from *Cystobacter* sp. (Figure 1).^5^, ^6^ Cystobactamids (**1 a**–**1 f**) have hexapeptidic structures comprised of three *p*‐aminobenzoic acid motifs (eastern part) and two *p*‐nitro/aminobenzoic acids (western part) connected through different linkers. The structurally simplest natural cystobactamid 507 (**2**), is a tripeptide representing the eastern part of most of the hexapeptides (**1 a**–**f**) (Figure 1). The cystobactamids display broad‐spectrum antibacterial activity through inhibition of topoisomerases type IIA, namely DNA gyrase and topoisomerase IV.^5^ Here, we report studies on the optimization of **2** because it is the only compound with appropriate physicochemical properties for oral absorption according to Lipiniski.^7^

## Results and Discussion

**Figure 2:**
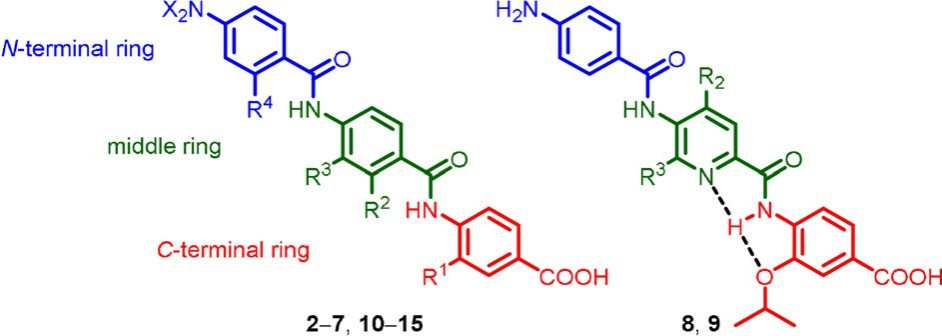
General structure of cystobactamid 507 analogues.

We used gyrase inhibition to establish the structure–activity relationship (SAR), as it is the primary target of cystobactamids in *Escherichia coli*.^5^ The assay evaluates the effect on the DNA supercoiling activity of gyrase using a relaxed circular plasmid as a substrate. Cystobactamids have been shown to inhibit gyrase similar to quinolones through stabilization of the covalent enzyme–DNA complex with double‐stranded DNA breaks resulting in an accumulation of linear DNA.^5^ Previously, we reported that the methyl homologue **3** (Figure 2 and Table 1) showed only a slight decrease in activity, suggesting that the natural antibiotic **2** could be amenable to structural modification.^8^

**Table 1:** In vitro inhibitory activities of compounds **2**–**16** in the gyrase supercoiling assay and topoisomerase IV relaxation assay.

Compd.	R^1^	R^2^	R^3^	R^4^	X	IC_50_ gyrase [μm]^[a]^	IC_50_ topo. IV [μm]^[b]^
**2**	*i*PrO	OH	*i*PrO	H	H	355±25	>500
**3**	MeO	OH	MeO	H	H	463±28	n.d.^[c]^
**4**	*i*PrO	OH	MeO	H	H	360±26	n.d.
**5**	*i*PrO	OH	Cl	H	H	>1000	n.d.
**6**	*i*PrO	MeO	*i*PrO	H	H	115±18	n.d.
**7**	*i*PrO	*i*PrO	*i*PrO	H	H	60±10	175±10
**8**	–	*i*PrO	H	–	–	195±20	n.d.
**9**	–	H	*i*PrO	–	–	50±10	147±10
**10**	*i*PrO	OH	H	H	H	>1000	n.d.
**11**	*i*PrO	H	*i*PrO	H	H	165±18	n.d.
**12**	*i*PrO	H	*i*PrO	OH	H	85±12	255±14
**13**	*i*PrO	OH	H	*i*PrO	H	101±15	n.d.
**14**	*i*PrO	H	*i*PrO	OH	O	110±20	n.d.
**15**	*i*PrO	OH	H	*i*PrO	O	106±18	n.d.
**16**	–	–	–	–	–	3.6	n.d.
CP^[d]^	–	–	–	–	–	0.4±0.05	n.d.

[a] *E. coli* gyrase (1 unit, 20.5 nm). [b] *E. coli* topoisomerase IV (1 unit, 20.5 nm). [c] Not determined. [d] Ciprofloxacin.

We therefore investigated the role of the substituents and the conformation of **2** for activity. Replacement of the isopropoxy side chain in the middle ring of **2** with a methoxy group (**4**) did not impact activity, whereas substitution with chlorine (**5**) resulted in a significant loss of gyrase inhibition (Table 1). This result highlights the importance of the alkoxy side chains for ligand–target interaction. On the other hand, the isopropoxy of the C‐terminal ring showed stronger inhibition than the methoxy in this position (**4** vs. **3**), probably due to its steric and hydrophobic properties. Consequently, we kept it in the optimization process.

**Figure 3:**
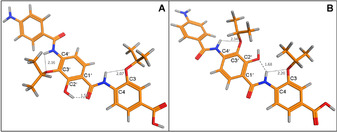
Conformational analysis of cystobactamid 507 (**2**): A) *anti* form (lowest energy conformation); B) *syn* form (Δ*E* 0.4 kcal mol^−1^).

As a next step, we performed in silico conformational analyses of compounds **2**–**4** using molecular dynamics (MD) to identify the potential conformations of the molecules and their relative energies. Generally, the compounds adopt linear conformations with a backbone curvature^9^ of about 158° (Figure S31 and S32). They can adopt two constrained conformations (*anti* and *syn*) with respect to the alkoxy side chains that are controlled by the hydroxy group at the middle ring. The lowest energy conformation is the *anti* form (Figure 3 A), which is stabilized by three intramolecular hydrogen bonds (IMHBs). An IMHB between C4‐NH and C3‐alkoxy group restricts rotation of the C‐terminal ring around the Ar‐NH axis. Another IMBH between C1′‐CO and C2′‐OH restricts rotation of the middle ring around the Ar‐CO axis. The third IMHB between C4′‐NH and C3′‐alkoxy group restricts rotation of the middle ring around Ar‐NH axis. The *syn* conformer is also stabilized by three IMHBs similar to the *anti* form, except that C2′‐OH switches from HB donor to HB acceptor and forms a six‐membered ring with C4‐NH (restricting rotation of the middle ring around the Ar‐CO axis) (Figure 3 B). The energy difference (Δ*E*) between the *anti* and *syn* forms is 0.4–0.7 kcal mol^−1^. Such a minor energy difference could allow the interconversion between both conformations at ambient temperature.

**Figure 4:**
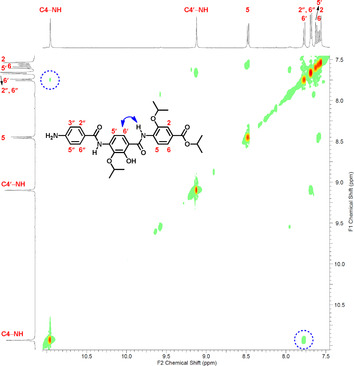
2D‐NOESY spectrum of compound **26** in a cryoprotective mixture (20 % H_2_O/[D_6_]DMSO) adopting *anti* conformation.

We then investigated the preferred conformation for **2**–**4**, their esters and nitro precursors experimentally in solution by a NOESY study in a biomimetic solvent (20 % H_2_O/[D_6_]DMSO).^10^ In agreement with MD calculations, all compounds show a strong cross‐peak between C4‐NH and C6′‐H, and a weak or no cross‐peak with C2′‐OH, indicating that these compounds predominantly exist in the *anti* conformation (Figure 4 and S8). Using standard NMR solvents, the same results were obtained (Figure S1–S10).

Subsequently, we verified whether the *syn* conformer could exist under physiological conditions by a ^1^H NMR experiment for the cystobactamid 507 isopropyl ester (**26**) at 20 and 37 °C. We found that the chemical shift of the C2′‐OH proton moved upfield from 12.40 ppm at 20 °C (mainly HB donor form, *anti*) to 12.35 ppm at 37 °C (pushing the conformational equilibrium to HB acceptor form, *syn*) (Figure S29). In addition, we observed the *syn* conformation in the solid state by determining the crystal structure for the dipeptide precursor of **3** (Figure S37E), whereas in solution there was a prevalence of the *anti* conformation (Figure S11). These results demonstrate that these compounds can easily interconvert from *anti* to *syn* at physiological temperature.

Since the *anti* form was the predominant conformation in solution for **2** and all derivatives so far, we designed compounds that preferentially adopt the *syn* form by blocking the ambiguous hydroxy motif by methylation, thus converting it into a HB acceptor group only (compound **6**). MD calculations indicated that **6** typically adopts an IMHB‐stabilized *syn* conformation with a large Δ*E* compared to the *anti* form (3.8 kcal mol^−1^). NOESY studies showed a cross‐peak between C4‐NH and C2′‐OMe, and no cross‐peak with C6′‐H, indicating that the *syn* conformation is predominant (Figure S15). Compound **6** showed a threefold higher activity than **2** (Table 1), indicating that the hydroxy group is not essential for activity.

To clarify whether this enhancement was due to the induction of the *syn* conformation or due to an additional hydrophobic interaction, we first designed compound **7**, bearing an isopropoxy group in place of the methoxy (**6**). This modification resulted in a six‐fold improvement in activity compared to **2**, indicating that, besides restricting the conformation to the *syn* form, alkoxy groups at position 2 of the middle ring also contribute to target interactions.

**Figure 5:**
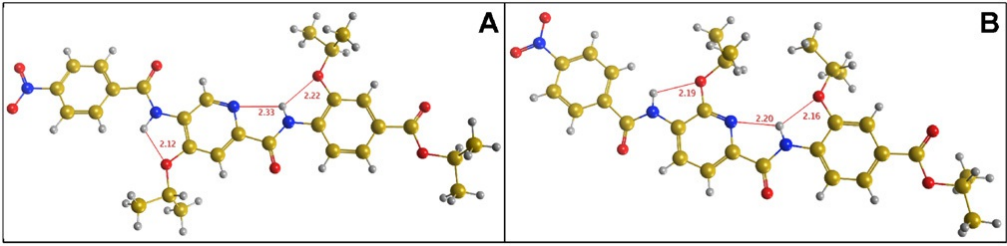
X‐ray crystal structures of the nitro ester precursors of **8** (**93**, A) and **9** (**94**, B) adopting *anti* and *syn* conformation, respectively, via IMHB.

To investigate whether the *syn* conformation is a decisive factor, we then designed two rigid cystobactamid 507 analogues, **8** and **9**, with a pyridine scaffold adopting only one conformation, *anti* or *syn*, respectively (Figure 2). Rigidity was achieved via a bifurcated IMHB between C4‐NH and oxygen atom of C3‐isopropoxy as well as the nitrogen atom of the pyridine ring. We confirmed the stability of **8** and **9** by MD calculations, which showed only the desired conformation in an energy window Δ*E* of 7.0 kcal mol^−1^. In NOESY experiments, no cross‐peak was observed between C4‐NH and pyridine C3‐H at 27 °C and at higher temperatures up to 67 °C (Figure S21–S27). Moreover, X‐ray crystal structures of the compounds were as expected (Figure 5, S37C and S37D). Results revealed that **9** is fourfold and sevenfold more potent than **8** and **2**, respectively (Table 1), indicating that the *syn* form is indeed the more active conformation of cystobactamid 507. Notably, the slightly better activity of **8** than **2** suggests that the introduced pyridine ring might contribute to the interaction with gyrase and the inhibitory effect.

The newly designed compound **9** is clearly advantageous compared to the natural compound **2**. Introduction of a polar pyridine ring in lieu of the benzene enhances water solubility and ligand‐lipophilicity efficiency^11^ (Sol_pH 7.4_: 59.4 μmol mL^−1^, LLE: 3.54 for **9** vs. 9.6 μmol mL^−1^, 1.75 for **2**, respectively). In addition, removal of the hydroxy group, while keeping the right conformation, increases ligand efficiency^11^ (LE: 0.17 for **9** vs. 0.13 for **2**). Moreover, the enhancement of the binding affinity of the rigid ligand **9** is at least in part due to the reduction of the unfavorable entropic contribution to the Gibbs free energy of binding.^12^

**Figure 6:**
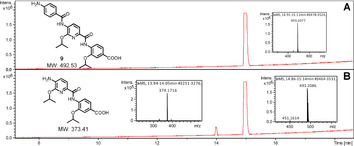
Stability of **9** against AlbD: HPLC‐MS analysis of **9** (12 μm) in phosphate buffer as control (A) and incubated with AlbD (24 μm) for 3 h at 28 °C showing only traces of the cleavage product (B).

Another important achievement could be that hopping of the cystobactamid 507 scaffold to the novel pyridine‐based chemotype (compound **9**) might circumvent the cystobactamids’ cleavage through AlbD, a known resistance protein inactivating the structurally related antibiotics albicidins.^13^ The AlbD endopeptidase hydrolyses the amide bond between the middle and the N‐terminal ring of the eastern part of albicidins/cystobactamids and the tripeptidic derivatives.^13^ By incubation of compound **9** with AlbD under the reported conditions,^13^ our hypothesis turned out to be correct; no cleavage of **9** in the presence of AlbD was observed (Figure 6).

For further SAR exploration, compound **2** was simplified by omitting either the isopropoxy or the hydroxy group from the central ring in **10** and **11**, respectively (Table 1). Removal of the former resulted in a loss of activity, emphasizing the importance of alkoxy substituents for activity. Interestingly, removing the hydroxy group increased the activity twofold compared to **2**. This confirms that the hydroxy group is not essential for activity. Its removal permits free rotation of the middle ring, as evidenced in the NOESY spectrum of **11** showing two cross‐peaks of equal intensities between C4‐NH and C2′‐H (*syn*) and C6′‐H (*anti*) (Figure S28).

Introduction of an isopropoxy or hydroxy group at the N‐terminal ring of **10** and **11**, to maintain the beneficial free rotation, resulted in restoration of activity with a fourfold enhancement compared to **2** (**13** and **12**, respectively) (Table 1). These results demonstrate the usefulness of the alkoxy motifs (**2** and **13** vs. **10**), and indicate that varying the distance between them can be tolerated because of the flexibility of the new analogues.

Esterification of **4** and the most potent compounds **6**, **7**, **9** and **11**–**13** decreased activity strongly, revealing that the terminal carboxyl group is important for activity (Table S2). Replacing the amino moiety of **12** and **13** with nitro groups, as in the hexapeptidic cystobactamids, resulted in similar inhibitory activities (**14** and **15**, respectively) (Table 1). This indicates that substitution in the 4′′‐position of cystobactamid 507 analogues provides only little contribution to activity.

**Scheme 1:**
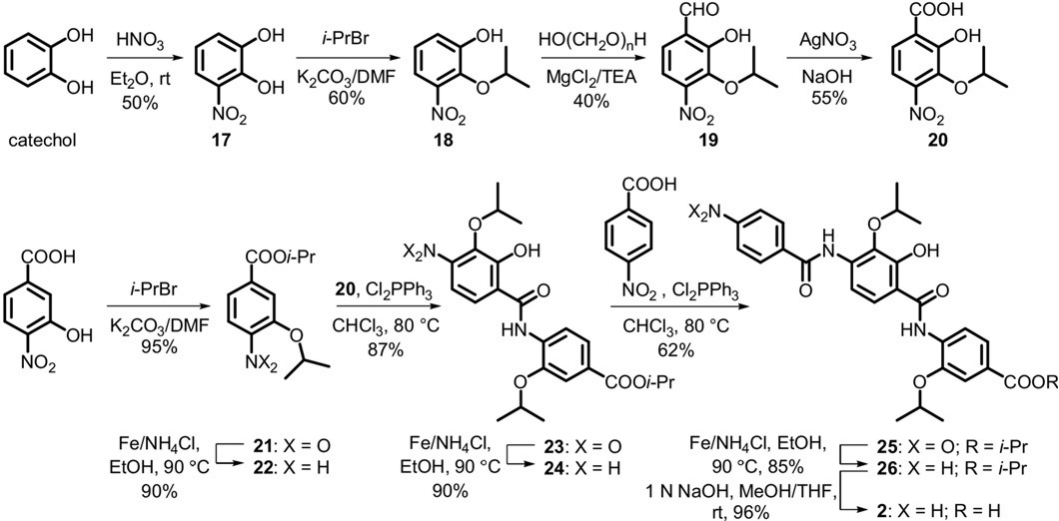
Synthesis of cystobactamid 507 (**2**).

For the synthesis of **2** and its analogues, retrosynthetic analysis revealed three units of either *p*‐aminobenzoic acid or 5‐aminopicolinic acid derivatives linked by amide bonds. Accordingly, the individual middle, C‐ and N‐terminal rings were prepared followed by the coupling of the constituents. We established brief and efficient synthetic pathways for novel as well as reported amino acids, which are also precursors of other NPs^14^, ^15^, ^16^, ^17^ (Scheme S1). Compared to previous methods,^6^, ^8^, ^18^ the middle ring of **2** (**20**) was prepared in only four steps using catechol as a starting material. Nitration of the catechol to the 3‐nitro derivative **17** followed by regioselective isopropylation at the 2‐hydroxy position using a stoichiometric amount of 2‐bromopropane provided **18**. *ortho*‐Formylation of **18** with paraformaldehyde in MgCl_2_/TEA/MeCN mixture under strictly anhydrous conditions produced the *p*‐nitrobenzaldehyde **19**. The latter was finally oxidized using AgNO_3_ under basic conditions to yield the corresponding acid **20** (Scheme 1), the structure of which was confirmed by X‐ray crystallography (Figure S37A). The C‐terminal ring **22** was prepared by isopropylation of 3‐hydroxy‐4‐nitrobenzoic acid followed by reduction of the resulting nitro derivative **21** via heating with iron in ethanol (Scheme 1).

For amide coupling, three obstacles were encountered: The free OH as an interfering group, the nonreactive carboxylic acids, and the weakly reactive aromatic amines.^6^, ^8^, ^16^, ^18^ We developed a straightforward strategy that could overcome these difficulties and bypass the activation of carboxylic acids and phenolic OH protection/deprotection.^6^, ^8^, ^16^, ^18^ The total synthesis of **2**, for example, was accomplished in overall 11 steps instead of 21 and 13, respectively.^8^, ^18^ Coupling of the central ring **20** to the C‐protected C‐terminal ring **22** was achieved by heating the mixture with dichlorotriphenylphosphorane in anhydrous chloroform to afford dipeptide **23** (Scheme 1). After reduction, the corresponding amine **24** was coupled with *p*‐nitrobenzoic acid by using the same procedure as described above. Reduction of the nitro substituted tripeptide **25** followed by C‐deprotection via ester saponification yielded the amino acid **2** (Scheme 1). This synthetic procedure was broadly applicable and enabled us to prepare a large series of peptidomimetics in short time with good to excellent yields (Scheme S2–S4).

We evaluated the inhibitory activity of **2** and the most potent gyrase inhibitors **7**, **9**, and **12** on the second bacterial target of cystobactamids (topoisomerase IV) using a relaxation assay, which assesses the effect on the conversion of a supercoiled plasmid into a relaxed form.^5^ Results indicated that **2** was not active up to 500 μm, whereas the new analogues displayed moderate inhibitory activities (Table 1). This demonstrates that the modifications applied to improve the gyrase inhibitory activity are also valid for topoisomerase IV, which is not surprising due to the high homology between both enzymes.^19^

**Figure 7:**
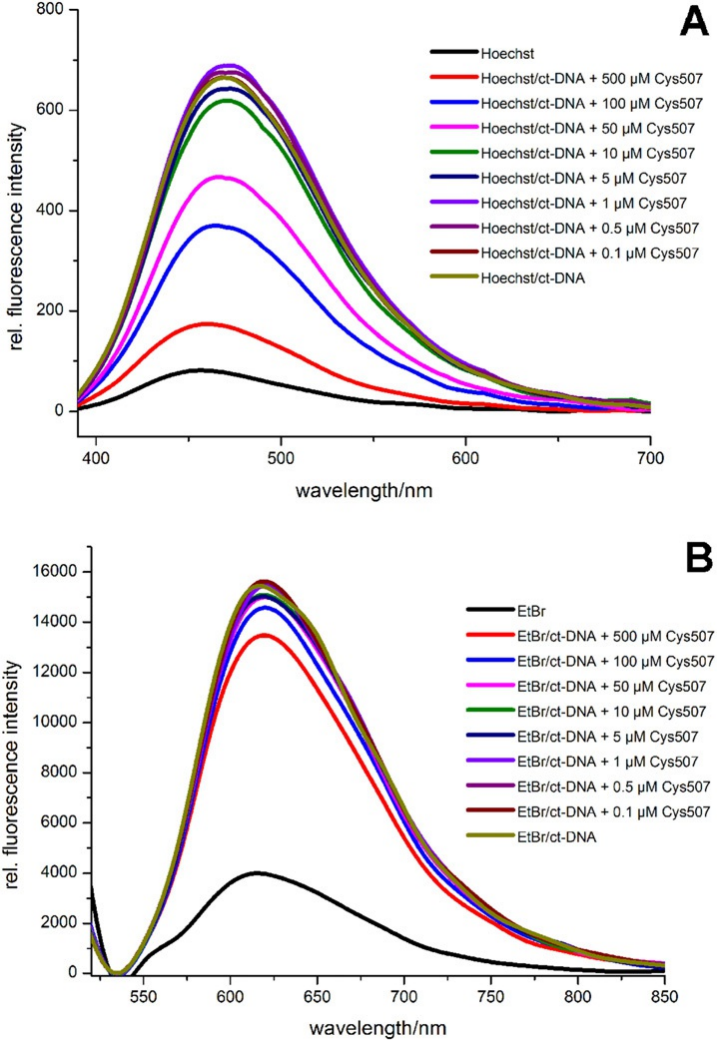
DNA interaction of cystobactamid 507 (**2**): A) Concentration‐dependent decrease in fluorescence of Hoechst 33342 bound to calf thymus DNA (ct‐DNA, 15 μm each) upon titration with **2**; B) no change in fluorescence of the ct‐DNA bound EtBr upon titration with **2**.

Moreover, the same activity trend of the compounds on both targets suggests a similar mode of action. The primary binding site of the cystobactamids is probably located at the gyrase–DNA interface overlapping that of the quinolone antibiotics.^5^ To gain a deeper insight into the mode of action, we investigated whether and eventually how cystobactamids and their analogues bind to the DNA part of the target complex. There are two main binding modes of small molecules to DNA: minor groove binding or intercalation.^20^ Intercalation is of particular concern, as compounds that adopt this binding mode may trigger genotoxic effects in eukaryotes.^21^ We carried out displacement titration experiments using fluorescent dyes that show increased fluorescence upon DNA binding: Hoechst 33342 for DNA minor‐groove binding and ethidium bromide (EtBr) for intercalation. Titration of calf thymus DNA bound Hoechst 33342 with cystobactamids **1 a**, **1 b**, and **2**–**15** induced a concentration‐dependent loss of fluorescence (Figure 7 A and S39). No compound‐induced fluorescence quenching was observed in the absence of DNA. In the presence of EtBr, no compound showed significant reduction in fluorescence (Figure 7 B and S39). These results indicate that the cystobactamids are able to bind to DNA utilizing the minor groove without significant intercalation. Moreover, they reveal that the cystobactamids and their analogues are a new chemical frame for minor groove recognition besides the known family of five‐membered fused and non‐fused heterocyclic polyamides.^22^

Evaluation of the antibacterial activity against a panel of Gram‐positive and Gram‐negative bacteria revealed that the new compounds show up to 8‐ to 16‐fold enhanced activities against Gram‐positive strains compared to the parent antibiotic **2** (Table 2). A good correlation between the antibacterial effects and topoisomerases IIA inhibitory activities was observed. In contrast to **1 a** and **1 b**,^5^, ^6^ compound **2** and its analogues did not show activity against *E. coli* wild‐type, but they were active against the efflux‐deficient *E. coli* tolC3 mutant. This implies that efflux is responsible for the inactivity toward *E. coli* wild‐type. Some compounds, like **7** and **12**, suffered from penetration issues through the Gram‐negative outer membrane, as indicated by the enhanced MIC values (eightfold) in the presence of a permeability enhancer.

**Table 2:** Antibacterial activities of cystobactamids **1 b**, **2** and synthetic analogues.

Compd.	MIC [μg mL^−1^]						
	*S. aureus* Newman	*S. pneumoniae* DSM‐20566	*M. luteus* DSM‐1790	*E. faecalis* ATCC‐29212	*E. coli* DSM‐1116	*E. coli* DSM‐26863^[b]^	*E. coli* DSM‐26863+PMBN^[c]^
**2**	n.d.^[a]^	64	128	64–128	>128	>128	32–64
**4**	64	16–32	>64	>64	>64	>64	>64
**6**	32	16	64	32	>64	4	2
**7**	8	8–16	32	16–32	>64	32	4
**9**	8	>64	8–16	>64	>64	16	4
**12**	32	8	>64	64	>64	32	4
**13**	8	4–8	64	32	>64	16	4
**16**	n.d.	n.d.	n.d.	n.d.	1	0.25	n.d.
**1 b**	n.d.	n.d.	n.d.	n.d.	2	0.5	n.d.
CP^[d]^	0.1	0.8	0.8	0.8	0.013	0.003	n.d.

[a] Not determined. [b] Δ*tolC3* genotype. [c] Polymyxin B nonapeptide (3 μg mL^−1^). [d] Ciprofloxacin.

Additionally, we investigated compounds **2**, **7**, **9**, and **12** for their phase I and II biotransformation using human liver S9 fraction. Cystobactamid 507 and all synthetic compounds displayed an extraordinarily high metabolic stability (*t*_1/2_>240 min, Figure S42). Surprisingly, stability was observed for both amide groups against peptidases as well as for the hydroxy groups of **2** and **12** toward conjugating enzymes. Moreover, conformational modification *anti* to *syn*, i.e., **2** to **7** and **9** maintained the outstanding metabolic stability of the natural compound.

**Scheme 2:**
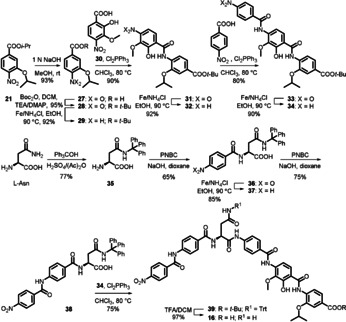
Synthesis of cystobactamid 919‐2 analogue **16**.

Finally, to demonstrate that structure optimization of **2** can be translated into potent hexapeptides, we picked an analogue with a hydroxy group at the middle ring to keep the general features of isolated cystobactamids.^6^ Accordingly, compound **4** was connected to the western part of the natural compounds through l‐asparagine as a simplified linker to form the cystobactamid 919‐2 analogue **16** (Scheme 2). Compound **16** showed potent gyrase inhibition and antibacterial activity on the Gram‐negative *E. coli* wild type (Table 1 and 2). This indicates that modification of the natural cystobactamid 919‐2 using the tripeptidic cystobactamid 507 analogues is an appropriate strategy to improve activity. Moreover, removal of the methoxy group at the linker was tolerated, offering a new space for further optimization.

The hexapeptide **16** was prepared via convergent synthesis including a coupling of two tripeptide fragments; namely, the eastern and the western part with a linker.^23^ The eastern part was prepared as described above as C‐Boc protected amino acid **34** (Scheme 2). Synthesis of the western fragment started with trityl protection of l‐asparagine at the amide residue to give **35** (Scheme 2). Reaction of **35** with *p*‐nitrobenzoylchloride (PNBC) under Schotten–Baumann conditions afforded the dipeptide **36**. Reduction of the nitro group to the amine **37** followed by benzoylation yielded tripeptide **38**. Coupling of the latter to the amine **34** using Cl_2_PPh_3_ afforded hexapeptide **39**. General deprotection of **39** using TFA 15 % (v/v) in DCM yielded **16** (Scheme 2).

## Conclusions

We described a brief synthetic route for the natural antibiotic cystobactamid 507 (**2**) and the design of new derivatives with improved topoisomerases IIA inhibition, and antibacterial activity. SAR studies revealed the importance of the alkoxy side chains and the irrelevance of the hydroxy group for gyrase inhibition. The terminal carboxy and amino/nitro moieties were found to be necessary for activity. The molecular conformation in solution as well as in the solid state was investigated to deduce the conformation–activity relationship. We pioneered the design of noncovalently bonded rigid structures through IMHBs to disclose the bioactive conformation of **2** as the *syn* form without using the classical methods of cyclization or introduction of sterically demanding moieties.^24^ Increase of chemical diversity by modification of the cystobactamid 507 scaffold into a pyridine‐based structure (compound **9**) turned out to make the molecule stable toward the albicidin resistance factor AlbD. It was shown that the cystobactamids’ mode of action could be, at least in part, mediated by DNA minor‐groove binding and not intercalation. An important advantage of **2** and the novel analogues is the high metabolic stability. Ultimately, we demonstrated that optimization of the tripeptides could be translated into hexapeptidic cystobactamids with improved gyrase inhibition and antibacterial activity against Gram‐negative strains. It is worth mentioning that compounds could be designed with better pharmacokinetic properties compared to the natural compounds. Thus, this work would be useful for the development of cystobactamids and similar natural compounds^14^, ^15^, ^16^ toward better antibiotics.

## Experimental Section

Experimental procedures for the synthesis of the final compounds and intermediates as well as their characterization, NMR spectra, computational work, biological experiments and X‐ray crystallographic data are described in detail in the Supporting Information.

CCDC 1958329 (**20**), 1958330 (**44**), 1958331 (**91**), 1958332 (**93**), 1958333 (**94**), 1958334 (**96**), and 1958335 (**97**) contain the supplementary crystallographic data for this paper. These data are provided free of charge by The Cambridge Crystallographic Data Centre.

## Conflict of interest

The authors declare no conflict of interest.

## Supporting information

As a service to our authors and readers, this journal provides supporting information supplied by the authors. Such materials are peer reviewed and may be re‐organized for online delivery, but are not copy‐edited or typeset. Technical support issues arising from supporting information (other than missing files) should be addressed to the authors.

Supplementary
