# Supplementary material for: Cystobactamid 507: Concise Synthesis, Mode of Action, and Optimization toward More Potent Antibiotics
Source: Chemistry. 2020 Apr 28;26(32):7219–25. doi: 10.1002/chem.202000117 (PMC7317206; doi:10.1002/chem.202000117)
Supplement: Supplementary file 1 — Supplementary [file CHEM-26-7219-s001.pdf]

Chemistry–A European Journal

Supporting Information

Cystobactamid 507: Concise Synthesis, Mode of Action, and Optimization toward More Potent Antibiotics

Walid A. M. Elgaher+,[a] Mostafa M. Hamed+,[a] Sascha Baumann,[b] Jennifer Herrmann,[b] Lorenz Siebenbürger,[c] Jana Krull,[d] Katarina Cirnski,[b] Andreas Kirschning,[e] Mark Brönstrup,[d] Rolf Müller,[b] and Rolf W. Hartmann*[a]

Table of Contents

Materials and Methods	page S3
Chemistry	page S4
2D-NOESY Measurement in a Cryoprotective Mixture	page S46
Conformational Interconversion at Physiological Temperature	page S46
Effect of Temperature on Rigidity	page S46
Computational Chemistry	page S75
X-ray Structure Determination	page S78
Biology	page S79
Gyrase Supercoiling Assay	page S80
Topoisomerase IV Relaxation Assay	page S81
DNA Competition Assay Using Hoechst 33342 and Ethidium Bromide (EtBr)	page S82
Minimal Inhibitory Concentration (MIC) Determination	page S84
AlbD Cleavage Assay	page S84
Metabolic Stability Assay	page S86
References	page S86
^{1}H- and ^{13}C-NMR Spectra of the Described Compounds	page S87

Materials and Methods

Starting materials and solvents were purchased from commercial suppliers, and used without further purification. All chemical yields refer to purified compounds and were not optimized. Reaction progress was monitored using TLC silica gel 60 F_{254} aluminum sheets, and visualization was accomplished by UV at 254 nm. Flash chromatography was performed using silica gel 60 Å (40−63 µm). Preparative RP-HPLC was carried out on a Waters Corporation setup containing a 2767 sample manager, a 2545 binary gradient module, a 2998 PDA detector and a 3100 electron spray mass spectrometer. Purification was performed using a Waters XBridge column (C18, 150 mm × 19 mm, 5 µm), a binary solvent system A and B (A = water with 0.1% formic acid; B = MeCN with 0.1% formic acid) as eluent, a flow rate of 20 mL/min, and a gradient of 60% to 95% B in 8 min were applied. Melting points were determined on a Stuart Scientific melting point apparatus SMP3 (Bibby Sterilin, UK), and are uncorrected. NMR spectra were recorded on either a Bruker DRX-500 (^{1}H, 500 MHz; ^{13}C, 126 MHz) or Bruker Fourier 300 (^{1}H, 300 MHz; ^{13}C, 75 MHz) spectrometer at 300 K. Chemical shifts were recorded as δ values in ppm units by reference to the hydrogenated residues of deuterated solvent as internal standard ($CDCl_3$, δ = 7.27, 77.00; DMSO-d_6, δ = 2.50, 39.51, acetone-d_6: δ = 2.05, 29.92, 206.68). Splitting patterns describe apparent multiplicities and are designated as s (singlet), br s (broad singlet), d (doublet), dd (doublet of doublet), t (triplet), q (quartet), m (multiplet). Coupling constants (*J*) are given in hertz (Hz). Weak or coalesced signals were elucidated by heteronuclear multiple quantum coherence (HMQC) and heteronuclear multiple bond coherence (HMBC) 2D-NMR techniques. Purity of all compounds used in biological assays was ≥ 95% as measured by LC/MS Finnigan Surveyor MSQ Plus (Thermo Fisher Scientific, Dreieich, Germany). The system consists of LC pump, autosampler, PDA detector, and single-quadrupole MS detector, as well as the standard software Xcalibur for operation. RP C18 Nucleodur 100-5 (125 mm × 3 mm) column (Macherey-Nagel GmbH, Dühren, Germany) was used as stationary phase, and a binary solvent system A and B (A = water with 0.1% TFA; B = MeCN with 0.1% TFA) was used as mobile phase. In a gradient run the percentage of B was increased from an initial concentration of 0% at 0 min to 100% at 15 min and kept at 100% for 5 min. The injection volume was 10 µL and flow rate was set to 800 µL/min. MS (ESI) analysis was carried out at a spray voltage of 3800 V, a capillary temperature of 350 °C, and a source CID of 10 V. Spectra were acquired in positive mode from 100 to 1000 m/z and at 254 nm for UV tracing. High-resolution mass spectrometry (HRMS) data was determined by a Thermo Scientific Q Exactive Focus system.

Chemistry

Synthesis and experimental data of compounds **30**, **40**, **41** and **97** were described in a previous work.[1] Compounds **56**, **57** and **68** are commercially available.

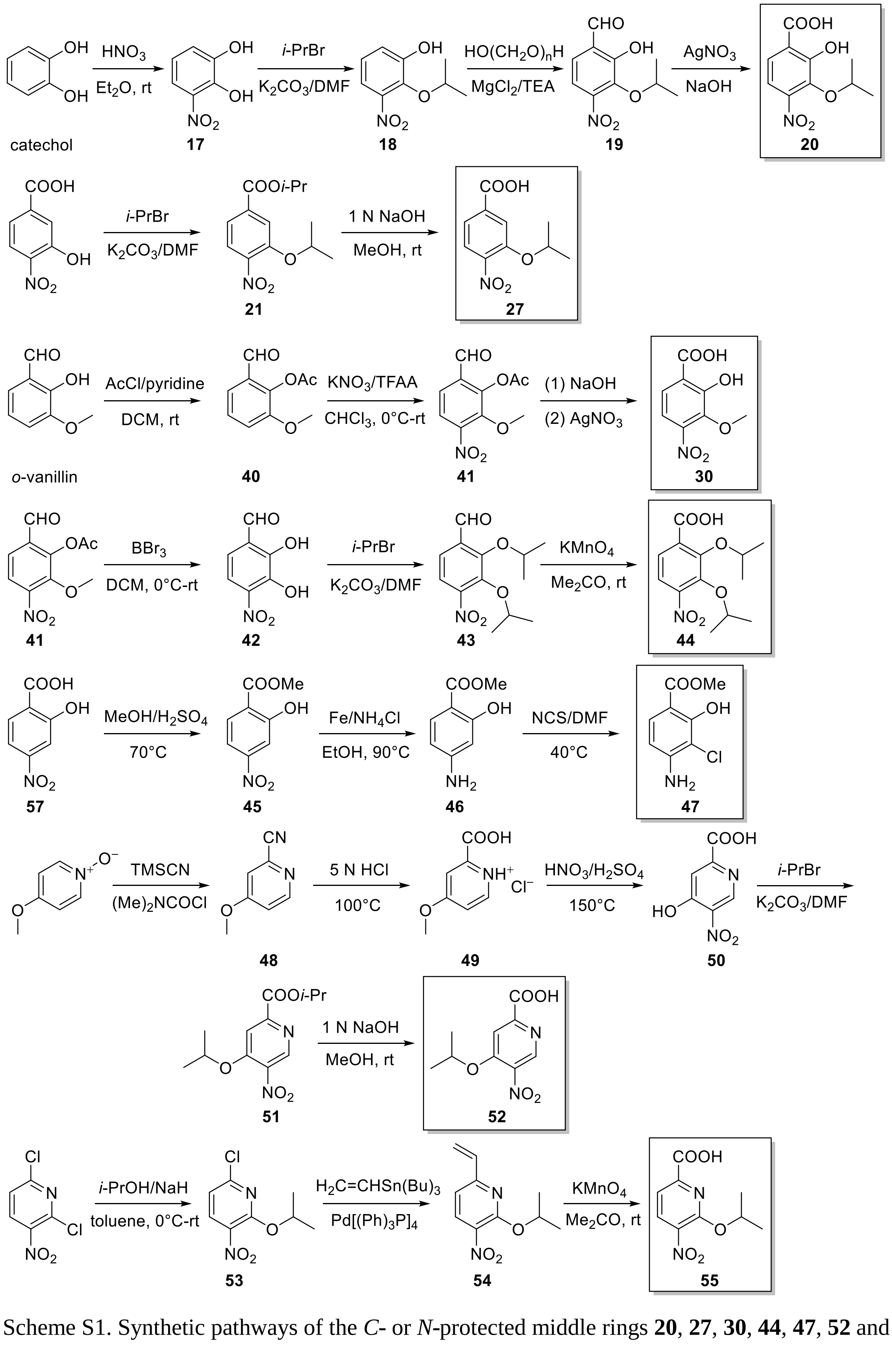

Scheme S1. Synthetic pathways of the *C*- or *N*-protected middle rings **20**, **27**, **30**, **44**, **47**, **52** and **55**.

Compound **27** was prepared by first alkylation of 3-hydroxy-4-nitrobenzoic acid then hydrolysis of the produced ester **21** (Scheme S1). Synthesis of **30** started via acetylation of *o*-vanillin followed by nitration of **40** using KNO_3/trifluoroacetic anhydride mixture to afford the *p*-nitrobenzaldehyde **41**. Oxidation of **41** with $AgNO_3$ delivered the acid **30**. Universal *O*-demethylation and deacetylation of **41** using BBr_3 produced the dihydroxy derivative **42**. Isopropylation of **42** to the aldehyde **43** followed by oxidation with $KMnO_4$ afforded the carboxylic acid **44**. Structure of **44** was confirmed by X-ray (Fig. S37B). Compound **47** was prepared through esterification of 4-nitrosalicylic acid to the methyl ester **45**. Chemical reduction of **45** via heating with iron in ethanol resulted in the corresponding amine **46**. Chlorination of the activated **46** using *N*-chlorosuccinimide yielded **47**. Synthesis of 4-isopropoxypicolinic acid **52** was accomplished via Fife reaction of 4-methoxypyridine-*N*-oxide to furnish the nitrile derivative **48**. Acidic hydrolysis of **48** then nitration of the hydrochloride salt **49** produced exclusively the *O*-demethylated 5-nitro derivative **50**. Isopropylation of **50** followed by saponification of the isopropyl ester **51** gave the picolinic acid **52**. Structure of **52** was evidenced by X-ray (Fig. S37C). The 6-isopropoxypicolinic acid **55** was obtained from 2,6-dichloro-3-nitropyridine via the reaction with isopropyl alcohol under basic condition to yield **53**. Stille coupling of **53** produced the vinyl derivative **54**, which was oxidized to afford the picolinic acid **55**.

3-Nitrobenzene-1,2-diol 17

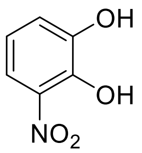

To a stirred solution of catechol (20.0 g, 182 mmol) in diethyl ether (450 mL) cooled at 0 °C in an ice bath, fuming HNO_3 (9 mL) was added dropwise. The reaction mixture was allowed to warm to room temperature and was further stirred for 24 h. Solvent was removed *in vacuo*. The residue was dissolved in EtOAc and purified using flash chromatography (SiO_2, *n*-hexane–EtOAc = 4:1).
Yield 50%; yellow crystals; ^1H NMR (300 MHz, $CDCl_3$) δ 10.63 (br s, 1H), 7.66 (dd, *J* = 8.7, 1.4 Hz, 1H), 7.25 (dq, *J* = 8.1, 0.7 Hz, 1H), 6.91 (dd, *J* = 8.7, 8.1 Hz, 1H), 5.87 (br s, 1H); ^{13}C NMR (75 MHz, $CDCl_3$) δ 146.50, 142.77, 133.74, 121.67, 119.74, 115.80; m/z (ESI+) 155 $[M]^+$.

2-Isopropoxy-3-nitrophenol 18

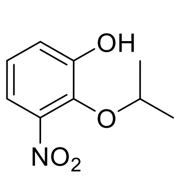

To a stirred mixture of **17** (12.41 g, 80 mmol) and K_2CO_3 (11.06 g, 80 mmol) in DMF (120 mL), 2-bromopropane (9.84 g, 80 mmol) was added. The reaction mixture was stirred at 90 °C overnight. Solvent was evaporated *in vacuo*. The residue was diluted with water (200 mL) and the medium was acidified cautiously by $KHSO_4$ (saturated aqueous solution) to pH 4–5. The resulting mixture was

extracted with EtOAc (3 × 200 mL). The combined organic extract was washed with brine, dried over anhydrous $MgSO_4$, and the solvent was removed by vacuum distillation. The residue was purified using flash chromatography (SiO_2, *n*-hexane–EtOAc = 5:1 to 3:1).

Yield 60%; yellow oil; ^1H NMR (300 MHz, $CDCl_3$) δ 7.47 (dd, *J* = 8.2, 1.7 Hz, 1H), 7.22 (dd, *J* = 8.2, 1.7 Hz, 1H), 7.09 (t, *J* = 8.2 Hz, 1H), 6.05 (br s, 1H), 4.32 (septet, *J* = 6.1 Hz, 1H), 1.37 (d, *J* = 6.1 Hz, 6H); ^{13}C NMR (75 MHz, $CDCl_3$) δ 151.35, 142.97, 138.61, 123.95, 120.07, 116.97, 79.58, 22.46 (2C); m/z (ESI+) 198 $[M + H]^+$.

2-Hydroxy-3-isopropoxy-4-nitrobenzaldehyde 19

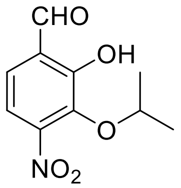

To a stirred mixture of **18** (2.96 g, 15 mmol), anhydrous $MgCl_2$ (7.14 g, 75 mmol) and dry TEA (Na) (15.18 g, 150 mmol) in dry MeCN (molecular sieve) (75 mL), dry paraformaldehyde (Al_2O_3) (3.15 g, 105 mmol) was added under a nitrogen atmosphere. The reaction mixture was stirred at 90 °C for 24 h. The reaction was quenched with water (100 mL) and the medium was acidified by 37% HCl to pH 4–5. The mixture was extracted with EtOAc (3 × 100 mL). The combined organic extract was washed with brine, dried over anhydrous $MgSO_4$, and the solvent was removed by vacuum distillation. The residue was purified using flash chromatography (SiO_2, *n*-hexane–EtOAc = 3:1).

Yield 40%; yellow oil; ^1H NMR (500 MHz, $CDCl_3$) δ 11.44 (br s, 1H), 9.98 (s, 1H), 7.39 (d, *J* = 8.5 Hz, 1H), 7.24 (d, *J* = 8.5 Hz, 1H), 4.89 (septet, *J* = 6.3 Hz, 1H), 1.33 (d, *J* = 6.3 Hz, 6H); ^{13}C NMR (126 MHz, $CDCl_3$) δ 196.24, 156.36, 149.19, 139.57, 127.12, 122.42, 114.15, 77.25, 22.32 (2C); m/z (ESI+) 225 $[M]^+$.

2-Hydroxy-3-isopropoxy-4-nitrobenzoic acid 20

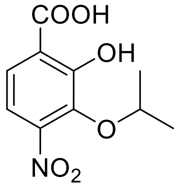

To a stirred solution of **19** (901 mg, 4 mmol) and NaOH (640 mg, 16 mmol) in water (30 mL), $AgNO_3$ (2.04 g, 12 mmol) was added. The reaction mixture was stirred at 100 °C overnight. The medium was adjusted to pH 9–10 by $NaHCO_3$ (saturated aqueous solution), if necessary, and was filtered through a pad of diatomaceous earth. The filtrate was cooled in an ice bath and was carefully acidified by 37% HCl to pH 3–4. The precipitated solid was collected by filtration, washed with cold water then *n*-hexane.

Yield 55%; yellow solid; ^1H NMR (500 MHz, DMSO-d_6) δ 7.66 (d, *J* = 8.8 Hz, 1H), 7.28 (d, *J* = 8.8 Hz, 1H), 4.75 (septet, *J* = 6.0 Hz, 1H), 1.20 (d, *J* = 6.0 Hz, 6H); ^{13}C NMR (126 MHz, DMSO-d_6) δ 171.22, 155.94, 148.28, 138.01, 124.75, 116.98, 112.63, 75.87, 22.08 (2C); m/z (ESI+) 241 $[M]^+$.

Isopropyl 3-isopropoxy-4-nitrobenzoate 21

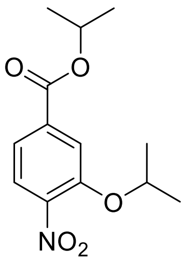

To a stirred mixture of 3-hydroxy-4-nitrobenzoic acid (7.33 g, 40 mmol) and K_2CO_3 (13.8 g, 100 mmol) in DMF (120 mL), 2-bromopropane (14.8 g, 120 mmol) was added. The reaction mixture was stirred at 90 °C overnight. The mixture was poured on to ice cooled water (400 mL) and extracted with EtOAc (3 × 100 mL). The combined organic extract was washed with brine, dried over anhydrous $MgSO_4$, and the solvent was removed by vacuum distillation. The obtained material was used directly in the next step without further purification.

Yield 95%; pale yellow liquid; ^{1}H NMR (300 MHz, $CDCl_3$) δ 7.73 (d, *J* = 8.0 Hz, 1H), 7.71 (d, *J* = 2.0 Hz, 1H), 7.61 (dd, *J* = 8.0, 2.0 Hz, 1H), 5.24 (septet, *J* = 6.3 Hz, 1H), 4.75 (septet, *J* = 6.0 Hz, 1H), 1.39 (d, *J* = 6.0 Hz, 6H), 1.37 (d, *J* = 6.3 Hz, 6H); ^{13}C NMR (75 MHz, $CDCl_3$) δ 164.25, 150.67, 143.56, 135.19, 124.86, 120.92, 116.89, 72.92, 69.54, 21.75 (2C), 21.71 (2C); m/z (ESI+) 268 $[M + H]^+$; t_R = 13.69 min.

3-Isopropoxy-4-nitrobenzoic acid 27

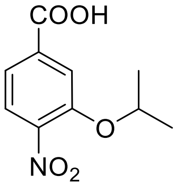

To a stirred solution of **21** (2.67 g, 10 mmol) in MeOH (25 mL), 1 N NaOH (50 mL) was added. The reaction was stirred at at 100 °C for 2 h, then solvent was concentrated *in vacuo*. The remaining residue was diluted with water (25 mL), cooled in an ice bath and acidified by $KHSO_4$ (saturated aqueous solution) to pH 3–4. The precipitate was collected by filtration, washed with water, then *n*-hexane.

Yield 93%; beige solid; ^{1}H NMR (500 MHz, DMSO-d_6) δ 13.62 (br s, 1H), 7.91 (d, *J* = 8.5 Hz, 1H), 7.76 (d, *J* = 1.6 Hz, 1H), 7.61 (dd, *J* = 8.5, 1.6 Hz, 1H), 4.90 (septet, *J* = 6.0 Hz, 1H), 1.29 (d, *J* = 6.0 Hz, 6H); ^{13}C NMR (126 MHz, DMSO-d_6) δ 165.86, 149.61, 143.27, 135.43, 124.88, 121.25, 116.53, 72.45, 21.51 (2C); m/z (ESI+) 226 $[M + H]^+$; t_R = 9.69 min.

2,3-Dihydroxy-4-nitrobenzaldehyde 42

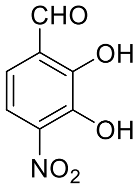

To a stirred solution of **41** (1.2 g, 5 mmol) in DCM (10 mL) cooled at 0 °C in an ice bath, BBr_3 (1 M solution in DCM, 20 mL) was added carefully under a nitrogen atmosphere. The reaction mixture was allowed to warm to room temperature and was further stirred overnight. Solvent was removed *in vacuo*. The residue was cautiously diluted with water (50 mL) and the medium was acidified by 2 N HCl to pH 4–5, if necessary. The mixture was extracted with EtOAc (3 × 30 mL). The combined organic extract was washed with brine, dried over anhydrous $MgSO_4$, and the solvent was removed by vacuum distillation. The residue was dissolved in $CHCl_3$ and purified using flash chromatography (SiO_2, DCM–MeOH = 98:2).

Yield 68%; red crystals; ^{1}H NMR (500 MHz, $CDCl_3$) δ 11.20 (br s, 1H), 10.60 (br s, 1H), 10.04 (s, 1H), 7.75 (d, *J* = 8.8 Hz, 1H), 7.25 (d, *J* = 8.8 Hz, 1H); ^{13}C NMR (126 MHz, $CDCl_3$) δ 195.87, 152.88, 145.63, 136.27, 123.09, 121.42, 114.52; m/z (ESI+) 183 $[M]^+$; t_R = 9.54 min.

2,3-Diisopropoxy-4-nitrobenzaldehyde 43

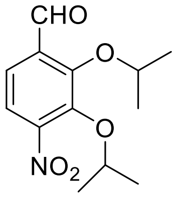

To a stirred mixture of **42** (732 mg, 4 mmol) and K_2CO_3 (1.38 g, 10 mmol) in DMF (20 mL), 2-bromopropane (1.48 g, 12 mmol) was added. The reaction mixture was stirred at 80 °C overnight. Solvent was evaporated *in vacuo*, and the residue was diluted with water (30 mL). The resulting mixture was extracted with EtOAc (3 × 30 mL). The combined organic extract was washed with brine, dried over anhydrous $MgSO_4$, and the solvent was removed by vacuum distillation. The residue was dissolved in toluene and purified using flash chromatography (SiO_2, *n*-hexane–EtOAc = 6:1).

Yield 76%; yellow oil; ^{1}H NMR (500 MHz, $CDCl_3$) δ 10.42 (s, 1H), 7.65 (d, *J* = 8.5 Hz, 1H), 7.48 (d, *J* = 8.5 Hz, 1H), 4.82 (septet, *J* = 6.0 Hz, 1H), 4.70 (septet, *J* = 6.3 Hz, 1H), 1.35 (d, *J* = 6.0 Hz, 6H), 1.31 (d, *J* = 6.3 Hz, 6H); ^{13}C NMR (126 MHz, $CDCl_3$) δ 189.07, 155.51, 150.01, 145.03, 133.69, 122.21, 118.94, 77.56, 77.21, 22.24 (2C), 22.22 (2C); m/z (ESI+) 267 $[M]^+$; t_R = 16.45 min.

2,3-Diisopropoxy-4-nitrobenzoic acid 44

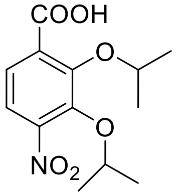

To a stirred solution of **43** (1.07 g, 4 mmol) in acetone (12 mL), $KMnO_4$ (1.26 g, 8 mmol) solution in 50% aq. acetone (28 mL) was added. The reaction mixture was stirred at room temperature for 24 h, then 1 N NaOH (5 mL) was added. The resulting mixture was filtered through a pad of diatomaceous earth, and the filtrate was concentrated *in vacuo*. The residue was cooled in an ice bath and carefully acidified by $KHSO_4$ (saturated aqueous solution) to pH 4–5, then extracted with EtOAc (3 × 25 mL). The combined organic extract was washed with brine, dried over anhydrous $MgSO_4$, and the solvent

was removed by vacuum distillation. The obtained material was triturated with *n*-hexane (25 mL), and collected by filtration.

Yield 90%; beige crystals; ^{1}H NMR (500 MHz, DMSO-d_6) δ 13.43 (br s, 1H), 7.61 (d, *J* = 8.5 Hz, 1H), 7.48 (d, *J* = 8.5 Hz, 1H), 4.68 (septet, *J* = 6.0 Hz, 1H), 4.49 (septet, *J* = 6.3 Hz, 1H), 1.22 (d, *J* = 6.3 Hz, 6H), 1.18 (d, *J* = 6.0 Hz, 6H); ^{13}C NMR (126 MHz, DMSO-d_6) δ 166.37, 150.26, 147.38, 143.72, 132.73, 124.38, 118.36, 76.81, 76.40, 22.04 (2C), 21.93 (2C); m/z (ESI+) 283 $[M]^+$; t_R = 14.09 min.

Methyl 2-hydroxy-4-nitrobenzoate 45

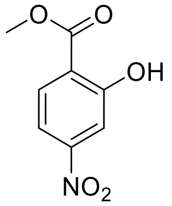

To a stirred solution of 2-hydroxy-4-nitrobenzoic acid **57** (1.10 g, 6 mmol) in MeOH (20 mL), concd H_2SO_4 (2 mL) was added drop wise. The reaction mixture was stirred at 70 °C overnight, then solvent was concentrated *in vacuo*. The residue was diluted with water (25 mL) and neutralized by Na_2CO_3 (saturated aqueous solution) to pH 7–8. The mixture was extracted with EtOAc (3 × 25 mL). The combined organic extract was washed with brine, dried over anhydrous $MgSO_4$, and the solvent was evaporated by vacuum distillation. The obtained material was triturated with *n*-hexane (50 mL), and collected by filtration.

Yield 96%; yellow crystals; ^{1}H NMR (300 MHz, $CDCl_3$) δ 10.98 (br s, 1H), 8.03 (d, *J* = 8.8 Hz, 1H), 7.82 (d, *J* = 2.2 Hz, 1H), 7.71 (dd, *J* = 8.8, 2.2 Hz, 1H), 4.03 (s, 3H); ^{13}C NMR (75 MHz, $CDCl_3$) δ 169.24, 161.99, 152.15, 131.21, 117.14, 113.50, 113.04, 53.09; m/z (ESI+) 198 $[M + H]^+$; t_R = 13.68 min.

Methyl 4-amino-2-hydroxybenzoate 46

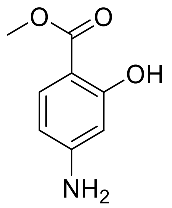

To a stirred solution of **45** (986 mg, 5 mmol) in EtOH (40 mL), iron powder (1.40 g, 25 mmol) was added at 55 °C followed by NH_4Cl (134 mg, 2.5 mmol) solution in water (15 mL). The reaction mixture was stirred at 90 °C for 1 h, then iron was filtered on hot and the filtrate was concentrated *in vacuo*. The residue was diluted with water (25 mL) and basified by $NaHCO_3$ (saturated aqueous solution) to pH 8–9. The mixture was extracted with EtOAc (3 × 25 mL). The combined organic extract was washed with brine, dried over anhydrous $MgSO_4$, and the solvent was removed by vacuum distillation. The obtained material was triturated with *n*-hexane (25 mL), and collected by filtration.

Yield 80%; beige crystals; ^{1}H NMR (300 MHz, $CDCl_3$) δ 10.93 (br s, 1H), 7.62 (d, *J* = 8.9 Hz, 1H), 6.17 (d, *J* = 2.2 Hz, 1H), 6.15 (dd, *J* = 8.9, 2.2 Hz, 1H), 4.10 (br s, 2H), 3.88 (s, 3H); ^{13}C NMR (75 MHz,

$CDCl_3$) δ 170.45, 163.58, 153.33, 131.63, 106.80, 103.05, 100.76, 51.72; m/z (ESI+) 168 $[M + H]^+$; t_R = 9.83 min.

Methyl 4-amino-3-chloro-2-hydroxybenzoate 47

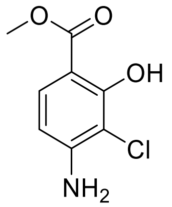

To a stirred solution of **46** (1 g, 6 mmol) in DMF (20 mL), *N*-chlorosuccinimide (801 mg, 6 mmol) solution in DMF (5 mL) was added drop wise. The reaction mixture was stirred at 40 °C overnight, then solvent was removed *in vacuo*. The residue was diluted with water (50 mL) and basified by Na_2CO_3 (saturated aqueous solution) to pH 8–9. The mixture was extracted with EtOAc (3 × 30 mL). The combined organic extract was washed with brine, dried over anhydrous $MgSO_4$, and the solvent was removed by vacuum distillation. The residue was dissolved in $CHCl_3$ and purified using flash chromatography (SiO_2, *n*-hexane–EtOAc = 4:1).

Yield 70%; white crystals; ^{1}H NMR (300 MHz, $CDCl_3$) δ 11.61 (br s, 1H), 7.57 (d, *J* = 8.8 Hz, 1H), 6.28 (d, *J* = 8.8 Hz, 1H), 4.59 (br s, 2H), 3.91 (s, 3H); ^{13}C NMR (75 MHz, $CDCl_3$) δ 170.42, 158.90, 149.26, 128.78, 106.10, 104.93, 103.31, 52.01; m/z (ESI+) 202 $[M + H]^+$; t_R = 13.10 min.

4-Methoxypicolinonitrile 48

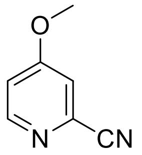

To a stirred solution of 4-methoxypyridine-*N*-oxide hydrate (2.50 g, 20 mmol) in DCM (25 mL), trimethylsilyl cyanide (2.58 g, 26 mmol) was added. The reaction mixture was stirred at room temperature for 10 min, then dimethylcarbamoyl chloride (2.80 g, 26 mmol) was added portion wise, and the reaction was further stirred at room temperature for 24 h. The reaction was quenched carefully with K_2CO_3 10% (25 mL) and allowed to stir for 15 min. The organic layer was separated and aqueous layer was extracted with DCM (2 × 20 mL) then diethyl ether (1 × 20 mL). The combined organic extract was dried over anhydrous $MgSO_4$, and the solvent was removed by vacuum distillation. The residue was triturated with *n*-hexane (50 mL), and collected by filtration.

Yield 70%; white crystals; ^{1}H NMR (300 MHz, $CDCl_3$) δ 8.51 (d, *J* = 5.8 Hz, 1H), 7.22 (d, *J* = 2.5 Hz, 1H), 7.01 (dd, *J* = 5.8, 2.5 Hz, 1H), 3.91 (s, 3H); ^{13}C NMR (75 MHz, $CDCl_3$) δ 165.92, 152.23, 134.99, 117.12, 115.37, 112.61, 55.80; m/z (ESI+) 135 $[M + H]^+$; t_R = 8.27 min.

2-Carboxy-4-methoxypyridin-1-ium chloride 49

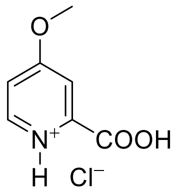

To the picolinonitrile **48** (1.61 g, 12 mmol), 5 N HCl (40 mL) was added. The reaction mixture was stirred at 100 °C overnight, then solvent was evaporated to dryness. The obtained material was triturated with *n*-hexane (50 mL), and collected by filtration.

Yield 95%; white crystals; ^{1}H NMR (300 MHz, DMSO-d_6) δ 9.80 (br s, 1H), 8.71 (d, *J* = 6.5 Hz, 1H), 7.81 (d, *J* = 2.7 Hz, 1H), 7.62 (dd, *J* = 6.5, 2.7 Hz, 1H), 7.47 (t, *J* = 50.0 Hz, 3H), 4.10 (s, 3H); ^{13}C NMR (75 MHz, DMSO-d_6) δ 171.09, 161.45, 145.70, 144.30, 114.16, 112.97, 57.77; m/z (ESI+) 154 [M - Cl]$^+$; t_R = 1.48 min.

4-Hydroxy-5-nitropicolinic acid 50

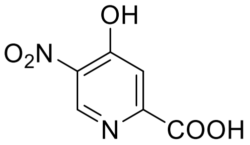

To the picolinic acid hydrchloride salt **49** (3.79 g, 20 mmol), concd H_2SO_4 (8 mL) was carefully added. The mixture was stirred for 5 min then a mixture of concd H_2SO_4 (2 mL) and fuming HNO_3 (10 mL) was added. The reaction mixture was stirred at 150 °C for 48 h, then it was cooled in an ice bath and carefully neutralized with NH_4OH 25% till pH 6–7. The pale yellow precipitate was collected by filtration, washed with cold water and *n*-hexane. Filtrate was extracted with THF (3 × 30 mL). The combined organic extract was dried over anhydrous $MgSO_4$, and the solvent was removed by vacuum distillation. The residue was triturated with *n*-hexane (50 mL), and filtered to afford a second crop of the product.

Yield 50%; pale yellow solid; ^{1}H NMR (500 MHz, DMSO-d_6) δ 8.50 (s, 1H), 7.56 (br s, 2H), 6.77 (s, 1H); ^{13}C NMR (126 MHz, DMSO-d_6) δ 169.66, 161.54, 145.32, 139.14, 137.77, 120.95; m/z (ESI+) 185 [M + H]$^+$; t_R = 1.16 min.

Isopropyl 4-isopropoxy-5-nitropicolinate 51

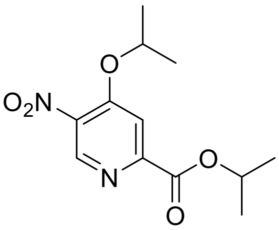

To a stirred mixture of **50** (921 mg, 5 mmol) and K_2CO_3 (1.38 g, 10 mmol) in DMF (25 mL), 2-bromopropane (1.84 g, 15 mmol) was added. The reaction mixture was stirred at 90 °C overnight. Solvent was evaporated *in vacuo*, and the residue was diluated with water (30 mL). The resulting mixture was extracted with EtOAc (3 × 30 mL). The combined organic extract was washed with brine, dried

over anhydrous $MgSO_4$, and the solvent was removed by vacuum distillation. The residue was dissolved in toluene and purified using flash chromatography (SiO_2, *n*-hexane–EtOAc = 1:1).

Yield 75%; pale yellow crystals; ^{1}H NMR (500 MHz, $CDCl_3$) δ 8.93 (s, 1H), 7.77 (s, 1H), 5.28 (septet, *J* = 6.3 Hz, 1H), 4.88 (septet, *J* = 6.0 Hz, 1H), 1.42 (d, *J* = 6.0 Hz, 6H), 1.38 (d, *J* = 6.3 Hz, 6H); ^{13}C NMR (126 MHz, $CDCl_3$) δ 163.24, 157.82, 152.31, 146.56, 138.47, 111.21, 73.77, 70.58, 21.56 (2C), 21.39 (2C); m/z (ESI+) 269 $[M + H]^+$; t_R = 15.57 min.

4-Isopropoxy-5-nitropicolinic acid 52

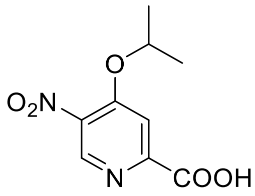

To a stirred solution of **51** (536 mg, 2 mmol) in MeOH (10 mL), 1 N NaOH (5 mL) was added. The reaction was stirred at room temperature overnight. Solvent was evaporated *in vacuo*. The remaining residue was dissolved in water (15 mL), cooled in an ice bath and acidified by $KHSO_4$ (saturated aqueous solution) to pH 6, then extracted with EtOAc/THF (1:1, 3 × 30 mL). The combined organic extract was washed with brine, dried over anhydrous $MgSO_4$, and the solvent was removed by vacuum distillation. The obtained material was triturated with *n*-hexane (30 mL), and collected by filtration.

Yield 85%; beige solid; ^{1}H NMR (500 MHz, DMSO-d_6) δ 13.76 (br s, 1H), 9.03 (s, 1H), 7.90 (s, 1H), 5.13 (septet, *J* = 6.0 Hz, 1H), 1.35 (d, *J* = 6.0 Hz, 6H); ^{13}C NMR (126 MHz, DMSO-d_6) δ 164.97, 157.17, 152.76, 146.07, 138.42, 111.53, 73.62, 21.28 (2C); m/z (ESI+) 227 $[M + H]^+$; t_R = 12.88 min.

6-Chloro-2-isopropoxy-3-nitropyridine 53

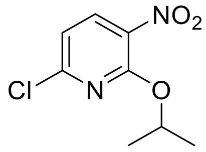

To a stirred solution of 2,6-dichloro-3-nitropyridine (3.86 g, 20 mmol) in toluene (30 mL) cooled at 0 °C in an ice bath, 2-propanol (1.44 g, 24 mmol) was added. The reaction mixture was stirred at 0 °C for 15 min, then NaH (50–60% in mineral oil, 1.22 g, 28 mmol) was added portion wise under a nitrogen atmosphere, and the reaction was further stirred at room temperature overnight. The reaction was quenched with brine, then diluted with water and extracted with EtOAc (3 × 30 mL). The combined organic extract was dried over anhydrous $MgSO_4$, and the solvent was removed by vacuum distillation. The residue was dissolved in toluene and purified using flash chromatography (SiO_2, *n*-hexane–EtOAc = 5:1).

Yield 70%; yellowish white crystals; ^{1}H NMR (300 MHz, $CDCl_3$) δ 8.22 (d, *J* = 8.2 Hz, 1H), 6.98 (d, *J* = 8.2 Hz, 1H), 5.51 (septet, *J* = 6.2 Hz, 1H), 1.44 (d, *J* = 6.2 Hz, 6H); ^{13}C NMR (75 MHz, $CDCl_3$) δ 155.71, 152.72, 137.33, 132.66, 115.88, 72.48, 21.71 (2C); m/z (ESI+) 217 $[M + H]^+$; t_R = 12.82 min.

2-Isopropoxy-3-nitro-6-vinylpyridine 54

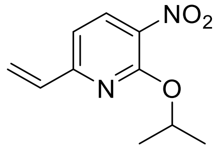

To a stirred solution of **53** (650 mg, 3 mmol), and tributyl(vinyl)tin (1.0 g, 3.15 mmol) in toluene (20 mL) under a nitrogen atmosphere, tetrakis(triphenylphosphine) palladium(0) (175 mg, 0.15 mmol) was added. The reaction mixture was stirred at 110 °C overnight. The reaction was quenched with brine, then extracted with EtOAc (3 × 25 mL). The combined organic extract was dried over anhydrous $MgSO_4$, and the solvent was removed by vacuum distillation. The obtained material was purified using flash chromatography (SiO_2, *n*-hexane–EtOAc = 6:1).

Yield 90%; yellow oil; ^{1}H NMR (500 MHz, $CDCl_3$) δ 8.22 (d, *J* = 8.2 Hz, 1H), 6.90 (d, *J* = 8.2 Hz, 1H), 6.73 (dd, *J* = 17.3, 10.7 Hz, 1H); 6.38 (dd, *J* = 17.3, 1.6 Hz, 1H); 5.63 (dd, *J* = 10.7, 1.6 Hz, 1H); 5.58 (septet, *J* = 6.3 Hz, 1H), 1.44 (d, *J* = 6.3 Hz, 6H); ^{13}C NMR (126 MHz, $CDCl_3$) δ 157.40, 155.57, 135.88, 134.92, 132.83, 122.37, 113.86, 70.67, 21.79 (2C); m/z (ESI+) 208 $[M]^+$; t_R = 13.37 min.

6-isopropoxy-5-nitropicolinic acid 55

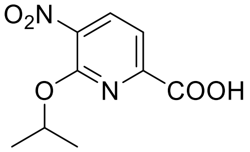

To a stirred solution of **54** (625 mg, 3 mmol) in acetone (10 mL), $KMnO_4$ (1.90 g, 12 mmol) solution in 50% aq. acetone (50 mL) was added. The reaction mixture was stirred at room temperature for 24 h, then 1 N NaOH (3 mL) was added. The resulting mixture was filtered through a pad of diatomaceous earth, and the filtrate was concentrated *in vacuo*. The residue was cooled in an ice bath and carefully acidified by $KHSO_4$ (saturated aqueous solution) to pH 4–5, then extracted with EtOAc (3 × 25 mL). The combined organic extract was washed with brine, dried over anhydrous $MgSO_4$, and the solvent was removed by vacuum distillation. The obtained material was triturated with *n*-hexane (25 mL), and collected by filtration.

Yield 75%; beige crystals; ^{1}H NMR (500 MHz, DMSO-d_6) δ 13.64 (br s, 1H), 8.50 (d, *J* = 7.9 Hz, 1H), 7.75 (d, *J* = 7.9 Hz, 1H), 5.52 (septet, *J* = 6.0 Hz, 1H), 1.35 (d, *J* = 6.0 Hz, 6H); ^{13}C NMR (126 MHz, DMSO-d_6) δ 164.37, 154.12, 149.14, 136.17, 136.09, 117.71, 71.00, 21.51 (2C); m/z (ESI+) 227 $[M + H]^+$; t_R = 9.01 min.

The first amide coupling strategy (Scheme S2) started with coupling of the *N*-protected middle rings **20**, **27**, **30**, **44**, **56**, and **57** to the *C*-protected *C*-terminal ring **22** using either dichlorotriphenylphosphorane or phosphorus trichloride as coupling reagent to afford the dipeptides **23**, and **58**–**62**. The nitro derivatives **23**, and **58**–**62** were chemically reduced to the corresponding amines **24**, and **63**–**67**. A second coupling cycle of the dipeptides **24**, and **63**–**67** to the *N*-terminal rings **57**, **68**, and **69** was performed to furnish the tripeptides **25**, and **70**–**76**. Compound **77** was obtained via alkylation of **71** with isopropyl bromide in K_2CO_3/DMF mixture. Further reduction of the nitro derivatives **25**, **70**, and **72**–**77** produced the amino esters **26**, and **78**–**84**. Finally, *C*-deprotection via ester hydrolysis yielded the amino acids **2**, **4**, **6**, **7**, and **10**–**13**. The nitro acids **14** and **15** were prepared using the same strategy where the tripeptide nitro esters **75** and **76**, respectively were saponified.

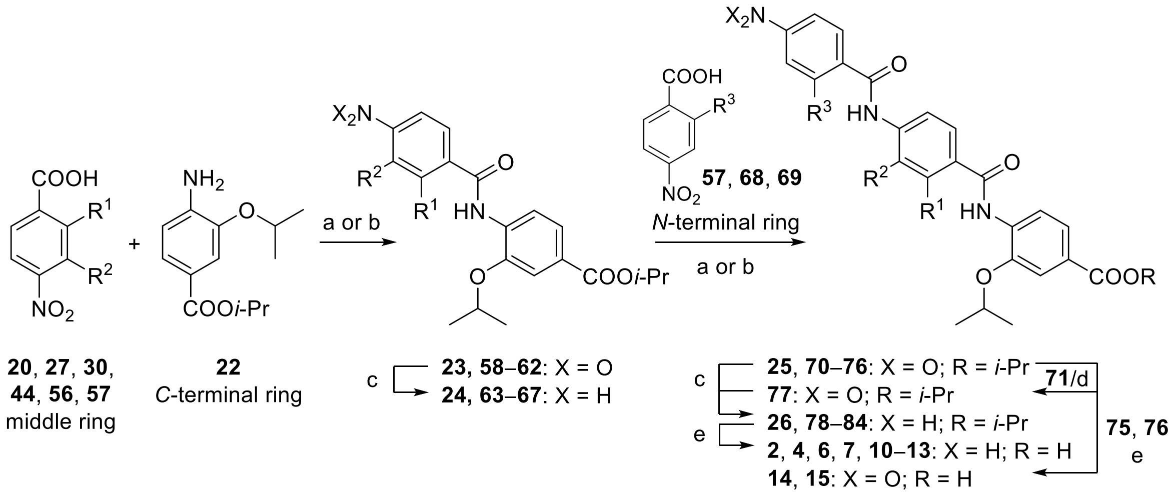

Compound	R^1	R^2
**20**, **23**, **24**	OH	O*i*-Pr
**30**, **58**, **63**	OH	OMe
**56**, **59**, **64**	OMe	OH
**44**, **60**, **65**	O*i*-Pr	O*i*-Pr
**57**, **61**, **66**	OH	H
**27**, **62**, **67**	H	O*i*-Pr

Compound	R^3
**57**	OH
**68**	H
**69**	O*i*-Pr

Compound	R^1	R^2	R^3
**2**, **25**, **26**	OH	O*i*-Pr	H
**4**, **70**, **78**	OH	OMe	H
**71**	OMe	OH	H
**6**, **77**, **79**	OMe	O*i*-Pr	H
**7**, **72**, **80**	O*i*-Pr	O*i*-Pr	H
**10**, **73**, **81**	OH	H	H
**11**, **74**, **82**	H	O*i*-Pr	H
**12**, **14**, **75**, **83**	H	O*i*-Pr	OH
**13**, **15**, **76**, **84**	OH	H	O*i*-Pr

Scheme S2. First coupling strategy for synthesizing cystobactamid 507 (**2**), **4**, **6**, **7**, **10**–**15**, and the corresponding esters. Reagents and conditions: (a) Cl_2PPh_3, $CHCl_3$, 80 °C, 12 h; (b) PCl_3, xylenes, 150 °C, 12 h; (c) Fe, NH_4Cl, EtOH/H_2O, 90 °C, 1 h; (d) 2-bromopropane, K_2CO_3, DMF, 80 °C, 12 h; (e) 1 N NaOH, MeOH/THF, rt, 12 h.

The second strategy (Scheme S3) started with coupling of the *C*-protected middle ring **47** to the *N*-protected *N*-terminal ring **68** via dichlorotriphenylphosphorane to yield the dipeptide **85**. Ester hydrolysis of **85** afforded the corresponding acid **86**, which was coupled to the *C*-terminal ring **22** using the same coupling reagent to produce the tripeptide **87**. Reduction of **87** to the corresponding amine **88** and final ester saponification afforded the amino acid **5**.

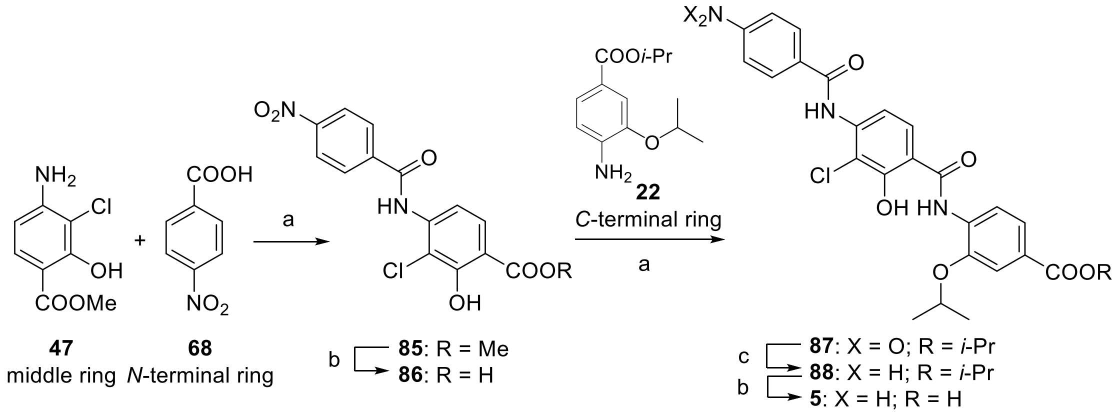

Scheme S3. Second coupling strategy for synthesizing compound **5**. Reagents and conditions: (a) Cl_2PPh_3, $CHCl_3$, 80 °C, 12 h; (b) 1 N NaOH, MeOH/THF, rt, 12 h; (c) Fe, NH_4Cl, EtOH/H_2O, 90 °C, 1 h.

Synthesis of the pyridine containing derivatives **8** and **9** (Scheme S4) was similar to the first strategy. However, as the picolinic acids **52** and **55** did not contain hydroxyl group, we used less selective coupling reagents. The first coupling was achieved using EDC/HOBt, whereas the second coupling was carried out via acylation of the amino dipeptides **91** and **92** with *p*-nitrobenzoyl chloride to give the tripeptides **93** and **94**, respectively. Noteworthy, the coupling reagent dichlorotriphenyl-phosphorane was also tried, and efficiently produced the target amides in good to excellent yields.

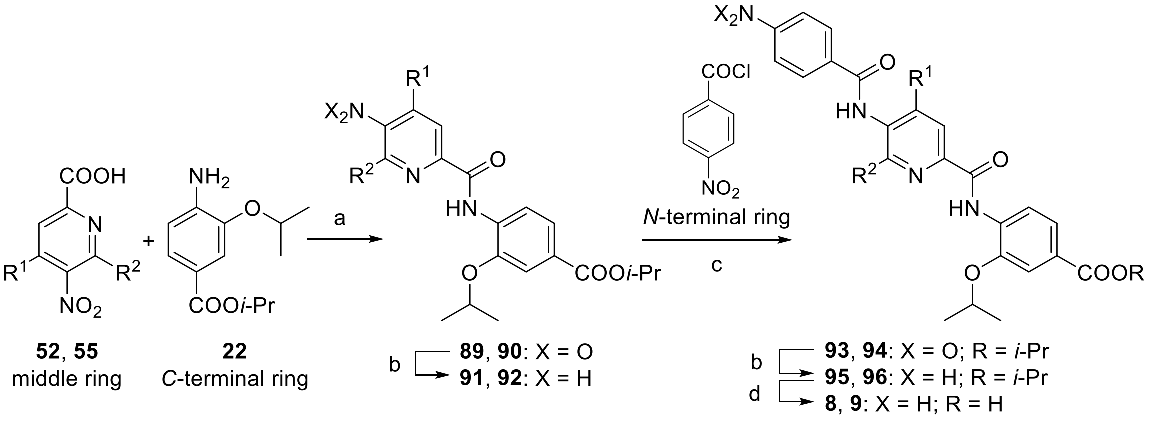

Compound	R^1	R^2
**8**, **52**, **89**, **91**, **93**, **95**	O*i*-Pr	H
**9**, **55**, **90**, **92**, **94**, **96**	H	O*i*-Pr

Scheme S4. Synthesis of the pyridine containing tripeptides **8** and **9**. Reagents and conditions: (a) EDC/HOBt, DMF, $CHCl_3$, 0 °C–rt, 12 h; (b) Fe, NH_4Cl, EtOH/H_2O, 90 °C, 1 h; (c) DCM, pyridine, rt, 24 h; (d) 1 N NaOH, MeOH/THF, rt, 12 h.

Isopropyl 4-amino-3-isopropoxybenzoate 22

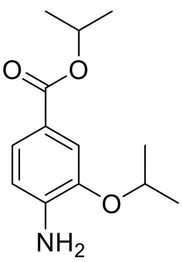

To a stirred solution of **21** (2.67 g, 10 mmol) in EtOH (60 mL), iron powder (2.80 g, 50 mmol) was added at 55 °C followed by NH_4Cl (266 mg, 5 mmol) solution in water (30 mL). The reaction mixture was stirred at 90 °C for 1 h, then iron was filtered on hot and the filtrate was concentrated *in vacuo*. The residue was diluted with water (30 mL) and basified by $NaHCO_3$ (saturated aqueous solution) to pH 8–9. The mixture was extracted with EtOAc (3 × 30 mL). The combined organic extract was washed with brine, dried over anhydrous $MgSO_4$, and the solvent was removed by vacuum distillation. The obtained material was used directly in the next step without further purification.

Yield 90%; pale green liquid; ^{1}H NMR (300 MHz, $CDCl_3$) δ 7.54 (dd, *J* = 8.1, 2.0 Hz, 1H), 7.50 (d, *J* = 2.0 Hz, 1H), 6.80 (d, *J* = 8.1 Hz, 1H), 5.21 (septet, *J* = 6.2 Hz, 1H), 4.82 (br s, 2H), 4.65 (septet, *J* = 6.0 Hz, 1H), 1.38 (d, *J* = 6.0 Hz, 6H), 1.35 (d, *J* = 6.2 Hz, 6H); ^{13}C NMR (75 MHz, $CDCl_3$) δ 166.22, 144.94, 139.89, 123.49, 121.73, 114.62, 114.16, 71.00, 67.72, 22.12 (2C), 21.99 (2C); m/z (ESI+) 238 $[M + H]^+$; t_R = 11.62 min.

2-Isopropoxy-4-nitrobenzoic acid 69

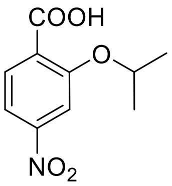

Synthesis of **69** was performed similarly as described for **27** starting with the carboxylic acid **57**.

Yield 87%; beige solid; ^{1}H NMR (500 MHz, DMSO-d_6) δ 12.85 (br s, 1H), 7.84 (d, *J* = 2.1 Hz, 1H), 7.80 (dd, *J* = 8.4, 2.1 Hz, 1H), 7.76 (d, *J* = 8.4 Hz, 1H), 4.83 (septet, *J* = 6.0 Hz, 1H), 1.30 (d, *J* = 6.0 Hz, 6H); ^{13}C NMR (126 MHz, DMSO-d_6) δ 166.60, 155.94, 149.68, 130.70, 129.73, 114.99, 109.38, 71.84, 21.54 (2C); m/z (ESI+) 226 $[M + H]^+$; t_R = 9.48 min.

General procedure for amide coupling using dichlorotriphenylphosphorane

To a stirred solution of the *N*-protected carboxylic acid (1 mmol) and the *C*-protected amine (1 mmol) in anhydrous $CHCl_3$ (50 mL) under a nitrogen atmosphere, dichlorotriphenylphosphorane (1.5 g, 4.5 mmol) was added. The reaction mixture was heated at 80 °C overnight. Solvent was removed by vacuum distillation. The residue was dissolved in toluene and purified using flash chromatography (SiO_2, *n*-hexane–EtOAc = 4:1 or 2:1 or 1:1).

General procedure for amide coupling using phosphorus trichloride

To a stirred solution of the *N*-protected carboxylic acid (1 mmol) in a mixture of xylenes (30 mL) and DCM (5 mL), the *C*-protected amine (1 mmol) was added. The reaction was warmed to 60 °C then phosphorus trichloride (0.05 mL, 0.5 mmol) was added. The reaction mixture was heated at 150 °C overnight. Solvent was removed by vacuum distillation. The residue was dissolved in MeOH and mixed with silica gel and the resulting paste was dried *in vacuo*. The silica adsorbed material was purified using flash chromatography (SiO_2, *n*-hexane–EtOAc = 1:1).

Isopropyl 4-(2-hydroxy-3-isopropoxy-4-nitrobenzamido)-3-isopropoxybenzoate 23

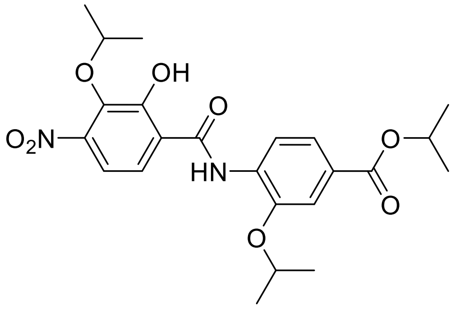

Yield 87%; yellow solid; ^{1}H NMR (500 MHz, $CDCl_3$) δ 11.86 (br s, 1H), 9.20 (br s, 1H), 8.51 (d, *J* = 8.5 Hz, 1H), 7.72 (dd, *J* = 8.5, 1.9 Hz, 1H), 7.62 (d, *J* = 1.9 Hz, 1H), 7.37 (d, *J* = 8.8 Hz, 1H), 7.29 (d, *J* = 8.8 Hz, 1H), 5.26 (septet, *J* = 6.3 Hz, 1H), 4.85 (septet, *J* = 6.0 Hz, 1H), 4.80 (septet, *J* = 6.0 Hz, 1H), 1.46 (d, *J* = 6.0 Hz, 6H), 1.39 (d, *J* = 6.3 Hz, 6H), 1.36 (d, *J* = 6.0 Hz, 6H); ^{13}C NMR (126 MHz, $CDCl_3$) δ 166.02, 165.56, 156.26, 147.20, 146.15, 140.59, 131.15, 127.14, 122.95, 120.00, 119.37, 118.88, 113.73, 113.19, 77.59, 72.02, 68.61, 22.37 (2C), 22.17 (2C), 21.94 (2C); m/z (ESI+) 461 $[M + H]^+$.

Isopropyl 4-(2-hydroxy-3-methoxy-4-nitrobenzamido)-3-isopropoxybenzoate 58

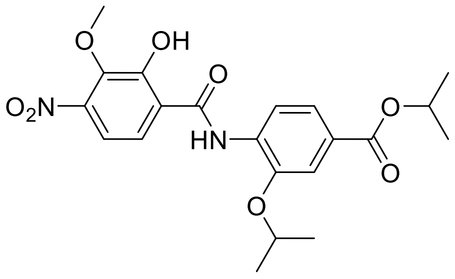

Yield 85%; yellow solid; ^{1}H NMR (500 MHz, $CDCl_3$) δ 12.14 (br s, 1H), 9.12 (br s, 1H), 8.50 (d, *J* = 8.5 Hz, 1H), 7.73 (dd, *J* = 8.5, 1.6 Hz, 1H), 7.63 (d, *J* = 1.6 Hz, 1H), 7.37 (d, *J* = 8.8 Hz, 1H), 7.32 (d, *J* = 8.8 Hz, 1H), 5.26 (septet, *J* = 6.3 Hz, 1H), 4.81 (septet, *J* = 6.0 Hz, 1H), 4.10 (s, 3H), 1.47 (d, *J* = 6.0 Hz, 6H), 1.39 (d, *J* = 6.3 Hz, 6H); ^{13}C NMR (126 MHz, $CDCl_3$) δ 166.14, 165.52, 156.70, 146.68, 146.16, 142.93, 130.94, 127.28, 122.93, 120.19, 119.40, 119.16, 113.64, 113.18, 72.06, 68.64, 62.01, 22.18 (2C), 21.94 (2C); m/z (ESI+) 433 $[M + H]^+$; t_R = 16.84 min.

Isopropyl 4-(3-hydroxy-2-methoxy-4-nitrobenzamido)-3-isopropoxybenzoate 59

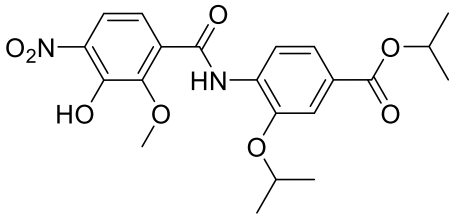

Yield 68%; yellow solid; ^{1}H NMR (500 MHz, DMSO-d_6) δ 10.99 (br s, 1H), 10.72 (s, 1H), 8.56 (d, *J* = 8.4 Hz, 1H), 7.81 (d, *J* = 9.0 Hz, 1H), 7.60 (dd, *J* = 8.4, 1.7 Hz, 1H), 7.58 (m, 2H), 5.12 (septet, *J* = 6.3 Hz, 1H), 4.85 (septet, *J* = 6.0 Hz, 1H), 3.99 (s, 3H), 1.39 (d, *J* = 6.0 Hz, 6H), 1.32 (d, *J* = 6.3 Hz, 6H); ^{13}C NMR (126 MHz, DMSO-d_6) δ 164.79, 161.19, 148.52, 146.16, 146.07, 139.79, 132.53, 130.59, 125.72, 122.22, 119.87, 119.82, 119.29, 112.95, 71.25, 68.10, 62.21, 21.67 (2C), 21.61 (2C); m/z (ESI+) 433 [M + H]$^+$; t_R = 16.85 min.

Isopropyl 4-(2,3-diisopropoxy-4-nitrobenzamido)-3-isopropoxybenzoate 60

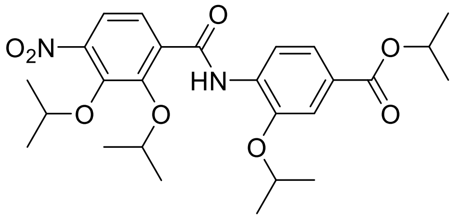

Yield 70%; yellowish orange solid; ^{1}H NMR (500 MHz, DMSO-d_6) δ 10.22 (s, 1H), 8.54 (d, *J* = 8.5 Hz, 1H), 7.78 (s, 2H), 7.61 (m, 2H), 5.13 (septet, *J* = 6.2 Hz, 1H), 4.78 (septet, *J* = 6.1 Hz, 1H), 4.68 (septet, *J* = 6.3 Hz, 1H), 4.59 (septet, *J* = 6.0 Hz, 1H), 1.34 (d, *J* = 6.0 Hz, 6H), 1.32 (d, *J* = 6.3 Hz, 6H), 1.27 (d, *J* = 6.1 Hz, 6H), 1.25 (d, *J* = 6.2 Hz, 6H); ^{13}C NMR (126 MHz, DMSO-d_6) δ 164.75, 161.91, 149.68, 147.63, 146.13, 143.89, 133.18, 132.42, 125.84, 125.22, 122.49, 119.44, 119.17, 113.73, 78.32, 77.38, 72.01, 68.12, 22.02 (2C), 21.88 (2C), 21.70 (2C), 21.68 (2C); m/z (ESI+) 503 [M + H]$^+$; t_R = 17.52 min.

Isopropyl 4-(2-hydroxy-4-nitrobenzamido)-3-isopropoxybenzoate 61

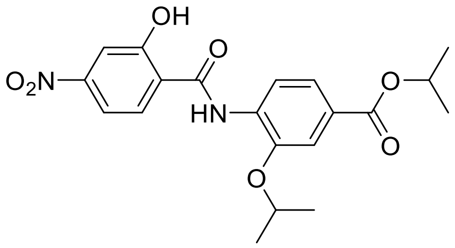

Yield 55%; yellow solid; ^{1}H NMR (500 MHz, DMSO-d_6) δ 12.73 (br s, 1H), 11.20 (s, 1H), 8.62 (d, *J* = 8.5 Hz, 1H), 8.26 (d, *J* = 8.7 Hz, 1H), 7.86 (d, *J* = 2.2 Hz, 1H), 7.80 (dd, *J* = 8.7, 2.3 Hz, 1H), 7.59 (dd, *J* = 8.5, 1.7 Hz, 1H), 7.56 (d, *J* = 1.7 Hz, 1H), 5.12 (septet, *J* = 6.3 Hz, 1H), 4.80 (septet, *J* = 6.0 Hz, 1H), 1.38 (d, *J* = 6.0 Hz, 6H), 1.32 (d, *J* = 6.3 Hz, 6H); ^{13}C NMR (126 MHz, DMSO-d_6) δ 164.79, 161.32, 156.36, 150.01, 145.89, 133.28, 132.79, 125.36, 124.35, 122.51, 118.86, 114.03, 113.39, 111.69, 71.67, 68.03, 21.74 (2C), 21.67 (2C); m/z (ESI+) 403 [M + H]$^+$; t_R = 14.18 min.

Isopropyl 3-isopropoxy-4-(3-isopropoxy-4-nitrobenzamido)benzoate 62

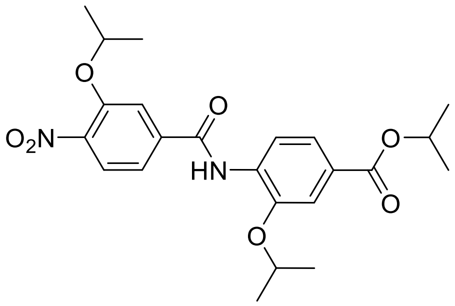

Yield 98%; yellow solid; ^{1}H NMR (500 MHz, $CDCl_3$) δ 8.79 (br s, 1H), 8.56 (d, *J* = 8.5 Hz, 1H), 7.85 (d, *J* = 8.5 Hz, 1H), 7.71 (d, *J* = 8.2 Hz, 1H), 7.68 (s, 1H), 7.60 (s, 1H), 7.35 (d, *J* = 8.2 Hz, 1H), 5.24 (septet, *J* = 6.3 Hz, 1H), 4.80 (septet, *J* = 6.0 Hz, 1H), 4.78 (septet, *J* = 6.0 Hz, 1H), 1.43 (d, *J* = 6.0 Hz, 12H), 1.38 (d, *J* = 6.3 Hz, 6H); ^{13}C NMR (126 MHz, $CDCl_3$) δ 165.58, 163.05, 151.46, 145.81, 142.79, 139.47, 131.97, 126.58, 125.67, 122.99, 118.72, 117.19, 115.37, 113.08, 73.05, 71.84, 68.44, 22.13 (2C), 21.90 (2C), 21.76 (2C); m/z (ESI+) 445 $[M + H]^+$; t_R = 15.79 min.

Isopropyl 4-(2-hydroxy-3-isopropoxy-4-(4-nitrobenzamido)benzamido)-3-isopropoxybenzoate 25

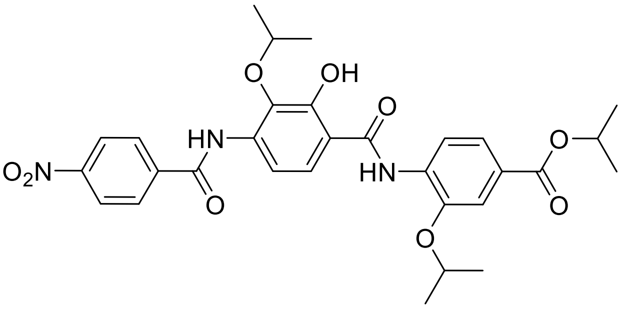

Yield 62%; yellow solid; ^{1}H NMR (500 MHz, $CDCl_3$) δ 12.47 (br s, 1H), 8.98 (br s, 1H), 8.94 (br s, 1H), 8.50 (d, *J* = 8.5 Hz, 1H), 8.41 (d, *J* = 8.8 Hz, 2H), 8.18 (d, *J* = 8.8 Hz, 1H), 8.08 (d, *J* = 8.8 Hz, 2H), 7.72 (dd, *J* = 8.5, 1.6 Hz, 1H), 7.62 (d, *J* = 1.6 Hz, 1H), 7.30 (d, *J* = 8.8 Hz, 1H), 5.26 (septet, *J* = 6.3 Hz, 1H), 4.94 (septet, *J* = 6.3 Hz, 1H), 4.80 (septet, *J* = 6.0 Hz, 1H), 1.48 (d, *J* = 6.0 Hz, 6H), 1.39 (d, *J* = 6.3 Hz, 12H); ^{13}C NMR (126 MHz, $CDCl_3$) δ 167.90, 165.65, 163.05, 154.91, 149.97, 146.04, 140.02, 136.51, 134.90, 131.60, 128.16 (2C), 126.58, 124.23 (2C), 123.01, 120.50, 119.04, 113.25, 112.04, 109.80, 75.43, 72.08, 68.49, 22.91 (2C), 22.21 (2C), 21.95 (2C); m/z (ESI+) 580 $[M + H]^+$.

Isopropyl 4-(2-hydroxy-3-methoxy-4-(4-nitrobenzamido)benzamido)-3-isopropoxybenzoate 70

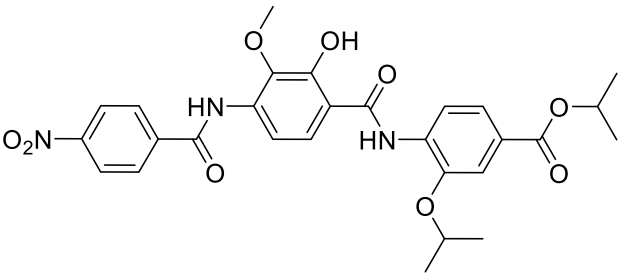

Yield 94%; yellow crystals; ^{1}H NMR (500 MHz, $CDCl_3$) δ 12.48 (br s, 1H), 8.97 (br s, 1H), 8.81 (br s, 1H), 8.50 (d, *J* = 8.5 Hz, 1H), 8.40 (d, *J* = 9.1 Hz, 2H), 8.17 (d, *J* = 8.8 Hz, 1H), 8.08 (d, *J* = 9.1 Hz, 2H), 7.72 (dd, *J* = 8.5, 1.9 Hz, 1H), 7.62 (d, *J* = 1.9 Hz, 1H), 7.31 (d, *J* = 8.8 Hz, 1H), 5.26 (septet, *J* =

6.3 Hz, 1H), 4.80 (septet, J = 6.0 Hz, 1H), 4.10 (s, 3H), 1.48 (d, J = 6.0 Hz, 6H), 1.39 (d, J = 6.3 Hz, 6H); ^{13}C NMR (126 MHz, $CDCl_3$) δ 167.79, 165.65, 163.42, 154.84, 150.01, 146.06, 139.98, 136.99, 135.63, 131.55, 128.37 (2C), 126.65, 124.19 (2C), 123.01, 120.84, 119.07, 113.27, 112.21, 109.94, 72.10, 68.52, 60.91, 22.21 (2C), 21.96 (2C); ^{1}H NMR (500 MHz, DMSO-d_6) δ 11.48 (br s, 1H), 11.07 (br s, 1H), 10.26 (br s, 1H), 8.61 (d, J = 8.5 Hz, 1H), 8.38 (d, J = 8.8 Hz, 2H), 8.19 (d, J = 8.8 Hz, 2H), 7.85 (d, J = 8.8 Hz, 1H), 7.60 (dd, J = 8.5, 1.6 Hz, 1H), 7.58 (d, J = 1.6 Hz, 1H), 7.52 (d, J = 8.8 Hz, 1H), 5.12 (septet, J = 6.0 Hz, 1H), 4.78 (septet, J = 6.0 Hz, 1H), 3.77 (s, 3H), 1.40 (d, J = 6.0 Hz, 6H), 1.32 (d, J = 6.0 Hz, 6H); ^{13}C NMR (126 MHz, DMSO-d_6) δ 164.91, 164.43, 162.97, 149.73, 149.30, 146.12, 141.00, 140.13, 135.25, 133.77, 129.45 (2C), 125.64, 125.02, 123.64 (2C), 122.53, 119.18, 117.22, 115.49, 113.62, 71.94, 68.02, 60.59, 21.73 (2C), 21.72 (2C); m/z (ESI+) 552 $[M + H]^+$; t_R = 16.56 min.

Isopropyl 4-(3-hydroxy-2-methoxy-4-(4-nitrobenzamido)benzamido)-3-isopropoxybenzoate 71

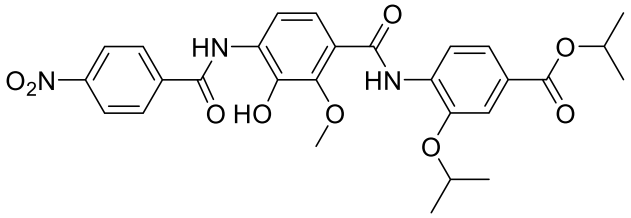

Yield 63%; yellow solid; ^{1}H NMR (500 MHz, DMSO-d_6) δ 10.87 (s, 1H), 10.21 (br s, 1H), 9.81 (br s, 1H), 8.66 (d, J = 8.4 Hz, 1H), 8.39 (m, 2H), 8.23 (m, 2H), 7.61 (m, 3H), 7.58 (d, J = 1.8 Hz, 1H), 5.13 (septet, J = 6.3 Hz, 1H), 4.87 (septet, J = 6.0 Hz, 1H), 3.98 (s, 3H), 1.41 (d, J = 6.0 Hz, 6H), 1.33 (d, J = 6.3 Hz, 6H); ^{13}C NMR (126 MHz, DMSO-d_6) δ 164.87, 164.24, 162.18, 149.32, 147.44, 145.69, 143.03, 139.80, 133.22, 131.04, 129.40 (2C), 125.01, 123.58 (2C), 122.46, 122.36, 120.83, 120.17, 118.79, 112.86, 71.18, 68.01, 61.75, 21.69 (2C), 21.67 (2C); m/z (ESI+) 552 $[M + H]^+$; t_R = 16.53 min.

Isopropyl 4-(2,3-diisopropoxy-4-(4-nitrobenzamido)benzamido)-3-isopropoxybenzoate 72

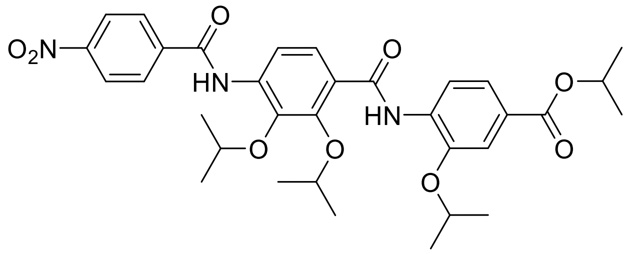

Yield 63%; yellow solid; ^{1}H NMR (500 MHz, DMSO-d_6) δ 10.39 (br s, 1H), 10.15 (br s, 1H), 8.61 (d, J = 8.5 Hz, 1H), 8.39 (d, J = 8.8 Hz, 2H), 8.21 (d, J = 8.8 Hz, 2H), 7.79 (d, J = 8.8 Hz, 1H), 7.78 (d, J = 8.8 Hz, 1H), 7.62 (dd, J = 8.5, 1.9 Hz, 1H), 7.59 (d, J = 1.9 Hz, 1H), 5.13 (septet, J = 6.3 Hz, 1H), 4.76 (septet, J = 6.0 Hz, 1H), 4.63 (septet, J = 6.3 Hz, 1H), 4.47 (septet, J = 6.3 Hz, 1H), 1.35 (d, J = 6.0 Hz, 6H), 1.33 (d, J = 6.3 Hz, 6H), 1.28 (d, J = 6.3 Hz, 6H), 1.27 (d, J = 6.3 Hz, 6H); ^{13}C NMR (126 MHz, DMSO-d_6) δ 164.78, 163.81, 162.70, 149.35, 149.09, 145.82, 143.60, 139.69, 136.49, 133.09, 129.14 (2C), 125.53, 125.22, 125.19, 123.74 (2C), 122.65, 120.25, 118.71, 113.87, 77.31, 76.02, 72.23, 68.00, 22.21 (2C), 21.89 (2C), 21.76 (2C), 21.68 (2C); m/z (ESI+) 622 $[M + H]^+$; t_R = 17.56 min.

Isopropyl 4-(2-hydroxy-4-(4-nitrobenzamido)benzamido)-3-isopropoxybenzoate 73

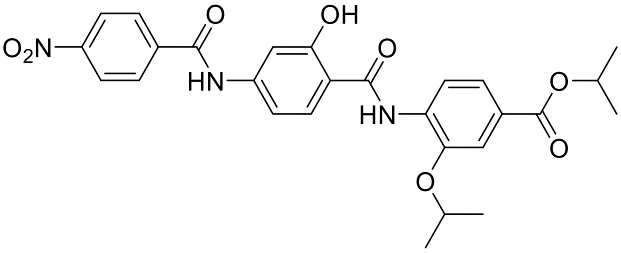

Yield 50%; yellow solid; ^{1}H NMR (500 MHz, DMSO-d$_6$) δ 11.82 (s, 1H), 11.18 (s, 1H), 10.78 (s, 1H), 8.65 (d, *J* = 8.5 Hz, 1H), 8.38 (m, 2H), 8.19 (m, 2H), 8.03 (d, *J* = 8.7 Hz, 1H), 7.90 (d, *J* = 2.0 Hz, 1H), 7.58 (m, 2H), 7.29 (dd, *J* = 8.8, 2.0 Hz, 1H), 5.12 (septet, *J* = 6.2 Hz, 1H), 4.78 (septet, *J* = 6.0 Hz, 1H), 1.38 (d, *J* = 6.0 Hz, 6H), 1.32 (d, *J* = 6.2 Hz, 6H); ^{13}C NMR (126 MHz, DMSO-d$_6$) δ 164.91, 164.48, 162.82, 156.51, 149.26, 145.68, 143.30, 140.38, 134.18, 131.68, 129.39 (2C), 124.53, 123.55 (2C), 122.61, 118.49, 114.55, 113.45, 111.81, 107.42, 71.63, 67.90, 21.74 (2C), 21.70 (2C); m/z (ESI+) 522 [M + H]$^+$; t$_R$ = 13.95 min.

Isopropyl 3-isopropoxy-4-(3-isopropoxy-4-(4-nitrobenzamido)benzamido)benzoate 74

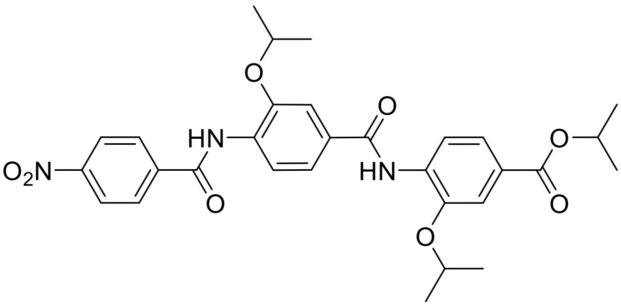

Yield 93%; yellow solid; ^{1}H NMR (500 MHz, CDCl$_3$) δ 8.88 (br s, 1H), 8.81 (br s, 1H), 8.68 (d, *J* = 8.5 Hz, 1H), 8.62 (d, *J* = 8.5 Hz, 1H), 8.41 (d, *J* = 8.8 Hz, 2H), 8.08 (d, *J* = 8.8 Hz, 2H), 7.73 (dd, *J* = 8.5, 1.9 Hz, 1H), 7.66 (d, *J* = 1.9 Hz, 1H), 7.62 (d, *J* = 1.9 Hz, 1H), 7.44 (dd, *J* = 8.5, 1.9 Hz, 1H), 5.26 (septet, *J* = 6.3 Hz, 1H), 4.85 (septet, *J* = 6.0 Hz, 1H), 4.79 (septet, *J* = 6.0 Hz, 1H), 1.48 (d, *J* = 6.0 Hz, 6H), 1.46 (d, *J* = 6.0 Hz, 6H), 1.39 (d, *J* = 6.3 Hz, 6H); ^{13}C NMR (126 MHz, CDCl$_3$) δ 165.82, 164.31, 163.07, 149.85, 146.68, 145.77, 140.28, 132.75, 131.23, 130.76, 128.19 (2C), 125.94, 124.22 (2C), 123.17, 119.28, 118.72, 118.50, 113.19, 111.90, 71.97, 71.89, 68.37, 22.24 (2C), 22.21 (2C), 21.98 (2C); m/z (ESI+) 564 [M + H]$^+$; t$_R$ = 16.49 min.

Isopropyl 4-(4-(2-hydroxy-4-nitrobenzamido)-3-isopropoxybenzamido)-3-isopropoxybenzoate 75

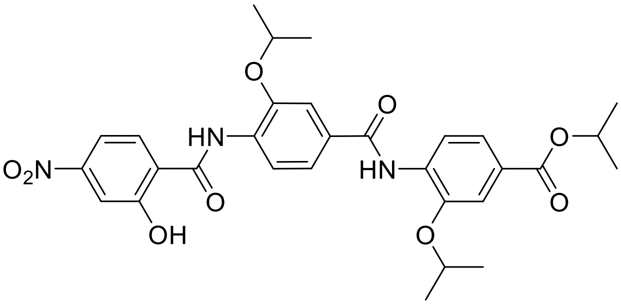

Yield 49%; yellow solid; ^1H NMR (500 MHz, DMSO-d_6) δ 12.73 (br s, 1H), 11.19 (s, 1H), 9.33 (s, 1H), 8.66 (d, *J* = 8.2 Hz, 1H), 8.29 (d, *J* = 8.5 Hz, 1H), 8.21 (d, *J* = 8.2 Hz, 1H), 7.88 (d, *J* = 1.5 Hz, 1H), 7.82 (dd, *J* = 8.5, 1.5 Hz, 1H), 7.64 (d, *J* = 1.5 Hz, 1H), 7.58 (m, 3H), 5.13 (septet, *J* = 4.7 Hz, 1H), 4.88 (septet, *J* = 5.0 Hz, 1H), 4.73 (septet, *J* = 5.4 Hz, 1H), 1.42 (d, *J* = 5.0 Hz, 6H), 1.36 (d, *J* = 5.4 Hz, 6H), 1.33 (d, *J* = 4.7 Hz, 6H); ^{13}C NMR (126 MHz, DMSO-d_6) δ 164.85, 164.17, 161.29, 156.29, 150.01, 147.66, 146.08, 132.79, 132.74, 132.15, 129.40, 126.08, 124.42, 122.01, 121.32, 120.34, 119.08, 114.11, 113.81, 112.01, 111.69, 71.58, 71.50, 68.09, 21.84 (2C), 21.68 (4C); m/z (ESI+) 580 $[M + H]^+$; t_R = 17.67 min.

Isopropyl 4-(2-hydroxy-4-(2-isopropoxy-4-nitrobenzamido)benzamido)-3-isopropoxybenzoate 76

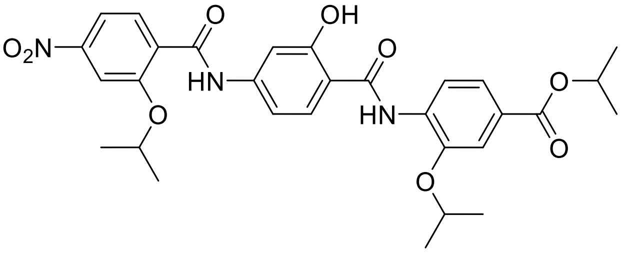

Yield 41%; yellow solid; ^1H NMR (500 MHz, DMSO-d_6) δ 11.80 (s, 1H), 11.17 (s, 1H), 10.54 (s, 1H), 8.65 (d, *J* = 8.5 Hz, 1H), 8.01 (d, *J* = 8.7 Hz, 1H), 7.90 (m, 2H), 7.83 (m, 2H), 7.56 (m, 2H), 7.16 (dd, *J* = 8.7, 1.9 Hz, 1H), 5.12 (septet, *J* = 6.2 Hz, 1H), 4.89 (septet, *J* = 6.0 Hz, 1H), 4.78 (septet, *J* = 6.0 Hz, 1H), 1.38 (d, *J* = 6.0 Hz, 6H), 1.35 (d, *J* = 6.0 Hz, 6H), 1.32 (d, *J* = 6.2 Hz, 6H); ^{13}C NMR (126 MHz, DMSO-d_6) δ 164.91, 163.79, 162.80, 156.66, 155.22, 149.47, 145.66, 143.13, 134.21, 132.33, 131.89, 130.46, 124.50, 122.61, 118.45, 115.31, 114.36, 113.45, 111.06, 108.87, 106.68, 72.36, 71.63, 67.90, 21.73 (2C), 21.70 (2C), 21.58 (2C); m/z (ESI+) 580 $[M + H]^+$; t_R = 15.71 min.

Methyl 3-chloro-2-hydroxy-4-(4-nitrobenzamido)benzoate 85

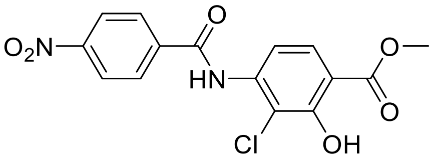

Yield 90%; yellow crystals; ^1H NMR (500 MHz, $CDCl_3$) δ 11.62 (br s, 1H), 8.39 (d, *J* = 9.1 Hz, 2H), 8.36 (d, *J* = 9.1 Hz, 2H), 7.85 (d, *J* = 8.5 Hz, 1H), 7.27 (br s, 1H), 6.53 (d, *J* = 8.5 Hz, 1H), 4.01 (s, 3H); ^{13}C NMR (126 MHz, $CDCl_3$) δ 170.08, 158.62, 150.84, 150.27, 145.28, 139.73, 130.59 (2C), 128.50, 123.70 (2C), 112.23, 110.85, 110.21, 52.73; m/z (ESI+) 351 $[M + H]^+$; t_R = 15.02 min.

Isopropyl 4-(3-chloro-2-hydroxy-4-(4-nitrobenzamido)benzamido)-3-isopropoxybenzoate 87

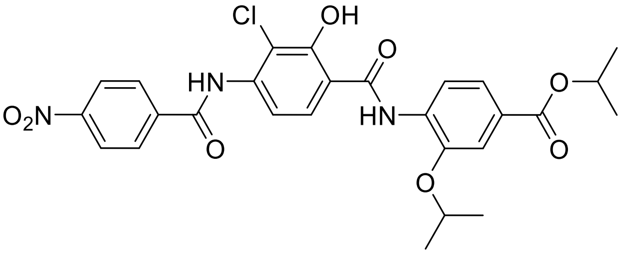

Yield 92%; beige solid; ^{1}H NMR (500 MHz, $CDCl_3$) δ 13.06 (br s, 1H), 8.98 (br s, 1H), 8.71 (br s, 1H), 8.48 (d, *J* = 8.5 Hz, 1H), 8.41 (d, *J* = 8.8 Hz, 2H), 8.28 (d, *J* = 9.1 Hz, 1H), 8.12 (d, *J* = 8.8 Hz, 2H), 7.71 (dd, *J* = 8.5, 1.9 Hz, 1H), 7.63 (d, *J* = 1.9 Hz, 1H), 7.50 (d, *J* = 9.1 Hz, 1H), 5.26 (septet, *J* = 6.3 Hz, 1H), 4.81 (septet, *J* = 6.0 Hz, 1H), 1.49 (d, *J* = 6.0 Hz, 6H), 1.39 (d, *J* = 6.3 Hz, 6H); ^{13}C NMR (126 MHz, $CDCl_3$) δ 167.06, 165.58, 163.38, 158.33, 150.20, 146.09, 139.47, 139.32, 131.22, 128.44 (2C), 126.94, 124.31, 124.29 (2C), 122.99, 119.20, 113.23, 111.90, 111.46, 110.75, 72.13, 68.58, 22.22 (2C), 21.95 (2C); m/z (ESI+) 556 $[M + H]^+$; t_R = 16.99 min.

Isopropyl 3-isopropoxy-4-(3-isopropoxy-2-methoxy-4-(4-nitrobenzamido)benzamido)benzoate 77

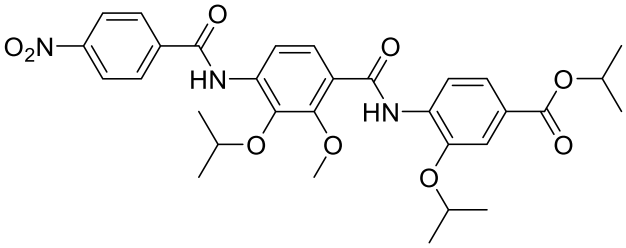

To a stirred mixture of **71** (138 mg, 0.25 mmol) and K_2CO_3 (35 mg, 0.25 mmol) in DMF (10 mL), 2-bromopropane (37 mg, 0.3 mmol) was added. The reaction mixture was stirred at 90 °C overnight. Solvent was evaporated *in vacuo*, and the residue was diluated with water (20 mL). The resulting mixture was extracted with EtOAc (3 × 20 mL). The combined organic extract was washed with brine, dried over anhydrous $MgSO_4$, and the solvent was removed by vacuum distillation. The residue was dissolved in toluene and purified using flash chromatography (SiO_2, *n*-hexane–EtOAc = 2:1).

Yield 67%; pale yellow solid; ^{1}H NMR (500 MHz, DMSO-d_6) δ 10.92 (br s, 1H), 10.18 (br s, 1H), 8.63 (d, *J* = 8.5 Hz, 1H), 8.40 (d, *J* = 8.8 Hz, 2H), 8.21 (d, *J* = 8.8 Hz, 2H), 7.88 (d, *J* = 8.8 Hz, 1H), 7.83 (d, *J* = 8.8 Hz, 1H), 7.61 (dd, *J* = 8.5, 1.6 Hz, 1H), 7.59 (d, *J* = 1.6 Hz, 1H), 5.13 (septet, *J* = 6.3 Hz, 1H), 4.86 (septet, *J* = 6.0 Hz, 1H), 4.43 (septet, *J* = 6.3 Hz, 1H), 4.06 (s, 3H), 1.40 (d, *J* = 6.0 Hz, 6H), 1.33 (d, *J* = 6.3 Hz, 6H), 1.29 (d, *J* = 6.3 Hz, 6H); ^{13}C NMR (126 MHz, DMSO-d_6) δ 164.85, 163.82, 161.78, 152.04, 149.37, 145.70, 143.11, 139.66, 136.83, 133.22, 129.16 (2C), 125.51, 125.07, 123.76 (2C), 122.66, 122.46, 120.03, 118.70, 113.06, 76.67, 71.36, 68.02, 61.89, 22.27 (2C), 21.68 (2C), 21.62 (2C); m/z (ESI+) 594 $[M + H]^+$; t_R = 17.26 min.

General procedure for synthesis of the dipeptides 89 and 90.

To a stirred solution of the 5-nitropicolinic acid **52** or **55** (226 mg, 1 mmol), and **22** (237 mg, 1 mmol) in a mixture of anhydrous $CHCl_3$ (50 mL) and DMF (1 mL) cooled at 0 °C in an ice bath, HOBt (676 mg, 5 mmol) was added under a nitrogen atmosphere followed by EDC (958 mg, 5 mmol). The reaction was stirred at 0 °C for 2 h, then at room temperature overnight. Solvent was removed by vacuum distillation. The residue was dissolved in toluene and purified using flash chromatography (SiO_2, *n*-hexane–EtOAc = 2:1).

Isopropyl 3-isopropoxy-4-(4-isopropoxy-5-nitropicolinamido)benzoate 89

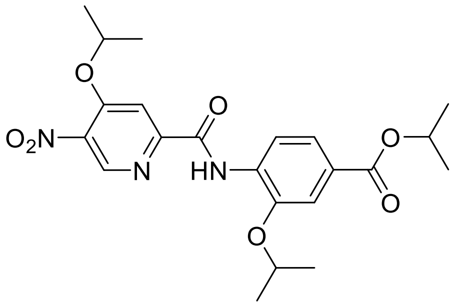

Yield 70%; pale yellow solid; ^{1}H NMR (500 MHz, $CDCl_3$) δ 10.80 (br s, 1H), 8.98 (s, 1H), 8.62 (d, *J* = 8.5 Hz, 1H), 8.01 (s, 1H), 7.73 (dd, *J* = 8.5, 1.9 Hz, 1H), 7.63 (d, *J* = 1.9 Hz, 1H), 5.26 (septet, *J* = 6.3 Hz, 1H), 4.99 (septet, *J* = 6.0 Hz, 1H), 4.75 (septet, *J* = 6.0 Hz, 1H), 1.50 (d, *J* = 6.0 Hz, 6H), 1.47 (d, *J* = 6.0 Hz, 6H), 1.39 (d, *J* = 6.3 Hz, 6H); ^{13}C NMR (126 MHz, $CDCl_3$) δ 165.72, 160.29, 158.81, 154.12, 146.66, 145.93, 138.43, 132.10, 126.68, 123.05, 118.68, 113.92, 108.61, 74.06, 72.21, 68.43, 22.11 (2C), 21.96 (2C), 21.60 (2C); m/z (ESI+) 446 $[M + H]^+$; t_R = 19.75 min.

Isopropyl 3-isopropoxy-4-(6-isopropoxy-5-nitropicolinamido)benzoate 90

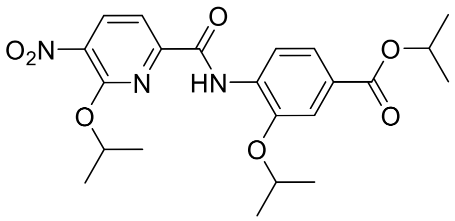

Yield 90%; yellow solid; ^{1}H NMR (500 MHz, $CDCl_3$) δ 10.30 (br s, 1H), 8.70 (d, *J* = 8.5 Hz, 1H), 8.38 (d, *J* = 8.2 Hz, 1H), 7.99 (d, *J* = 8.2 Hz, 1H), 7.73 (dd, *J* = 8.5, 1.9 Hz, 1H), 7.63 (d, *J* = 1.9 Hz, 1H), 5.71 (septet, *J* = 6.3 Hz, 1H), 5.26 (septet, *J* = 6.0 Hz, 1H), 4.84 (septet, *J* = 6.3 Hz, 1H), 1.52 (d, *J* = 6.3 Hz, 6H), 1.44 (d, *J* = 6.3 Hz, 6H), 1.39 (d, *J* = 6.0 Hz, 6H); ^{13}C NMR (126 MHz, $CDCl_3$) δ 165.72, 160.07, 154.66, 150.44, 146.20, 136.53, 136.47, 131.64, 126.65, 122.90, 118.89, 115.18, 112.97, 71.38, 71.22, 68.48, 22.09 (2C), 21.96 (2C), 21.79 (2C); m/z (ESI+) 446 $[M + H]^+$; t_R = 20.00 min.

General procedure for synthesis of the tripeptides 93 and 94.

To a stirred solution of the amino ester **91** or **92** (207 mg, 0.5 mmol), and pyridine (0.1 mL) in DCM (20 mL), 4-nitrobenzoyl chloride (185 mg, 1 mmol) was added. The reaction mixture was stirred at room temperature for 24 h then water (20 mL) and 1 N HCl (2 mL) were added. The mixture was extracted with DCM (2 × 20 mL) then EtOAc (1 × 20 mL). The combined organic extract was dried over anhydrous $MgSO_4$, and the solvent was removed by vacuum distillation. The residue was dissolved in toluene and purified using flash chromatography (SiO_2, *n*-hexane–EtOAc = 1:1).

Isopropyl 3-isopropoxy-4-(4-isopropoxy-5-(4-nitrobenzamido)picolinamido)benzoate 93

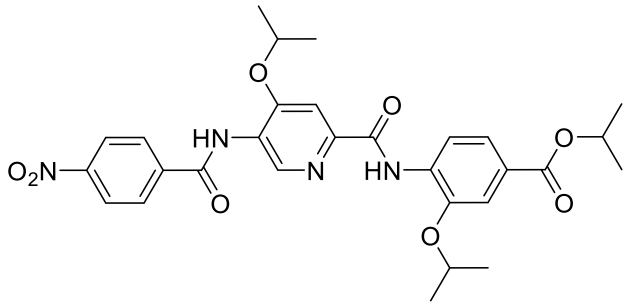

Yield 90%; pale yellow crystals; ^{1}H NMR (500 MHz, $CDCl_3$) δ 10.91 (br s, 1H), 9.72 (s, 1H), 8.65 (d, *J* = 8.5 Hz, 1H), 8.51 (br s, 1H), 8.42 (d, *J* = 9.1 Hz, 2H), 8.08 (d, *J* = 9.1 Hz, 2H), 7.89 (s, 1H), 7.73 (dd, *J* = 8.5, 1.9 Hz, 1H), 7.63 (d, *J* = 1.9 Hz, 1H), 5.25 (septet, *J* = 6.3 Hz, 1H), 4.96 (septet, *J* = 6.0 Hz, 1H), 4.74 (septet, *J* = 6.0 Hz, 1H), 1.51 (d, *J* = 6.0 Hz, 6H), 1.49 (d, *J* = 6.0 Hz, 6H), 1.39 (d, *J* = 6.3 Hz, 6H); ^{13}C NMR (126 MHz, CDCl3) δ 165.87, 162.95, 161.98, 153.17, 150.02, 147.07, 146.57, 139.65, 139.60, 132.89, 128.30 (2C), 127.08, 125.95, 124.26 (2C), 123.15, 118.39, 114.12, 105.44, 72.59, 72.29, 68.27, 22.14 (2C), 21.97 (2C), 21.94 (2C); m/z (ESI+) 565 $[M + H]^+$; t_R = 19.73 min.

Isopropyl 3-isopropoxy-4-(6-isopropoxy-5-(4-nitrobenzamido)picolinamido)benzoate 94

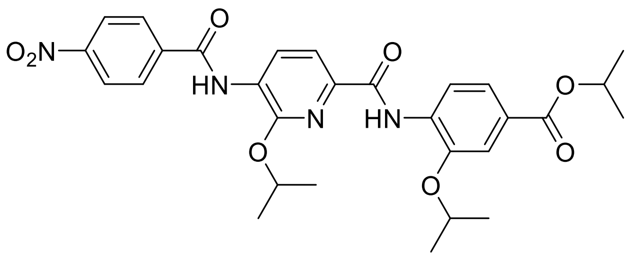

Yield 80%; yellow crystals; ^{1}H NMR (500 MHz, $CDCl_3$) δ 10.33 (br s, 1H), 8.95 (d, *J* = 8.2 Hz, 1H), 8.74 (d, *J* = 8.5 Hz, 1H), 8.59 (br s, 1H), 8.41 (d, *J* = 8.8 Hz, 2H), 8.07 (d, *J* = 8.8 Hz, 2H), 8.01 (d, *J* = 8.2 Hz, 1H), 7.72 (dd, *J* = 8.5, 1.6 Hz, 1H), 7.62 (d, *J* = 1.6 Hz, 1H), 5.69 (septet, *J* = 6.3 Hz, 1H), 5.25 (septet, *J* = 6.0 Hz, 1H), 4.83 (septet, *J* = 6.3 Hz, 1H), 1.54 (d, *J* = 6.3 Hz, 6H), 1.45 (d, *J* = 6.3 Hz, 6H), 1.39 (d, *J* = 6.0 Hz, 6H); ^{13}C NMR (126 MHz, CDCl3) δ 165.90, 163.44, 161.81, 150.71, 150.03, 146.02, 141.18, 139.62, 132.47, 128.25 (2C), 127.14, 125.85, 125.59, 124.26 (2C), 123.01, 118.66, 117.18, 113.00, 71.11, 70.03, 68.34, 22.15 (2C), 22.13 (2C), 21.97 (2C); m/z (ESI+) 565 $[M + H]^+$; t_R = 16.93 min.

General procedure for reduction of the nitro derivatives.

To a stirred solution of the nitro ester (0.4 mmol) in EtOH (20 mL), iron powder (112 mg, 2 mmol) was added at 55 °C followed by NH_4Cl (11 mg, 0.2 mmol) solution in water (2 mL). The reaction was heated at 90 °C for 1 h, then iron was filtered on hot and the filtrate was concentrated *in vacuo*. The residue was diluted with water (20 mL) and basified by $NaHCO_3$ (saturated aqueous solution) to pH 7–8. The mixture was extracted with EtOAc/THF (1:1, 3 × 20 mL). The combined organic extract was washed with brine, dried over anhydrous $MgSO_4$, and the solvent was removed by vacuum distillation. The

obtained material was dissolved in toluene and purified using flash chromatography (SiO_2, *n*-hexane–EtOAc = 2:1 or 1:1).

Isopropyl 4-(4-amino-2-hydroxy-3-isopropoxybenzamido)-3-isopropoxybenzoate 24

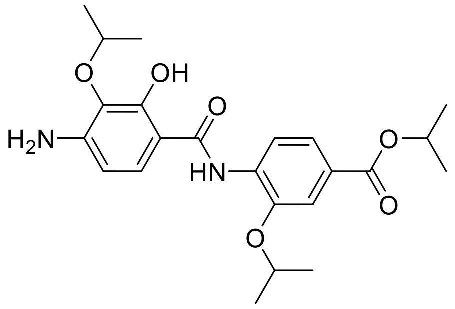

Yield 90%; colorless crystals; ^{1}H NMR (500 MHz, $CDCl_3$) δ 12.26 (br s, 1H), 8.82 (br s, 1H), 8.49 (d, *J* = 8.5 Hz, 1H), 7.69 (dd, *J* = 8.5, 1.9 Hz, 1H), 7.59 (d, *J* = 1.9 Hz, 1H), 7.08 (d, *J* = 8.8 Hz, 1H), 6.29 (d, *J* = 8.8 Hz, 1H), 5.25 (septet, *J* = 6.3 Hz, 1H), 4.76 (septet, *J* = 6.0 Hz, 1H), 4.69 (septet, *J* = 6.0 Hz, 1H), 4.29 (br s, 2H), 1.44 (d, *J* = 6.0 Hz, 6H), 1.38 (d, *J* = 6.3 Hz, 6H), 1.34 (d, *J* = 6.0 Hz, 6H); ^{13}C NMR (126 MHz, $CDCl_3$) δ 168.34, 165.83, 156.21, 146.27, 145.80, 132.40, 131.79, 125.73, 123.10, 121.30, 118.83, 113.26, 106.35, 106.11, 74.25, 71.86, 68.34, 22.70 (2C), 22.20 (2C), 21.96 (2C); m/z (ESI+) 431 $[M + H]^+$.

Isopropyl 4-(4-amino-2-hydroxy-3-methoxybenzamido)-3-isopropoxybenzoate 63

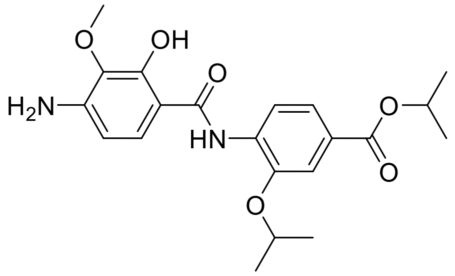

Yield 92%; beige crystals; ^{1}H NMR (500 MHz, $CDCl_3$) δ 12.31 (br s, 1H), 8.80 (br s, 1H), 8.49 (d, *J* = 8.5 Hz, 1H), 7.69 (dd, *J* = 8.5, 1.6 Hz, 1H), 7.59 (d, *J* = 1.6 Hz, 1H), 7.09 (d, *J* = 8.8 Hz, 1H), 6.30 (d, *J* = 8.8 Hz, 1H), 5.25 (septet, *J* = 6.3 Hz, 1H), 4.76 (septet, *J* = 6.0 Hz, 1H), 4.33 (br s, 2H), 3.92 (s, 3H), 1.44 (d, *J* = 6.0 Hz, 6H), 1.38 (d, *J* = 6.3 Hz, 6H); ^{13}C NMR (126 MHz, $CDCl_3$) δ 168.26, 165.80, 156.15, 145.81, 145.32, 134.04, 132.28, 125.81, 123.08, 121.64, 118.84, 113.26, 106.40, 106.14, 71.87, 68.36, 59.72, 22.20 (2C), 21.96 (2C); m/z (ESI+) 403 $[M + H]^+$; t_R = 15.56 min.

Isopropyl 4-(4-amino-3-hydroxy-2-methoxybenzamido)-3-isopropoxybenzoate 64

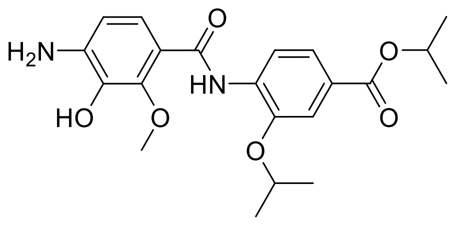

Yield 71%; orange solid; ^{1}H NMR (500 MHz, DMSO-d_6) δ 10.74 (s, 1H), 8.74 (s, 1H), 8.66 (d, *J* = 8.5 Hz, 1H), 7.56 (m, 2H), 7.40 (d, *J* = 8.6 Hz, 1H), 6.52 (d, *J* = 8.6 Hz, 1H), 5.50 (s, 2H), 5.12 (septet, *J* =

6.2 Hz, 1H), 4.83 (septet, *J* = 6.3 Hz, 1H), 3.87 (s, 3H), 1.40 (d, *J* = 5.9 Hz, 6H), 1.32 (d, *J* = 6.1 Hz, 6H); ^{13}C NMR (126 MHz, DMSO-d_6) δ 164.95, 163.07, 147.39, 145.32, 144.09, 134.86, 134.04, 124.07, 123.13, 122.41, 118.31, 112.73, 112.24, 109.60, 71.00, 67.86, 61.37, 21.70 (2C), 21.67 (2C); m/z (ESI+) 403 [M + H]$^+$; t_R = 14.99 min.

Isopropyl 4-(4-amino-2,3-diisopropoxybenzamido)-3-isopropoxybenzoate 65

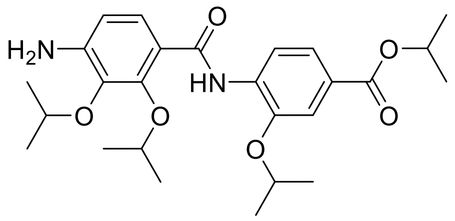

Yield 68%; yellow solid; ^{1}H NMR (500 MHz, DMSO-d_6) δ 10.37 (s, 1H), 8.59 (d, *J* = 8.5 Hz, 1H), 7.57 (dd, *J* = 8.4, 1.8 Hz, 1H), 7.55 (d, *J* = 1.8 Hz, 1H), 7.50 (d, *J* = 8.7 Hz, 1H), 6.56 (d, *J* = 8.7 Hz, 1H), 5.62 (s, 2H), 5.11 (septet, *J* = 6.3 Hz, 1H), 4.71 (septet, *J* = 6.1 Hz, 1H), 4.59 (septet, *J* = 6.2 Hz, 1H), 4.44 (septet, *J* = 6.1 Hz, 1H), 1.34 (d, *J* = 6.1 Hz, 6H), 1.32 (d, *J* = 6.3 Hz, 6H), 1.28 (d, *J* = 6.1 Hz, 6H), 1.23 (d, *J* = 6.2 Hz, 6H); ^{13}C NMR (126 MHz, DMSO-d_6) δ 164.87, 163.56, 149.71, 148.04, 145.61, 135.22, 134.00, 126.43, 124.26, 122.72, 118.45, 115.06, 114.01, 110.04, 76.24, 73.55, 72.29, 67.84, 22.11 (2C), 21.92 (2C), 21.79 (2C), 21.69 (2C); m/z (ESI+) 473 [M + H]$^+$; t_R = 16.86 min.

Isopropyl 4-(4-amino-2-hydroxybenzamido)-3-isopropoxybenzoate 66

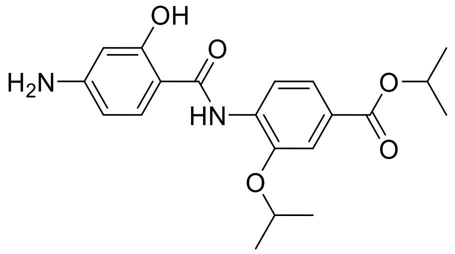

Yield 87%; beige solid; ^{1}H NMR (500 MHz, DMSO-d_6) δ 11.05 (s, 1H), 10.90 (s, 1H), 8.62 (d, *J* = 8.5 Hz, 1H), 7.69 (d, *J* = 9.2 Hz, 1H), 7.55 (dd, *J* = 8.5, 1.7 Hz, 1H), 7.52 (d, *J* = 1.7 Hz, 1H), 6.18 (m, 2H), 5.84 (s, 2H), 5.11 (septet, *J* = 6.3 Hz, 1H), 4.73 (septet, *J* = 6.0 Hz, 1H), 1.35 (d, *J* = 6.0 Hz, 6H), 1.31 (d, *J* = 6.3 Hz, 6H); ^{13}C NMR (126 MHz, DMSO-d_6) δ 164.99, 163.82, 157.64, 153.98, 145.46, 134.93, 132.41, 123.70, 122.64, 118.22, 113.46, 106.77, 106.69, 99.37, 71.52, 67.77, 21.75 (2C), 21.71 (2C); m/z (ESI+) 373 [M + H]$^+$; t_R = 12.26 min.

Isopropyl 4-(4-amino-3-isopropoxybenzamido)-3-isopropoxybenzoate 67

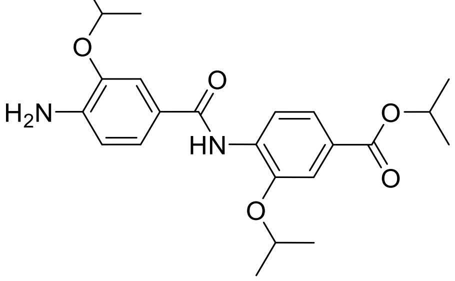

Yield 99%; beige solid; ^{1}H NMR (500 MHz, $CDCl_3$) δ 8.75 (br s, 1H), 8.62 (d, *J* = 8.5 Hz, 1H), 7.71 (dd, *J* = 8.5, 1.9 Hz, 1H), 7.59 (d, *J* = 1.9 Hz, 1H), 7.46 (d, *J* = 1.9 Hz, 1H), 7.27 (dd, *J* = 8.2, 1.9 Hz, 1H), 6.74 (d, *J* = 8.2 Hz, 1H), 5.25 (septet, *J* = 6.3 Hz, 1H), 4.76 (septet, *J* = 6.0 Hz, 1H), 4.69 (septet, *J* = 6.0 Hz, 1H), 4.22 (br s, 2H), 1.44 (d, *J* = 6.0 Hz, 6H), 1.41 (d, *J* = 6.0 Hz, 6H), 1.38 (d, *J* = 6.3 Hz, 6H); ^{13}C NMR (126 MHz, $CDCl_3$) δ 165.95, 165.06, 145.56, 144.85, 141.33, 133.40, 125.21, 124.17, 123.25, 119.83, 118.30, 113.59, 113.18, 112.36, 71.70, 70.81, 68.23, 22.22 (2C), 22.19 (2C), 21.97 (2C); m/z (ESI+) 415 $[M + H]^+$; t_R = 14.82 min.

Isopropyl 4-(4-(4-aminobenzamido)-2-hydroxy-3-isopropoxybenzamido)-3-isopropoxybenzoate 26

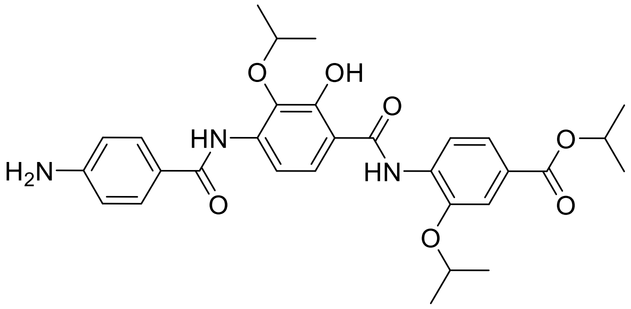

Yield 85%; white solid; ^{1}H NMR (500 MHz, $CDCl_3$) δ 12.38 (br s, 1H), 8.96 (br s, 1H), 8.81 (br s, 1H), 8.50 (d, *J* = 8.5 Hz, 1H), 8.21 (d, *J* = 9.1 Hz, 1H), 7.76 (d, *J* = 8.8 Hz, 2H), 7.71 (dd, *J* = 8.5, 1.9 Hz, 1H), 7.61 (d, *J* = 1.9 Hz, 1H), 7.27 (d, *J* = 9.1 Hz, 1H), 6.75 (d, *J* = 8.8 Hz, 2H), 5.26 (septet, *J* = 6.3 Hz, 1H), 4.88 (septet, *J* = 6.3 Hz, 1H), 4.78 (septet, *J* = 6.0 Hz, 1H), 4.10 (br s, 2H), 1.47 (d, *J* = 6.0 Hz, 6H), 1.39 (d, *J* = 6.3 Hz, 6H), 1.38 (d, *J* = 6.3 Hz, 6H); ^{13}C NMR (126 MHz, $CDCl_3$) δ 168.13, 165.72, 164.91, 154.92, 150.25, 146.01, 137.97, 134.36, 131.87, 128.97 (2C), 126.32, 123.85, 123.02, 120.58, 118.95, 114.33 (2C), 113.26, 110.84, 109.83, 75.12, 72.07, 68.43, 22.83 (2C), 22.19 (2C), 21.95 (2C); m/z (ESI+) 550 $[M + H]^+$.

Isopropyl 4-(4-(4-aminobenzamido)-2-hydroxy-3-methoxybenzamido)-3-isopropoxybenzoate 78

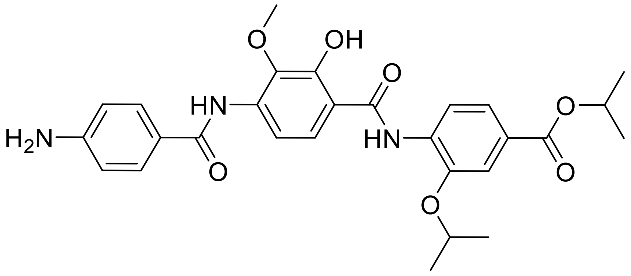

Yield 90%; pale yellow crystals; ^{1}H NMR (500 MHz, $CDCl_3$) δ 12.39 (br s, 1H), 8.95 (br s, 1H), 8.69 (br s, 1H), 8.50 (d, *J* = 8.5 Hz, 1H), 8.19 (d, *J* = 9.1 Hz, 1H), 7.75 (d, *J* = 8.8 Hz, 2H), 7.71 (dd, *J* = 8.5, 1.9 Hz, 1H), 7.61 (d, *J* = 1.9 Hz, 1H), 7.28 (d, *J* = 9.1 Hz, 1H), 6.74 (d, *J* = 8.8 Hz, 2H), 5.26 (septet, *J* = 6.3 Hz, 1H), 4.78 (septet, *J* = 6.0 Hz, 1H), 4.11 (br s, 2H), 4.05 (s, 3H), 1.47 (d, *J* = 6.0 Hz, 6H), 1.39 (d, *J* = 6.3 Hz, 6H); ^{13}C NMR (126 MHz, $CDCl_3$) δ 168.01, 165.71, 165.20, 154.86, 150.36, 146.03, 137.08, 136.57, 131.80, 129.15 (2C), 126.39, 123.77, 123.03, 120.96, 118.98, 114.28 (2C), 113.28, 111.00, 109.96, 72.10, 68.45, 60.68, 22.20 (2C), 21.96 (2C); m/z (ESI+) 522 $[M + H]^+$; t_R = 16.20 min.

Isopropyl 4-(4-(4-aminobenzamido)-3-isopropoxy-2-methoxybenzamido)-3-isopropoxybenzoate 79

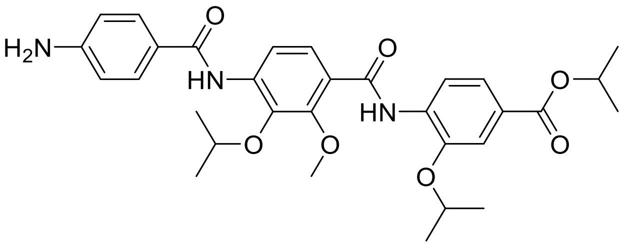

Yield 65%; pale orange solid; ^{1}H NMR (500 MHz, DMSO-d_6) δ 10.90 (br s, 1H), 9.08 (br s, 1H), 8.63 (d, *J* = 8.5 Hz, 1H), 8.06 (d, *J* = 8.8 Hz, 1H), 7.84 (d, *J* = 8.8 Hz, 1H), 7.70 (d, *J* = 8.8 Hz, 2H), 7.61 (dd, *J* = 8.5, 1.6 Hz, 1H), 7.58 (d, *J* = 1.6 Hz, 1H), 6.64 (d, *J* = 8.8 Hz, 2H), 5.91 (br s, 2H), 5.13 (septet, *J* = 6.3 Hz, 1H), 4.85 (septet, *J* = 6.0 Hz, 1H), 4.47 (septet, *J* = 6.3 Hz, 1H), 4.04 (s, 3H), 1.40 (d, *J* = 6.0 Hz, 6H), 1.33 (d, *J* = 6.3 Hz, 6H), 1.32 (d, *J* = 6.3 Hz, 6H); ^{13}C NMR (126 MHz, DMSO-d_6) δ 164.86, 164.44, 161.90, 152.73, 151.59, 145.62, 140.67, 138.06, 133.35, 129.10 (2C), 125.78, 124.92, 122.47, 120.54, 119.78, 118.61, 117.27, 113.01, 112.87 (2C), 76.48, 71.33, 68.00, 61.77, 22.34 (2C), 21.68 (2C), 21.62 (2C); m/z (ESI+) 564 $[M + H]^+$; t_R = 16.82 min.

Isopropyl 4-(4-(4-aminobenzamido)-2,3-diisopropoxybenzamido)-3-isopropoxybenzoate 80

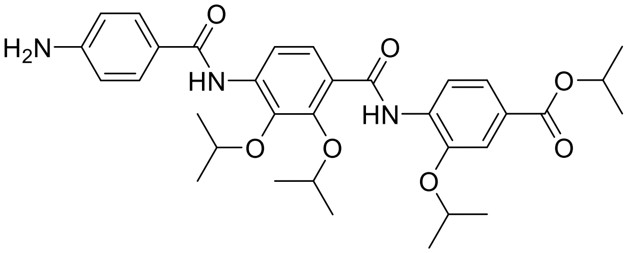

Yield 51%; white solid; ^{1}H NMR (500 MHz, DMSO-d_6) δ 10.38 (br s, 1H), 9.07 (br s, 1H), 8.62 (d, *J* = 8.2 Hz, 1H), 8.02 (d, *J* = 8.5 Hz, 1H), 7.74 (d, *J* = 8.5 Hz, 1H), 7.70 (d, *J* = 8.8 Hz, 2H), 7.61 (dd, *J* = 8.2, 1.6 Hz, 1H), 7.58 (d, *J* = 1.6 Hz, 1H), 6.63 (d, *J* = 8.8 Hz, 2H), 5.89 (br s, 2H), 5.13 (septet, *J* = 6.3 Hz, 1H), 4.76 (septet, *J* = 6.0 Hz, 1H), 4.62 (septet, *J* = 6.0 Hz, 1H), 4.52 (septet, *J* = 6.3 Hz, 1H), 1.35 (d, *J* = 6.0 Hz, 6H), 1.32 (d, *J* = 6.3 Hz, 6H), 1.31 (d, *J* = 6.3 Hz, 6H), 1.27 (d, *J* = 6.0 Hz, 6H); ^{13}C NMR (126 MHz, DMSO-d_6) δ 164.79, 164.45, 162.81, 152.68, 148.61, 145.77, 141.15, 137.73, 133.22, 129.08 (2C), 125.43, 125.03, 123.45, 122.65, 119.84, 118.65, 117.51, 113.86, 112.85 (2C), 77.15, 75.70, 72.23, 67.97, 22.24 (2C), 21.89 (2C), 21.76 (2C), 21.67 (2C); m/z (ESI+) 592 $[M + H]^+$; t_R = 17.23 min.

Isopropyl 4-(4-(4-aminobenzamido)-2-hydroxybenzamido)-3-isopropoxybenzoate 81

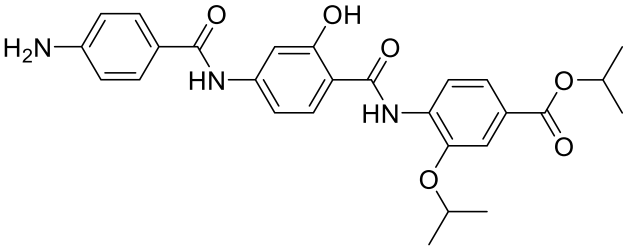

Yield 79%; beige solid; ^{1}H NMR (500 MHz, DMSO-d_6) δ 11.67 (s, 1H), 11.17 (s, 1H), 9.98 (s, 1H), 8.65 (d, *J* = 8.5 Hz, 1H), 7.95 (d, *J* = 8.7 Hz, 1H), 7.90 (d, *J* = 2.0 Hz, 1H), 7.74 (d, *J* = 8.7 Hz, 2H), 7.57 (m, 2H), 7.24 (dd, *J* = 8.8, 2.0 Hz, 1H), 6.61 (d, *J* = 8.7 Hz, 2H), 5.82 (s, 2H), 5.12 (septet, *J* = 6.2 Hz, 1H), 4.77 (septet, *J* = 6.0 Hz, 1H), 1.37 (d, *J* = 6.0 Hz, 6H), 1.32 (d, *J* = 6.2 Hz, 6H); ^{13}C NMR (126 MHz, DMSO-d_6) δ 165.68, 164.93, 163.05, 156.50, 152.46, 145.65, 144.56, 134.34, 131.35, 129.63 (2C), 124.38, 122.61, 120.67, 118.46, 113.46, 113.31, 112.51 (2C), 111.53, 106.82, 71.63, 67.88, 64.89, 21.73 (2C), 21.70 (2C); m/z (ESI+) 492 [M + H]$^+$; t_R = 12.94 min.

Isopropyl 4-(4-(4-aminobenzamido)-3-isopropoxybenzamido)-3-isopropoxybenzoate 82

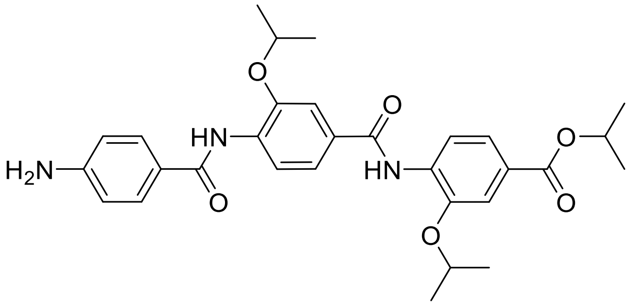

Yield 96%; white solid; ^{1}H NMR (500 MHz, $CDCl_3$) δ 8.88 (br s, 1H), 8.70 (br s, 1H), 8.69 (d, *J* = 8.5 Hz, 1H), 8.62 (d, *J* = 8.5 Hz, 1H), 7.74 (d, *J* = 8.8 Hz, 2H), 7.72 (dd, *J* = 8.5, 1.6 Hz, 1H), 7.62 (d, *J* = 1.9 Hz, 1H), 7.61 (d, *J* = 1.6 Hz, 1H), 7.41 (dd, *J* = 8.5, 1.9 Hz, 1H), 6.75 (d, *J* = 8.8 Hz, 2H), 5.25 (septet, *J* = 6.3 Hz, 1H), 4.80 (septet, *J* = 6.0 Hz, 1H), 4.77 (septet, *J* = 6.0 Hz, 1H), 4.09 (br s, 2H), 1.47 (d, *J* = 6.0 Hz, 6H), 1.45 (d, *J* = 6.0 Hz, 6H), 1.39 (d, *J* = 6.3 Hz, 6H); ^{13}C NMR (126 MHz, $CDCl_3$) δ 165.87, 164.98, 164.63, 150.16, 146.40, 145.74, 132.98, 132.65, 129.23, 128.94 (2C), 125.69, 124.17, 123.17, 118.88, 118.78, 118.42, 114.32 (2C), 113.19, 111.87, 71.88, 71.73, 68.30, 22.21 (4C), 21.97 (2C); m/z (ESI+) 534 [M + H]$^+$; t_R = 15.30 min.

Isopropyl 4-(4-(4-amino-2-hydroxybenzamido)-3-isopropoxybenzamido)-3-isopropoxybenzoate 83

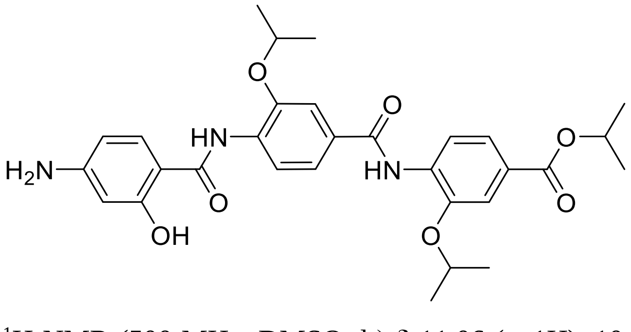

Yield 41%; beige solid; ^{1}H NMR (500 MHz, DMSO-d_6) δ 11.06 (s, 1H), 10.87 (s, 1H), 9.26 (s, 1H), 8.65 (d, *J* = 8.5 Hz, 1H), 8.23 (d, *J* = 8.5 Hz, 1H), 7.70 (d, *J* = 9.1 Hz, 1H), 7.60 (dd, *J* = 8.5, 1.9 Hz, 1H), 7.58 (d, *J* = 1.9 Hz, 1H), 7.57 (d, *J* = 1.9 Hz, 1H), 7.53 (dd, *J* = 8.5, 1.9 Hz, 1H), 6.18 (m, 2H), 5.83 (s, 2H), 5.13 (septet, *J* = 6.3 Hz, 1H), 4.81 (septet, *J* = 6.0 Hz, 1H), 4.74 (septet, *J* = 6.0 Hz, 1H), 1.39 (d, *J* = 6.0 Hz, 6H), 1.36 (d, *J* = 6.0 Hz, 6H), 1.33 (d, *J* = 6.3 Hz, 6H); ^{13}C NMR (126 MHz, DMSO-d_6) δ 164.85, 164.29, 163.83, 157.64, 153.92, 147.46, 145.67, 133.77, 132.93, 132.36, 127.68, 125.86,

122.05, 121.03, 120.33, 118.48, 113.76, 111.98, 106.78, 106.65, 99.37, 71.57, 71.28, 68.05, 21.84 (2C), 21.66 (4C); m/z (ESI+) 550 [M + H]$^+$; t_R = 16.67 min.

Isopropyl 4-(4-(4-amino-2-isopropoxybenzamido)-2-hydroxybenzamido)-3-isopropoxybenzoate 84

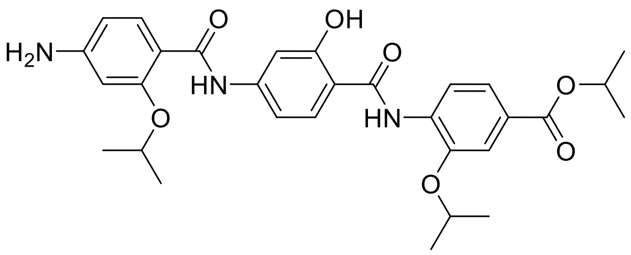

Yield 73%; pale orange solid; ^{1}H NMR (500 MHz, DMSO-d_6) δ 11.72 (s, 1H), 11.15 (s, 1H), 10.18 (s, 1H), 8.65 (d, *J* = 8.5 Hz, 1H), 7.98 (d, *J* = 8.5 Hz, 1H), 7.79 (d, *J* = 1.9 Hz, 1H), 7.71 (d, *J* = 8.5 Hz, 1H), 7.58 (dd, *J* = 8.5, 1.6 Hz, 1H), 7.55 (d, *J* = 1.6 Hz, 1H),), 7.04 (dd, *J* = 8.5, 1.9 Hz, 1H), 6.33 (d, *J* = 1.9 Hz, 1H), 6.27 (dd, *J* = 8.5, 1.9 Hz, 1H), 5.92 (br s, 2H), 5.12 (septet, *J* = 6.3 Hz, 1H), 4.78 (septet, *J* = 6.0 Hz, 1H), 4.72 (septet, *J* = 6.0 Hz, 1H), 1.46 (d, *J* = 6.0 Hz, 6H), 1.38 (d, *J* = 6.0 Hz, 6H), 1.32 (d, *J* = 6.3 Hz, 6H); ^{13}C NMR (126 MHz, DMSO-d_6) δ 164.91, 163.64, 162.89, 157.15, 156.79, 154.19, 145.62, 143.70, 134.28, 132.95, 131.92, 124.39, 122.60, 118.41, 113.45, 113.43, 110.69, 108.97, 106.85, 106.11, 97.87, 71.59, 71.49, 67.88, 21.95 (2C), 21.73 (2C), 21.69 (2C); m/z (ESI+) 550 [M + H]$^+$; t_R = 14.40 min.

Isopropyl 4-(4-(4-aminobenzamido)-3-chloro-2-hydroxybenzamido)-3-isopropoxybenzoate 88

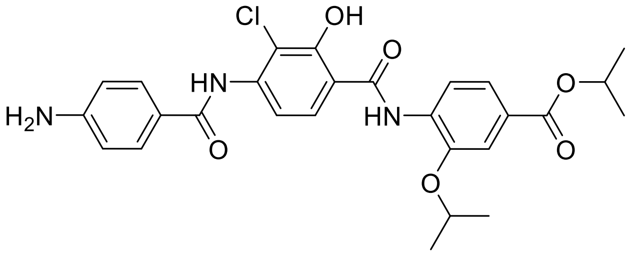

Yield 92%; white solid; ^{1}H NMR (500 MHz, $CDCl_3$) δ 12.98 (br s, 1H), 8.96 (br s, 1H), 8.62 (br s, 1H), 8.48 (d, *J* = 8.5 Hz, 1H), 8.31 (d, *J* = 8.8 Hz, 1H), 7.77 (d, *J* = 8.5 Hz, 2H), 7.70 (dd, *J* = 8.5, 1.6 Hz, 1H), 7.61 (d, *J* = 1.6 Hz, 1H), 7.45 (d, *J* = 8.8 Hz, 1H), 6.74 (d, *J* = 8.5 Hz, 2H), 5.25 (septet, *J* = 6.3 Hz, 1H), 4.78 (septet, *J* = 6.0 Hz, 1H), 4.20 (br s, 2H), 1.47 (d, *J* = 6.0 Hz, 6H), 1.38 (d, *J* = 6.3 Hz, 6H); ^{13}C NMR (126 MHz, $CDCl_3$) δ 167.33, 165.63, 165.07, 158.19, 150.76, 146.04, 140.65, 131.46, 129.22 (2C), 126.65, 124.18, 123.08, 122.97, 119.09, 114.29 (2C), 113.22, 110.74, 110.61, 110.56, 72.10, 68.48, 22.17 (2C), 21.92 (2C); m/z (ESI+) 526 [M + H]$^+$; t_R = 16.82 min.

Isopropyl 4-(5-amino-4-isopropoxypicolinamido)-3-isopropoxybenzoate 91

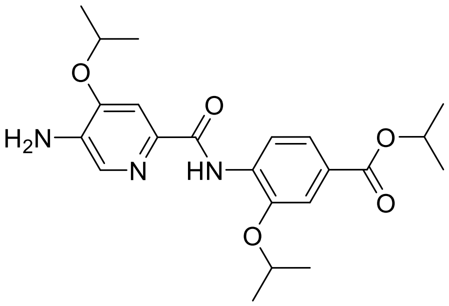

Yield 95%; white crystals; ^{1}H NMR (500 MHz, $CDCl_3$) δ 10.73 (br s, 1H), 8.65 (d, *J* = 8.5 Hz, 1H), 7.98 (s, 1H), 7.72 (dd, *J* = 8.5, 1.9 Hz, 1H), 7.71 (s, 1H), 7.61 (d, *J* = 1.9 Hz, 1H), 5.24 (septet, *J* = 6.3 Hz, 1H), 4.82 (septet, *J* = 6.0 Hz, 1H), 4.70 (septet, *J* = 6.0 Hz, 1H), 4.09 (br s, 2H), 1.46 (d, *J* = 6.0 Hz, 6H), 1.42 (d, *J* = 6.0 Hz, 6H), 1.38 (d, *J* = 6.3 Hz, 6H); ^{13}C NMR (126 MHz, $CDCl_3$) δ 166.00, 163.08, 151.07, 146.39, 141.90, 136.09, 134.22, 133.52, 125.29, 123.28, 118.24, 114.24, 105.70, 72.18, 70.84, 68.15, 22.13 (2C), 21.97 (2C), 21.92 (2C); m/z (ESI+) 416 $[M + H]^+$; t_R = 18.92 min.

Isopropyl 4-(5-amino-6-isopropoxypicolinamido)-3-isopropoxybenzoate 92

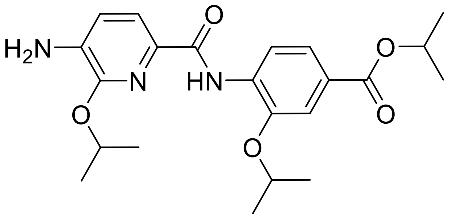

Yield 92%; beige crystals; ^{1}H NMR (500 MHz, $CDCl_3$) δ 10.31 (br s, 1H), 8.74 (d, *J* = 8.5 Hz, 1H), 7.77 (d, *J* = 7.9 Hz, 1H), 7.70 (dd, *J* = 8.5, 1.9 Hz, 1H), 7.60 (d, *J* = 1.9 Hz, 1H), 6.97 (d, *J* = 7.9 Hz, 1H), 5.59 (septet, *J* = 6.3 Hz, 1H), 5.25 (septet, *J* = 6.3 Hz, 1H), 4.80 (septet, *J* = 6.0 Hz, 1H), 4.22 (br s, 2H), 1.45 (d, *J* = 6.3 Hz, 6H), 1.44 (d, *J* = 6.0 Hz, 6H), 1.38 (d, *J* = 6.3 Hz, 6H); ^{13}C NMR (126 MHz, $CDCl_3$) δ 166.04, 163.03, 149.67, 145.96, 135.41, 134.92, 133.22, 125.18, 123.07, 119.16, 118.42, 117.67, 113.08, 71.13, 68.31, 68.17, 22.15 (2C), 22.11 (2C), 21.98 (2C); m/z (ESI+) 416 $[M + H]^+$; t_R = 15.35 min.

Isopropyl 4-(5-(4-aminobenzamido)-4-isopropoxypicolinamido)-3-isopropoxybenzoate 95

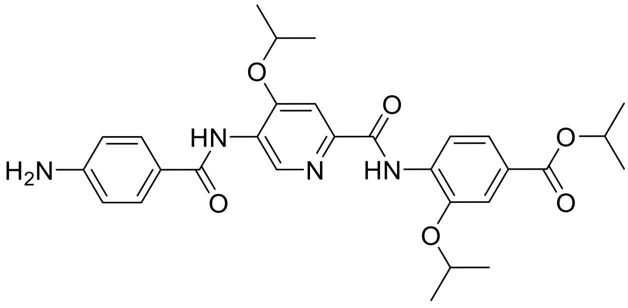

Yield 95%; white crystals; mp 196–198 °C; ^{1}H NMR (500 MHz, $CDCl_3$) δ 10.95 (br s, 1H), 9.74 (s, 1H), 8.65 (d, *J* = 8.2 Hz, 1H), 8.40 (br s, 1H), 7.84 (s, 1H), 7.74 (d, *J* = 8.8 Hz, 2H), 7.73 (dd, *J* = 8.2, 1.9 Hz, 1H), 7.62 (d, *J* = 1.9 Hz, 1H), 6.75 (d, *J* = 8.8 Hz, 2H), 5.25 (septet, *J* = 6.3 Hz, 1H), 4.92 (septet, *J* = 6.0 Hz, 1H), 4.72 (septet, *J* = 6.0 Hz, 1H), 4.13 (br s, 2H), 1.50 (d, *J* = 6.0 Hz, 6H), 1.48 (d, *J* = 6.0

Hz, 6H), 1.38 (d, J = 6.3 Hz, 6H); ^{13}C NMR (126 MHz, $CDCl_3$) δ 165.94, 164.74, 162.38, 152.72, 150.36, 146.57, 145.72, 139.25, 133.16, 129.08 (2C), 128.25, 125.71, 123.41, 123.20, 118.30, 114.30 (2C), 114.21, 105.25, 72.33, 72.09, 68.21, 22.12 (2C), 21.97 (2C), 21.94 (2C); m/z (ESI+) 535 [M + H]$^+$; t_R = 19.36 min.

Isopropyl 4-(5-(4-aminobenzamido)-6-isopropoxypicolinamido)-3-isopropoxybenzoate 96

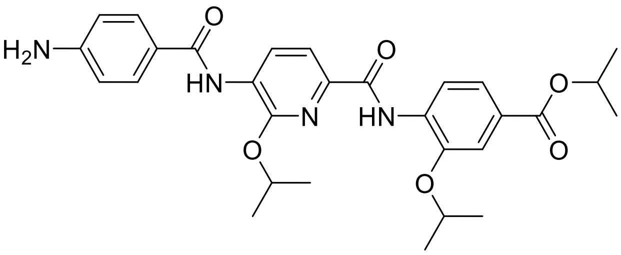

Yield 95%; beige crystals; ^{1}H NMR (500 MHz, $CDCl_3$) δ 10.34 (br s, 1H), 8.95 (d, J = 8.2 Hz, 1H), 8.75 (d, J = 8.5 Hz, 1H), 8.49 (br s, 1H), 7.97 (d, J = 8.2 Hz, 1H), 7.73 (d, J = 8.5 Hz, 2H), 7.72 (dd, J = 8.5, 1.9 Hz, 1H), 7.62 (d, J = 1.9 Hz, 1H), 6.75 (d, J = 8.5 Hz, 2H), 5.67 (septet, J = 6.3 Hz, 1H), 5.25 (septet, J = 6.3 Hz, 1H), 4.82 (septet, J = 6.3 Hz, 1H), 4.12 (br s, 2H), 1.52 (d, J = 6.3 Hz, 6H), 1.45 (d, J = 6.3 Hz, 6H), 1.39 (d, J = 6.3 Hz, 6H); ^{13}C NMR (126 MHz, $CDCl_3$) δ 165.97, 165.27, 162.22, 150.52, 150.40, 146.03, 139.77, 132.78, 129.02 (2C), 126.87, 126.29, 125.63, 123.55, 123.07, 118.64, 117.35, 114.30 (2C), 113.07, 71.15, 69.52, 68.26, 22.17 (2C), 22.13 (2C), 21.98 (2C); m/z (ESI+) 535 [M + H]$^+$; t_R = 16.12 min.

General procedure for synthesis of the carboxylic acids 2 (cystobactamid 507), **4**–**15**, and **86**.

To a stirred solution of the amino/nitro ester (0.2 mmol) in a mixture of MeOH (6 mL) and THF (2 mL), 1 N NaOH (1 mL) was added. The reaction was stirred at room temperature overnight. Solvent was concentrated *in vacuo*. The remaining residue was dissolved in water (10 mL), cooled in an ice bath and acidified by $KHSO_4$ (saturated aqueous solution) to pH 6, then extracted with EtOAc/THF (1:1, 3 × 20 mL). The combined organic extract was washed with brine, dried over anhydrous $MgSO_4$, and the solvent was removed by vacuum distillation. The obtained material was triturated with *n*-hexane/EtOAc (4:1, 25 mL), and collected by filtration.

4-(4-(4-Aminobenzamido)-2-hydroxy-3-isopropoxybenzamido)-3-isopropoxybenzoic acid 2

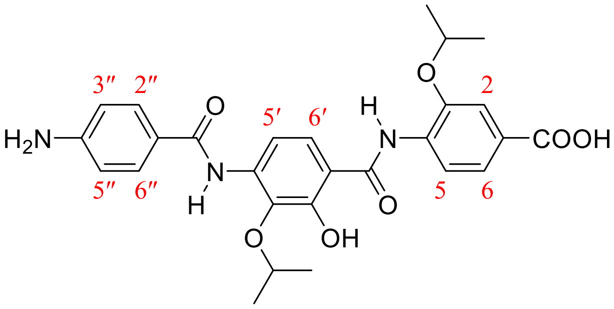

Yield 96%; beige solid; m/z (ESI+) 508 [M + H]$^+$.

Table S1. ^{1}H and ^{13}C NMR data of cystobactamid 507 (**2**) in DMSO-d_6 and acetone-d_6.[a]

Position	DMSO-d_6		Acetone-d_6	
	δ_H (multi., *J* in Hz)	δ_C	δ_H (multi., *J* in Hz)	δ_C
1	-	125.80	-	127.11
2	7.57 (d, 1.9)	113.91	7.69 (d, 1.6)	114.63
3	-	146.47	-	147.84
4	-	133.24	-	133.36
5	8.48 (d, 8.2)	119.76	8.50 (d, 8.5)	120.79
6	7.59 (dd, 8.2, 1.9)	122.61	7.72 (dd, 8.5, 1.6)	123.70
C1–<u>COOH</u>	12.77	166.97	not appeared	167.26
C3–O<u>CH</u>$(CH_3)_2$	4.75 (sept, 6.0)	71.73	4.88 (sept, 6.0)	72.89
C3–OCH<u>$(CH_3)_2$</u>	1.37 (d, 6.0)	21.69	1.47 (d, 6.0)	22.26
C4–<u>NH</u>	10.92 (br s)	-	9.64 (br s)	-
1′	-<br>-	115.51<br>115.66	-	112.39
2′	-	150.33	-	154.45
3′	-<br>-	137.16<br>137.39	-	135.22
4′	-<br>-	136.88<br>136.99	-	139.16
5′	7.64 (d, 8.8)<br>7.67 (d, 8.8)	114.20<br>113.91	8.18 (d, 8.8)	111.31
6′	7.78 (d, 8.8)	124.95	7.65 (d, 8.8)	123.38
C1′–<u>C=O</u>	-<br>-	163.86<br>163.89	-	168.26
C2′–<u>OH</u>	11.22 (br s)	-	not appeared	-
C3′–O<u>CH</u>$(CH_3)_2$	4.35 (sept, 6.0)	75.38	4.78 (sept, 6.0)	76.02
C3′–OCH<u>$(CH_3)_2$</u>	1.28 (d, 6.0)<br>1.29 (d, 6.0)	22.05<br>22.05	1.38 (d, 6.0)	22.90
C4′–<u>NH</u>	9.10 (br s)<br>9.20 (br s)	-<br>-	8.82 (br s)	-
1″	-<br>-	120.14<br>121.02	-	122.59
2″, 6″	7.70 (d, 8.8)<br>7.79 (d, 8.8)	129.12<br>129.00	7.75 (d, 8.8)	129.85
3″, 5″	6.63 (d, 8.8)<br>6.82 (d, 8.8)	112.83<br>111.72	6.79 (d, 8.8)	114.39
4″	-<br>-	150.77<br>152.59	-	153.49
C1″–<u>C=O</u>	-<br>-	164.49<br>164.53	-	165.26
C4″–<u>NH_2</u>	4.63 (t, 5.7)<br>5.87 (br s)<br>7.20 (t, 5.7)	-<br>-<br>-	5.43 (br s)	-

[a]Two rotamers of cystobactamid 507 were observed in DMSO-d_6. By changing the solvent, only single values were observed in acetone-d_6.

4-(4-(4-Aminobenzamido)-2-hydroxy-3-methoxybenzamido)-3-isopropoxybenzoic acid 4

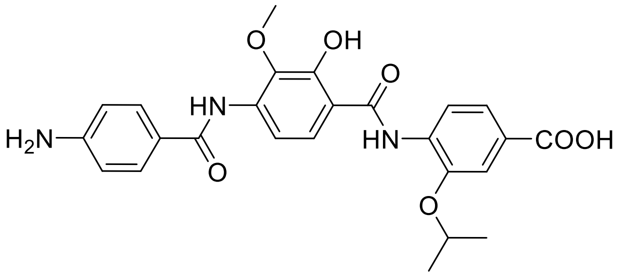

Yield 85%; pale yellow crystals; ^{1}H NMR (500 MHz, DMSO-d6) δ 12.79 (br s, 1H), 11.38 (br s, 1H), 10.98 (br s, 1H), 9.22 (br s, 1H), 8.56 (d, *J* = 8.5 Hz, 1H), 7.80 (d, *J* = 8.8 Hz, 1H), 7.73 (d, *J* = 8.5 Hz, 2H), 7.65 (d, *J* = 8.8 Hz, 1H), 7.59 (dd, *J* = 8.5, 1.6 Hz, 1H), 7.57 (d, *J* = 1.6 Hz, 1H), 6.69 (d, *J* = 8.5 Hz, 2H), 5.39 (br s, 2H), 4.76 (septet, *J* = 6.0 Hz, 1H), 3.78 (s, 3H), 1.39 (d, *J* = 6.0 Hz, 6H); ^{13}C NMR (126 MHz, DMSO-d6) δ 166.99, 165.03, 163.28, 151.46, 149.53, 146.13, 139.38, 136.34, 133.45, 129.43 (2C), 125.62, 125.55, 122.65, 121.21, 119.28, 115.71, 113.89, 113.75, 113.43 (2C), 71.72, 60.40, 21.73 (2C); m/z (ESI+) 480 $[M + H]^+$; t_R = 14.53 min.

4-(4-(4-Aminobenzamido)-3-chloro-2-hydroxybenzamido)-3-isopropoxybenzoic acid 5

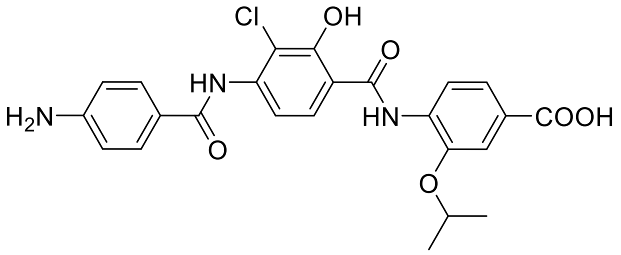

Yield 90%; pale yellow solid; ^{1}H NMR (500 MHz, DMSO-d6) δ 12.82 (br s, 1H), 11.96 (br s, 1H), 10.88 (br s, 1H), 9.44 (br s, 1H), 8.39 (d, *J* = 8.2 Hz, 1H), 8.03 (d, *J* = 8.8 Hz, 1H), 7.74 (d, *J* = 8.8 Hz, 2H), 7.60 (dd, *J* = 8.2, 1.6 Hz, 1H), 7.59 (d, *J* = 1.6 Hz, 1H), 7.58 (d, *J* = 8.8 Hz, 1H), 6.63 (d, *J* = 8.8 Hz, 2H), 5.99 (br s, 2H), 4.77 (septet, *J* = 6.3 Hz, 1H), 1.38 (d, *J* = 6.3 Hz, 6H); ^{13}C NMR (126 MHz, DMSO-d6) δ 166.95, 164.88, 163.82, 153.43, 152.73, 147.09, 140.51, 132.57, 129.56 (2C), 128.25, 126.50, 122.40, 120.72, 119.82, 116.44, 116.30, 116.12, 113.81, 112.70 (2C), 71.54, 21.71 (2C); m/z (ESI+) 484 $[M + H]^+$; t_R = 15.32 min.

4-(4-(4-Aminobenzamido)-3-isopropoxy-2-methoxybenzamido)-3-isopropoxybenzoic acid 6

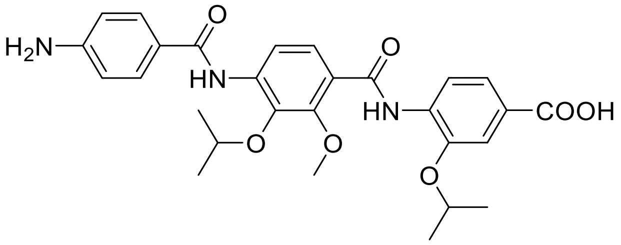

Yield 43%; beige solid; ^{1}H NMR (500 MHz, DMSO-d6) δ 12.82 (br s, 1H), 10.90 (br s, 1H), 9.09 (br s, 1H), 8.62 (d, *J* = 8.2 Hz, 1H), 8.06 (d, *J* = 8.8 Hz, 1H), 7.84 (d, *J* = 8.8 Hz, 1H), 7.70 (d, *J* = 8.5 Hz, 2H), 7.60 (dd, *J* = 8.2, 1.6 Hz, 1H), 7.58 (d, *J* = 1.6 Hz, 1H), 6.63 (d, *J* = 8.5 Hz, 2H), 5.92 (br s, 2H), 4.85 (septet, *J* = 6.0 Hz, 1H), 4.47 (septet, *J* = 6.0 Hz, 1H), 4.04 (s, 3H), 1.40 (d, *J* = 6.0 Hz, 6H), 1.32

(d, *J* = 6.0 Hz, 6H); ^{13}C NMR (126 MHz, DMSO-d_6) δ 166.96, 164.45, 161.87, 152.74, 151.59, 145.55, 140.72, 138.04, 133.03, 129.11 (2C), 125.79, 125.47, 122.67, 120.61, 119.78, 118.58, 117.31, 113.14, 112.87 (2C), 76.50, 71.14, 61.78, 22.36 (2C), 21.66 (2C); m/z (ESI+) 522 [M + H]$^+$; t_R = 15.58 min.

4-(4-(4-Aminobenzamido)-2,3-diisopropoxybenzamido)-3-isopropoxybenzoic acid 7

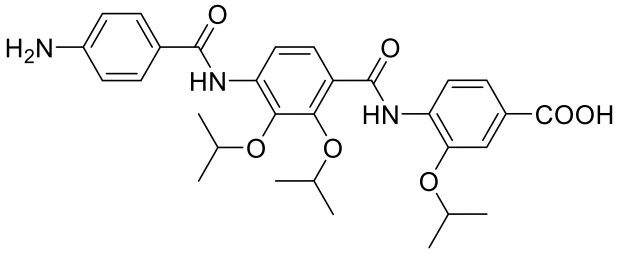

Yield 81%; beige solid; ^{1}H NMR (500 MHz, DMSO-d_6) δ 12.82 (br s, 1H), 10.36 (br s, 1H), 9.06 (br s, 1H), 8.60 (d, *J* = 8.5 Hz, 1H), 8.01 (d, *J* = 8.8 Hz, 1H), 7.75 (d, *J* = 8.8 Hz, 1H), 7.70 (d, *J* = 8.8 Hz, 2H), 7.61 (dd, *J* = 8.5, 1.9 Hz, 1H), 7.58 (d, *J* = 1.9 Hz, 1H), 6.63 (d, *J* = 8.8 Hz, 2H), 5.90 (br s, 2H), 4.75 (septet, *J* = 6.0 Hz, 1H), 4.63 (septet, *J* = 6.3 Hz, 1H), 4.52 (septet, *J* = 6.0 Hz, 1H), 1.35 (d, *J* = 6.0 Hz, 6H), 1.31 (d, *J* = 6.0 Hz, 6H), 1.27 (d, *J* = 6.3 Hz, 6H); ^{13}C NMR (126 MHz, DMSO-d_6) δ 166.88, 164.45, 162.79, 152.66, 148.60, 145.71, 141.15, 137.69, 132.89, 129.08 (2C), 125.58, 125.44, 123.52, 122.83, 119.87, 118.64, 117.50, 113.94, 112.87 (2C), 77.12, 75.70, 72.02, 22.25 (2C), 21.90 (2C), 21.79 (2C); m/z (ESI+) 550 [M + H]$^+$; t_R = 13.10 min.

4-(5-(4-Aminobenzamido)-4-isopropoxypicolinamido)-3-isopropoxybenzoic acid 8

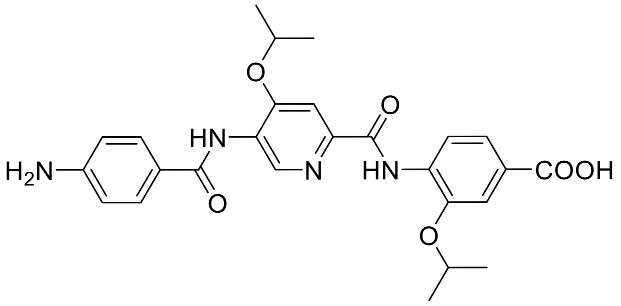

Yield 93%; beige solid; mp 299–301 °C; ^{1}H NMR (500 MHz, DMSO-d_6) δ 12.87 (br s, 1H), 10.79 (br s, 1H), 9.15 (s, 1H), 9.07 (br s, 1H), 8.56 (d, *J* = 8.5 Hz, 1H), 7.80 (s, 1H), 7.70 (d, *J* = 8.5 Hz, 2H), 7.64 (dd, *J* = 8.5, 1.6 Hz, 1H), 7.60 (d, *J* = 1.6 Hz, 1H), 6.63 (d, *J* = 8.5 Hz, 2H), 5.92 (br s, 2H), 4.99 (septet, *J* = 6.0 Hz, 1H), 4.77 (septet, *J* = 6.0 Hz, 1H), 1.40 (d, *J* = 6.0 Hz, 6H), 1.39 (d, *J* = 6.0 Hz, 6H); ^{13}C NMR (126 MHz, DMSO-d_6) δ 166.92, 164.88, 161.48, 155.39, 152.80, 146.01, 145.67, 142.30, 132.39, 129.52 (2C), 128.30, 125.85, 123.02, 119.68, 117.75, 114.22, 112.74 (2C), 106.03, 72.03, 71.69, 21.86 (2C), 21.42 (2C); m/z (ESI+) 493 [M + H]$^+$; t_R = 18.34 min.

4-(5-(4-Aminobenzamido)-6-isopropoxypicolinamido)-3-isopropoxybenzoic acid 9

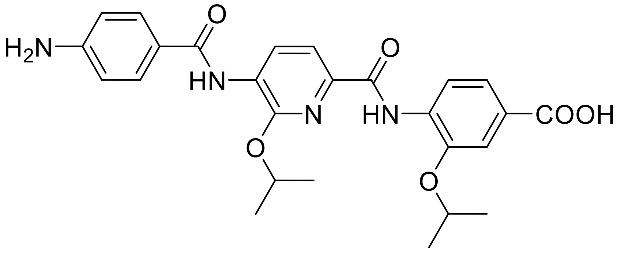

Yield 90%; off-white crystals; ^{1}H NMR (500 MHz, DMSO-d$_6$) δ 13.61 (br s, 1H), 10.31 (br s, 1H), 8.99 (br s, 1H), 8.62 (d, *J* = 7.9 Hz, 1H), 8.60 (d, *J* = 8.2 Hz, 1H), 7.82 (d, *J* = 7.9 Hz, 1H), 7.67 (d, *J* = 8.5 Hz, 2H), 7.62 (dd, *J* = 8.2, 1.6 Hz, 1H), 7.61 (d, *J* = 1.6 Hz, 1H), 6.64 (d, *J* = 8.5 Hz, 2H), 5.93 (br s, 2H), 5.54 (septet, *J* = 6.3 Hz, 1H), 4.86 (septet, *J* = 6.0 Hz, 1H), 1.47 (d, *J* = 6.3 Hz, 6H), 1.38 (d, *J* = 6.0 Hz, 6H); ^{13}C NMR (126 MHz, DMSO-d$_6$) δ 167.12, 165.07, 161.33, 152.91, 151.88, 145.61, 139.36, 131.67, 129.34 (2C), 128.38, 127.07, 126.58, 122.54, 119.69, 117.80, 116.33, 113.16, 112.82 (2C), 71.07, 69.26, 21.70 (2C), 21.64 (2C); m/z (ESI+) 493 [M + H]$^+$; t$_R$ = 12.75 min.

4-(4-(4-Aminobenzamido)-2-hydroxybenzamido)-3-isopropoxybenzoic acid 10

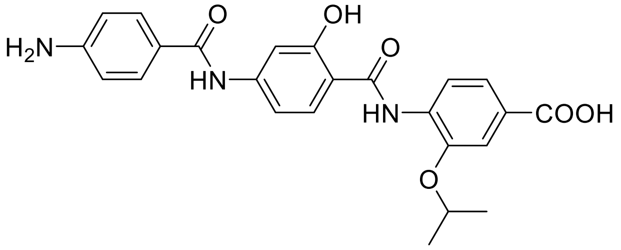

Yield 73%; beige solid; ^{1}H NMR (500 MHz, DMSO-d$_6$) δ 12.75 (br s, 1H), 11.67 (s, 1H), 11.15 (s, 1H), 10.00 (s, 1H), 8.63 (d, *J* = 8.5 Hz, 1H), 7.96 (d, *J* = 8.5 Hz, 1H), 7.90 (d, *J* = 1.6 Hz, 1H), 7.75 (d, *J* = 8.5 Hz, 2H), 7.58 (dd, *J* = 8.5, 1.6 Hz, 1H), 7.56 (d, *J* = 1.6 Hz, 1H), 7.25 (dd, *J* = 8.5, 1.6 Hz, 1H), 6.65 (d, *J* = 8.5 Hz, 2H), 5.85 (br s, 2H), 4.77 (septet, *J* = 6.0 Hz, 1H), 1.37 (d, *J* = 6.0 Hz, 6H); ^{13}C NMR (126 MHz, DMSO-d$_6$) δ 167.03, 165.66, 163.04, 156.50, 151.79, 145.58, 144.49, 133.99, 131.36, 129.63 (2C), 124.93, 122.79, 121.18, 118.44, 113.55, 113.40, 112.91 (2C), 111.54, 106.86, 71.43, 21.77 (2C); m/z (ESI+) 450 [M + H]$^+$; t$_R$ = 9.33 min.

4-(4-(4-Aminobenzamido)-3-isopropoxybenzamido)-3-isopropoxybenzoic acid 11

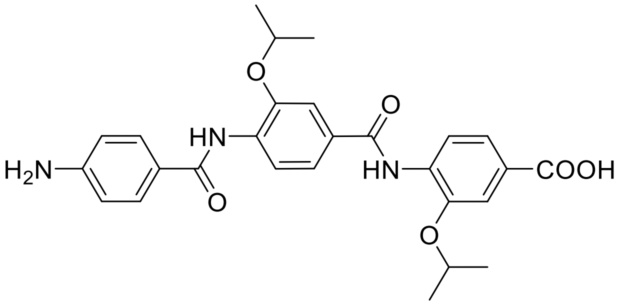

Yield 94%; white solid; ^{1}H NMR (500 MHz, DMSO-d$_6$) δ 12.69 (br s, 1H), 9.29 (br s, 1H), 8.89 (br s, 1H), 8.32 (d, *J* = 8.2 Hz, 1H), 8.19 (d, *J* = 8.2 Hz, 1H), 7.65 (d, *J* = 8.5 Hz, 2H), 7.60 (dd, *J* = 8.2, 1.6 Hz, 1H), 7.60 (d, *J* = 1.6 Hz, 1H), 7.58 (d, *J* = 1.6 Hz, 1H), 7.55 (dd, *J* = 8.2, 1.6 Hz, 1H), 6.64 (d, *J* =

8.5 Hz, 2H), 5.89 (br s, 2H), 4.80 (septet, *J* = 6.0 Hz, 1H), 4.72 (septet, *J* = 6.0 Hz, 1H), 1.40 (d, *J* = 6.0 Hz, 6H), 1.36 (d, *J* = 6.0 Hz, 6H); ^{13}C NMR (126 MHz, DMSO-d_6) δ 167.02, 164.48, 164.31, 152.67, 147.58, 146.90, 132.48, 132.34, 129.11, 128.96 (2C), 126.75, 122.23, 121.24, 120.23, 120.19, 120.04, 113.92, 112.95 (2C), 112.14, 71.44, 71.41, 21.81 (2C), 21.73 (2C); m/z (ESI+) 492 [M + H]$^+$; t_R = 12.06 min.

4-(4-(4-Amino-2-hydroxybenzamido)-3-isopropoxybenzamido)-3-isopropoxybenzoic acid 12

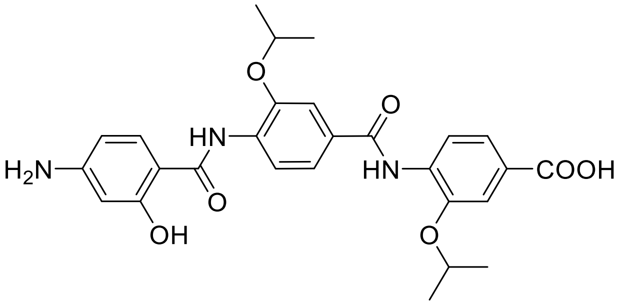

Yield 70%; beige solid; ^{1}H NMR (500 MHz, DMSO-d_6) δ 11.20 (s, 1H), 10.91 (s, 1H), 9.26 (s, 1H), 8.65 (d, *J* = 8.5 Hz, 1H), 8.19 (d, *J* = 8.2 Hz, 1H), 7.71 (d, *J* = 8.5 Hz, 1H), 7.59 (dd, *J* = 8.2, 1.6 Hz, 1H), 7.58 (d, *J* = 1.9 Hz, 1H), 7.56 (d, *J* = 1.6 Hz, 1H), 7.53 (dd, *J* = 8.5, 1.9 Hz, 1H), 6.24 (d, *J* = 1.6 Hz, 1H), 6.20 (dd, *J* = 8.5, 1.6 Hz, 1H), 5.97 (br s, 3H), 4.81 (septet, *J* = 6.0 Hz, 1H), 4.73 (septet, *J* = 6.0 Hz, 1H), 1.39 (d, *J* = 6.0 Hz, 6H), 1.36 (d, *J* = 6.0 Hz, 6H); ^{13}C NMR (126 MHz, DMSO-d_6) δ 167.00, 164.34, 163.80, 157.68, 153.29, 147.50, 145.69, 133.77, 132.59, 132.43, 127.81, 126.50, 122.25, 121.13, 120.41, 118.49, 113.86, 111.98, 107.18, 106.99, 99.89, 71.37, 71.33, 21.89 (2C), 21.73 (2C); m/z (ESI+) 508 [M + H]$^+$; t_R = 15.23 min.

4-(4-(4-Amino-2-isopropoxybenzamido)-2-hydroxybenzamido)-3-isopropoxybenzoic acid 13

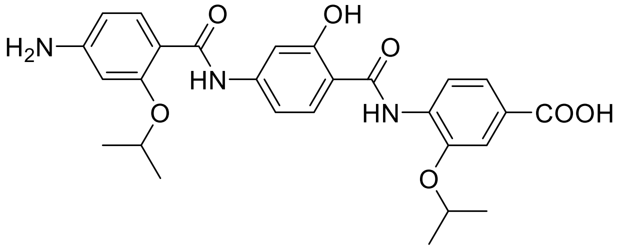

Yield 69%; beige solid; ^{1}H NMR (500 MHz, DMSO-d_6) 12.77 (br s, 1H), 11.72 (s, 1H), 11.13 (s, 1H), 10.18 (s, 1H), 8.63 (d, *J* = 8.5 Hz, 1H), 7.98 (d, *J* = 8.8 Hz, 1H), 7.77 (d, *J* = 1.6 Hz, 1H), 7.71 (d, *J* = 8.5 Hz, 1H), 7.58 (dd, *J* = 8.5, 1.6 Hz, 1H), 7.55 (d, *J* = 1.6 Hz, 1H), 7.05 (dd, *J* = 8.8, 1.6 Hz, 1H), 6.33 (d, *J* = 1.6 Hz, 1H), 6.27 (dd, *J* = 8.5, 1.6 Hz, 1H), 5.91 (br s, 2H), 4.77 (septet, *J* = 6.0 Hz, 1H), 4.72 (septet, *J* = 6.0 Hz, 1H), 1.46 (d, *J* = 6.0 Hz, 6H), 1.37 (d, *J* = 6.0 Hz, 6H); ^{13}C NMR (126 MHz, DMSO-d_6) δ 167.05, 163.67, 162.92, 157.19, 156.82, 154.22, 145.59, 143.70, 133.96, 132.99, 131.96, 124.98, 122.81, 118.45, 113.55, 113.54, 110.72, 109.00, 106.89, 106.15, 97.92, 71.54, 71.43, 21.97 (2C), 21.79 (2C); m/z (ESI+) 508 [M + H]$^+$; t_R = 11.35 min.

4-(4-(2-Hydroxy-4-nitrobenzamido)-3-isopropoxybenzamido)-3-isopropoxybenzoic acid 14

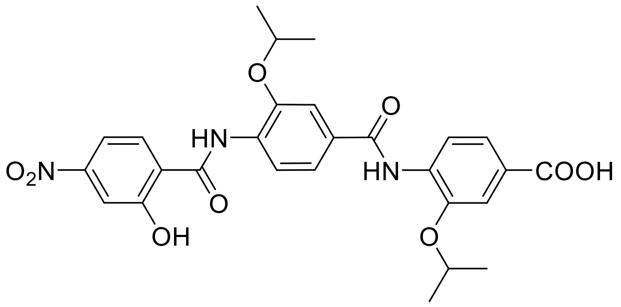

Yield 76%; yellow solid; ^{1}H NMR (500 MHz, DMSO-d_6) δ 12.36 (br s, 2H), 11.38 (br s, 1H), 9.29 (s, 1H), 8.67 (d, *J* = 8.5 Hz, 1H), 8.28 (d, *J* = 8.8 Hz, 1H), 8.19 (d, *J* = 8.5 Hz, 1H), 7.86 (d, *J* = 1.9 Hz, 1H), 7.77 (dd, *J* = 8.8, 1.9 Hz, 1H), 7.63 (d, *J* = 1.6 Hz, 1H), 7.60 (dd, *J* = 8.5, 1.6 Hz, 1H), 7.58 (dd, *J* = 8.5, 1.6 Hz, 1H), 7.57 (d, *J* = 1.6 Hz, 1H), 4.87 (septet, *J* = 6.0 Hz, 1H), 4.73 (septet, *J* = 6.0 Hz, 1H), 1.41 (d, *J* = 6.0 Hz, 6H), 1.36 (d, *J* = 6.0 Hz, 6H); ^{13}C NMR (126 MHz, DMSO-d_6) δ 167.02, 164.16, 161.51, 157.13, 150.03, 147.56, 146.11, 132.69, 132.42, 132.25, 129.38, 126.75, 124.44, 122.19, 121.21, 120.30, 119.10, 113.91, 113.51, 111.99, 111.85, 71.47, 71.39, 21.80 (2C), 21.69 (2C); m/z (ESI+) 538 [M + H]$^+$; t_R = 14.46 min.

4-(2-Hydroxy-4-(2-isopropoxy-4-nitrobenzamido)benzamido)-3-isopropoxybenzoic acid 15

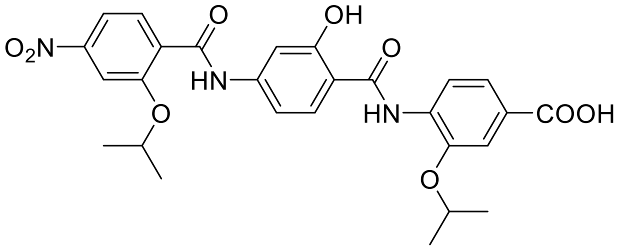

Yield 75%; pale yellow solid; ^{1}H NMR (500 MHz, DMSO-d_6) δ 12.75 (br s, 1H), 11.79 (s, 1H), 11.15 (s, 1H), 10.54 (s, 1H), 8.63 (d, *J* = 8.4 Hz, 1H), 8.01 (d, *J* = 8.7 Hz, 1H), 7.90 (m, 2H), 7.81 (m, 2H), 7.56 (m, 2H), 7.16 (dd, *J* = 8.7, 1.8 Hz, 1H), 4.89 (septet, *J* = 6.0 Hz, 1H), 4.78 (septet, *J* = 6.0 Hz, 1H), 1.38 (d, *J* = 6.0 Hz, 6H), 1.35 (d, *J* = 6.0 Hz, 6H); ^{13}C NMR (126 MHz, DMSO-d_6) δ 167.00, 163.80, 162.78, 156.65, 155.22, 149.47, 145.59, 143.09, 133.86, 132.35, 131.89, 130.46, 125.06, 122.78, 118.45, 115.32, 114.41, 113.54, 111.05, 108.88, 106.69, 72.37, 71.42, 21.77 (2C), 21.59 (2C); m/z (ESI+) 538 [M + H]$^+$; t_R = 16.01 min.

3-Chloro-2-hydroxy-4-(4-nitrobenzamido)benzoic acid 86

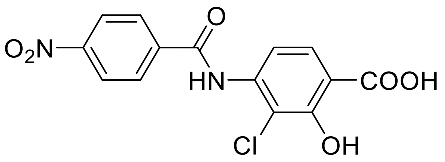

Yield 75%; pale yellow solid; m/z (ESI+) 337 [M + H]$^+$; t_R = 17.50 min.

***tert*-Butyl 3-isopropoxy-4-nitrobenzoate 28**

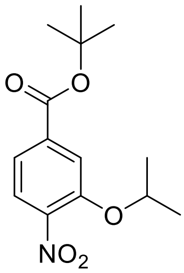

To a stirred ice-cooled solution of **27** (9.0 g, 40 mmol) in DCM (160 mL), TEA (12.14 g, 120 mmol) and 4-dimethylaminopyridine (2.44 g, 20 mmol), di-*tert*-butyl dicarbonate (13.1 g, 60 mmol) was added cautiously. The reaction mixture was stirred at room temperature overnight. Solvent was removed under reduced pressure, and residue was dissolved in EtOAc (150 mL) and was washed with 1 N HCl (2 × 50 mL) then 1 N $NaHCO_3$ (50 mL). Organic layer was dried over anhydrous $MgSO_4$ and solvent was removed under vacuum. The residue was purified using flash chromatography (SiO_2, *n*-hexane–EtOAc = 8:1).

Yield 95%; yellow oil; ^{1}H NMR (500 MHz, $CDCl_3$) δ 7.73 (d, *J* = 8.2 Hz, 1H), 7.70 (d, *J* = 1.6 Hz, 1H), 7.57 (dd, *J* = 8.2, 1.6 Hz, 1H), 4.75 (septet, *J* = 6.3 Hz, 1H), 1.61 (s, 9H), 1.41 (d, *J* = 6.3 Hz, 6H); ^{13}C NMR (126 MHz, $CDCl_3$) δ 163.91, 150.74, 143.38, 136.39, 124.89, 120.89, 116.80, 82.41, 72.92, 28.04 (3C), 21.78 (2C); m/z (ESI+) 281 $[M]^+$; t_R = 9.51 min.

***tert*-Butyl 4-amino-3-isopropoxybenzoate 29**

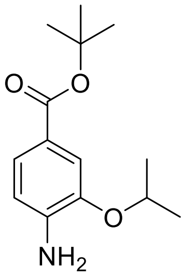

To a stirred solution of **28** (562 mg, 2 mmol) in EtOH (20 mL), iron powder (560 mg, 10 mmol) was added at 55 °C followed by NH_4Cl (54 mg, 1 mmol) solution in water (5 mL). The reaction mixture was stirred at 90 °C for 1 h, then iron was filtered on hot and the filtrate was concentrated *in vacuo*. The residue was diluted with water (20 mL) and basified by $NaHCO_3$ (saturated aqueous solution) to pH 8–9. The mixture was extracted with EtOAc (3 × 30 mL). The combined organic extract was washed with brine, dried over anhydrous $MgSO_4$, and the solvent was removed by vacuum distillation. The residue was purified using flash chromatography (SiO_2, *n*-hexane–EtOAc = 5:1).

Yield 92%; colorless oil; ^{1}H NMR (500 MHz, $CDCl_3$) δ 7.47 (dd, *J* = 8.2, 1.9 Hz, 1H), 7.44 (d, *J* = 1.9 Hz, 1H), 6.65 (d, *J* = 8.2 Hz, 1H), 4.62 (septet, *J* = 6.0 Hz, 1H), 4.09 (br s, 2H), 1.58 (s, 9H), 1.37 (d, *J* = 6.0 Hz, 6H); ^{13}C NMR (126 MHz, $CDCl_3$) δ 166.17, 144.17, 141.64, 123.53, 121.48, 113.97, 113.29, 80.03, 70.79, 28.28 (3C), 22.19 (2C); m/z (ESI+) 251 $[M]^+$; t_R = 8.46 min.

***tert*-Butyl 4-(2-hydroxy-3-methoxy-4-nitrobenzamido)-3-isopropoxybenzoate 31**

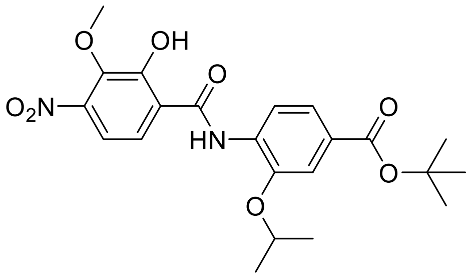

To a stirred solution of the *N*-protected carboxylic acid **30** (213 mg, 1 mmol) and the *C*-protected amine **29** (301 mg, 1.2 mmol) in anhydrous $CHCl_3$ (50 mL) under a nitrogen atmosphere, dichlorotriphenylphosphorane (1.5 g, 4.5 mmol) was added. The reaction mixture was heated at 80 °C overnight. Solvent was removed by vacuum distillation. The residue was dissolved in toluene and purified using flash chromatography (SiO_2, *n*-hexane–EtOAc = 3:1).

Yield 90%; yellow crystals; ^{1}H NMR (500 MHz, $CDCl_3$) δ 12.15 (br s, 1H), 9.11 (br s, 1H), 8.47 (d, *J* = 8.5 Hz, 1H), 7.66 (dd, *J* = 8.5, 1.9 Hz, 1H), 7.59 (d, *J* = 1.9 Hz, 1H), 7.37 (d, *J* = 8.8 Hz, 1H), 7.30 (d, *J* = 8.8 Hz, 1H), 4.78 (septet, *J* = 6.3 Hz, 1H), 4.08 (s, 3H), 1.61 (s, 9H), 1.46 (d, *J* = 6.3 Hz, 6H); ^{13}C NMR (126 MHz, $CDCl_3$) δ 166.09, 165.19, 156.65, 146.64, 146.15, 142.90, 130.67, 128.41, 122.80, 120.21, 119.31, 119.19, 113.59, 113.07, 81.28, 72.03, 61.98, 28.16 (3C), 22.15 (2C).

***tert*-Butyl 4-(4-amino-2-hydroxy-3-methoxybenzamido)-3-isopropoxybenzoate 32**

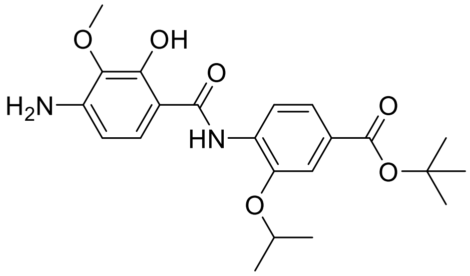

Compound **32** was synthesized according to the general procedure for reduction of the nitro derivatives starting with **31**. The crude material was purified using flash chromatography (SiO_2, *n*-hexane–EtOAc = 2:1).

Yield 92%; colorless oil; ^{1}H NMR (500 MHz, $CDCl_3$) δ 12.32 (br s, 1H), 8.78 (br s, 1H), 8.46 (d, *J* = 8.5 Hz, 1H), 7.63 (dd, *J* = 8.5, 1.9 Hz, 1H), 7.56 (d, *J* = 1.9 Hz, 1H), 7.09 (d, *J* = 8.8 Hz, 1H), 6.30 (d, *J* = 8.8 Hz, 1H), 4.73 (septet, *J* = 6.0 Hz, 1H), 4.33 (br s, 2H), 3.92 (s, 3H), 1.60 (s, 9H), 1.44 (d, *J* = 6.0 Hz, 6H); ^{13}C NMR (126 MHz, $CDCl_3$) δ 168.23, 165.50, 156.13, 145.80, 145.29, 134.04, 132.00, 126.96, 122.96, 121.62, 118.77, 113.15, 106.40, 106.12, 80.94, 71.86, 59.70, 28.19 (3C), 22.20 (2C).

***tert*-Butyl 4-(2-hydroxy-3-methoxy-4-(4-nitrobenzamido)benzamido)-3-isopropoxybenzoate 33**

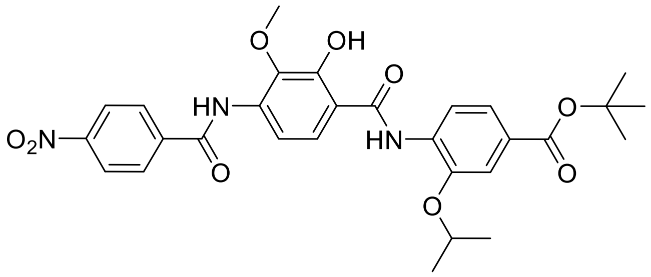

Compound **33** was synthesized according to the general procedure for amide coupling using dichlorotriphenylphosphorane starting with *p*-nitrobenzoic acid (**68**) and **32**. The crude material was purified using flash chromatography (SiO_2, DCM–MeOH = 99:1).

Yield 80%; yellow solid.

***tert*-Butyl 4-(4-(4-aminobenzamido)-2-hydroxy-3-methoxybenzamido)-3-isopropoxybenzoate 34**

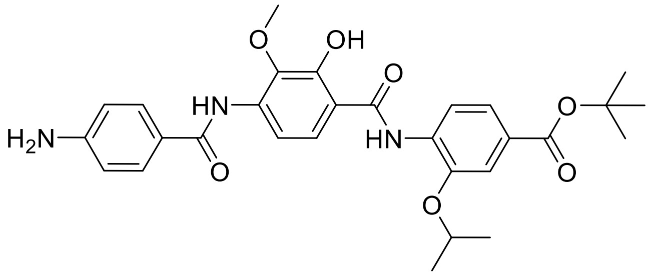

Compound **34** was synthesized according to the general procedure for reduction of the nitro derivatives starting with **33**. The crude material was purified using flash chromatography (SiO_2, DCM–MeOH = 98:2).

Yield 90%; beige oil; m/z (ESI+) 536 $[M + H]^+$.

***N*^{4}-Trityl-L-asparagine 35**

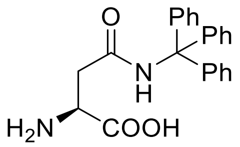

To a stirred suspension of L-asparagine (2.64 g, 20 mmol) and triphenylcarbinol (10.41 g, 40 mmol) in glacial acetic acid (60 mL) and acetic anhydride (4.08 g, 40 mmol), concd H_2SO_4 (2.25 g, 23 mmol) was added. The reaction mixture was stirred at 60 °C for 3 h, then it was allowed to cool to room temperature. The solution was poured slowly on an ice-cooled water (120 mL) with stirring, pH was adjusted to 6 by 2 M NaOH, and the mixture was further stirred in an ice bath for 1 h. The precipitate was filtered, was washed with cold water (2 × 50 mL), toluene–diethyl ether (1:1) mixture (2 × 50 mL), then petroleum ether 40–60 °C (50 mL) and was dried.

Yield 77%; white solid; ^{1}H NMR (500 MHz, DMSO-d_6) δ 9.93 (br s, 1H), 7.67 (br s, 2H), 7.26 (m, 6H), 7.19 (m, 9H), 3.45 (t, *J* = 6.3 Hz, 1H), 2.92 (dd, *J* = 16.1, 6.6 Hz, 1H), 2.42 (dd, *J* = 16.1, 6.6 Hz, 1H); ^{13}C NMR (126 MHz, DMSO-d_6) δ 169.71 (2C), 144.92 (3C), 128.60 (6C), 127.51 (6C), 126.36 (3C), 69.42, 50.14, 37.97; m/z (ESI+) 375 $[M + H]^+$; t_R = 3.14 min.

N^2-(4-Nitrobenzoyl)-N^4-trityl-L-asparagine 36

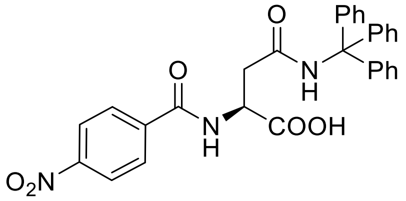

To a stirred ice-cooled solution of **35** (3.74 g, 10 mmol) in a mixture of 1 N NaOH (10 mL) and dioxane (5 mL), 1 N NaOH (10 mL) and a solution of *p*-nitrobenzoyl chloride (1.85 g, 10 mmol) in dioxane (10 mL) were added dropwise and concurrently. The reaction mixture was stirred at 0–5 °C for 1 h, and then at room temperature for further 1 h. The reaction was diluted with water (20 mL) and was acidified with HCl to pH 4–5. The mixture was extracted with EtOAc (3 × 30 mL). The organic layer was dried over anhydrous $MgSO_4$ and the solvent was removed by vacuum distillation. The crude material was triturated with diethyl ether (30 mL), was filtered, and was washed with diethyl ether (2 × 30 mL).

Yield 65%; beige solid; ^{1}H NMR (500 MHz, DMSO-d_6) δ 12.84 (br s, 1H), 9.12 (d, *J* = 7.9 Hz, 1H), 8.71 (br s, 1H), 8.38 (d, *J* = 8.8 Hz, 2H), 8.11 (d, *J* = 8.8 Hz, 2H), 7.17 (m, 15H), 4.74 (m, 1H), 2.89 (dd, *J* = 14.8, 9.8 Hz, 1H), 2.81 (dd, *J* = 15.1, 4.7 Hz, 1H); ^{13}C NMR (126 MHz, DMSO-d_6) δ 172.82, 168.65, 164.47, 149.25, 144.72 (3C), 139.40, 128.87 (2C), 128.55 (6C), 127.48 (6C), 126.41 (3C), 123.69 (2C), 69.39, 50.18, 37.64; m/z (ESI+) 524 $[M + H]^+$; t_R = 8.17 min.

N^2-(4-Aminobenzoyl)-N^4-trityl-L-asparagine 37

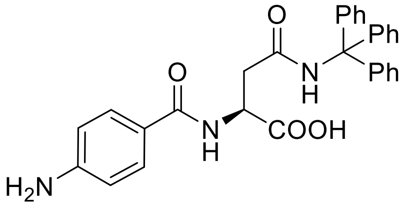

To a stirred solution of **36** (3.66 g, 7 mmol) in EtOH (300 mL), iron powder (1.96 g, 35 mmol) was added at 55 °C followed by NH_4Cl (189 mg, 3.5 mmol) solution in water (15 mL). The reaction was refluxed for 3 h, then it was filtered while hot through a pad of diatomaceous earth and the filter cake was washed by THF (50 mL). The filtrate was concentrated under vacuum near dryness. The residue was diluted with water (30 mL), was filtered, and was washed with diethyl ether (30 mL).

Yield 85%; beige solid; ^{1}H NMR (500 MHz, DMSO-d_6) δ 8.92 (br s, 1H), 8.06 (br s, 1H), 7.57 (m, 2H), 7.17 (m, 15H), 6.56 (m, 2H), 5.67 (br s, 2H), 4.54 (m, 1H), 2.79 (m, 1H), 2.67 (m, 1H); ^{13}C NMR (126 MHz, DMSO-d_6) δ 173.79, 169.22, 166.36, 152.04, 144.84 (3C), 129.08 (2C), 128.70 (6C), 127.64 (6C), 126.56 (3C), 120.68, 112.74 (2C), 69.54, 50.18, 38.28; m/z (ESI+) 494 $[M + H]^+$; t_R = 7.19 min.

N^2-(4-(4-Nitrobenzamido)benzoyl)-N^4-trityl-L-asparagine 38

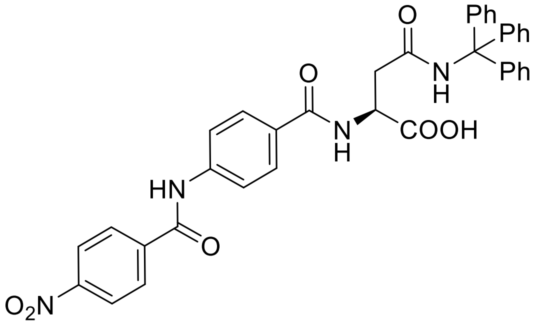

To a stirred ice-cooled solution of **37** (2.96 g, 6 mmol) in a mixture of 1 N NaOH (6 mL) and dioxane (8 mL), 1 N NaOH (6 mL) and a solution of *p*-nitrobenzoyl chloride (1.56 g, 8.4 mmol) in dioxane (18 mL) were added dropwise and concurrently. The reaction mixture was stirred at 0–5 °C for 1 h, and then at room temperature for further 1 h. The reaction was diluted with water (20 mL) and was acidified with HCl to pH 3–4. The mixture was extracted with EtOAc–THF (1:1) (3 × 30 mL). The organic layer was dried over anhydrous $MgSO_4$ and the solvent was removed by vacuum distillation. The crude material was triturated with diethyl ether (30 mL), was filtered, and was washed with diethyl ether (2 × 30 mL). Yield 75%; beige solid; ^{1}H NMR (500 MHz, DMSO-d$_6$) δ 12.73 (br s, 1H), 10.83 (br s, 1H), 8.68 (br s, 1H), 8.66 (d, *J* = 7.9 Hz, 1H), 8.39 (d, *J* = 8.8 Hz, 2H), 8.22 (d, *J* = 8.8 Hz, 2H), 7.92 (m, 4H), 7.18 (m, 15H), 4.70 (m, 1H), 2.89 (dd, *J* = 14.8, 10.1 Hz, 1H), 2.76 (dd, *J* = 14.8, 4.7 Hz, 1H); ^{13}C NMR (126 MHz, DMSO-d$_6$) δ 173.23, 168.81, 165.49, 164.26, 149.30, 144.71 (3C), 141.59, 140.35, 129.39 (2C), 129.21, 128.58 (6C), 128.22 (2C), 127.47 (6C), 126.39 (3C), 123.65 (2C), 119.61 (2C), 69.40, 50.03, 37.81; m/z (ESI+) 643 $[M + H]^+$; t_R = 8.34 min.

***tert*-Butyl (*S*)-4-(2-hydroxy-3-methoxy-4-(4-(2-(4-(4-nitrobenzamido)benzamido)-4-oxo-4-(tritylamino)butanamido)benzamido)benzamido)-3-isopropoxybenzoate 39**

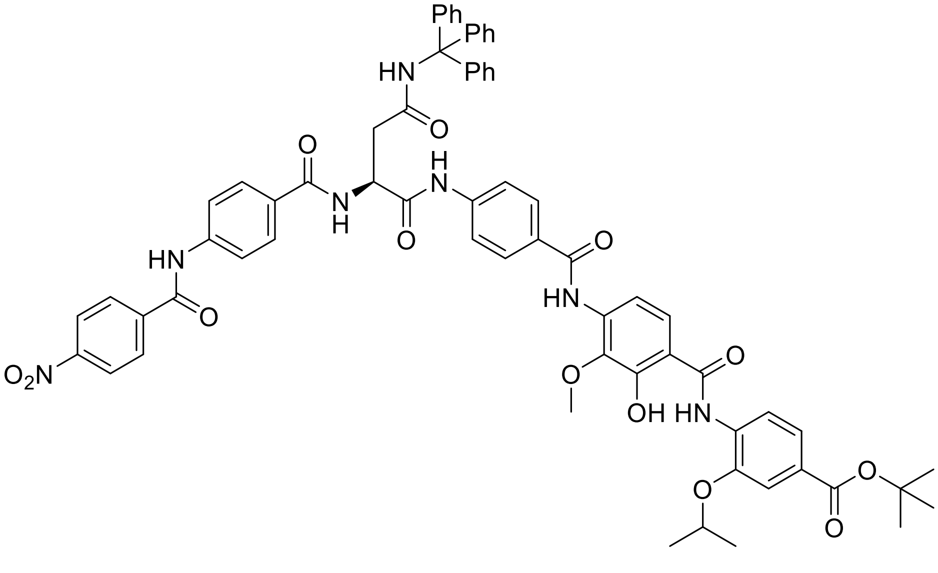

Compound **39** was synthesized according to the general procedure for amide coupling using dichlorotriphenylphosphorane starting with the *N*-protected carboxylic acid **38** and the *C*-protected amine **34**. The crude material was purified using flash chromatography (SiO_2, DCM–MeOH = 98:2).

Yield 75%; beige solid; m/z (ESI+) 1161 $[M + H]^+$; t_R = 10.55 min.

(*S*)-4-(4-(4-(4-Amino-2-(4-(4-nitrobenzamido)benzamido)-4-oxobutanamido)benzamido)-2-hydroxy-3-methoxybenzamido)-3-isopropoxybenzoic acid 16

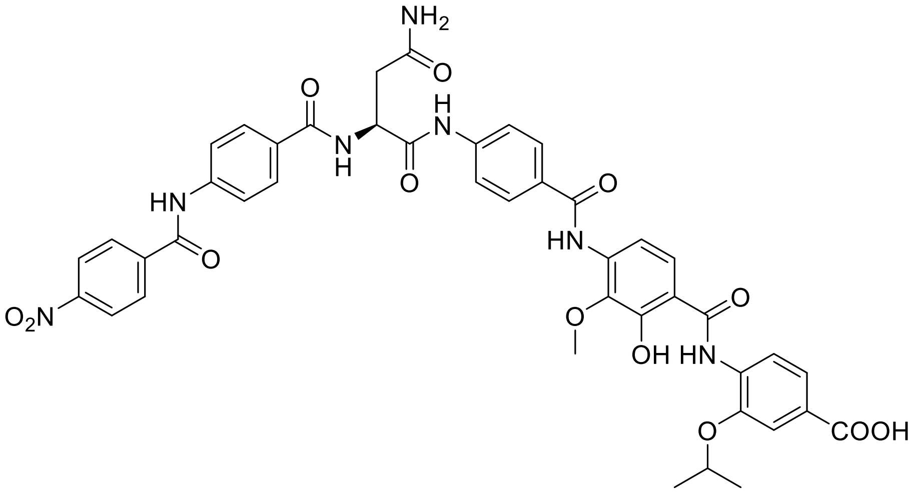

To a stirred solution of **39** (58 mg, 0.5 mmol) in DCM (8.5 mL), TFA (1.5 mL) was added. The reaction was stirred at room temperature for 2 h. Solvent was removed under reduced pressure. The residue was purified by preparative RP-HPLC.

Yield 97%; beige solid; ^{1}H NMR (500 MHz, DMSO-d_6) δ 11.43 (br s, 1H), 11.11 (br s, 1H), 10.80 (br s, 2H), 10.45 (br s, 1H), 9.61 (br s, 1H), 8.68 (d, *J* = 7.3 Hz, 1H), 8.58 (d, *J* = 8.2 Hz, 1H), 8.38 (d, *J* = 8.5 Hz, 2H), 8.21 (d, *J* = 8.5 Hz, 2H), 7.96 (d, *J* = 8.8 Hz, 2H), 7.94 (d, *J* = 8.5 Hz, 2H), 7.90 (d, *J* = 8.5 Hz, 2H), 7.80 (m, 3H), 7.57 (m, 3H), 7.41 (br s, 1H), 6.99 (br s, 1H), 4.93 (m, 1H), 4.76 (septet, *J* = 6.0 Hz, 1H), 3.79 (s, 3H), 2.70 (m, 2H), 1.39 (d, *J* = 6.0 Hz, 6H); ^{13}C NMR (126 MHz, DMSO-d_6) δ 171.64, 170.88, 167.32, 166.11, 165.13, 164.57, 163.69, 150.96, 149.46, 146.30, 142.52, 141.74, 140.69, 140.51, 135.77, 133.80, 129.53 (2C), 129.38, 128.88 (2C), 128.84, 128.59 (2C), 125.68, 125.63, 123.83 (2C), 122.87, 119.84 (2C), 119.44, 118.97 (2C), 116.69, 113.98 (2C), 71.93, 60.52, 51.83, 36.96, 21.91 (2C); HRMS (ESI+) calcd. for $C_{43}H_{40}N_7O_{13}$ $[M + H]^+$: 862.26841, found: 862.26670; t_R = 7.45 min.

2D-NOESY Measurement in a Cryoprotective Mixture

NMR spectrum was recorded on Bruker DRX-500 spectrometer at 300 K. Compounds **26** and **70** were prepared as 20 mM solution in H_2O/DMSO-d_6 (20% V/V). Samples were degased *via* flushing the tube with nitrogen gas, cooling in liquid nitrogen until freezing, then application of vacuum until attaining the room temperature. The process was repeated three times and then the tubes were covered and sealed with parafilm.

Conformational Interconversion at Physiological Temperature

^{1}H NMR spectra were determined for compounds **26** and **70** (20 mM solution in $CDCl_3$) at 293 and 310 K. Analysis of spectra was performed using ACD/NMR Processor Academic Edition version 12.01 (Figure S29 and S30).

Effect of Temperature on Rigidity 2D-NOESY was measured for compound **8** in DMSO-d_6 at diffrent temperaturs (300, 320, 340, and 360 K). No change in NOESY spectra up to 340 K. At 360 K, a very weak cross peak between the C4–NH and the pyridine C3–H started to appear (Figure S21 and S22).

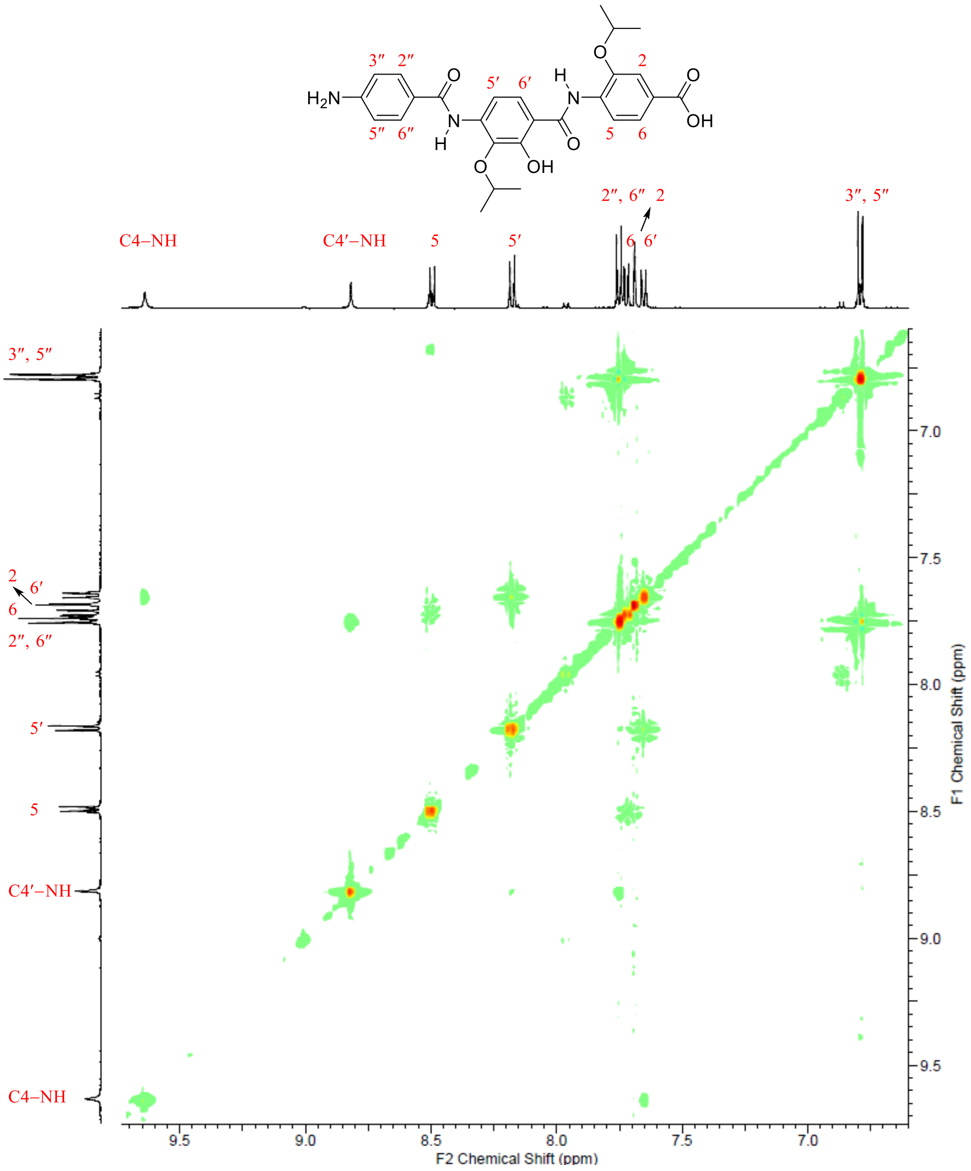

Figure S1. 2D-NOESY spectrum of cystobactamid 507 (**2**) in acetone-d_6.

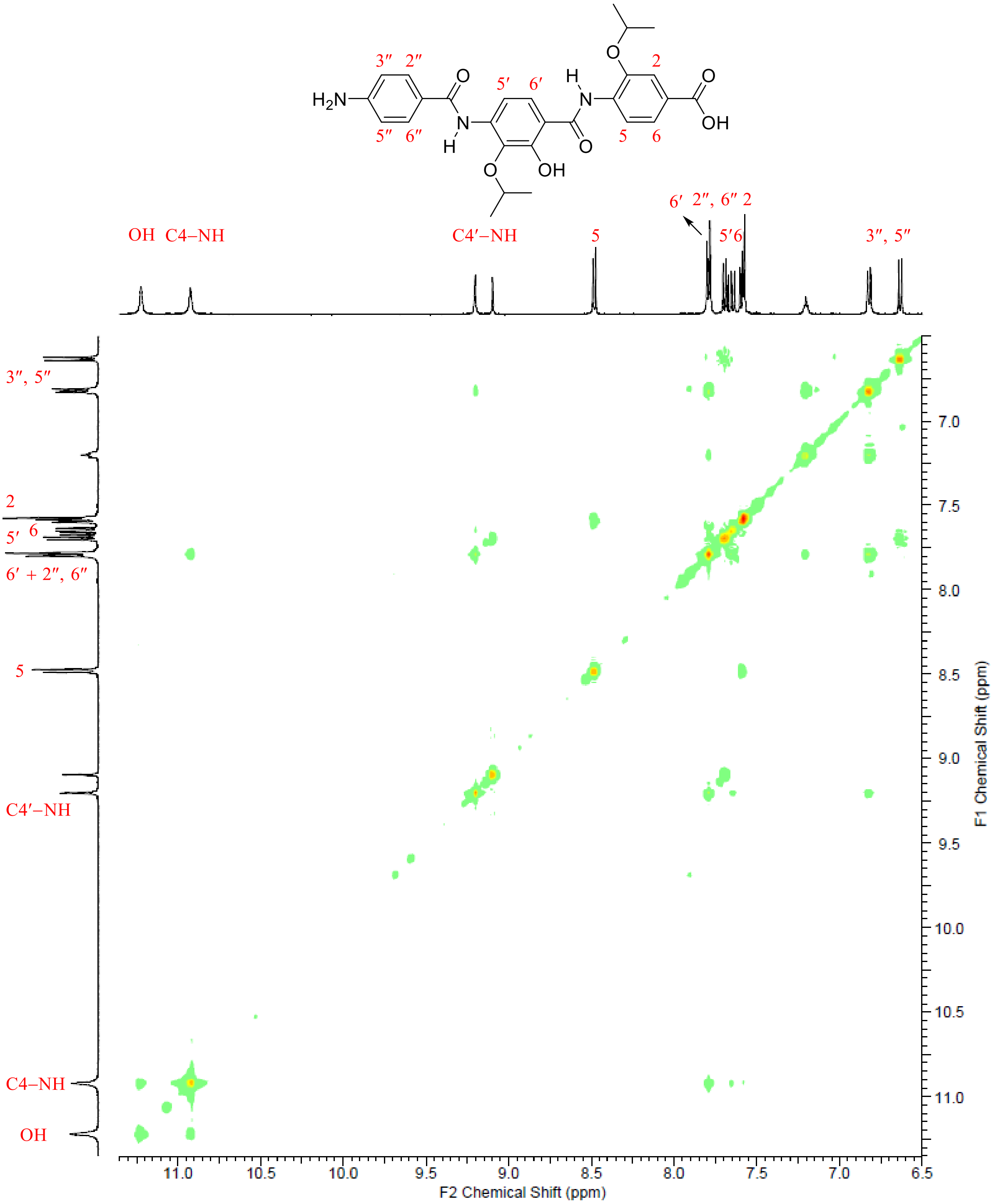

Figure S2. 2D-NOESY spectrum of cystobactamid 507 (**2**) in DMSO-d_6.

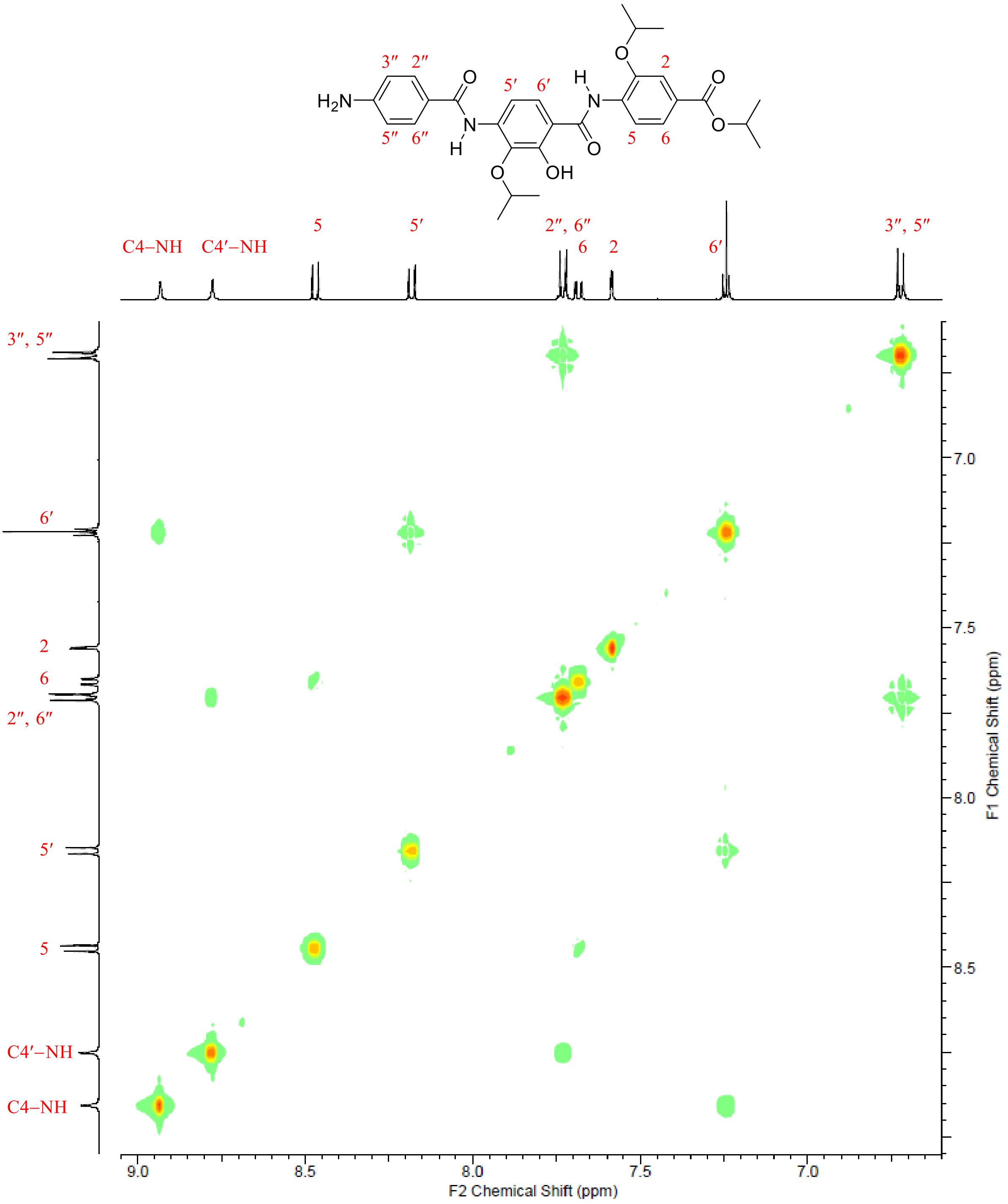

Figure S3. 2D-NOESY spectrum of compound **26** in $CDCl_3$.

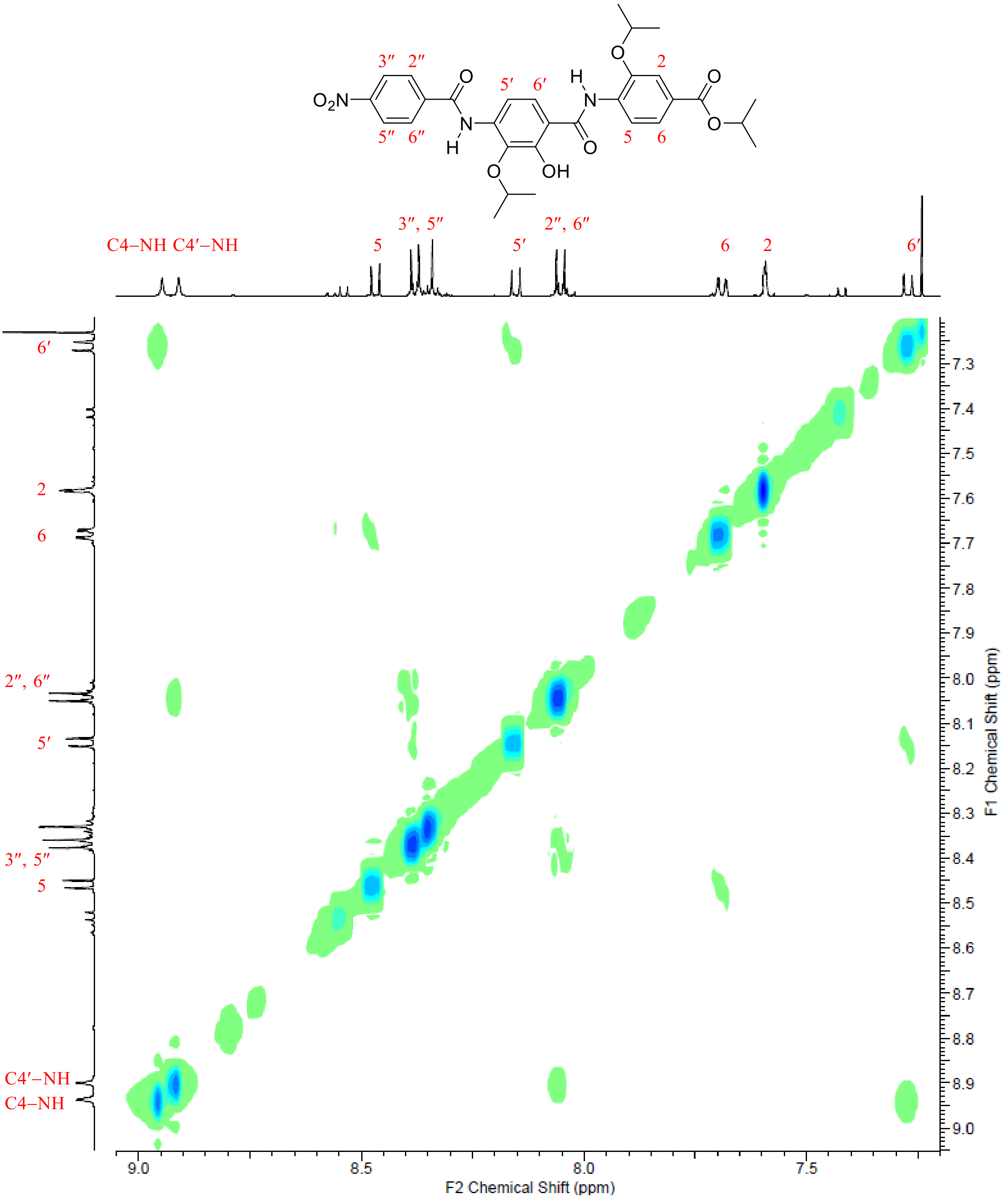

Figure S4. 2D-NOESY spectrum of compound **25** in $CDCl_3$.

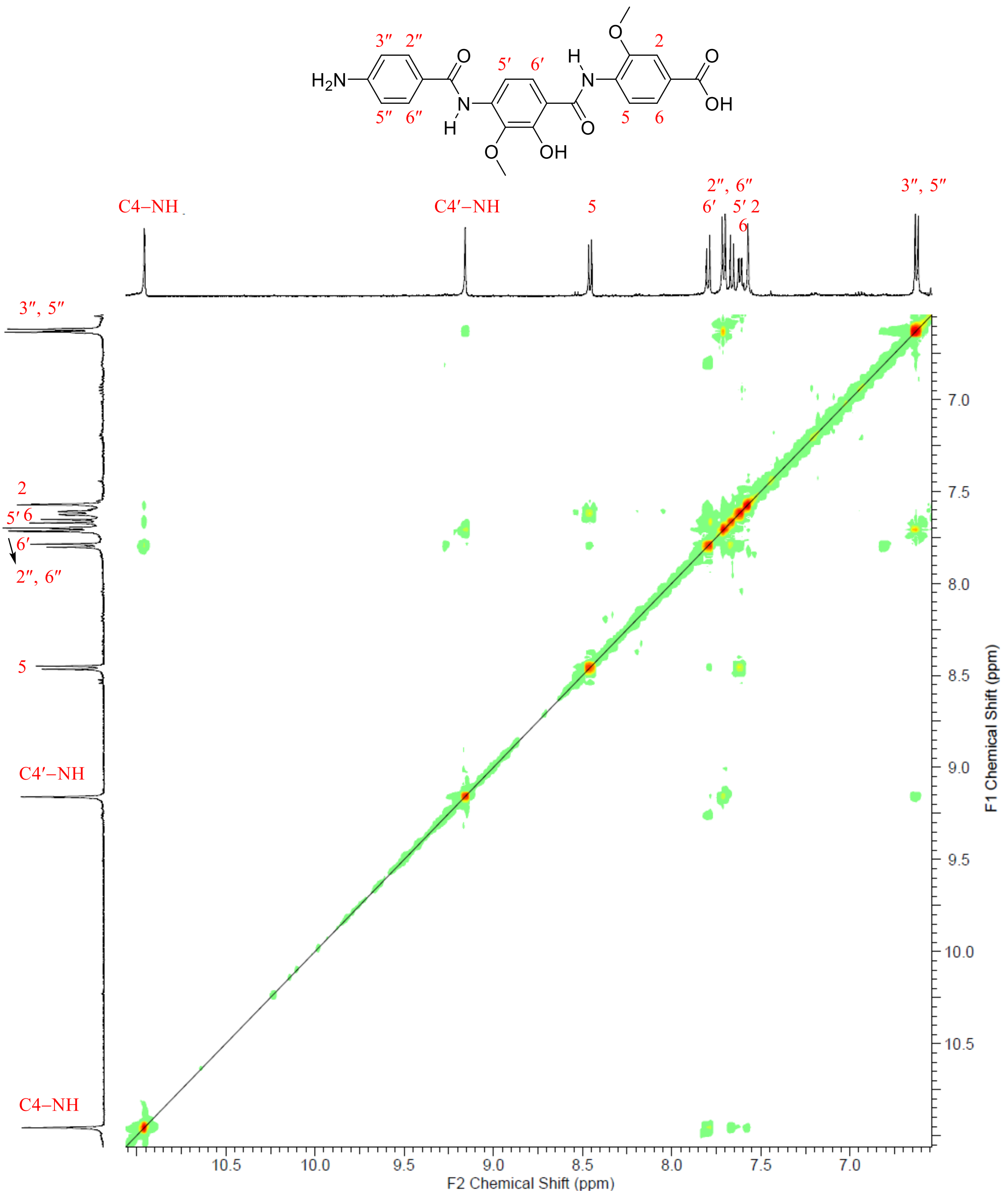

Figure S5. 2D-NOESY spectrum of compound **3** in DMSO-d$_6$.

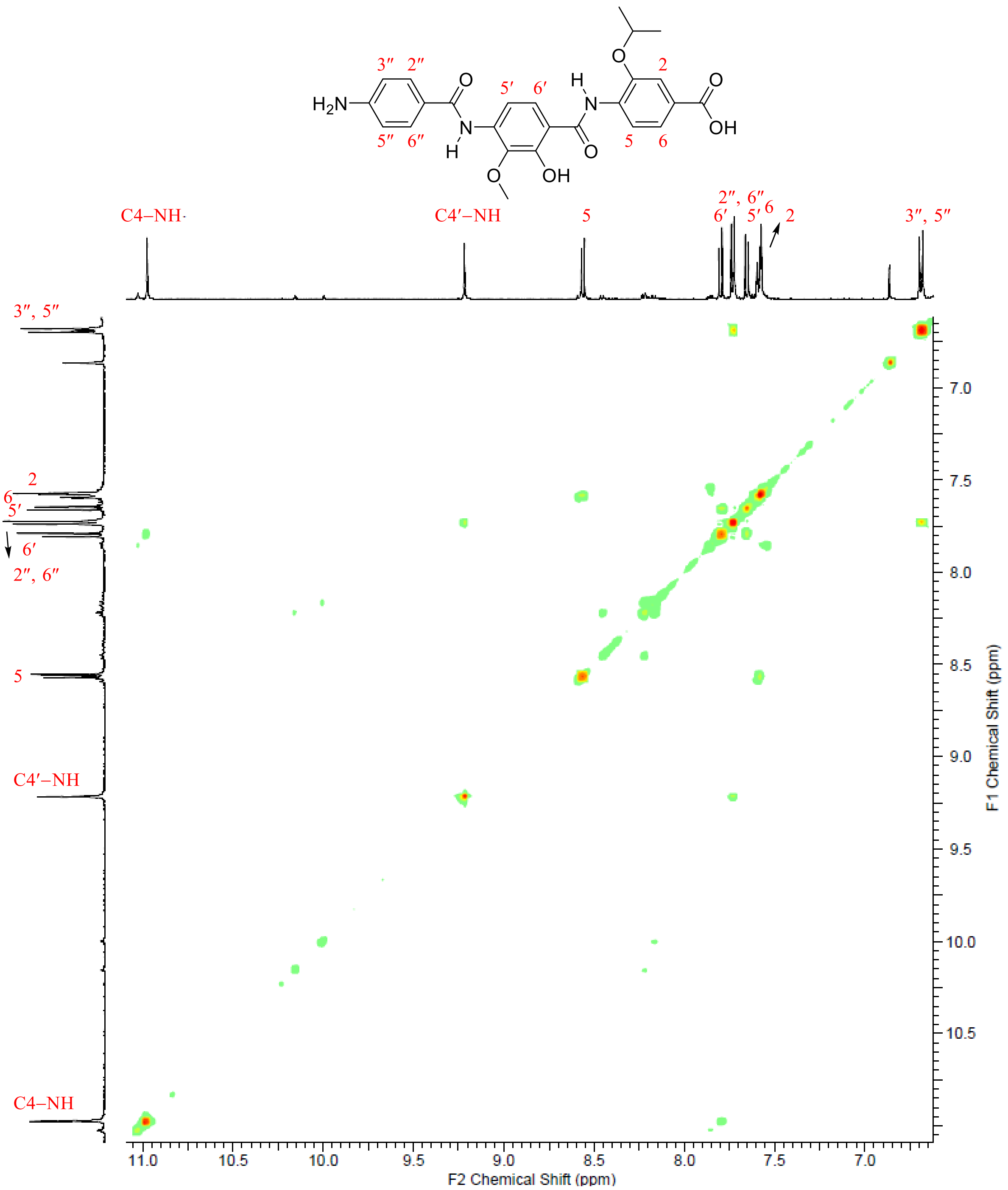

Figure S6. 2D-NOESY spectrum of compound **4** in DMSO-d_6.

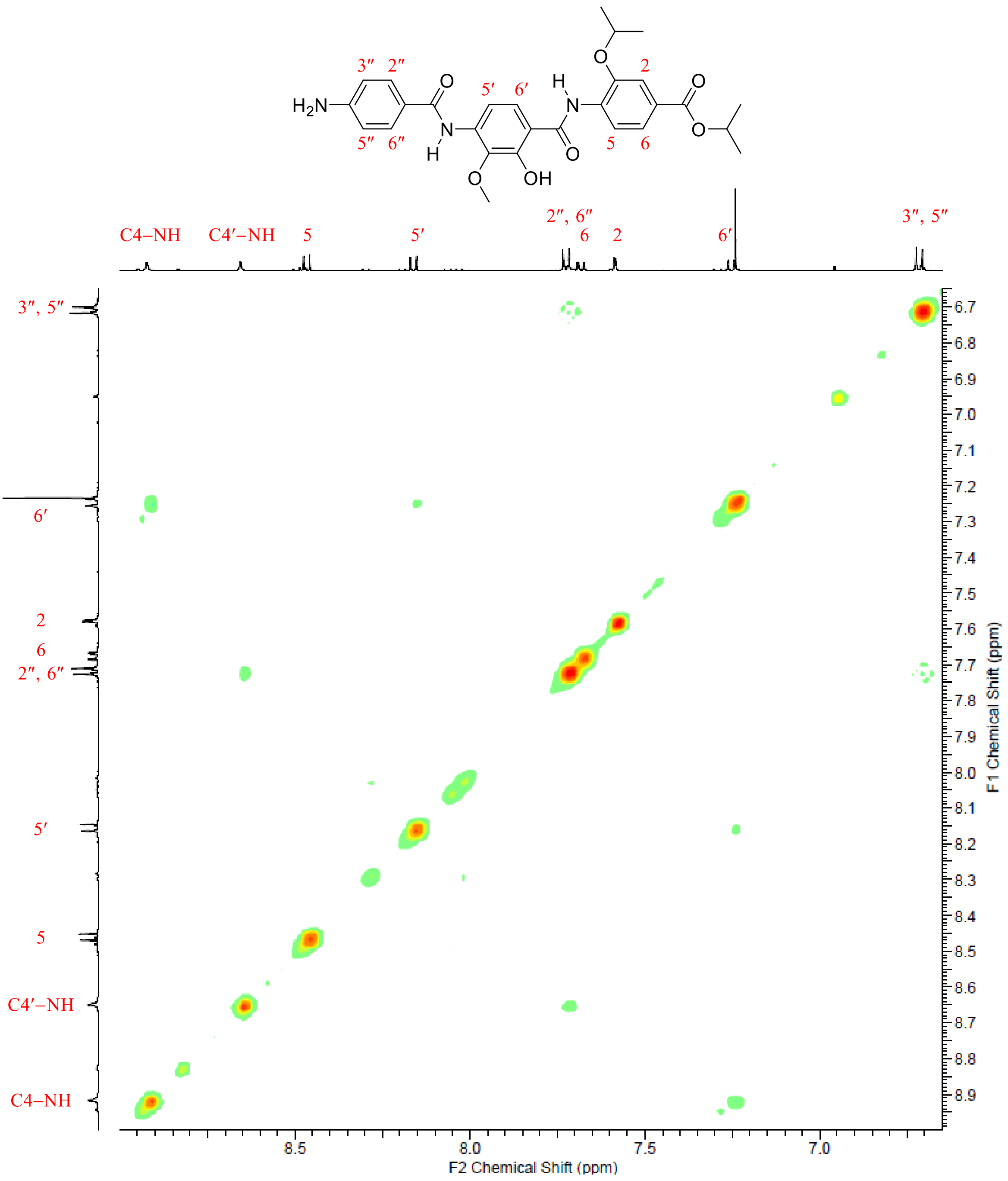

Figure S7. 2D-NOESY spectrum of compound **78** in $CDCl_3$.

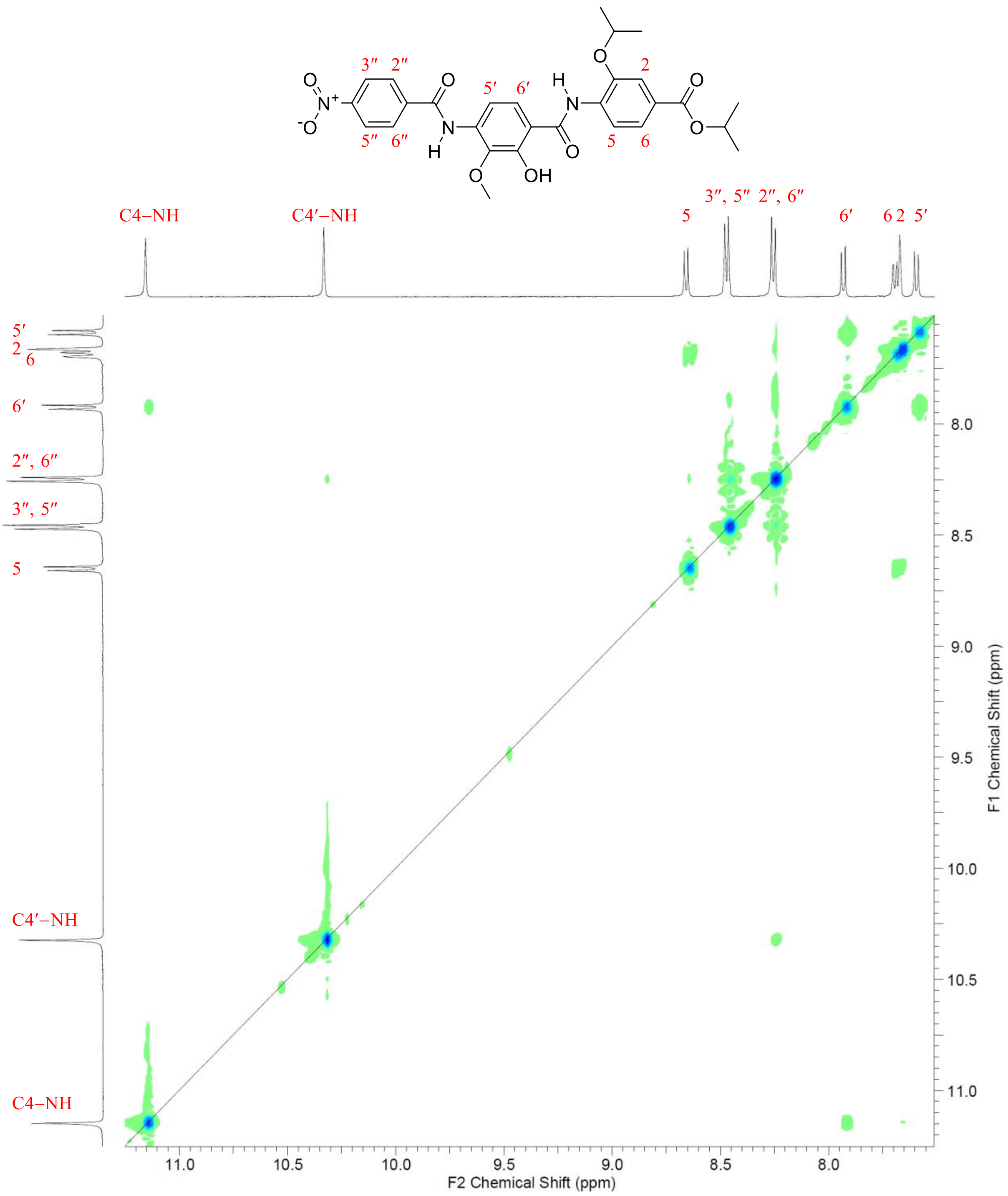

Figure S8. 2D-NOESY spectrum of compound **70** in a cryoprotective mixture (20% H_2O/DMSO-d_6).

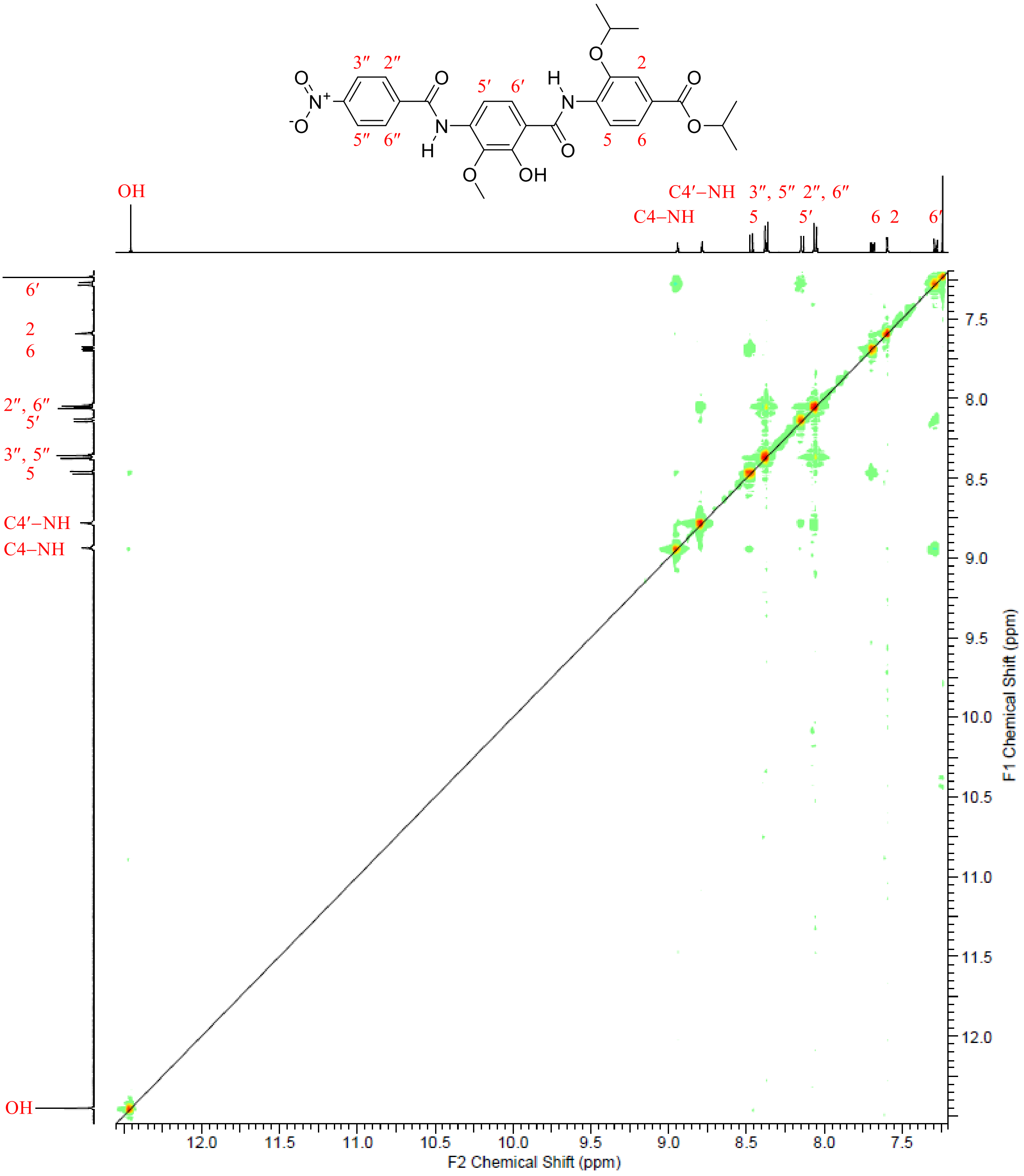

Figure S9. 2D-NOESY spectrum of compound **70** in $CDCl_3$.

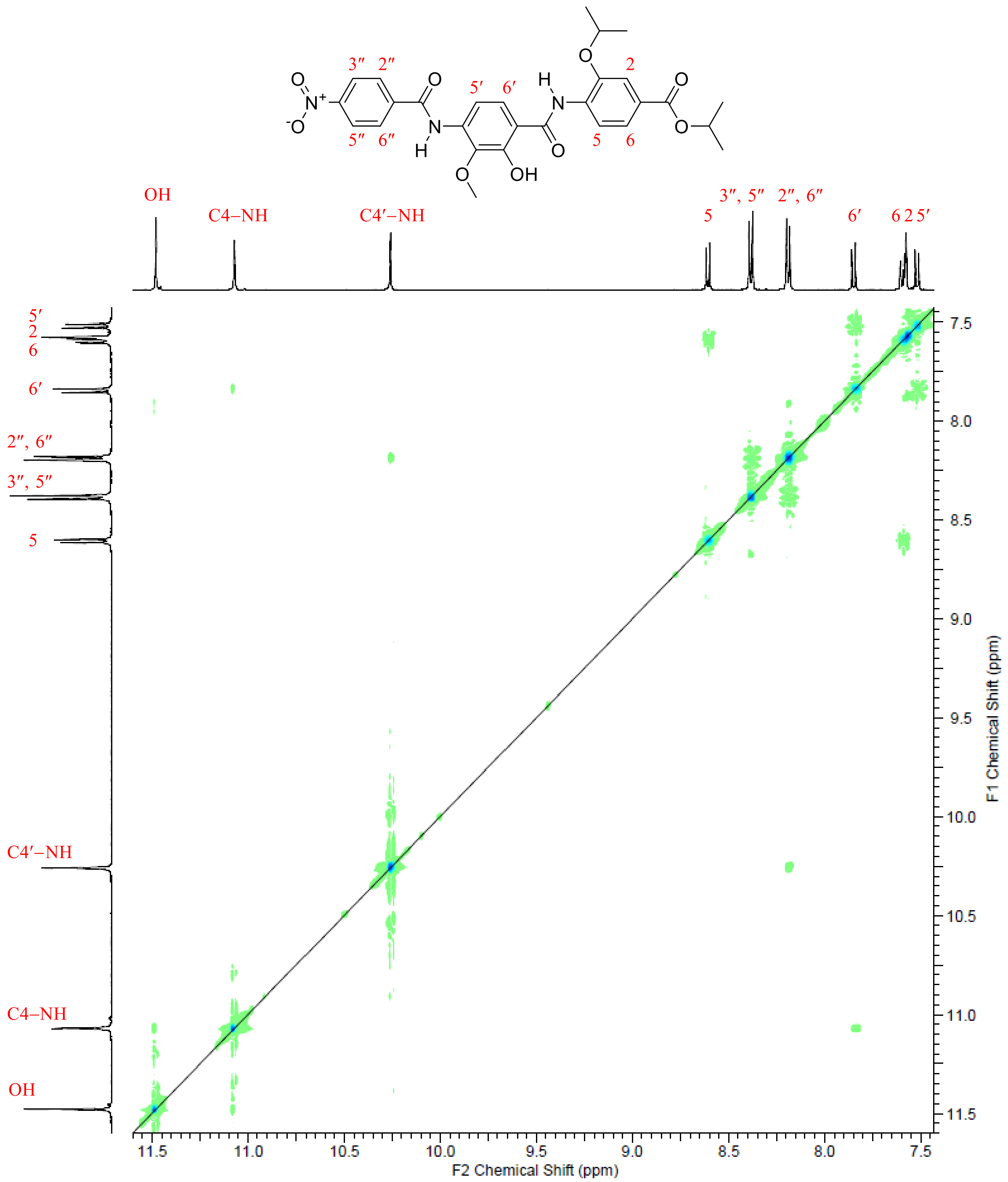

Figure S10. 2D-NOESY spectrum of compound **70** in DMSO-d_6.

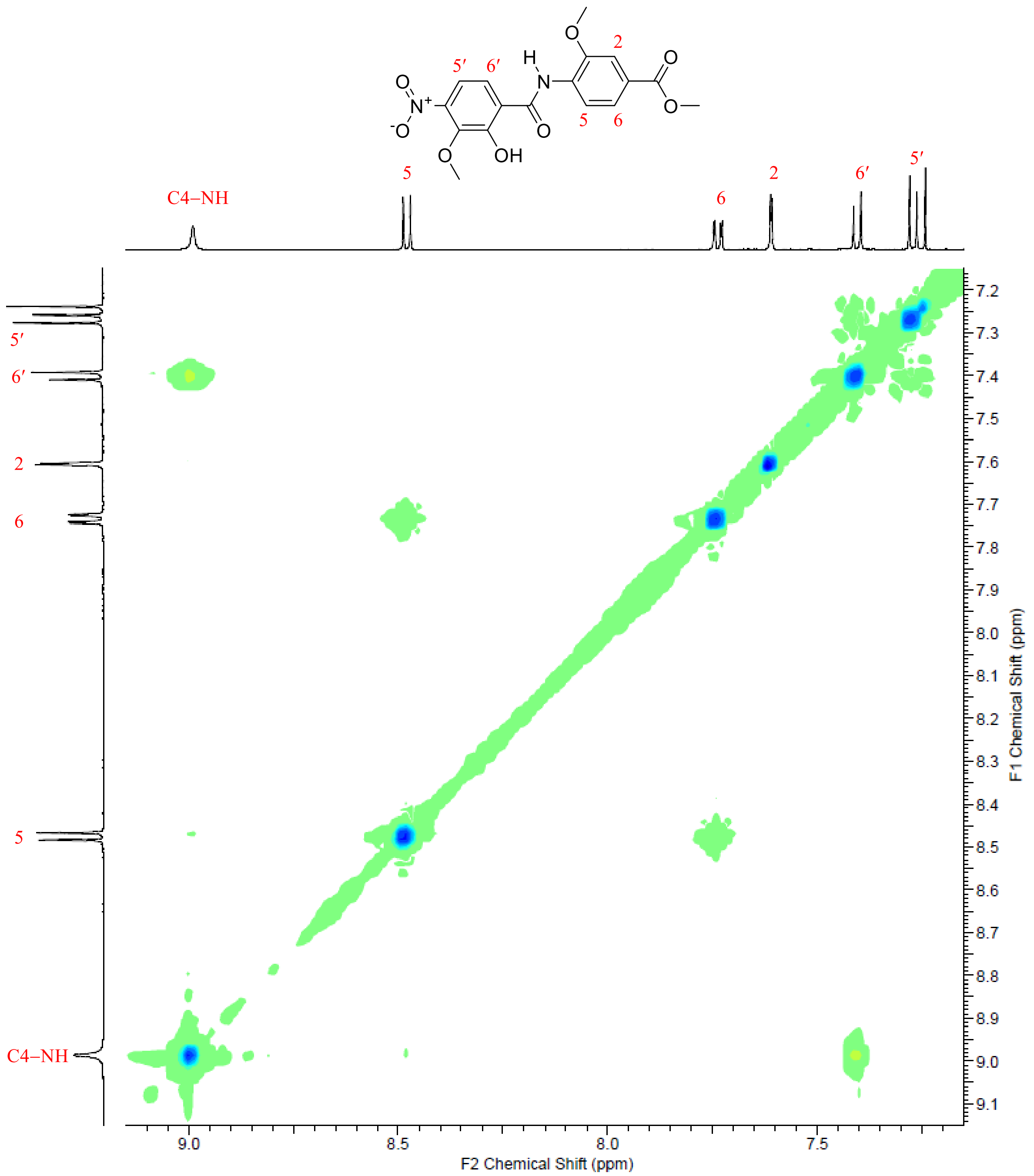

Figure S11. 2D-NOESY spectrum of compound **97** in $CDCl_3$.

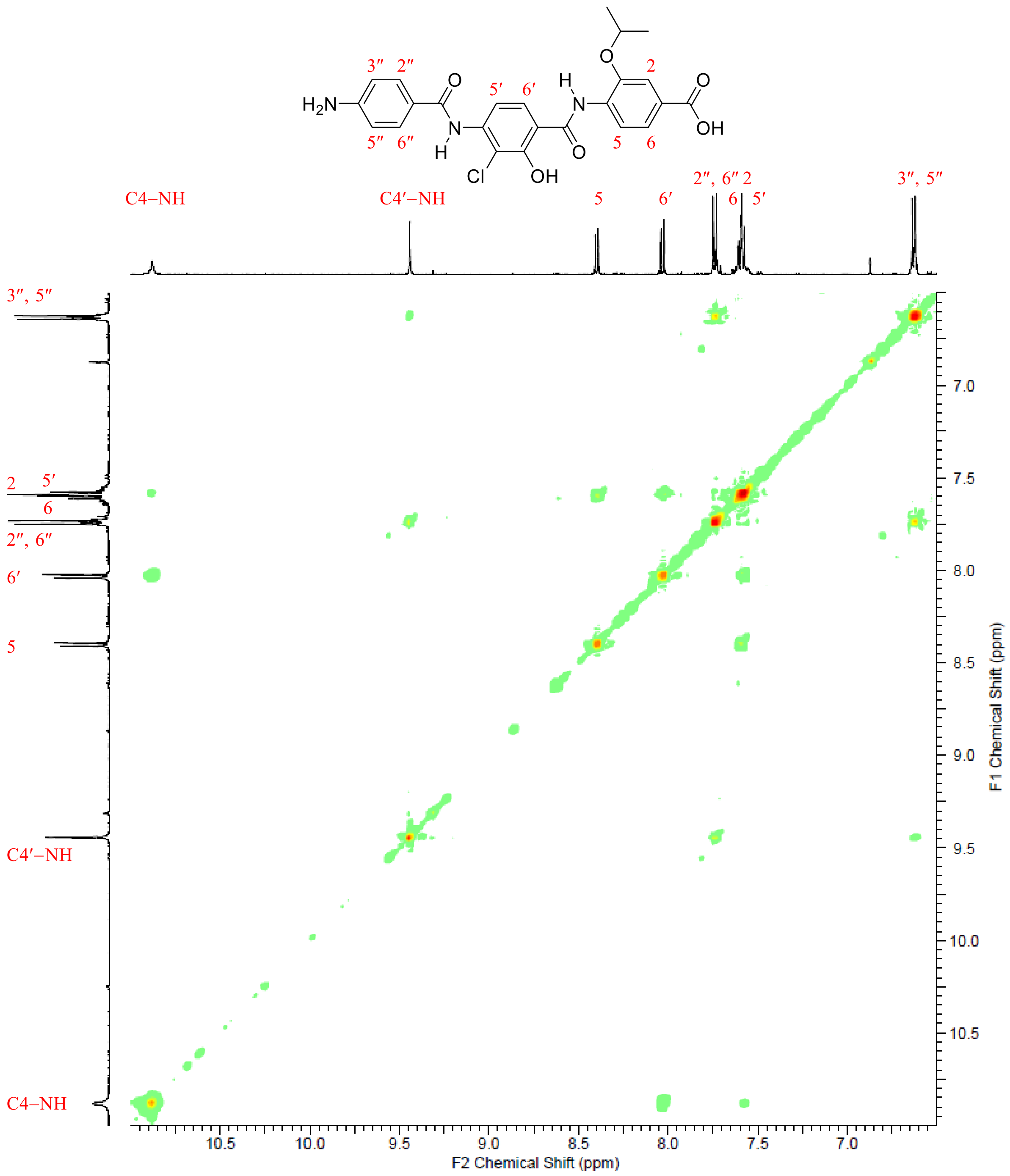

Figure S12. 2D-NOESY spectrum of compound **5** in DMSO-d6.

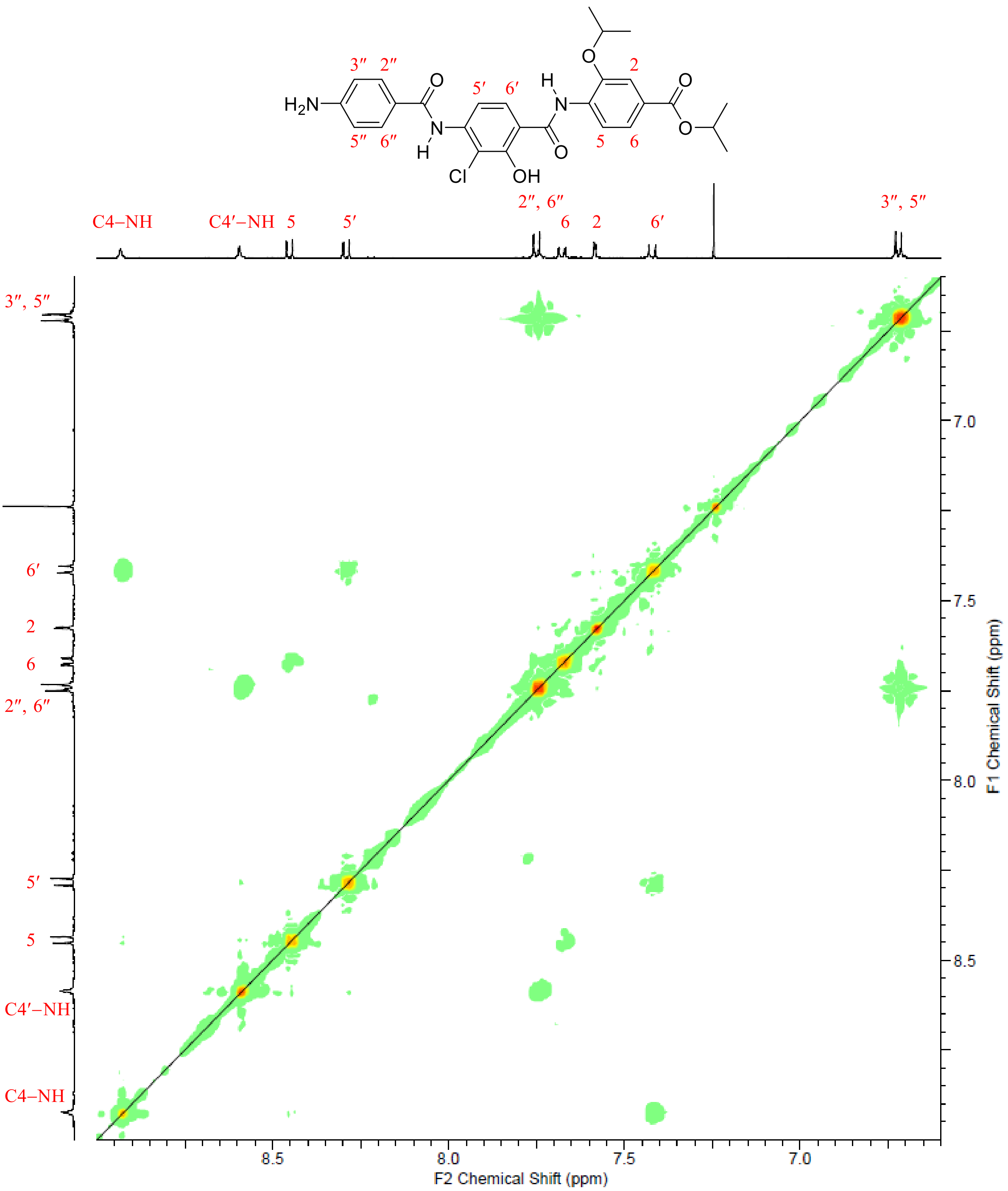

Figure S13. 2D-NOESY spectrum of compound **88** in $CDCl_3$.

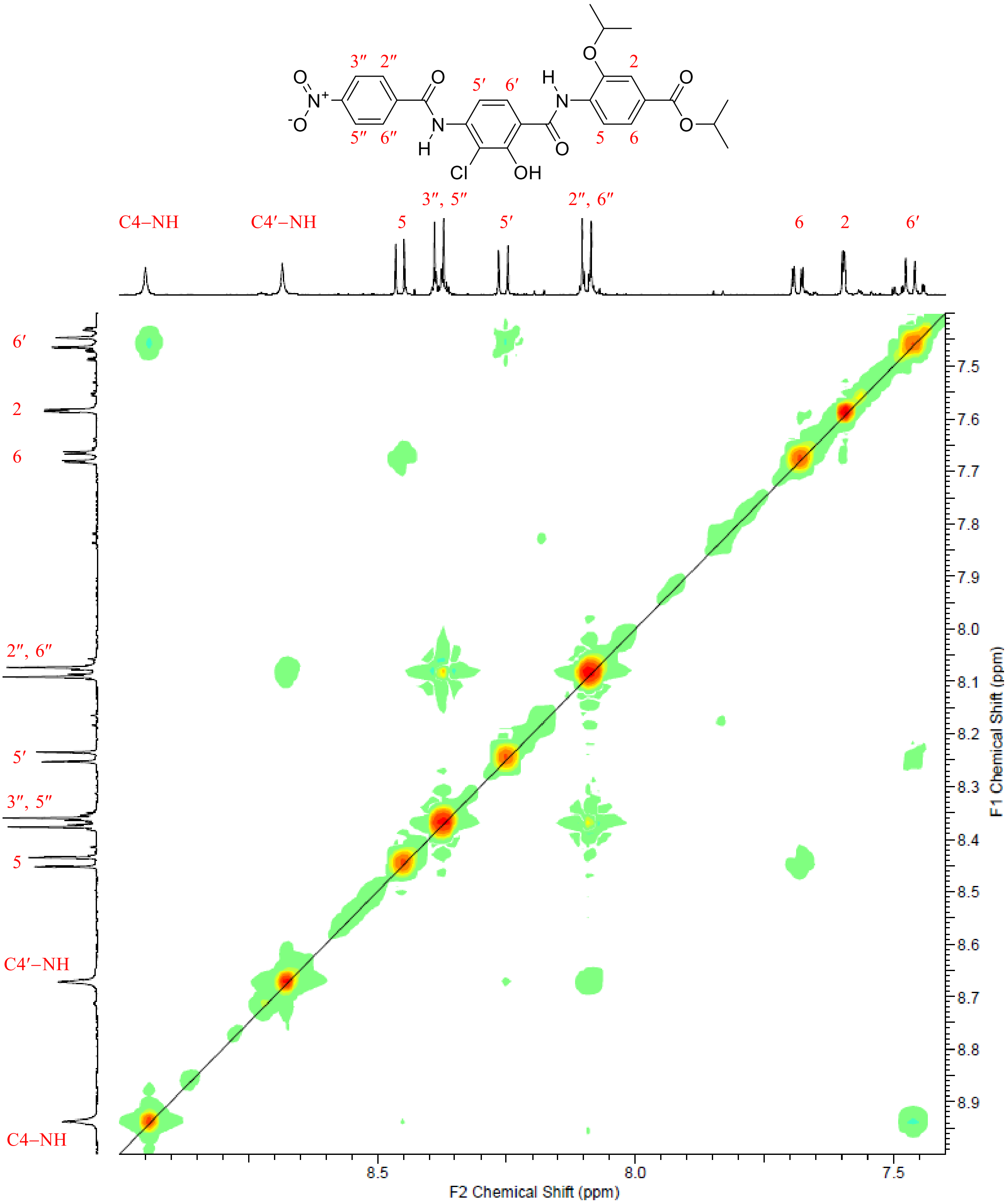

Figure S14. 2D-NOESY spectrum of compound **87** in $CDCl_3$.

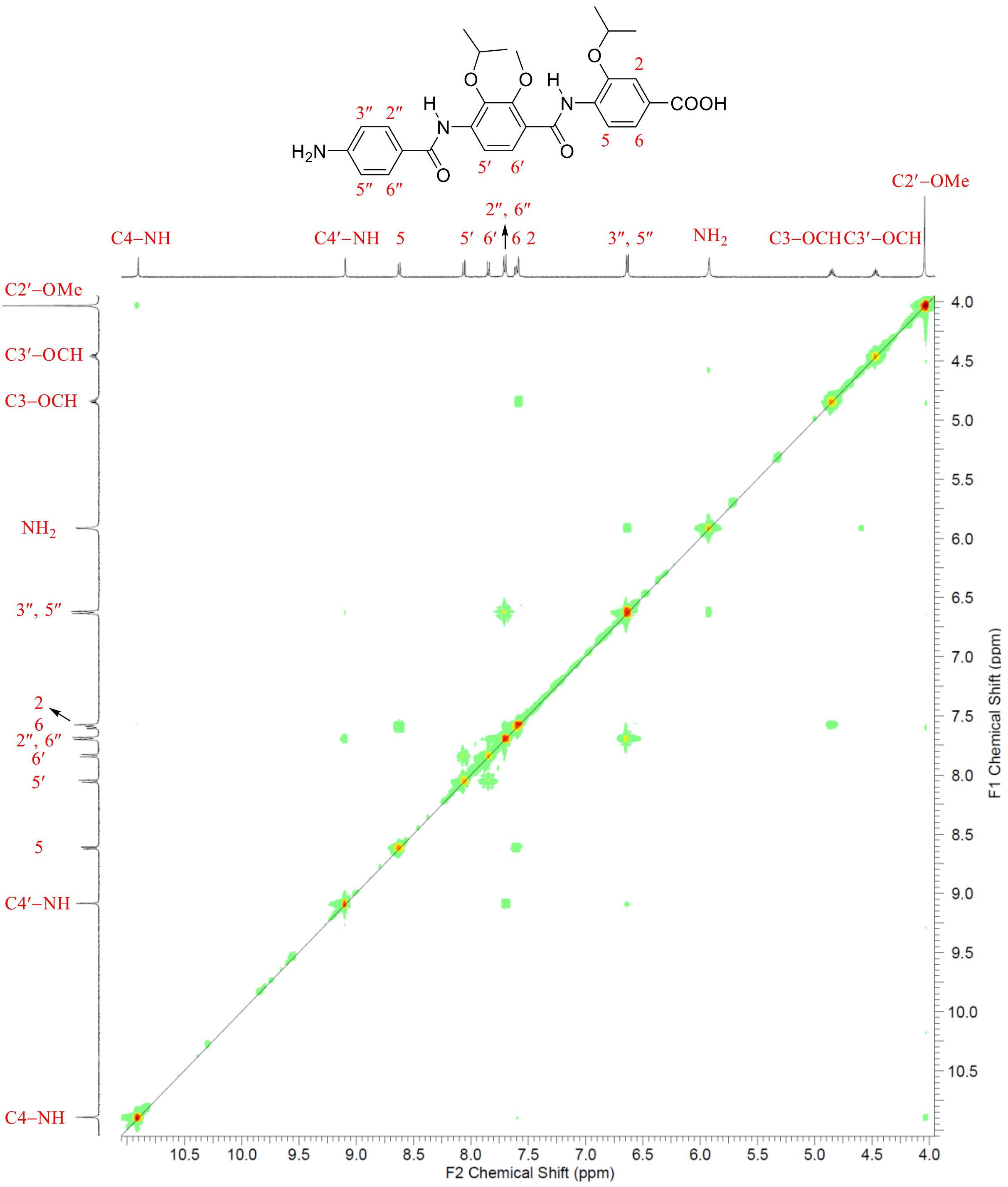

Figure S15. 2D-NOESY spectrum of compound **6** in DMSO-d_6.

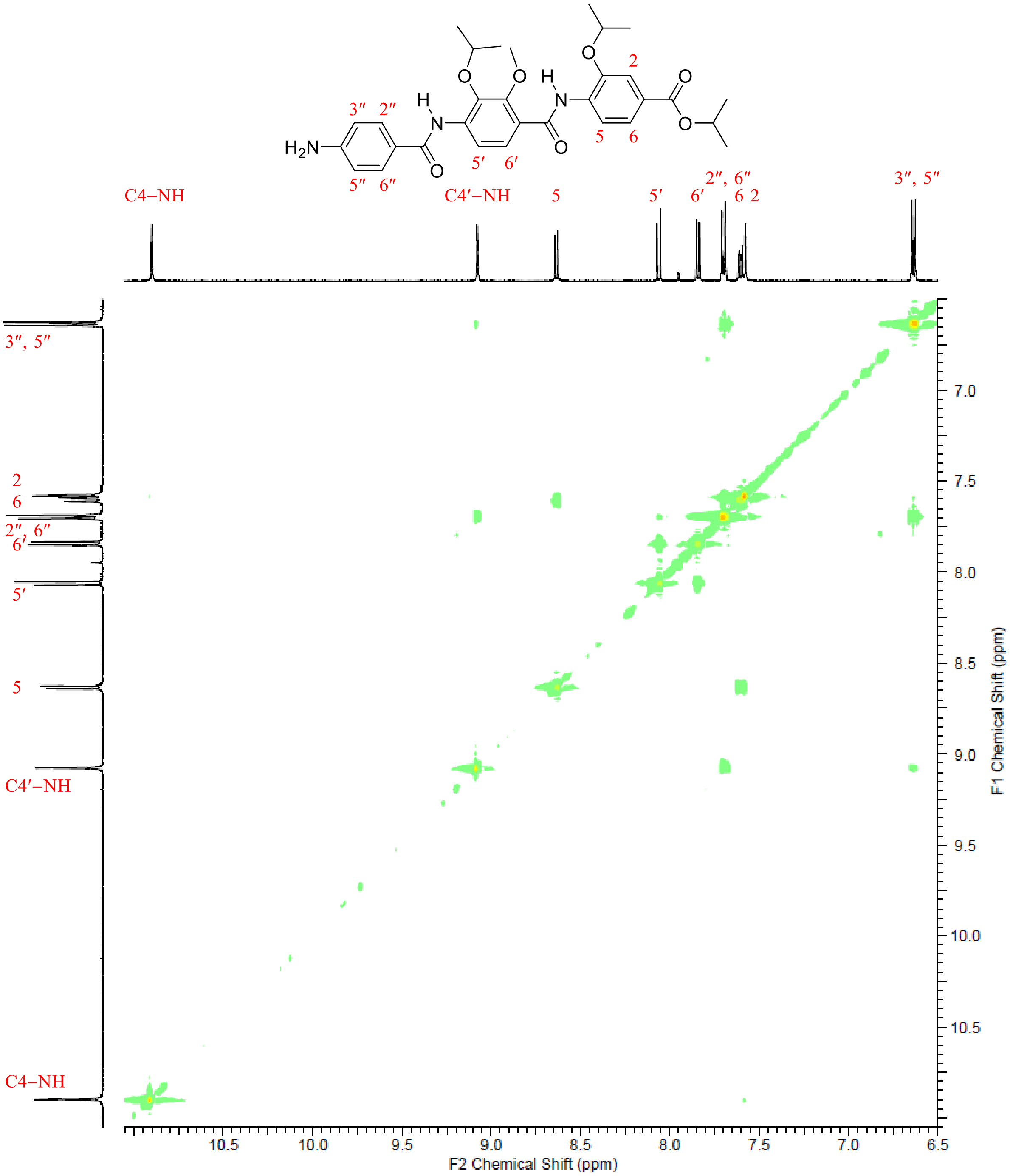

Figure S16. 2D-NOESY spectrum of compound **79** in DMSO-d_6.

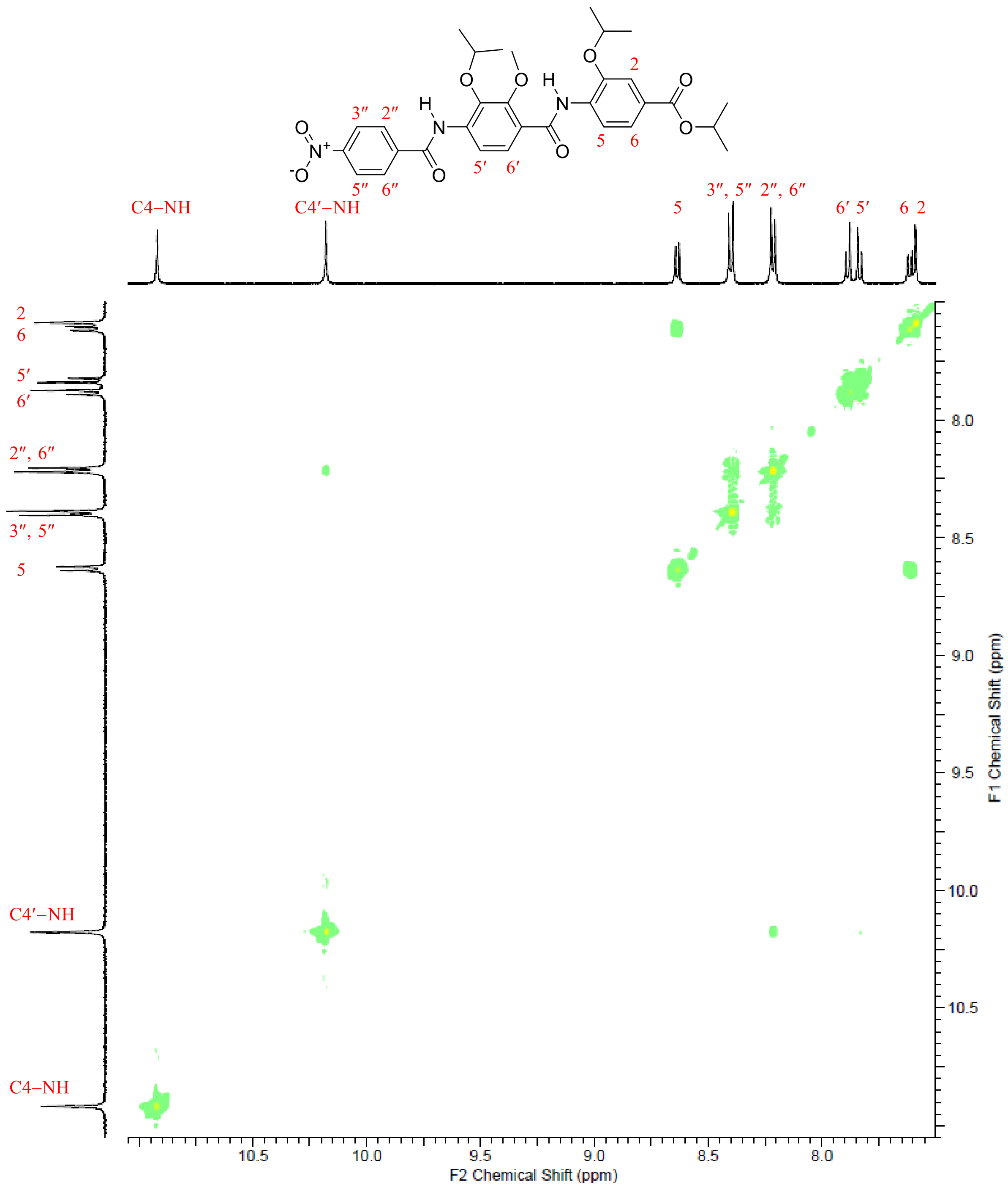

Figure S17. 2D-NOESY spectrum of compound **77** in DMSO-d_6.

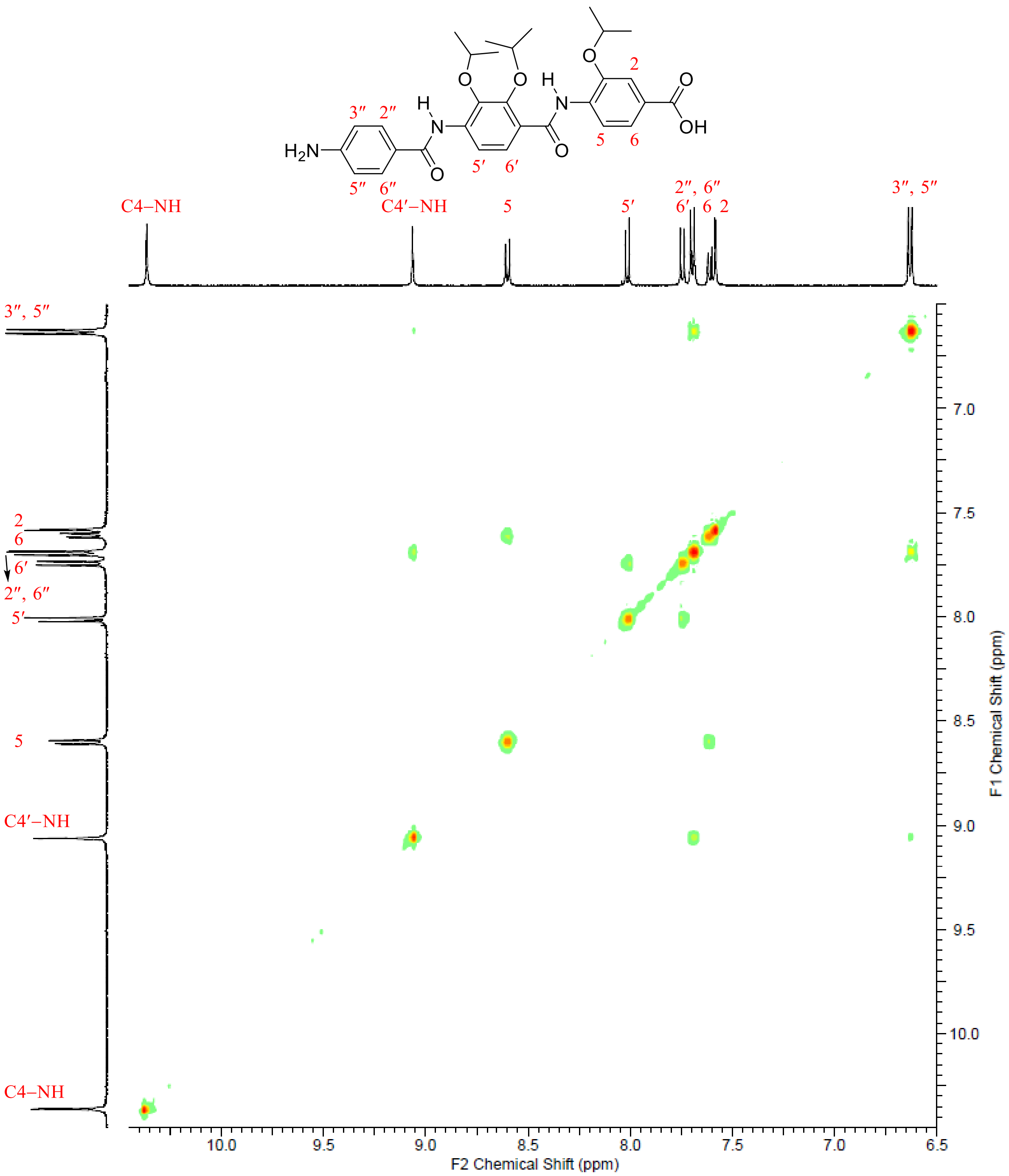

Figure S18. 2D-NOESY spectrum of compound **7** in DMSO-d_6.

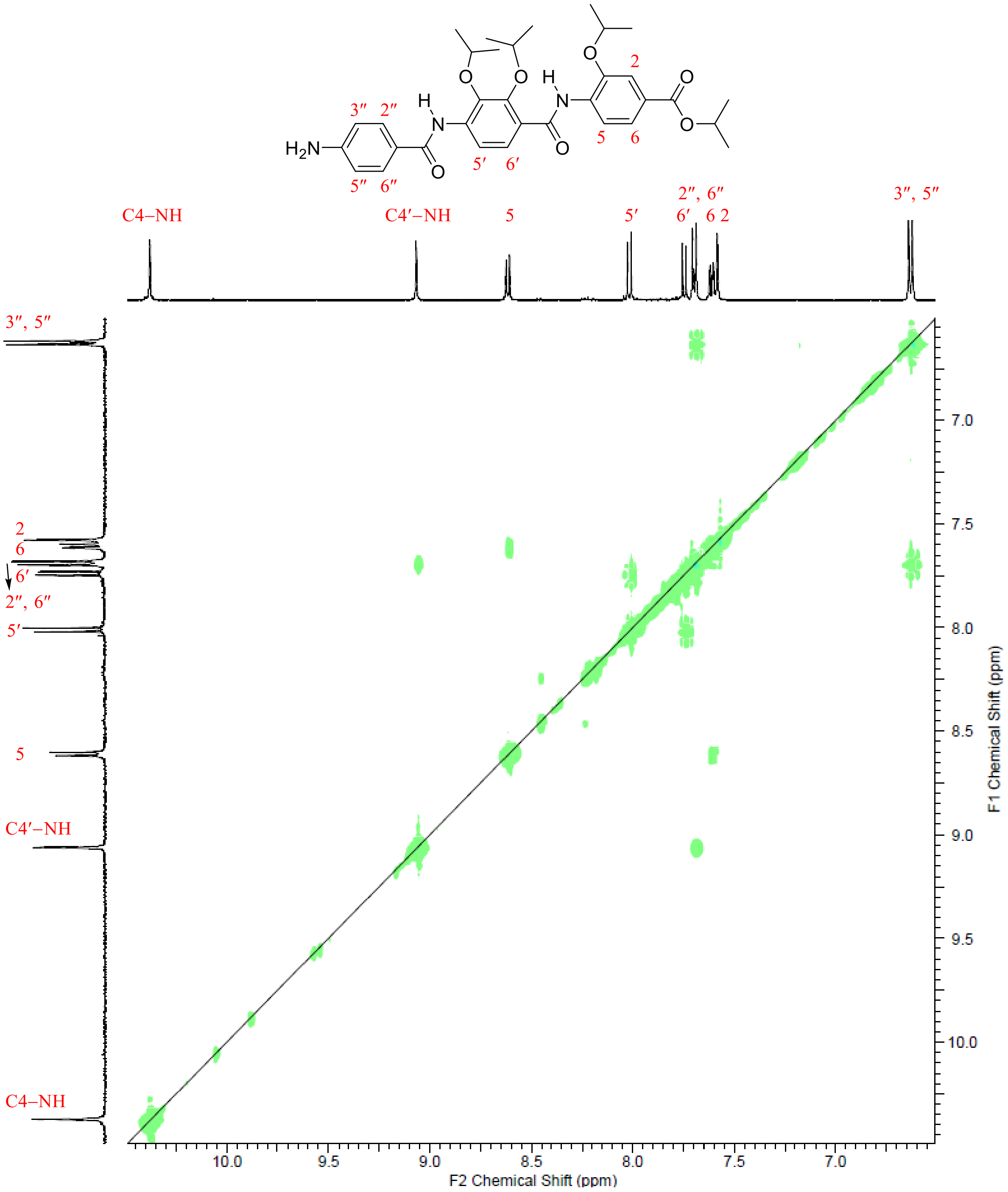

Figure S19. 2D-NOESY spectrum of compound **80** in DMSO-d$_6$.

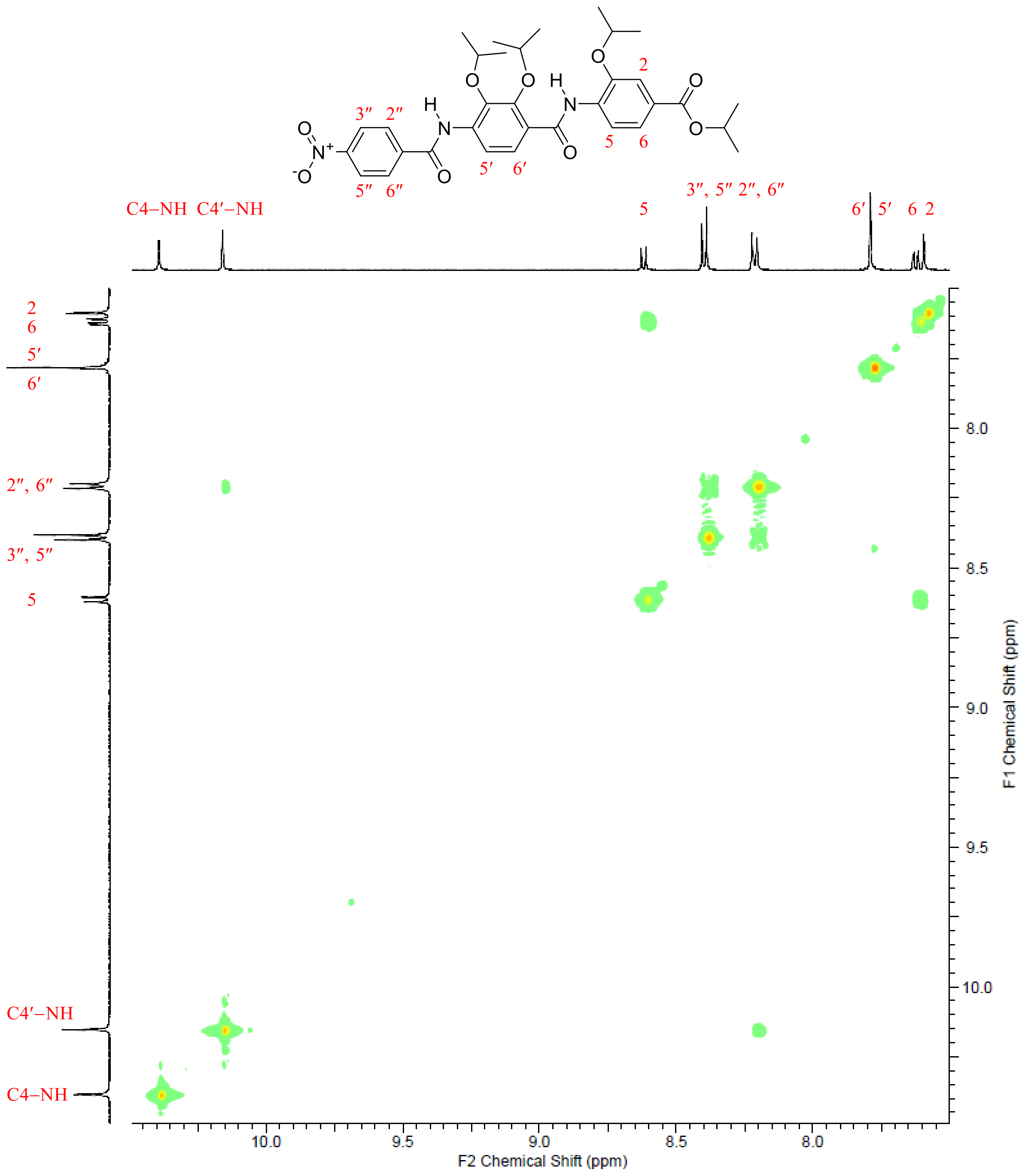

Figure S20. 2D-NOESY spectrum of compound **72** in DMSO-d6.

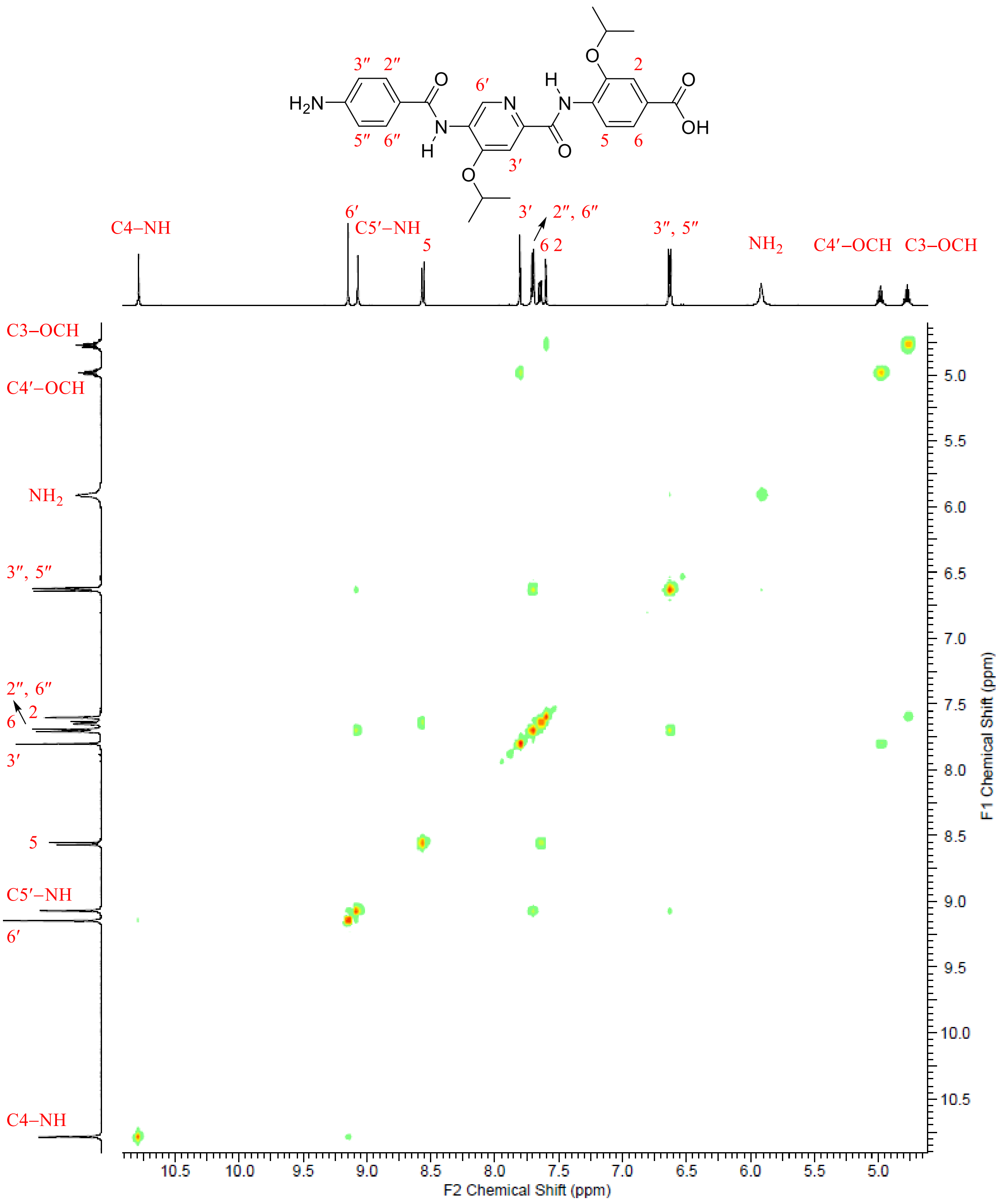

Figure S21. 2D-NOESY spectrum of compound **8** in DMSO-d_6 at 300 K.

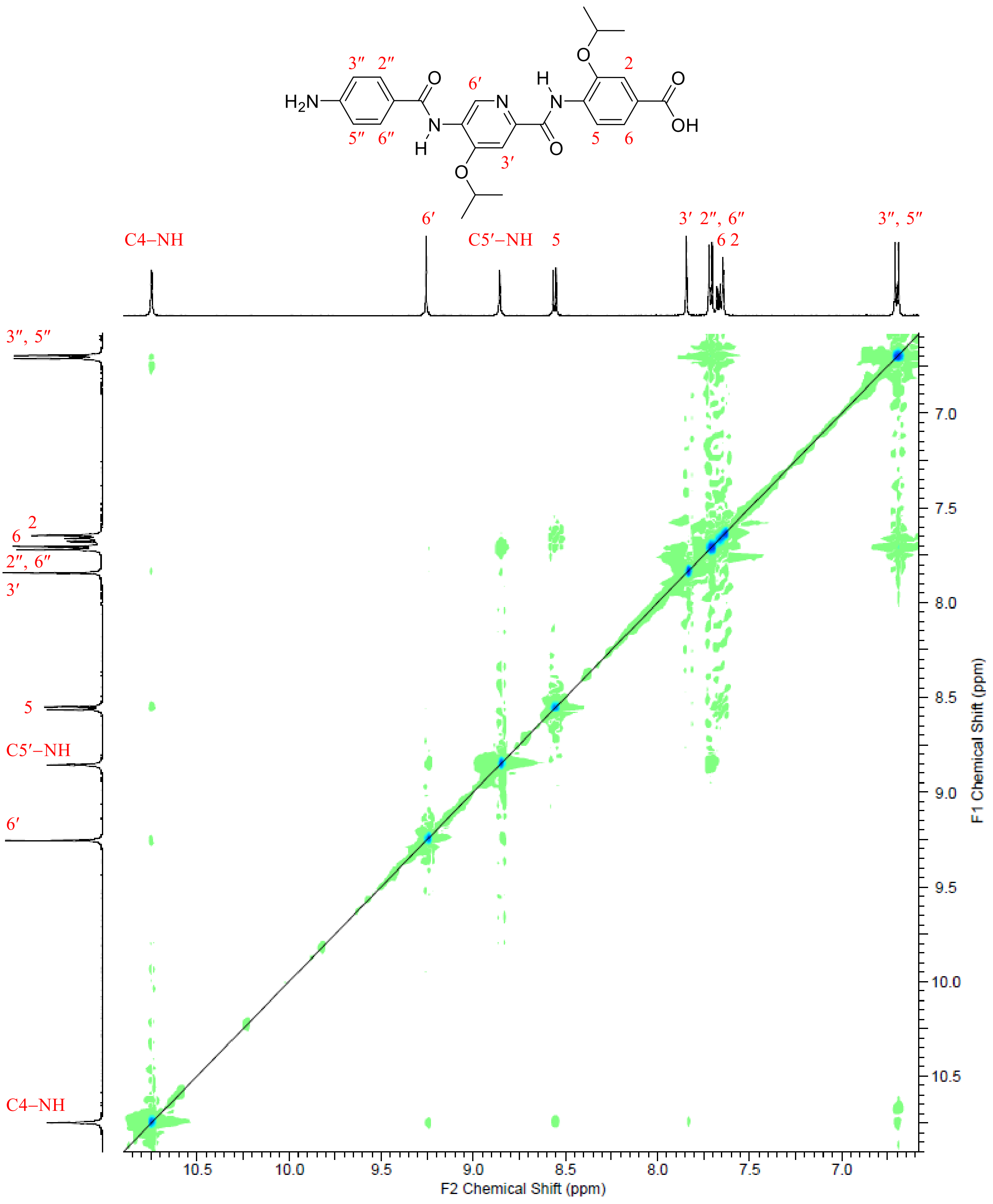

Figure S22. 2D-NOESY spectrum of compound **8** in DMSO-d_6 at 360 K.

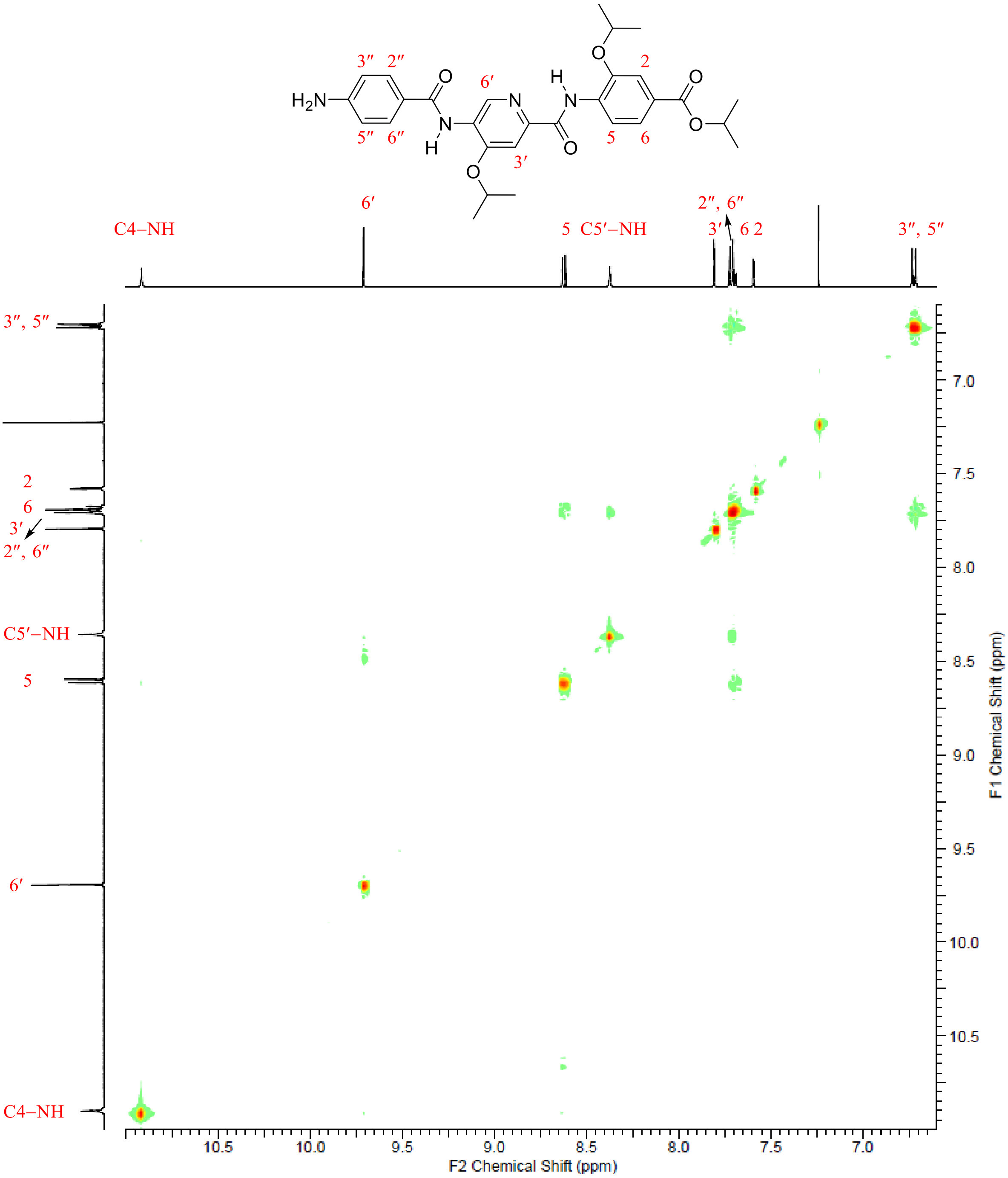

Figure S23. 2D-NOESY spectrum of compound **95** in $CDCl_3$.

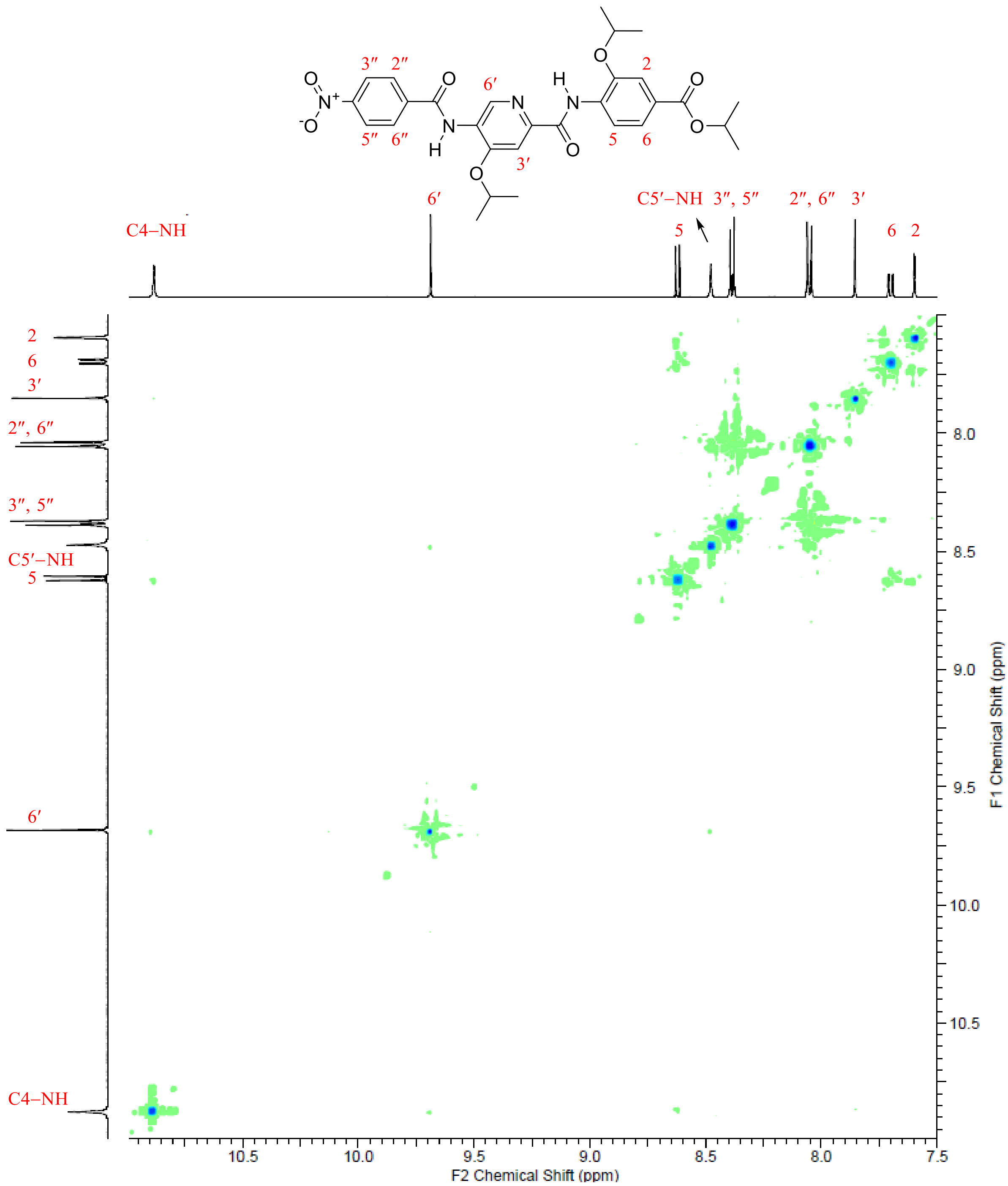

Figure S24. 2D-NOESY spectrum of compound **93** in $CDCl_3$.

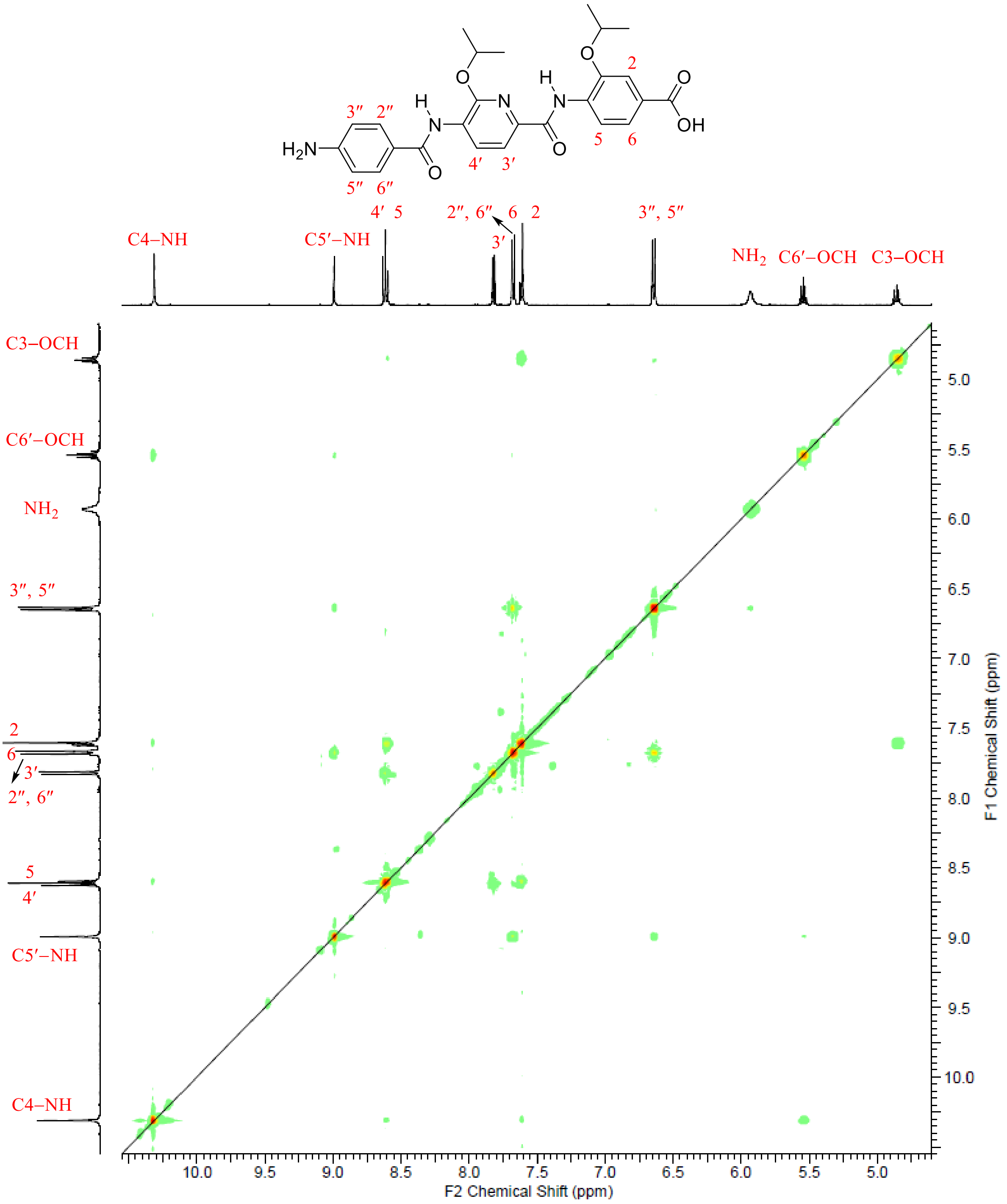

Figure S25. 2D-NOESY spectrum of compound **9** in DMSO-d_6.

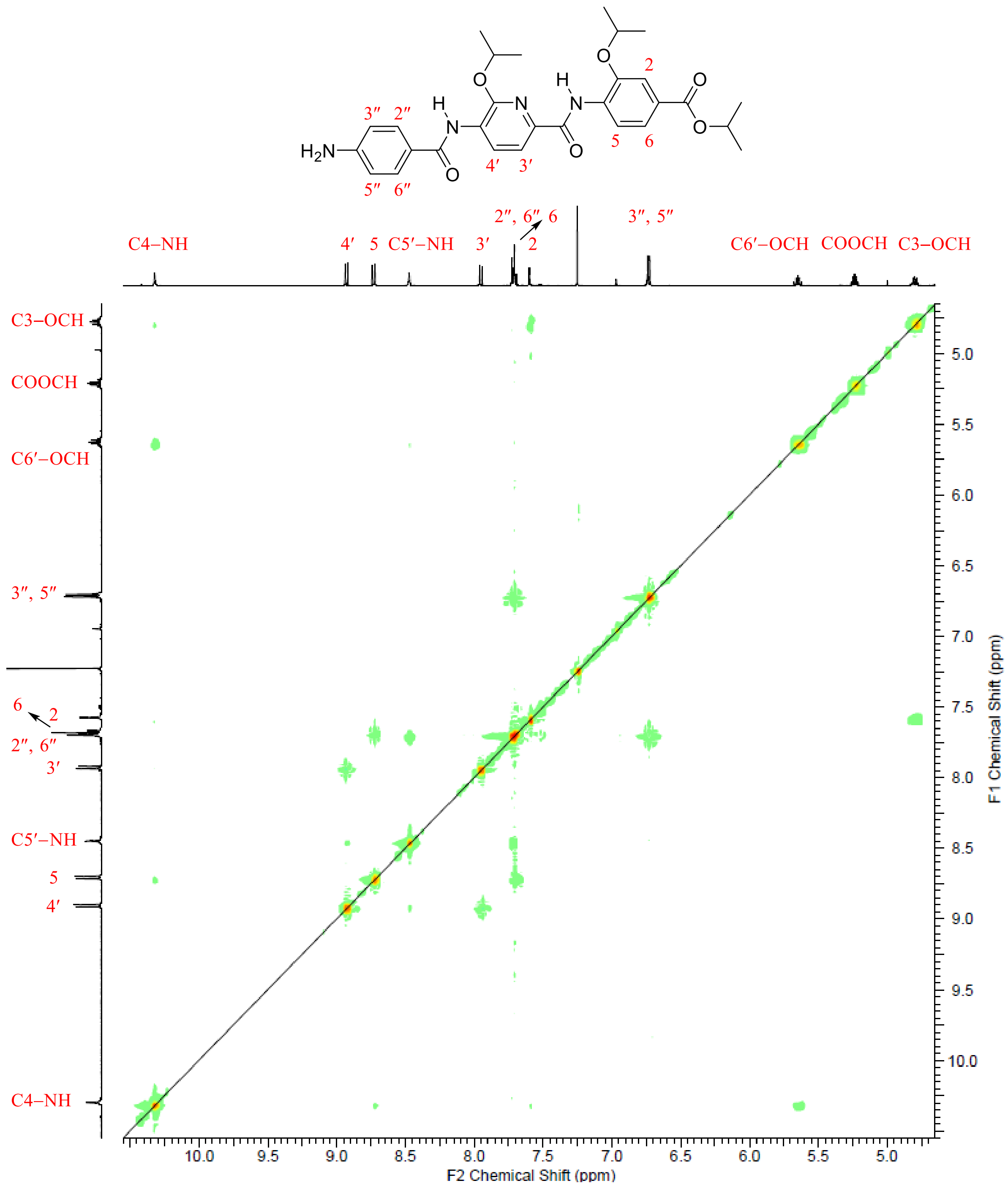

Figure S26. 2D-NOESY spectrum of compound **96** in $CDCl_3$.

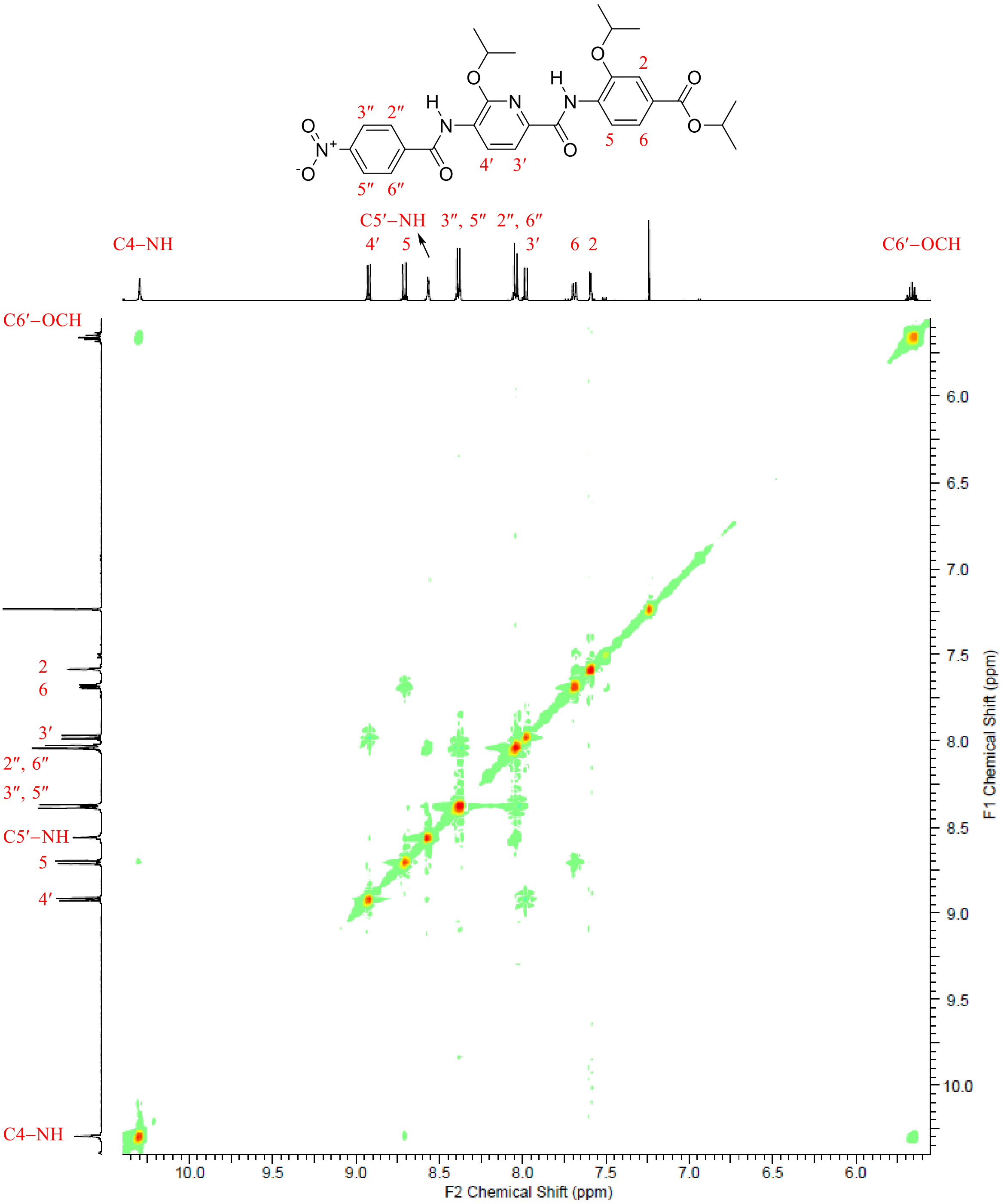

Figure S27. 2D-NOESY spectrum of compound **94** in $CDCl_3$.

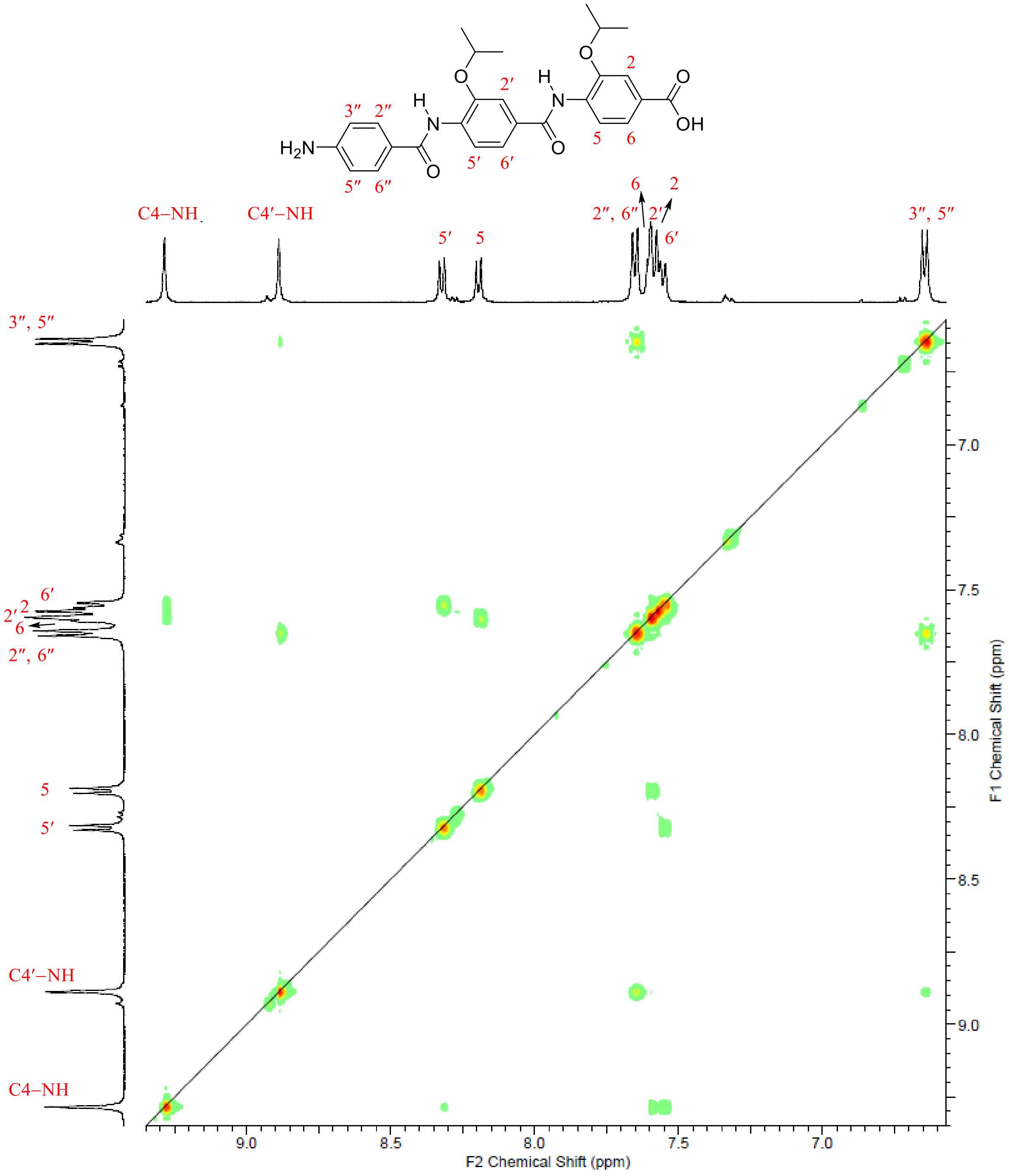

Figure S28. 2D-NOESY spectrum of compound **11** in DMSO-d_6.

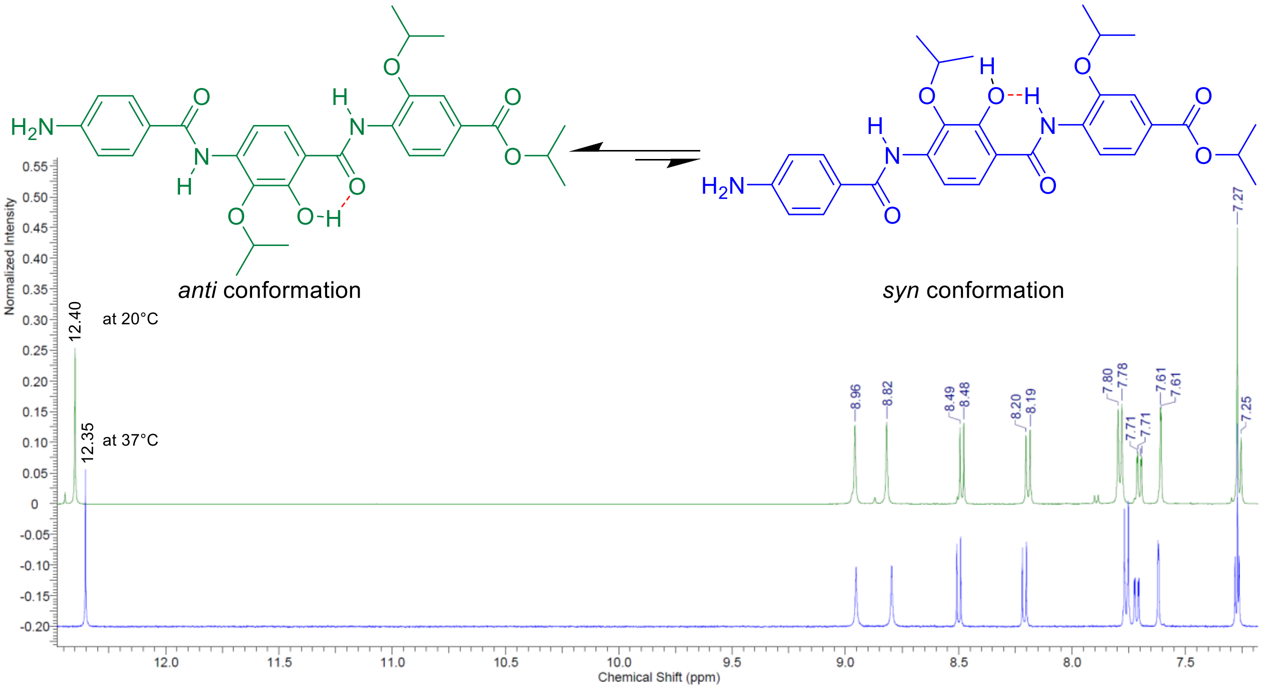

Figure S29. ^{1}H NMR chemical shift of compound **26** at 20 °C (green) and 37 °C (blue) in $CDCl_3$. IMHBs are indicated as red dashed lines.

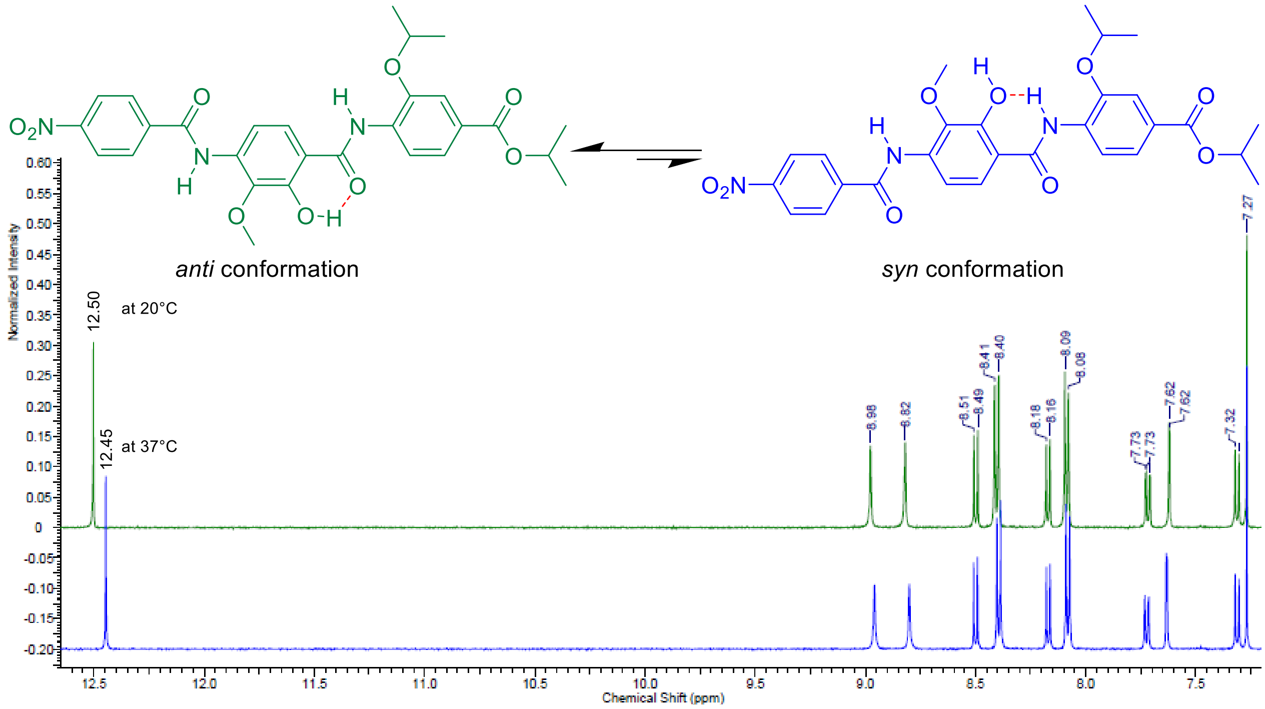

Figure S30. ^{1}H NMR chemical shift of compound **70** at 20 °C (green) and 37 °C (blue) in $CDCl_3$. IMHBs are indicated as red dashed lines.

Computational Chemistry

All computational work was performed using Molecular Operating Environment (MOE) version 2019.01, Chemical Computing Group ULC, 910–1010 Sherbrooke St. W. Montreal, Quebec, H3A 2R7, Canada.

Conformational Analysis A database containing cystobactamid 507 (**2**) and all analogs was created, and each structure was subjected to energy minimization up to a gradient 0.01 kcal/mol/Å using the MMFF94x force field and distance solvation model. Conformational search was performed using low mode MD method,[2] with QM refine option, an energy window of 7.0 kcal/mol and conformation limit of 10000 as conformer filters.

Backbone Curvature Calculation Structures of *anti* or *syn* conformation were loaded separately from the previously prepared conformational database into the MOE window. Angle of inclination of the aryl rings on each other was determined via activating the measure button, choosing angles option, then selecting the carbon atom of the aryl ring bound to the amide nitrogen atom, the carbon atom on the corresponding aryl ring bound to the amide carbonyl group, and the γ carbon atom on the same ring respectively.

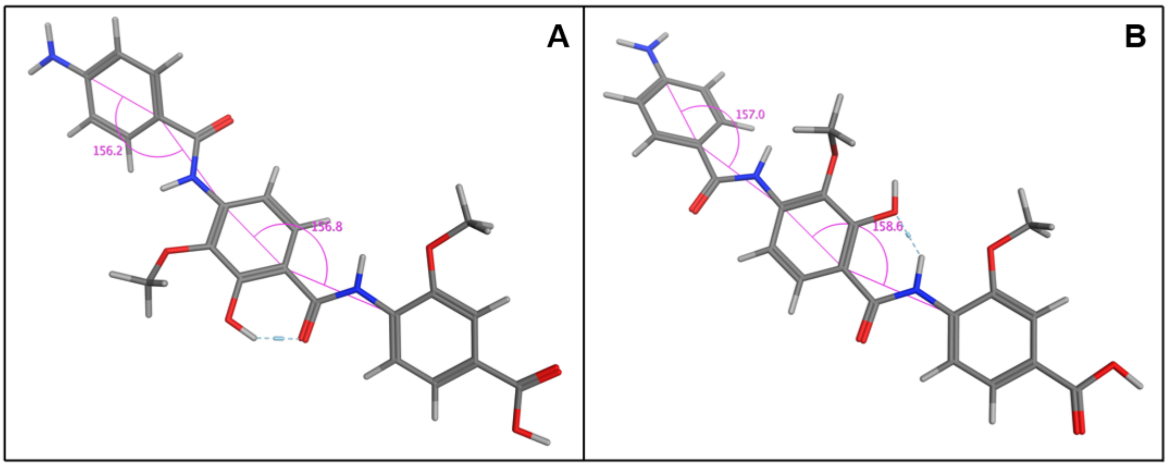

Figure S31. Conformational analysis of **3**: A) *anti*-form (lowest energy conformation); B) *syn*-form (ΔE 0.7 kcal/mol).

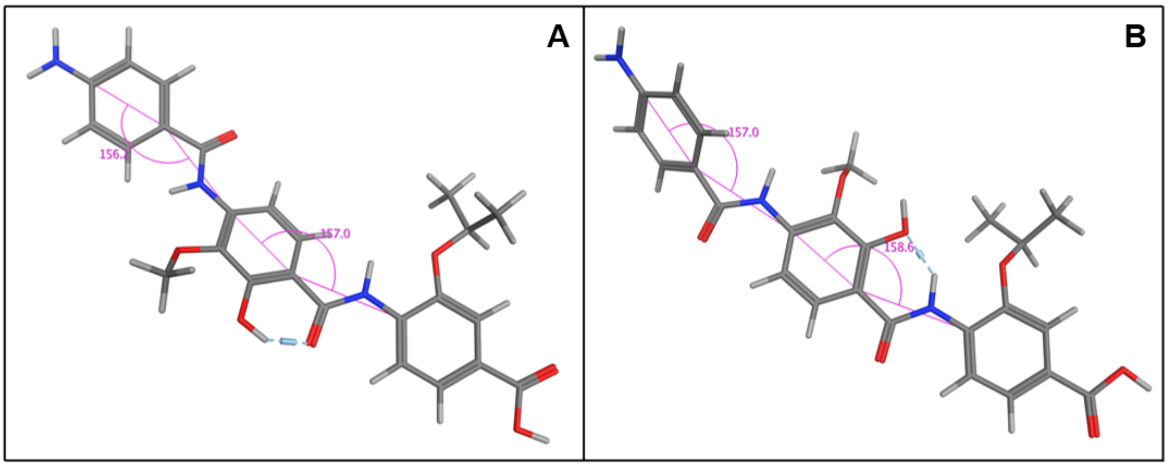

Figure S32. Conformational analysis of **4**: A) *anti*-form (lowest energy conformation); B) *syn*-form (ΔE 0.5 kcal/mol).

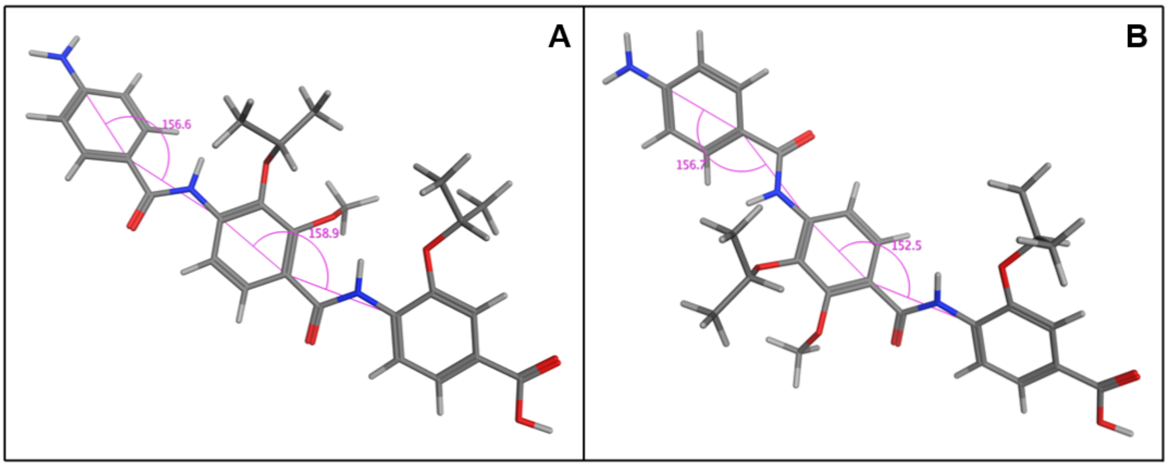

Figure S33. Conformational analysis of **6**: A) *syn*-form (lowest energy conformation); B) *anti*-form (ΔE 3.8 kcal/mol).

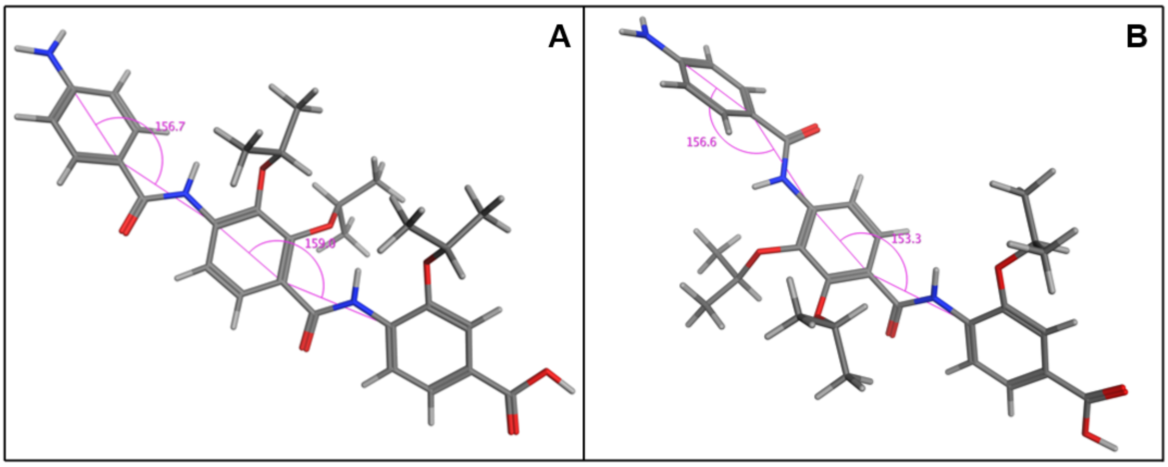

Figure S34. Conformational analysis of **7**: A) *syn*-form (lowest energy conformation); B) *anti*-form (ΔE 4.6 kcal/mol).

Calculation of Electrostatic Surface Structure of cystobactamid 507 (**2**) or **12** was loaded from the previously prepared conformational database into the MOE window. Electrostatic surface was calculated via activating the compute panel, choosing surfaces and maps, then molecular surface option. Atoms were selected as ligand atoms near ligand atoms and color as electrostatics. Electrostatic field was calculated using Gaussian screened Coulomb potential.

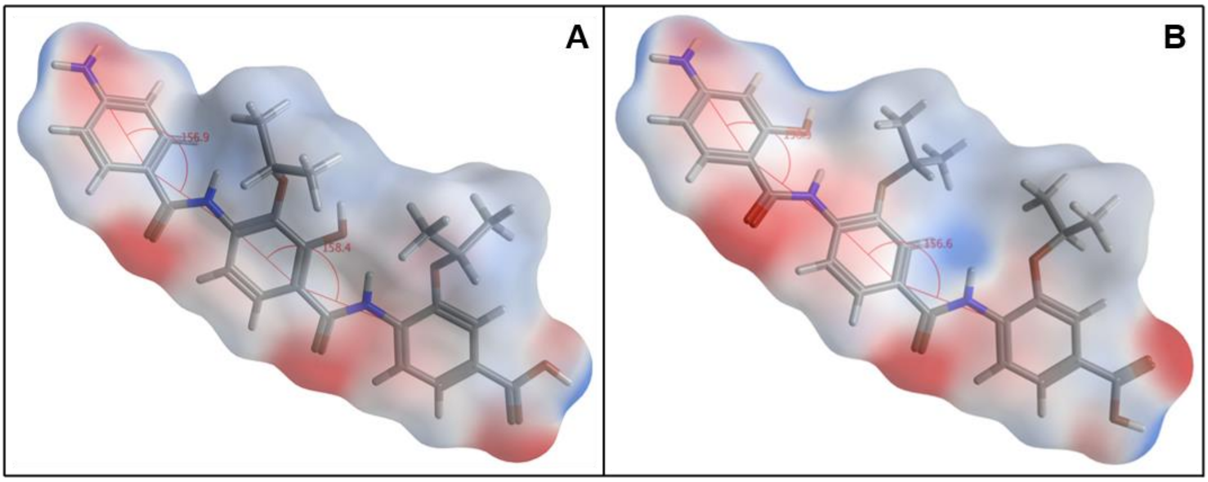

Figure S35. Electrostatic molecular surface and backbone curvature of **2** (A) and **12** (B): (red) negatively charged surface; (blue) positively charged surface; (white) neutral surface.

Calculation of Molecular Descriptors In the database viewer window, molecular descriptors were calculated for all entries via activating the compute panel, choosing descriptors calculate option. Compounds **2**, **12**, and **13** show the same values.

Compound	**2**, **12**, **13**
Total hydrophobic VDW surface area (Q_VSA_HYD)	335.4886
Total polar VDW surface area (Q_VSA_POL)	173.7101
Total positive VDW surface area (Q_VSA_POS)	330.7362
Total negative VDW surface area (Q_VSA_NEG)	178.4624

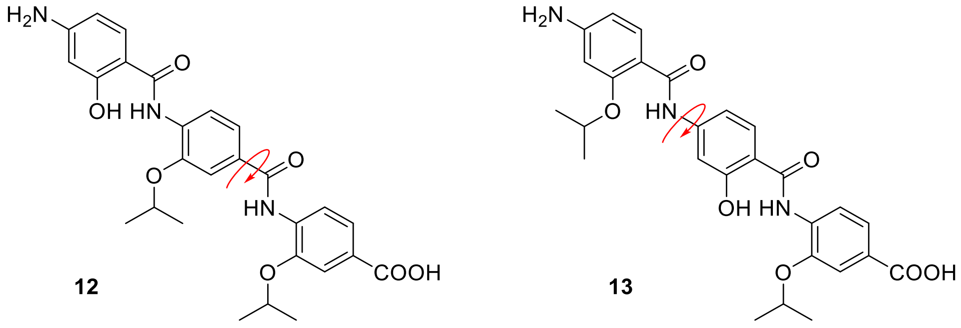

Figure S36. Structures of the freely rotating cystobactamid 507 analogs **12** and **13**.

X-ray Structure Determination

Compounds were dissolved either in $CDCl_3$ (**20**, **91**, **94** and **97**), EtOAc (**44**), THF (**93**) or *n*-hexane:EtOAc, 1:1 (**96**) at room temperature. Crystals were obtained by slow evaporation of solvent. Single crystal X-ray diffraction data were collected at 152 K on a Bruker AXS X8APEX CCD diffractometer operating with graphite-monochromatized Mo Kα radiation. Frames of 0.5° oscillation were exposed. Deriving reflections were in the θ range of 2–29° with a completeness of ~ 99%. Structure solution and full least-squares refinement with anisotropic thermal parameters of all non-hydrogen atoms were performed using SHELX.[3]

Crystallographic data of the compounds:

20: Monoclinic, $P2_1/n$, a = 7.8958(4), b = 6.7919(4), c = 19.8138(11) Å, β = 91.547(3)°.

44: Monoclinic, $P2_1/c$, a = 11.3140(4), b = 8.3739(3), c = 15.1390(6) Å, β = 107.3082(18)°.

91: Monoclinic, $P2_1/n$, a = 8.5870(5), b = 21.3246(11), c = 12.3202(5) Å, β = 102.7567(8)°.

93: Triclinic, P-1, a = 11.1067(15), b = 12.0269(18), c = 12.2433(17) Å, α = 71.838(7), β = 69.391(6), γ = 69.464(7)°.

94: Orthorhombic, $Pca2_1$, a = 23.9033(8), b = 10.6916(3), c = 22.4998(7) Å.

96: Monoclinic, $P2_1/c$, a = 13.4709(10), b = 21.9747(16), c = 9.5297(6) Å, β = 97.940(2)°.

97: Triclinic, P-1, a = 7.5890(3), b = 10.7739(5), c = 11.6933(6) Å, α = 64.668(2), β = 83.738(2), γ = 71.654(2)°.

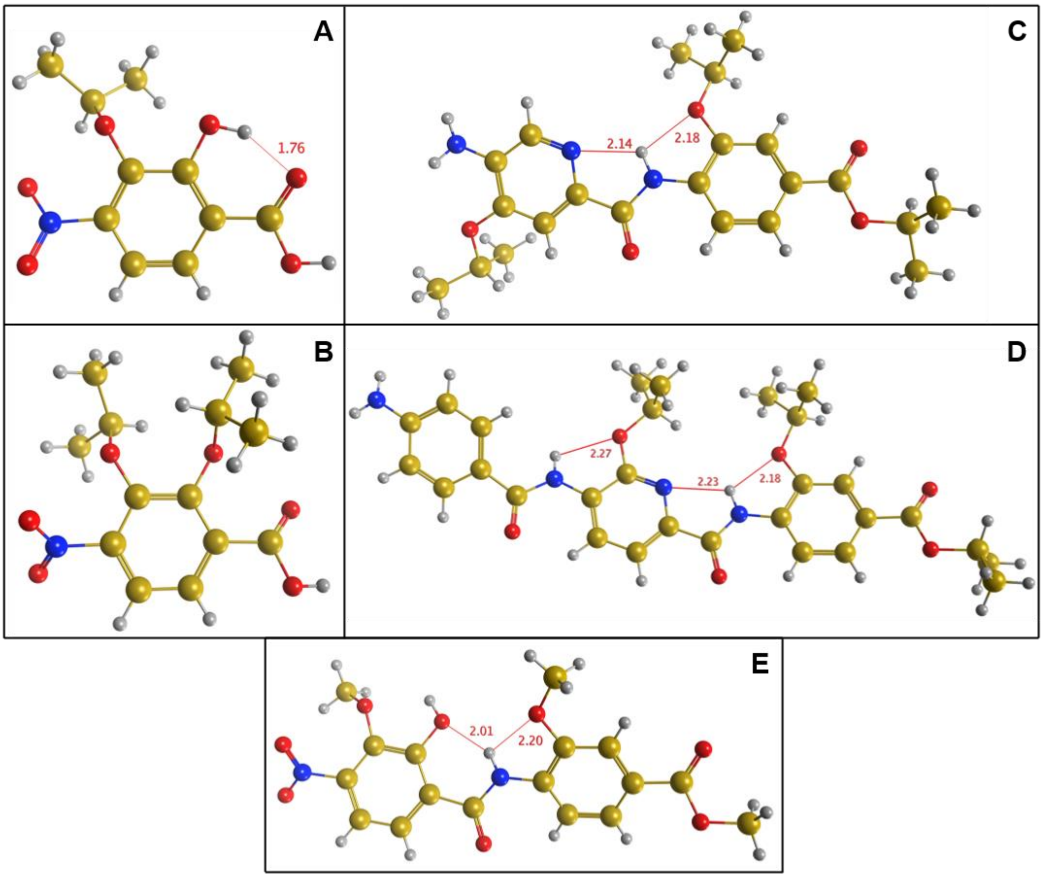

Figure S37. X-ray crystal structures of compounds **20** (A), **44** (B), **91** adopting *anti* conformation via IMHB (C), **96** and **97** adopting *syn* conformation via IMHB (D and E, respectively).

Biology

Cloning, Expression and Purification of *E. coli* GyrA and GyrB

GyrA and GyrB full-length genes were amplified by PCR from *E. coli* genomic DNA (Phusion polymerase, Thermo Scientific) using the following primer pairs (Sigma-Aldrich):

GyrA Forward (*Nde*I): ATCATATGAGCGACCTTGCGAGAGAAATTAC

GyrA Reverse (*Xho*I): ATCTCGAGTTCTTCTTCTGGCTCGTCGTCAACG

GyrB Forward (*Nde*I): ATCATATGTCGAATTCTTATGACTCCTCCAG

GyrB Reverse (*Xho*I): ATCTCGAGAATATCGATATTCGCCGCTTTCAGG

The obtained amplicons were ligated into the pET-28b expression vector system (N-terminal His_6-tag)(Novagen) using an *Nde*I/*Xho*I strategy and transformed into *E. coli* HS996 cells for selection (kanamycin). Positive clones were picked and verified by Sanger sequencing. For protein expression the constructs pET28-GyrA and pET28-GyrB were transformed into *E. coli* BL21 cells. 1–2 L of LB-medium were inoculated 1:10 with fresh overnight cultures and incubated for 1–2 h at 37 °C, 200 rpm. The cultures were then transferred to 16 °C, 200 rpm and the expression was induced after 30 min by the addition of 0.1 mM IPTG. The cells were harvested after 24 h, washed with ice-cold 50 mM NaH_2PO_4/Na_2HPO_4 pH 8.0, 300 mM NaCl buffer and the cell pellet was stored at -80 °C.

For purification, protein crude extracts were prepared by ultrasonification in ice-cold 50 mM NaH_2PO_4/Na_2HPO_4 pH 8.0, 300 mM NaCl, 40 mM imidazole (2 mL/g cell fresh weight). The N-terminally His_6-tagged GyrA and GyrB fusion constructs were then purified using Ni^{2+}-NTA affinity chromatography (ÄKTA FPLC system + 5mL Ni^{2+}-NTA columns, GE Healthcare) followed by size-exclusion chromatography (Superdex 200 increase 10/300 GL, GE Healthcare). The purity of the protein constructs was verified by 15% SDS-PAGE. Standard yields for GyrA and GyrB were in the range of 5–10 mg fusion protein per liter culture. Purified GyrA and GyrB were desalted using PD10 columns and stored in GyrA storage buffer (50 mM Tris-HCl pH 7.5, 100 mM KCl, 1 mM EDTA, 2 mM dithiothreitol, 20% (v/v) glycerol)[3] and GyrB storage buffer (50 mM Tris-HCl pH 7.5, 150 mM NaCl, 1 mM EDTA, 2 mM dithiothreitol, 20% (v/v) glycerol)[3] at -80 °C, respectively. Molar concentrations were determined by UV spectroscopy using the following extinction coefficients: e_{280}(GyrA): 48270 M^{-1} cm^{-1}; e_{280}(GyrB): 68020 M^{-1} cm^{-1}.

Reconstitution of *E. coli* Gyrase

E. coli gyrase tetramers were reconstituted by mixing 5 µM of each subunit. Final concentration of the gyrase stock: 1.25 µM.

Cloning, Expression and Purification of *E. coli* Topoisomerase (TopA)

The TopA full-length gene were amplified by PCR from *E. coli* genomic DNA (Phusion polymerase, Thermo Scientific) using the following primer pairs (Sigma-Aldrich):

TopA Forward (Nde): ATCATATGGGTAAAGCTCTTGTCATCG

TopA Reverse (Xho): ATCTCGAGTTATTTTTTTCCTTCAACCCATTTGC

The obtained amplicons were ligated into the pET-28b expression vector system (N-terminal His_6-tag) (Novagen) using an *Nde*I/*Xho*I strategy and transformed into *E. coli* HS996 cells for selection (kanamycin). Positive clones were picked and verified by Sanger sequencing. For protein expression the constructs pET28-TopA was transformed into *E. coli* BL21 cells. 1–2 L of LB-medium were inoculated 1:10 with fresh overnight cultures and incubated for 1–2 h at 37 °C, 200 rpm. The cultures were then transferred to 16 °C, 200 rpm and the expression was induced after 30 min by the addition of 0.1 mM IPTG. The cells were harvested after 24 h, washed with ice-cold 50 mM NaH_2PO_4/Na_2HPO_4 pH 8.0, 300 mM NaCl buffer and the cell pellet was stored at -80 °C.

For purification, protein crude extracts were prepared by ultrasonification in ice-cold 50 mM NaH_2PO_4/Na_2HPO_4 pH 8.0, 300 mM NaCl, 40 mM imidazole (2 mL/g cell fresh weight). The N-terminally His_6-tagged TopA fusion constructs was then purified using Ni^{2+}-NTA affinity chromatography (ÄKTA FPLC system + 5mL Ni^{2+}-NTA columns, GE Healthcare) followed by size-exclusion chromatography (Superdex 200 increase 10/300 GL, GE Healthcare). The purity of the protein constructs was verified by 15% SDS-PAGE. Standard yields for TopA were in the range of 5–10 mg fusion protein per liter culture. Purified TopA was desalted using PD10 columns and stored in TopA storage buffer (25 mM Tris-HCl pH 7.5, 150 mM NaCl, 1 mM EDTA, 1 mM dithiothreitol, 20% (v/v) glycerol)[3] at -80 °C. Molar concentrations were determined by UV spectroscopy using the following extinction coefficient: e_{280}(TopA): 95700 M^{-1} cm^{-1}.

Preparation of pBR322 Plasmid Substrate

An original batch of pBR322 plasmid was purchased from Inspiralis (Norwich, UK), transformed into *E. coli* HS996 for amplification (selection using ampicillin) and stored at -80 °C as glycerol stock. For plasmid preparation 5 L of LB medium (ampicillin) were inoculated using this strain and grown over night at 37 °C, 220 rpm. The plasmids were isolated using the Qiagen GigaPrep Kit (Qiagen, Hilden, Germany) and stored in MilliQ-H_2O at -20 °C. The plasmid isolated by this strategy is 100% supercoiled and could be used for TopA assays directly.

For the preparation of 100% relaxed plasmid as substrate for gyrase assays, 2 mg/mL plasmid were combined with 1 µM *E. coli* TopA in TopA raction buffer (20 mM Tris-HCl pH 8.0, 50 mM potassium acetate, 10 mM magnesium acetate, 2 mM dithiothreitol and 100 µg/mL (w/v) bovine serum albumin) at 37 °C for 2 h.[4] After phenol-chloroform extraction, the DNA was precipitated using EtOH/sodium acetate method, dissolved in MilliQ-H_2O and stored at -20 °C.

Gyrase Supercoiling Assay

N-terminally His-tagged *E. coli* gyrase was used. For standard reactions 0.5 µg relaxed plasmid were mixed with 1 unit (20.5 nM) gyrase in 1× reaction buffer (35 mM Tris-HCl pH 7.6, 24 mM KCl, 2 mM dithiothreitol, 4 mM $MgCl_2$, 1.8 mM spermidine, 0.1 mg/mL bovine serum albumin, 1 mM ATP, 5% (v/v) glycerol) (30 µL final volume) and incubated for 30 min at 37 °C. The reactions were quenched by the addition of DNA gel loading buffer containing 1% (w/v) SDS. The samples were separated on 0.8% (w/v) agarose gels and DNA was visualized using ethidium bromide. All NPs and compounds

stock solutions and dilutions were prepared in DMSO and added to the supercoiling reactions giving a final DMSO concentration of 5% (v/v). Control reactions were: no enzyme and a standard reaction in presence of 5% (v/v) DMSO. All reaction samples were equilibrated for 15 min at room temperature in the absence of DNA. Then the relaxed plasmid was added to start the reaction. All reactions were performed in triplicates

Topoisomerase IV Relaxation Assay

Commercial *E. coli* topoisomerase IV relaxing kits (Inspiralis, Norwich, UK) were used. For standard reactions 0.5 µg supercoiled plasmid were mixed with 1 unit (~20.5 nM) topoisomerase IV in 1× reaction buffer (see kit manual) and incubated for 30 min at 37 °C. The reactions were quenched by the addition of DNA gel loading buffer containing 1% (w/v) SDS. The samples were separated on 0.8% (w/v) agarose gels and DNA was visualized using ethidium bromide. Control reactions were: no enzyme and a standard reaction in presence of 5% (v/v) DMSO. All reaction samples were equilibrated for 15 min at room temperature in the absence of DNA. Then the relaxed plasmid was added to start the reaction. All reactions were performed in triplicates.

Quantification and Analysis

To determine IC_{50} values, agarose gels were digitalized using standard gel documentation instruments and supercoiled (gyrase) and relaxed (topoisomerase IV) plasmid was quantified using Adobe Photoshop (Histogram mode). Intensities were normalized (% enzyme activity = SC / (SC + relaxed)). Plotting of these values versus the compound concentration yielded sigmoidal shaped curves, which were fitted using Hill's equation (Origin Pro 8.5).[5] All determined IC_{50} values are the averages of three independent experiments.

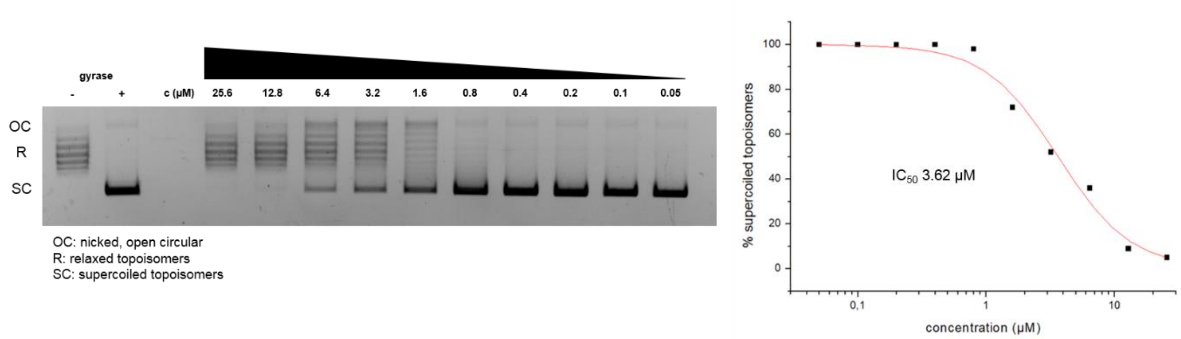

Figure S38. *E. coli* gyrase supercoiling assay of compound **16**.

Table S2. *In vitro* inhibitory activities of esterified cystobactamid 507 analogs and their parent compounds in the gyrase supercoiling assay.

Free acid	IC_{50} *E. coli* gyrase (µM)	Corresponding ester	IC_{50} *E. coli* gyrase (µM)
**4**	360 ± 26	**78**	> 500
**6**	115 ± 18	**79**	> 500
**7**	60 ± 10	**80**	> 500
**9**	50 ± 10	**96**	> 500
**11**	165 ± 18	**82**	> 500
**12**	85 ± 12	**83**	473 ± 20
**13**	101 ± 15	**84**	180 ± 38

DNA Competition Assay Using Hoechst 33342 and Ethidium Bromide (EtBr)

EtBr and Hoechst 33342 competitive binding assays were performed[6] by recording the emission spectra of solutions (30 µL) containing varying concentrations of cystobactamid derivatives (in DMSO; 500–0.1 µM and 5% DMSO final), 15 µM calf thymus DNA (Sigma-Aldrich) and 15 µM of EtBr or Hoechst 33342 in 25 mM sodium phosphate buffer (pH 7.5), 150 mM NaCl.

All measurements (triplicates) were performed in 384 well plates (black, low volume) (Corning, Corning, NY, USA) using a Tecan infinite II reader (Tecan, Switzerland) using the following (standard) parameters: Bandwidth 20 nm, 10 flashes, integration time 20 µs, no delay, no pause, Z: 20000

Hoechst 33342: λ_{ex}: 355 nm, λ_{em}: 370–850 nm in 20 nm steps

Ethidium bromide: λ_{ex}: 480 nm, λ_{em}: 490–850 nm in 20 nm steps

All samples were mixed and incubated at room temperature for 30 min before each measurement.

Quantification and Analysis

To determine apparent values for the compounds' "minor groove affinities" (50% displacement of Hoechst 33342), the values of the Hoechst 33342 fluorescence spectra peak maxima were plotted vs. compound concentration (in µM) and fitted using Hill's equation (Origin Pro 8.5).[5] All determined values are the averages of three independent experiments.

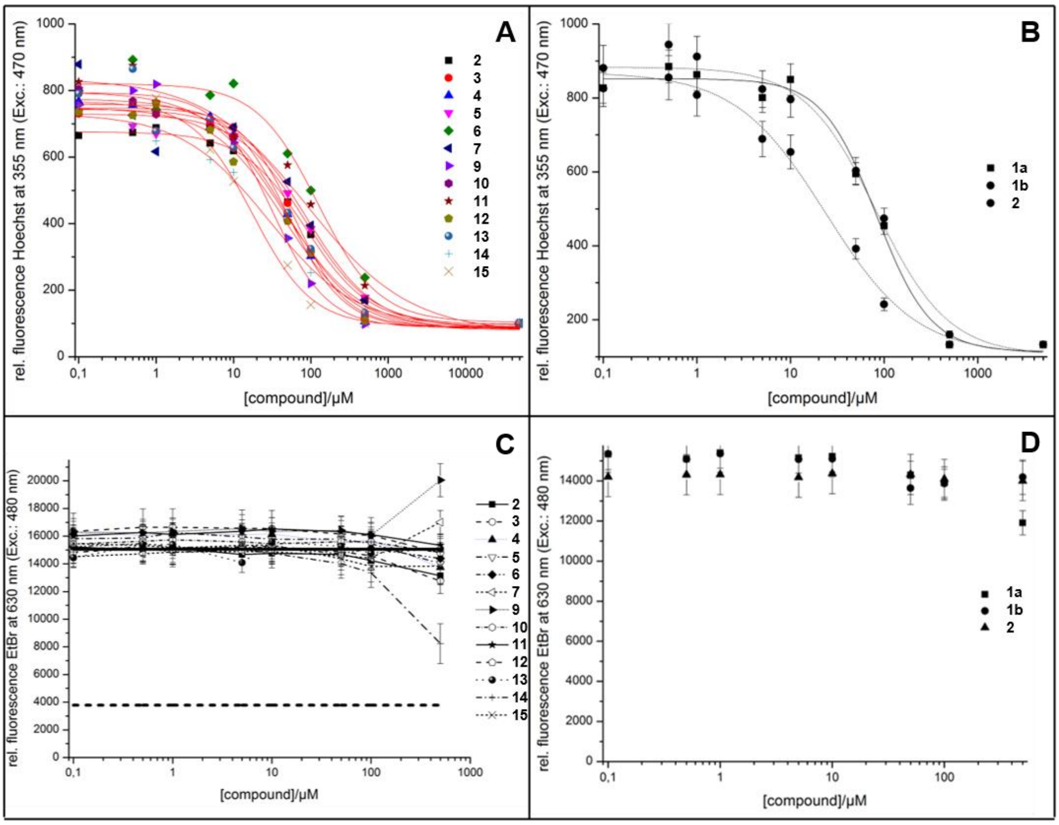

Figure S39. Competition titration of ct-DNA (15 µM) bound Hoechst 33342 (15 µM) (A, B) and EtBr (15 µM) (C, D) with cystobactamid 507 analogs (A, C) and natural cystobactamids (B, D). The relative fluorescence intensity at the peak maximum (470 nm for Hoechst 33342 and 630 nm for EtBr, respectively) is plotted vs the respective cystobactamid 507 analog concentration. The solid- and dashed line represent the fluorescence intensity of the ct-DNA bound and DNA-free dyes, respectively.

Plotting the maxima of the Hoechst 33342 spectra vs compounds' concentrations delivered sigmoidal shaped curves, which could be fitted using Hill's equation (Fig. S39). This allowed the determination of a value for "50% displacement of Hoechst 33342". Although this value does not contain absolute information about DNA affinity and the number of binding sites, it allows a "face-to-face" comparison of the apparent "minor groove affinity" of the different compounds (Table S3). Remarkably, these values do not significantly correlate with the gyrase activity of the respective compounds (Fig. S40). This indicates that DNA interaction by minor groove binding alone is only of secondary importance for the specificity of the cystobactamids/analogs–target interaction. The main fraction of activity-conferring interactions (the ligand–target specificity) could thus be interactions of the inhibitor with a specific conformation or state of DNA, the single or complexed proteins (GyrA and GyrB) or the whole DNA–protein complex. This is also underlined by the fact that all tested cystobactamids have a preference for gyrase over topoisomerase IV (Table 1), which is in accordance to literature for **1b**.[5]

Table S3. Apparent "minor groove affinities" (50% displacement of Hoechst 33342) and IC_{50} values (gyrase supercoiling) for **1a**, **1b**, and **2–15**

Compound	50% Displacement of Hoechst 33342 (μM)	IC_{50} *E. coli* gyrase (μM)
**1a**	83 ± 10	21 ± 6
**1b**	24 ± 4	0.26 ± 0.06
**2**	85 ± 18	355 ± 25
**3**	61 ± 5	463 ± 28
**4**	51 ±3	360 ± 26
**5**	83 ± 21	>1000
**6**	123 ± 33	115 ± 18
**7**	89 ± 4	60 ± 10
**8**	n.d.	195 ± 20
**9**	31 ± 5	50 ± 10
**10**	49 ± 7	>1000
**11**	89 ± 34	165 ± 18
**12**	45 ± 8	85 ± 12
**13**	44 ± 16	101 ± 15
**14**	29 ± 6	110 ± 20
**15**	18 ± 3	106 ± 18

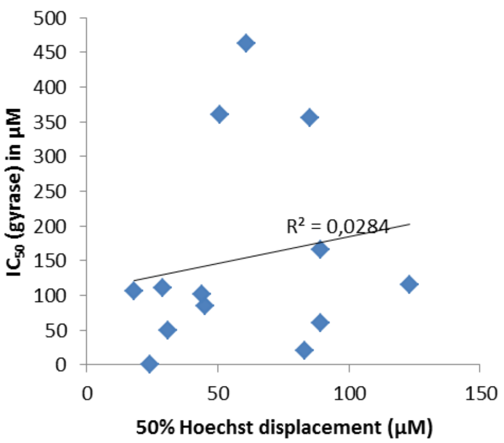

Figure S40. Scatterplot of apparent "minor groove affinities" (50% displacement of Hoechst 33342) vs the IC_{50} values (gyrase supercoiling) for **1a**, **1b**, and **2–15**.

Minimal Inhibitory Concentration (MIC) Determination

MIC values were determined as described elsewhere.[5] Bacterial cultures were handled according to standard procedures and were obtained from the German Collection of Microorganisms and Cell Cultures (*Deutsche Sammlung von Mikroorganismen und Zellkulturen*, DSMZ), the American Type Culture Collection (ATCC) or were part of our internal strain collection. In brief, bacteria in mid-log phase were diluted to achieve a final inoculum of ca. 5×10^5–5×10^6 cfu/mL in Tryptic Soy broth (1.7% peptone casein, 0.3% peptone soymeal, 0.25% glucose, 0.5% NaCl, 0.25% K_2HPO_4; pH 7.3; *E. faecalis*, *S. pneumoniae*), or Mueller-Hinton broth (1.75% casein hydrolysate, 0.2% beef infusion, 0.15% starch; pH 7.4; used for all other listed bacteria). *E. faecalis* and *S. pneumoniae* cultures were grown under microaerophilic conditions without shaking and all other listed microorganisms were grown on a shaker (200 rpm) at 37 °C. *E. coli* DSM-26863 was grown with or without PMBN (polymyxin B nonapeptide) at sublethal concentration (3 µg/mL) for permeabilization. Serial dilutions of the compounds were prepared from DMSO stocks in sterile 96-well plates. The cell suspension was added and microorganisms were grown for 16–20 h. Growth inhibition was assessed by visual inspection and given MIC values determined in two independent experiments are the lowest concentration of antibiotic at which no visible growth was observed.

AlbD Cleavage Assay

The AlbD cleavage assay was carried out in a total volume of 100 µL. The enzyme AlbD (final concentration 24 µM) and the respective amount of compound to get a final concentration of 12 µM in 0.2 M phosphate buffer (pH 7.0) were incubated at 28 °C for 3 h. For each compound, a negative control was performed in parallel without enzyme to prove compound stability at 28 °C during the incubation time. Furthermore, a positive control with albicidin, which is known to be cleaved by AlbD, was used as confirmation for the enzyme activity. After incubation, the proteins were participated by adding MeOH (350 µL) and centrifugation at 20,000 g for 20 min. The supernatant (250 µL) was evaporated

in a concentrator (Eppendorf centrifugal vacuum concentrator) at 30 °C for 2 h. After dissolving the dried supernatant in MeOH (100 µL), the samples were measured via LC-MS (Bruker Daltonics maXis HD QTof).

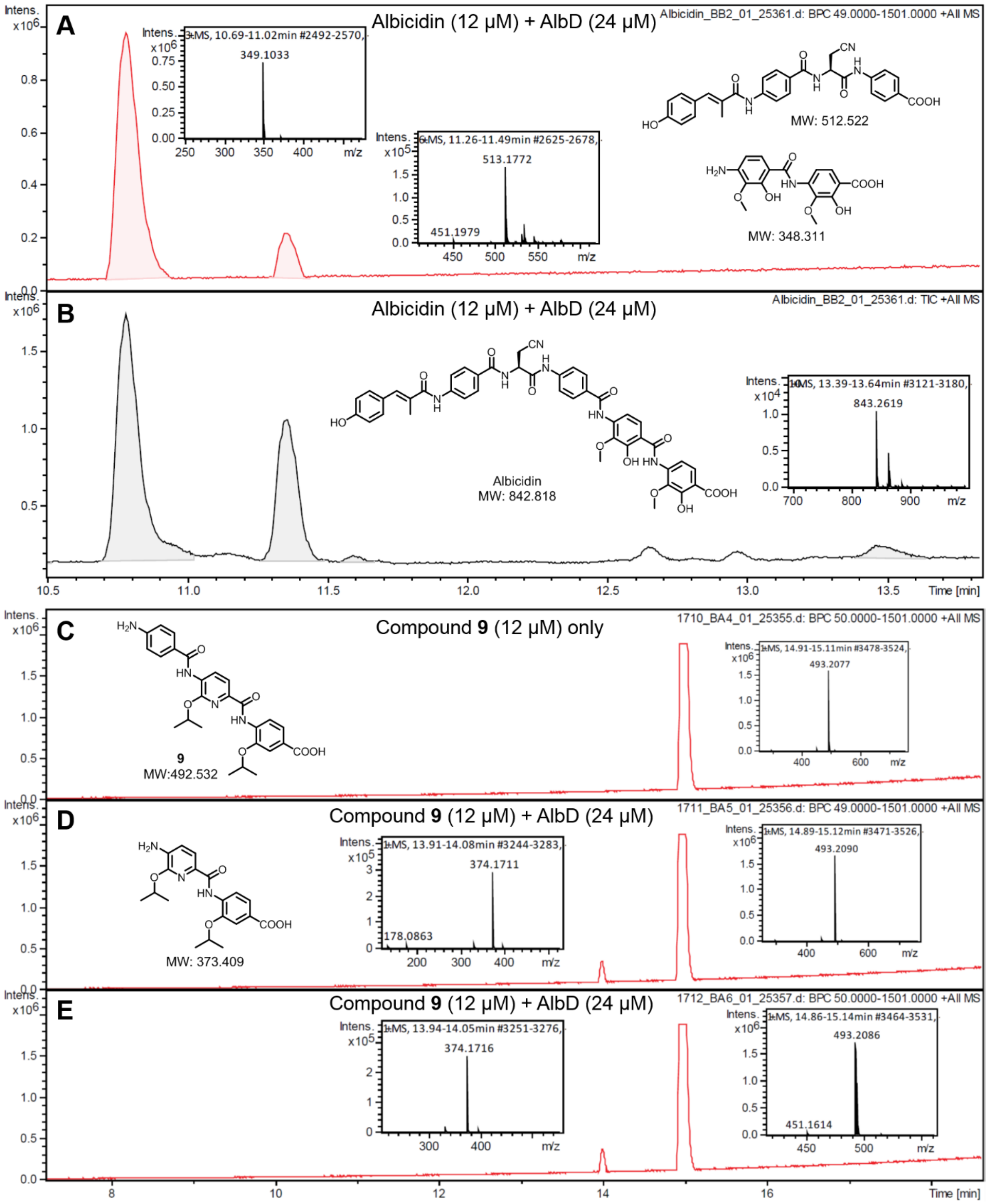

Figure S41. Stability of **9** against AlbD-mediated hydrolysis: (A and B) HPLC-MS analysis of albicidin (12 µM) incubated with AlbD (24 µM) at 28 °C for 3 h (in duplicate) showing almost total cleavage; (C) HPLC-MS analysis of compound **9** (12 µM) incubated in phosphate buffer at 28 °C for 3 h as a negative control; (D and E) HPLC-MS analysis of **9** (12 µM) incubated with AlbD (24 µM) for 3 h at 28 °C showing only traces of the cleavage product (in duplicate).

Metabolic Stability Assay

Metabolic stability of compounds **2**, **7**, **9**, and **12** was determined by incubation of 1 µM compound with 1 mg/mL pooled mammalian liver S9 fraction (BD Gentest), 2 mM NADPH regenerating system, 1 mM UDPGA, 0.1 mM PAPS and 10 mM magnesium chloride in 200 mM potassium hydrogen phosphate buffer (pH 7.4) at 37 °C for 0, 5, 15 and 60 min. At the given time points, two volumes of acetonitrile containing internal standard were added to stop the incubation. Concentration of the remaining test compound was determined using LC-MS/MS and used to determine the half-life ($t_{1/2}$). MS/MS measurements were performed on a TSQ Quantum Acess Max (ThermoFisher, Dreieich, Germany) coupled to an Acella UHPLC system. An electrospray interface (ESI) was used as an ion source. The Acella-LC-system consisted of a pump and an auto sampler. The system was operated by the standard software Xcalibur.

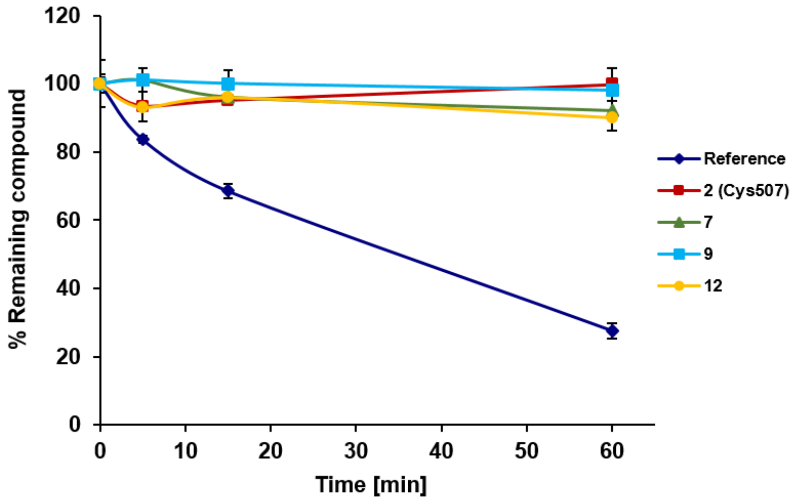

Compound	$t_{1/2}$ (min)
**2**	> 1000
**7**	471
**9**	1703
**12**	562
Reference	33

Figure S42. Percent of remaining cystobactamid 507 (**2**) and the analogs after incubation with human liver S9 fraction for 60 min revealing high stability (left) and their predicted $t_{1/2}$ values (right).

References

1. Moreno, M.; Elgaher, W. A. M.; Herrmann, J.; Schläger, N.; Hamed, M. M.; Baumann, S.; Müller, R.; Hartmann, R. W.; Kirschning, A. *Synlett* **2015**, *26*, 1175–1178.
2. Chen, I.-J.; Foloppe, N. *Bioorg. Med. Chem.* **2013**, *21*, 7898–7920.
3. Sheldrick, G. M. *Acta Cryst.* **2008**, *A64*, 112–122.
4. Shapiro, A.; Jahic, H.; Prasad, S.; Ehmann, D.; Thresher, J.; Gao, N.; Hajec, L. *J. Biomol. Screen.* **2010**, *15*, 1088–1098.
5. Baumann, S.; Herrmann, J.; Raju, R.; Steinmetz, H.; Mohr, K. I.; Huttel, S.; Harmrolfs, K.; Stadler, M.; Müller, R. *Angew. Chem. Int. Ed.* **2014**, *53*, 14605–14609.
6. Kunwar, A.; Simon, E.; Singh, U.; Chittela, R. K.; Sharma, D.; Sandur, S. K.; Priyadarsini, I. K. *Chem. Biol. Drug Des.* **2011**, *77*, 281–287.

^{1}H- and ^{13}C-NMR Spectra of the Described Compounds

Compound **17**

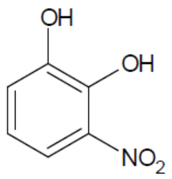

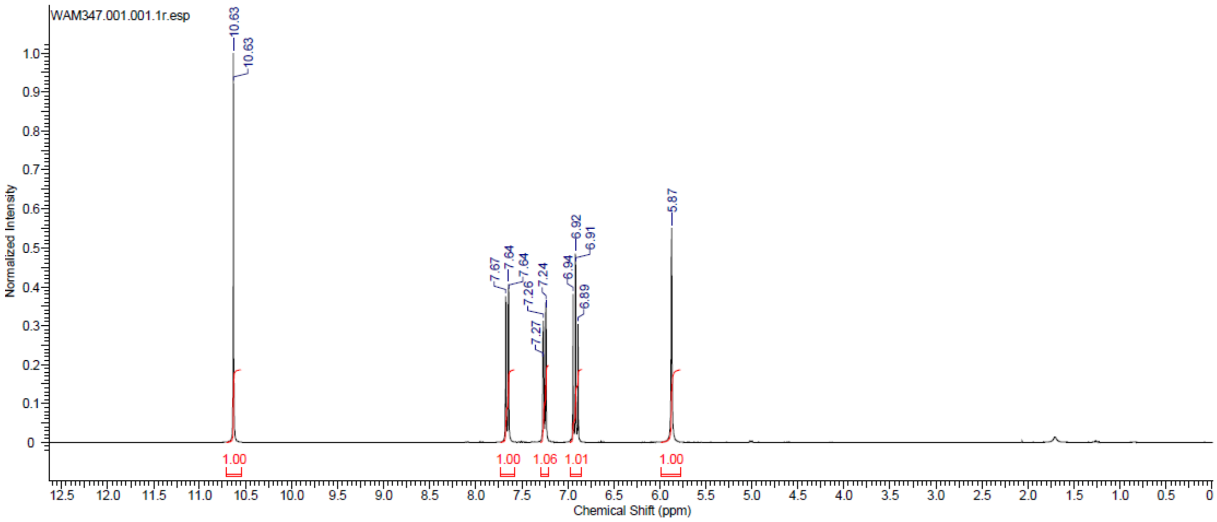

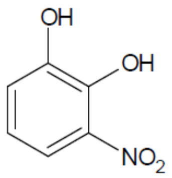

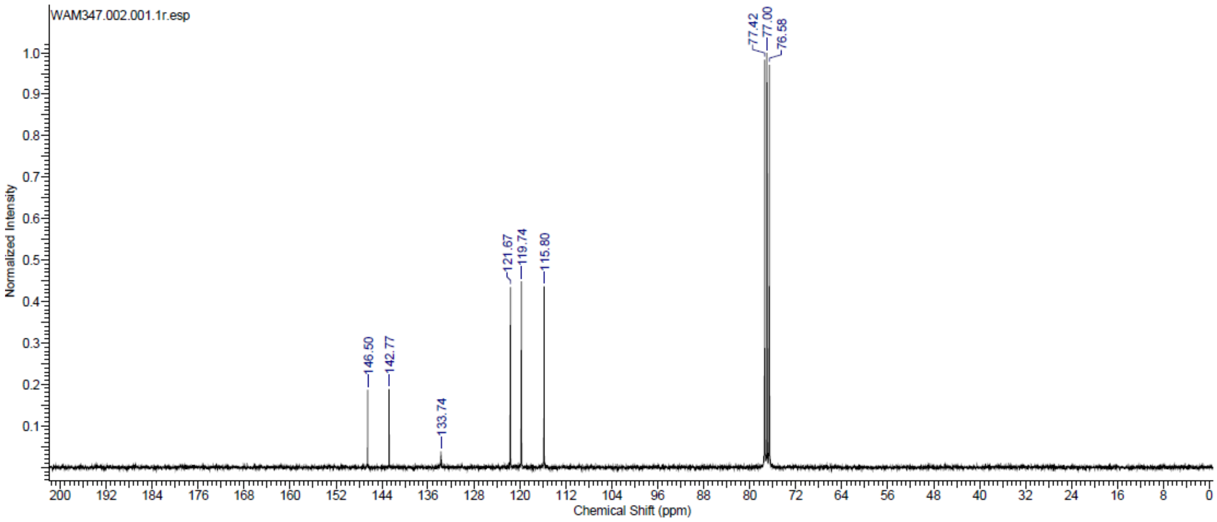

Compound **18**

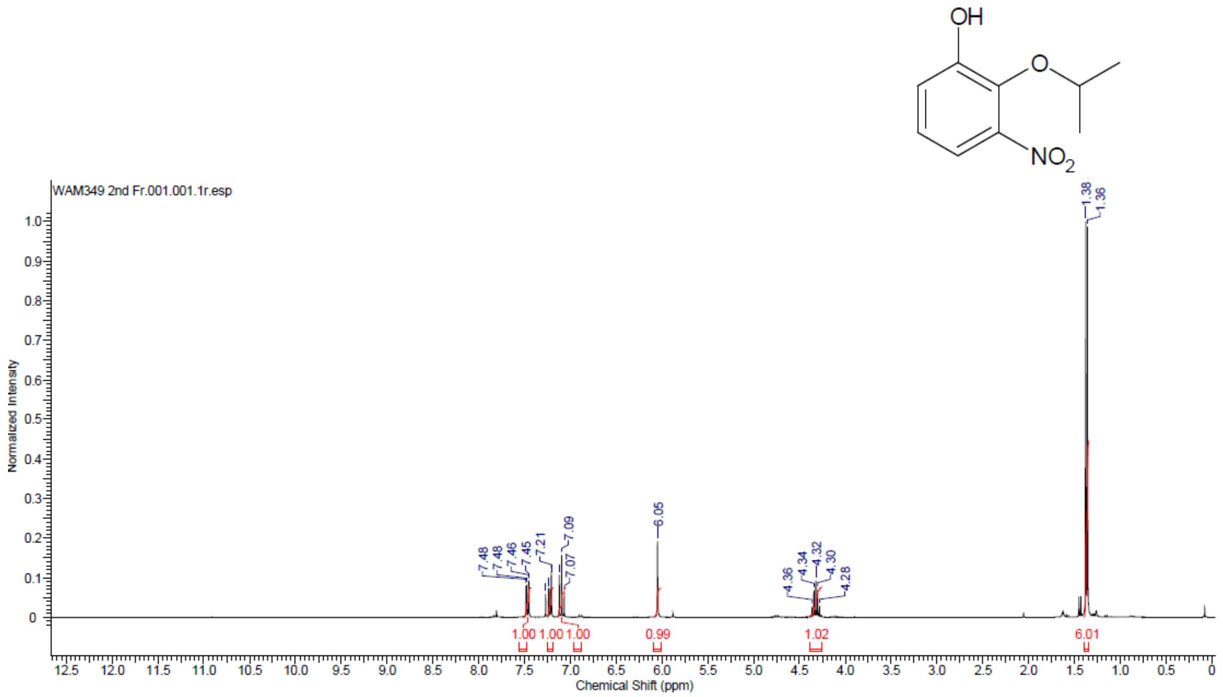
OH
O
NO2
WAM349 2nd Fr.001.001.1r.esp
Normalized Intensity
Chemical Shift (ppm)
7.48
7.48
7.46
7.45
7.21
7.09
7.07
6.05
4.36
4.34
4.32
4.30
4.28
1.38
1.36
1.00
1.00
1.00
0.99
1.02
6.01

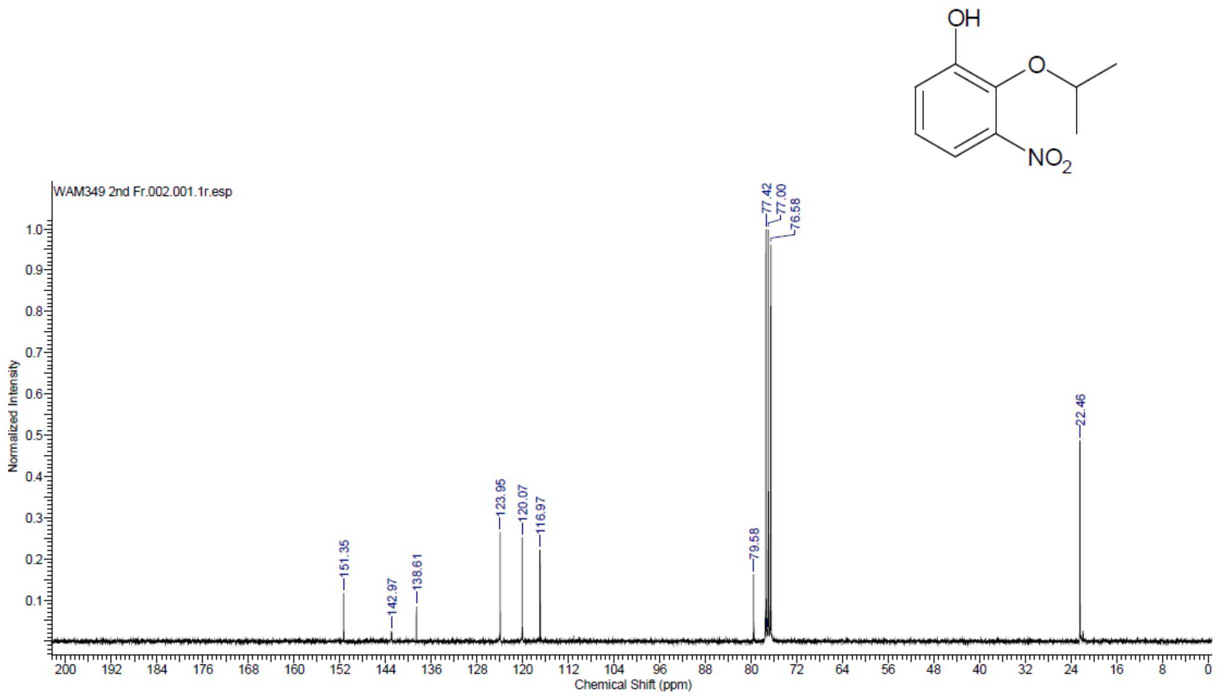
OH
O
NO2
WAM349 2nd Fr.002.001.1r.esp
Normalized Intensity
Chemical Shift (ppm)
151.35
142.97
138.61
123.95
120.07
116.97
79.58
77.42
77.00
76.58
22.46

Compound **19**

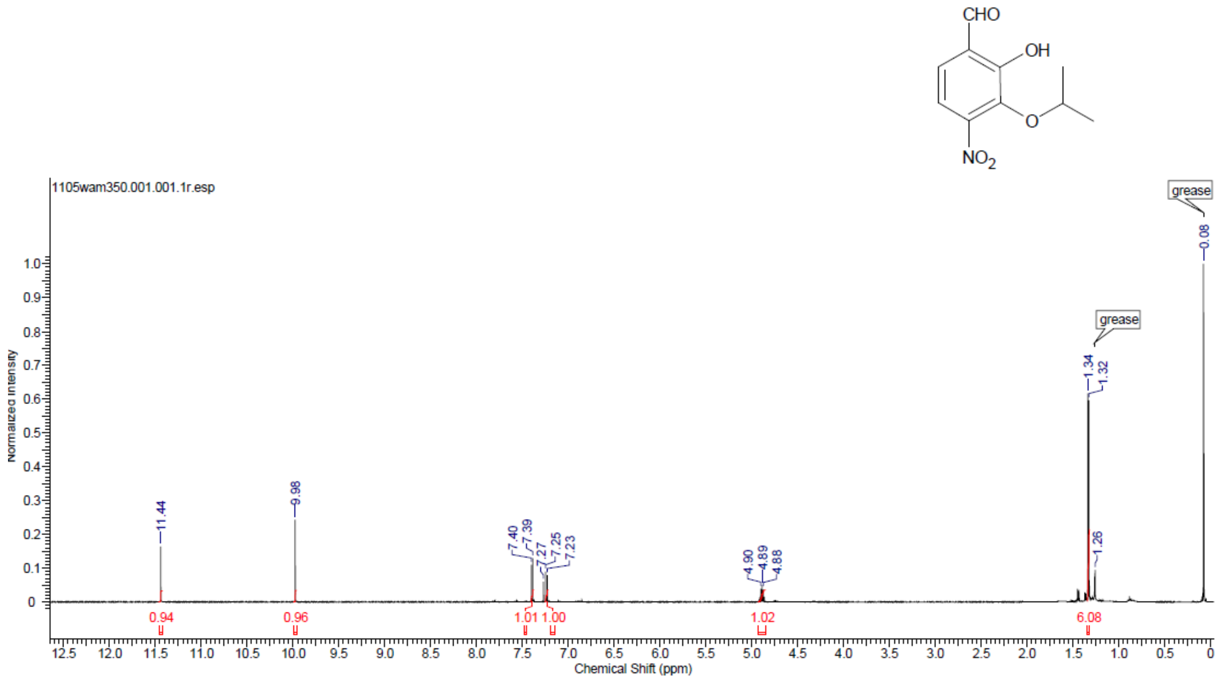

CHO
OH
O
NO_2
1105wam350.001.001.1r.esp
grease
0.08
grease
1.34
1.32
1.26
11.44
9.98
7.40
7.39
7.27
7.25
7.23
4.90
4.89
4.88
0.94
0.96
1.01
1.00
1.02
6.08
Normalized Intensity
Chemical Shift (ppm)

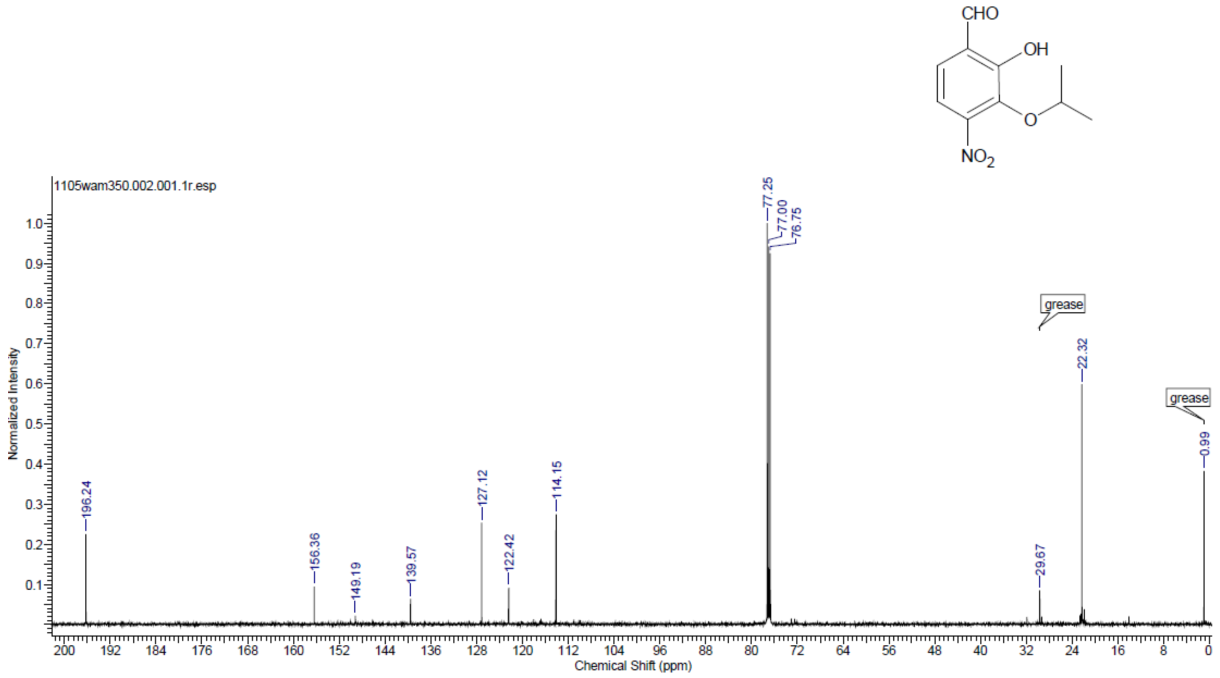

CHO
OH
O
NO_2
1105wam350.002.001.1r.esp
77.25
77.00
76.75
grease
22.32
grease
0.99
196.24
156.36
149.19
139.57
127.12
122.42
114.15
29.67
Normalized Intensity
Chemical Shift (ppm)

Compound **20**

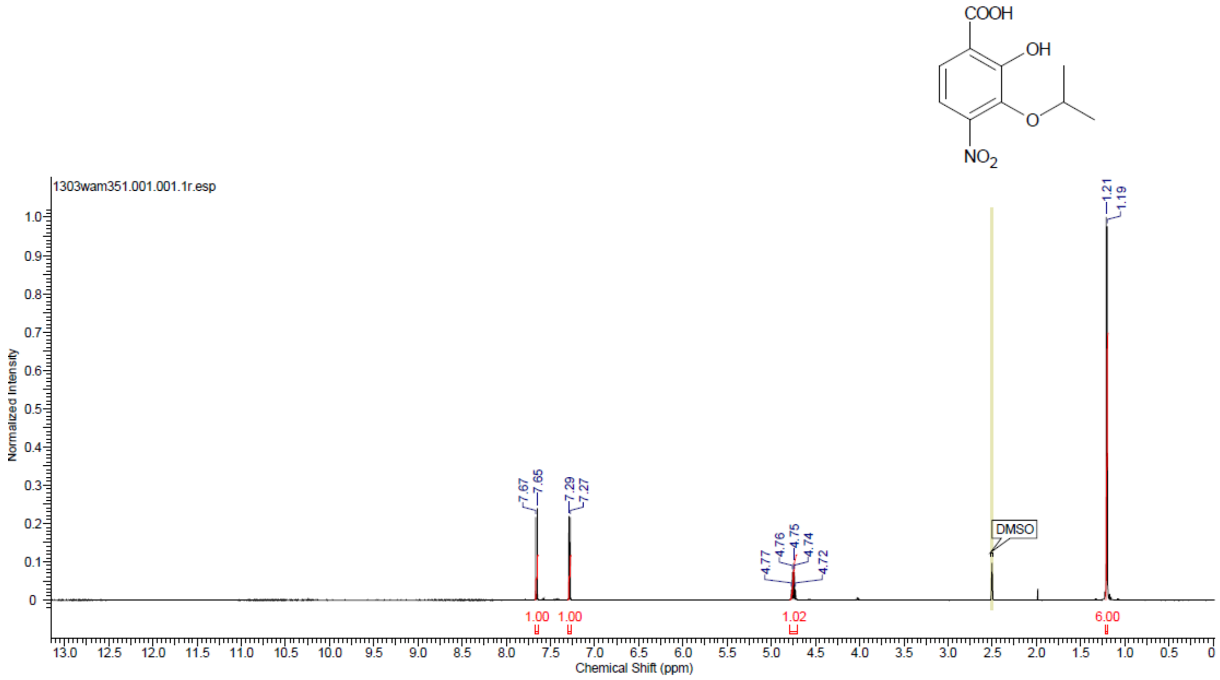

COOH
OH
O
NO2
1303wam351.001.001.1r.esp
Normalized Intensity
7.67
7.65
7.29
7.27
4.77
4.76
4.75
4.74
4.72
DMSO
1.21
1.19
1.00
1.00
1.02
6.00
Chemical Shift (ppm)

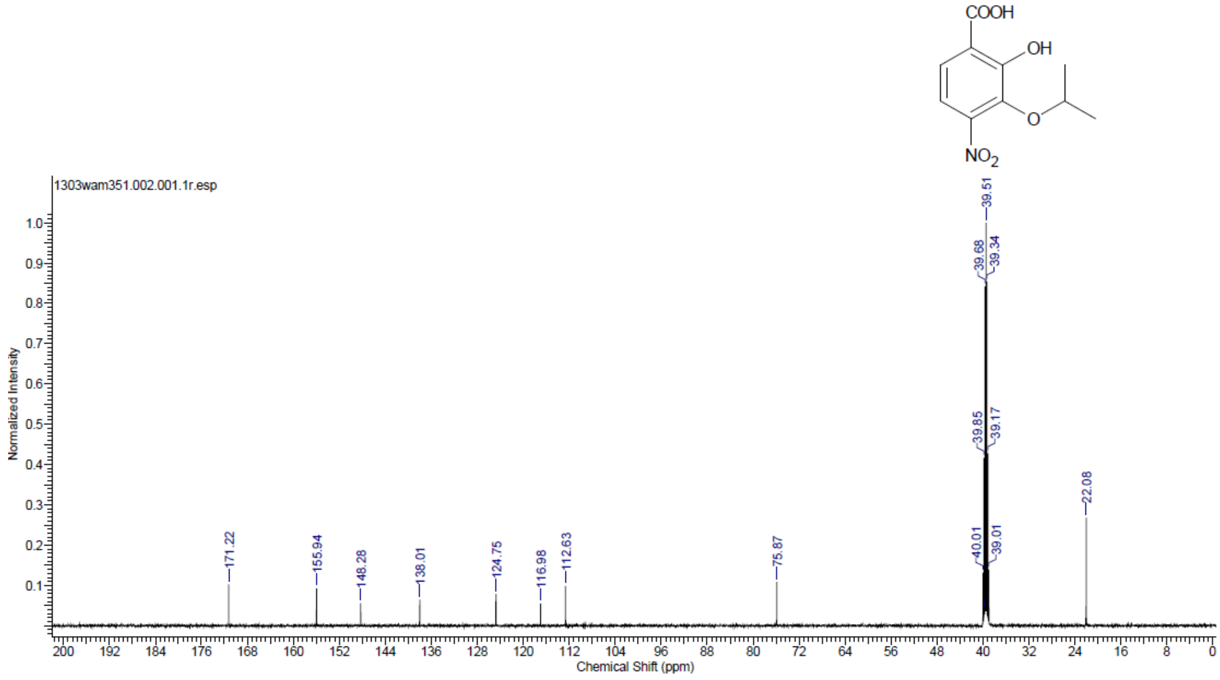

COOH
OH
O
NO2
1303wam351.002.001.1r.esp
Normalized Intensity
171.22
155.94
148.28
138.01
124.75
116.98
112.63
75.87
40.01
39.85
39.68
39.51
39.34
39.17
39.01
22.08
Chemical Shift (ppm)

Compound **42**

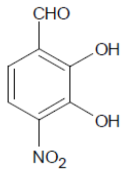
CHO
OH
OH
NO2

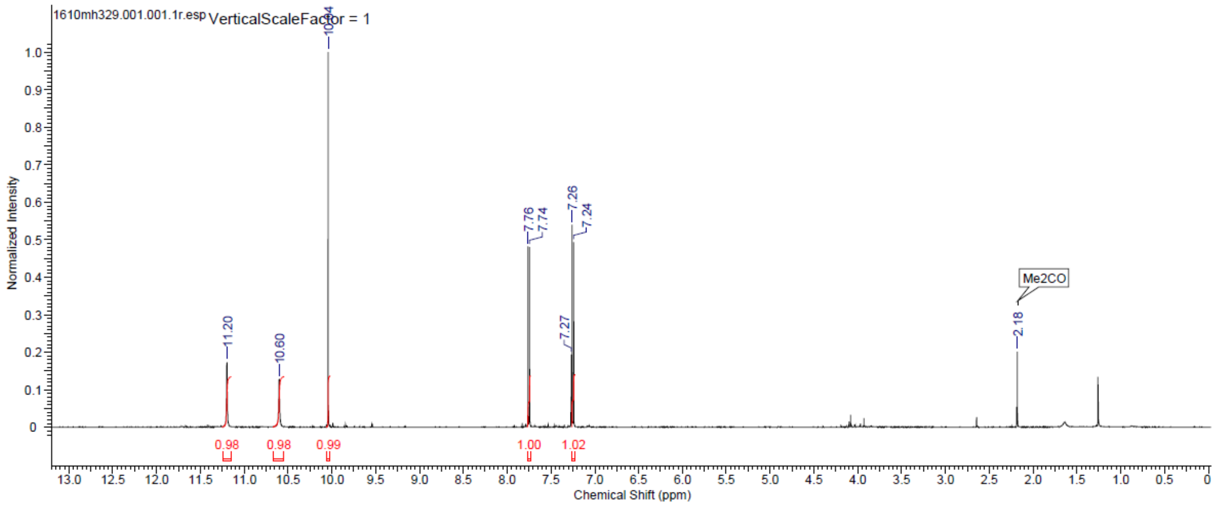
1610mh329.001.001.1r.esp VerticalScaleFactor = 1
Normalized Intensity
11.20
10.60
7.76
7.74
7.26
7.24
7.27
Me2CO
2.18
0.98
0.98
0.99
1.00
1.02
Chemical Shift (ppm)

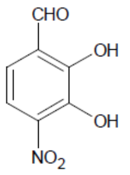
CHO
OH
OH
NO2

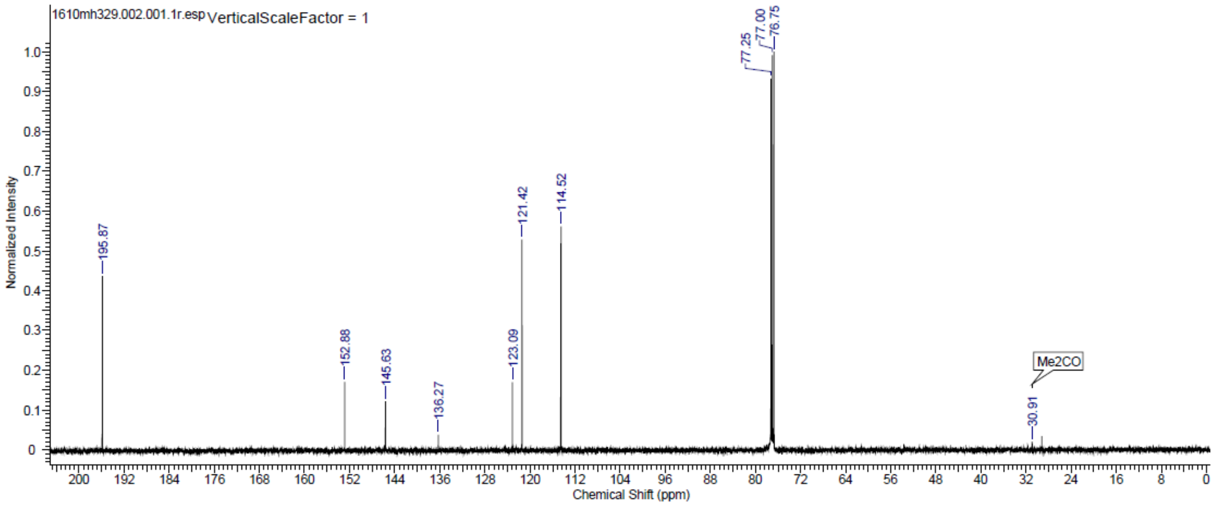
1610mh329.002.001.1r.esp VerticalScaleFactor = 1
Normalized Intensity
195.87
152.88
145.63
136.27
123.09
121.42
114.52
77.25
77.00
76.75
Me2CO
30.91
Chemical Shift (ppm)

Compound **43**

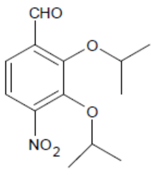
CHO
O
O
NO_2

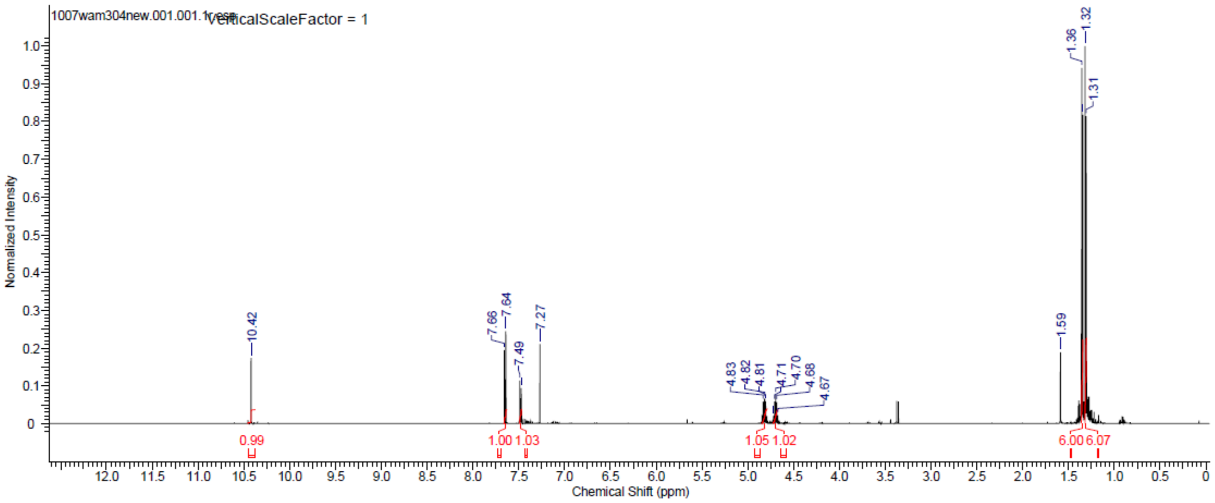
1007wam304new.001.001.1r.esp
VerticalScaleFactor = 1
Normalized Intensity
Chemical Shift (ppm)
10.42
7.66
7.64
7.49
7.27
4.83
4.82
4.81
4.71
4.70
4.68
4.67
1.59
1.36
1.32
1.31
0.99
1.00
1.03
1.05
1.02
6.00
6.07

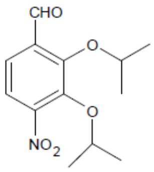
CHO
O
O
NO_2

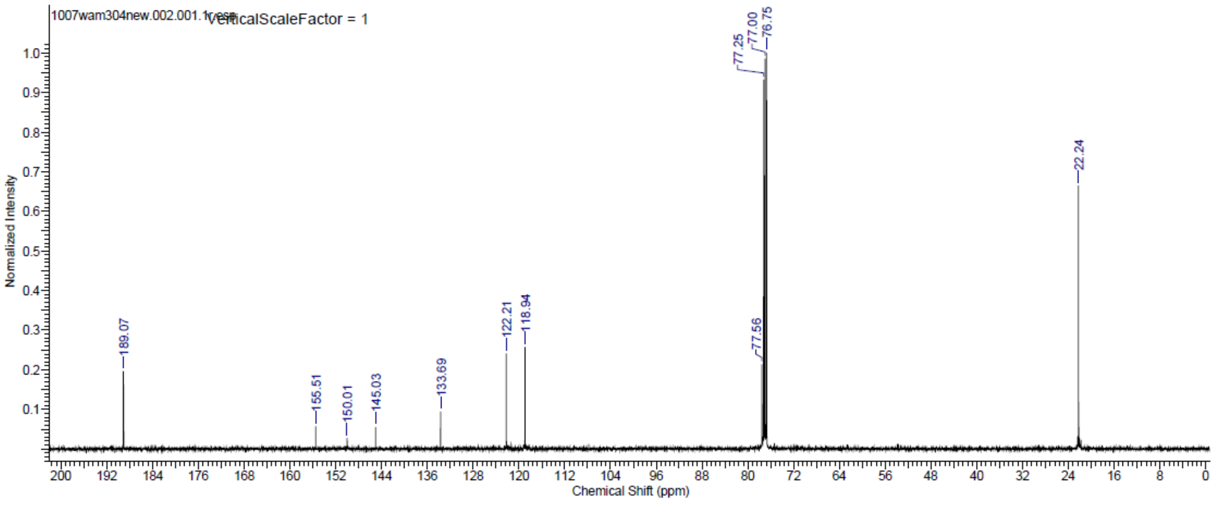
1007wam304new.002.001.1r.esp
VerticalScaleFactor = 1
Normalized Intensity
Chemical Shift (ppm)
189.07
155.51
150.01
145.03
133.69
122.21
118.94
77.56
77.25
77.00
76.75
22.24

Compound **44**

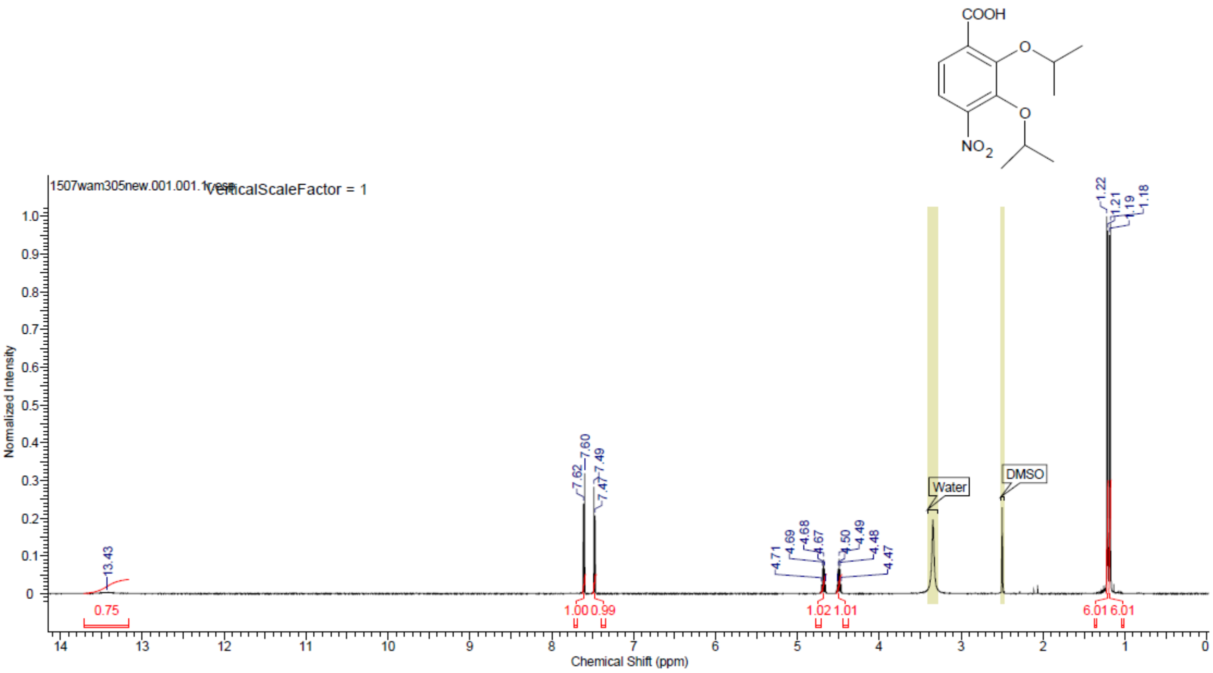

COOH
O
O
NO2
1507wam305new.001.001.1r.esp
VerticalScaleFactor = 1
Normalized Intensity
Chemical Shift (ppm)
13.43
7.62
7.60
7.49
7.47
4.71
4.69
4.68
4.67
4.50
4.49
4.48
4.47
1.22
1.21
1.19
1.18
Water
DMSO
0.75
1.00
0.99
1.02
1.01
6.01
6.01

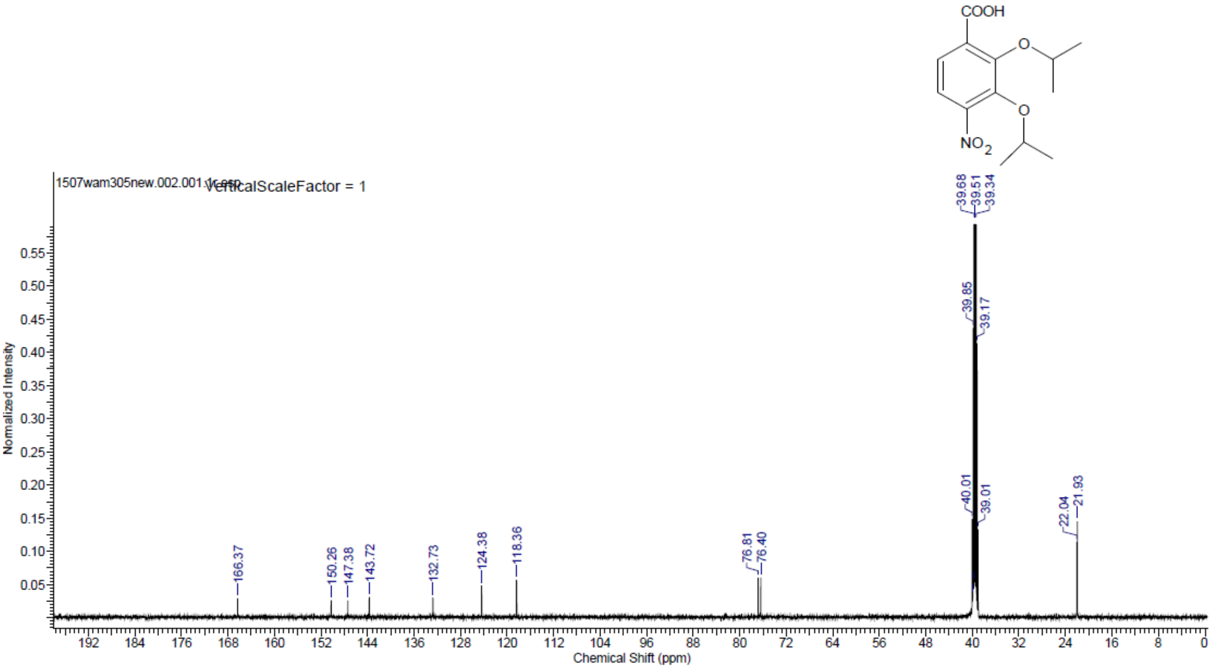

COOH
O
O
NO2
1507wam305new.002.001.1r.esp
VerticalScaleFactor = 1
Normalized Intensity
Chemical Shift (ppm)
166.37
150.26
147.38
143.72
132.73
124.38
118.36
76.81
76.40
39.68
39.51
39.34
39.85
39.17
40.01
39.01
22.04
21.93

Compound **45**

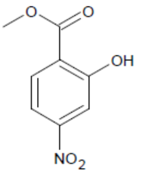
O
O
OH
NO2

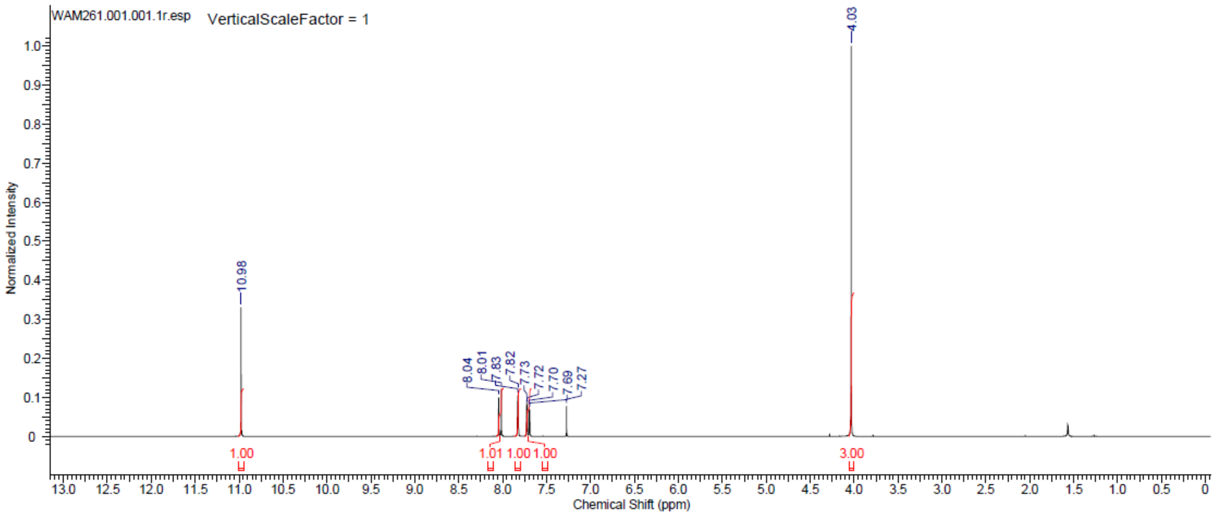
WAM261.001.001.1r.esp
VerticalScaleFactor = 1
Normalized Intensity
Chemical Shift (ppm)
10.98
8.04
8.01
7.83
7.82
7.73
7.72
7.70
7.69
7.27
4.03
1.00
1.01
1.00
1.00
3.00

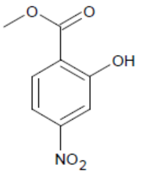
O
O
OH
NO2

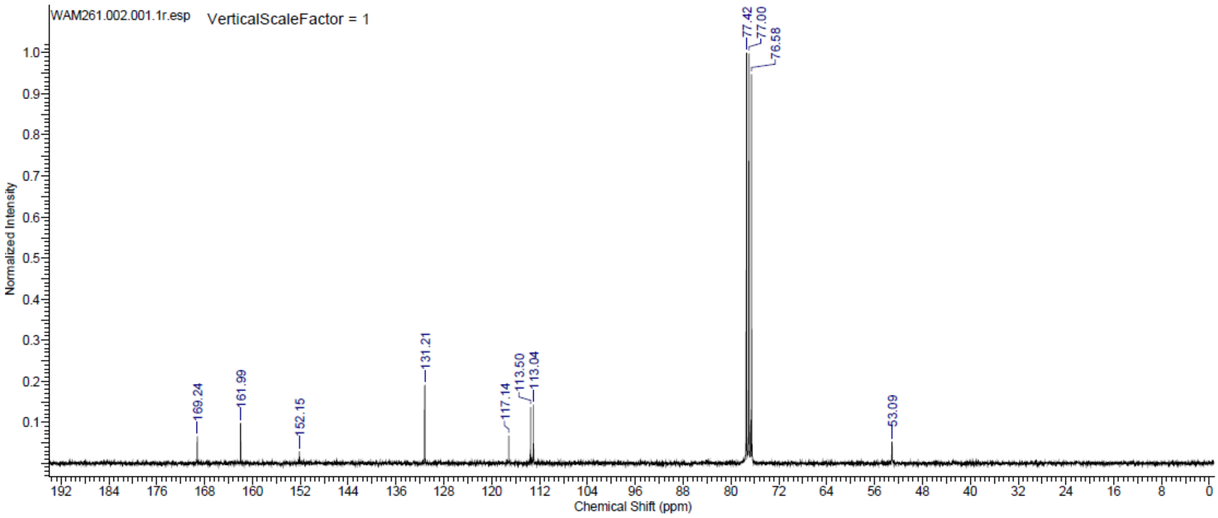
WAM261.002.001.1r.esp
VerticalScaleFactor = 1
Normalized Intensity
Chemical Shift (ppm)
169.24
161.99
152.15
131.21
117.14
113.50
113.04
77.42
77.00
76.58
53.09

Compound **46**

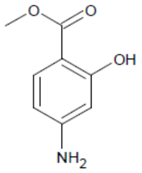
O
O
OH
NH2

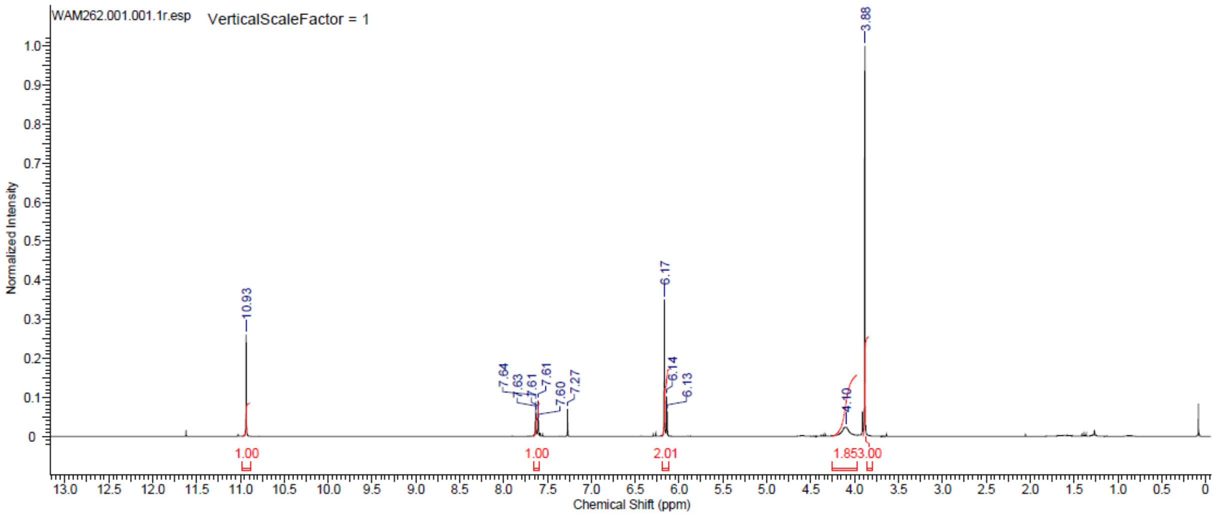
WAM262.001.001.1r.esp
VerticalScaleFactor = 1
Normalized Intensity
Chemical Shift (ppm)
10.93
7.64
7.63
7.61
7.61
7.60
7.27
6.17
6.14
6.13
4.10
3.88
1.00
1.00
2.01
1.85
3.00

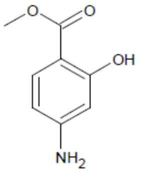
O
O
OH
NH2

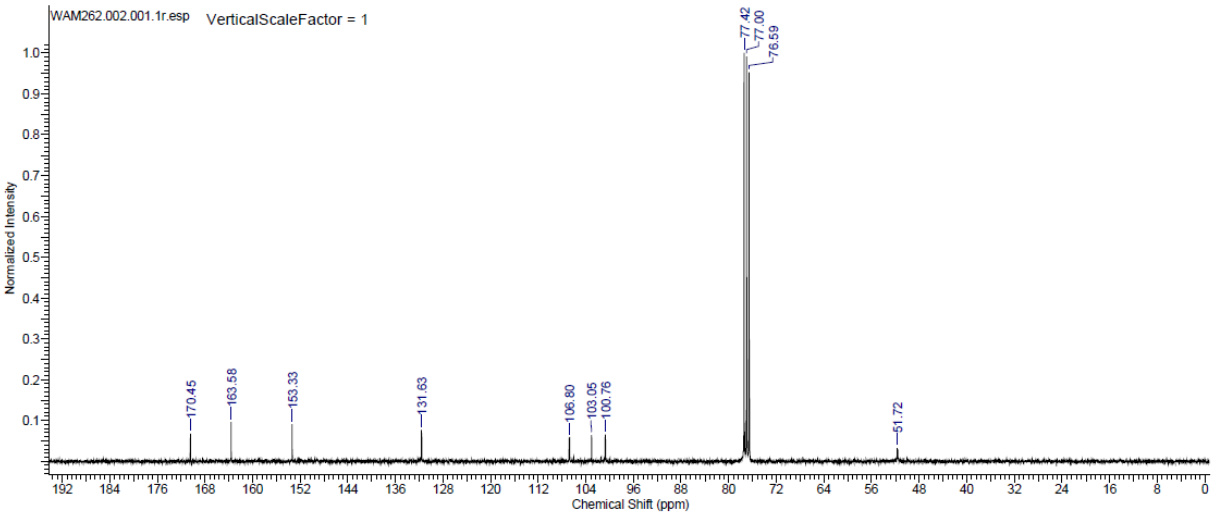
WAM262.002.001.1r.esp
VerticalScaleFactor = 1
Normalized Intensity
Chemical Shift (ppm)
170.45
163.58
153.33
131.63
106.80
103.05
100.76
77.42
77.00
76.59
51.72

Compound **47**

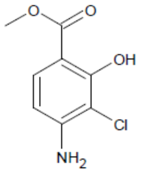
O
O
OH
Cl
NH2

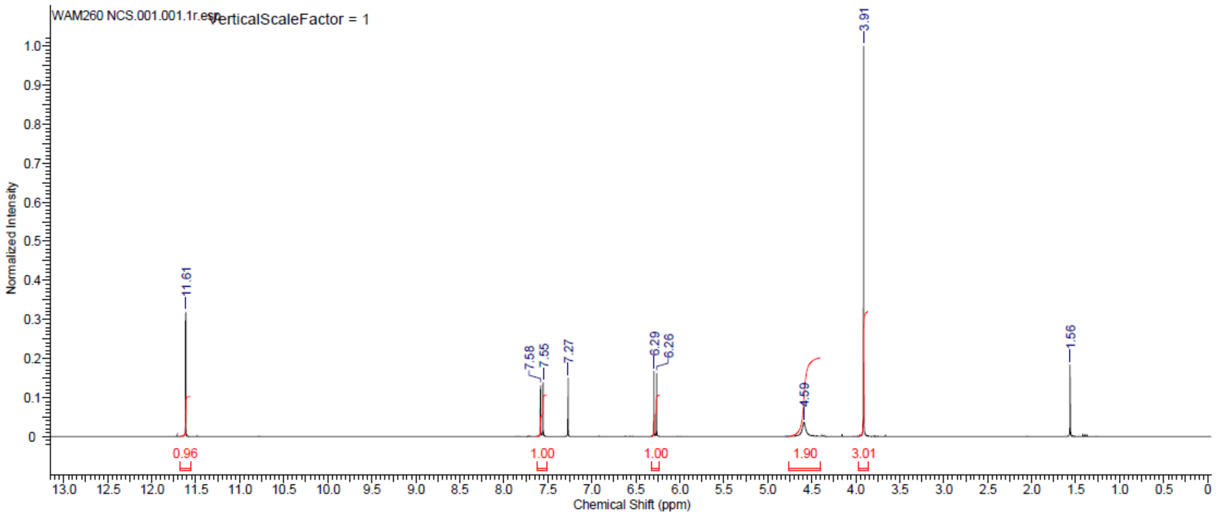
WAM260 NCS.001.001.1r.esp
VerticalScaleFactor = 1
Normalized Intensity
11.61
7.58
7.55
7.27
6.29
6.26
4.59
3.91
1.56
0.96
1.00
1.00
1.90
3.01
Chemical Shift (ppm)

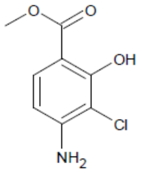
O
O
OH
Cl
NH2

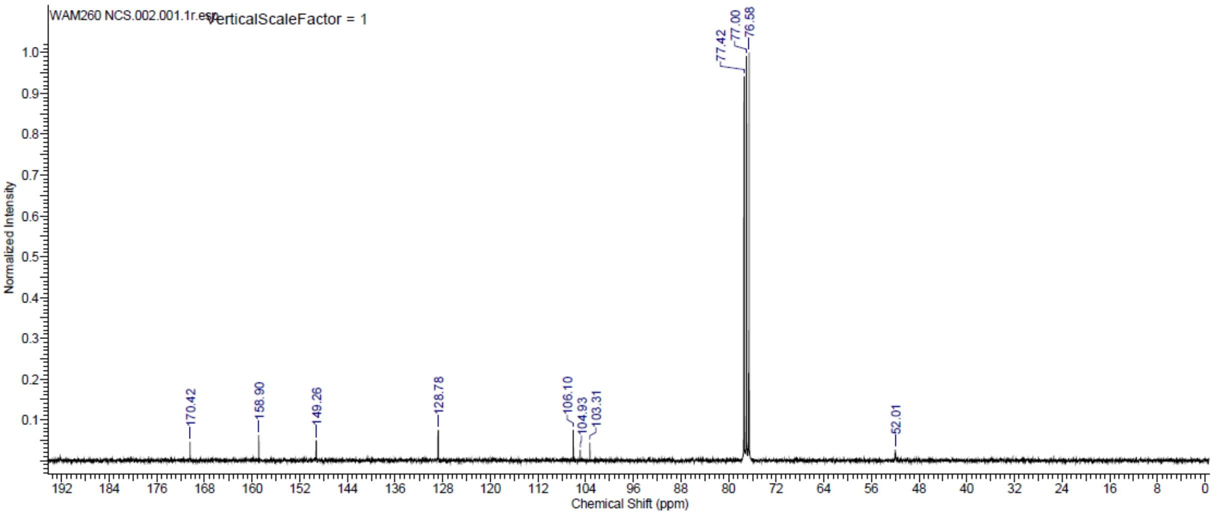
WAM260 NCS.002.001.1r.esp
VerticalScaleFactor = 1
Normalized Intensity
170.42
158.90
149.26
128.78
106.10
104.93
103.31
77.42
77.00
76.58
52.01
Chemical Shift (ppm)

Compound **48**

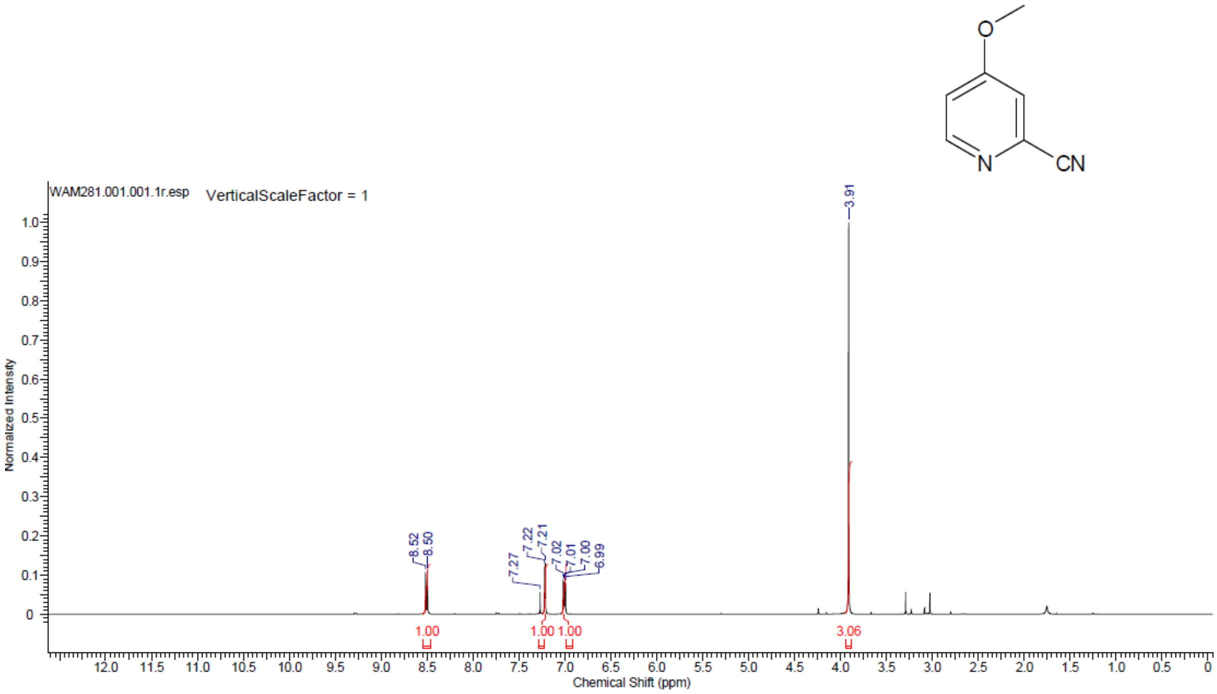

WAM281.001.001.1r.esp
VerticalScaleFactor = 1
Normalized Intensity
Chemical Shift (ppm)
3.91
8.52
8.50
7.27
7.22
7.21
7.02
7.01
7.00
6.99
1.00
1.00
1.00
3.06
O
N
CN

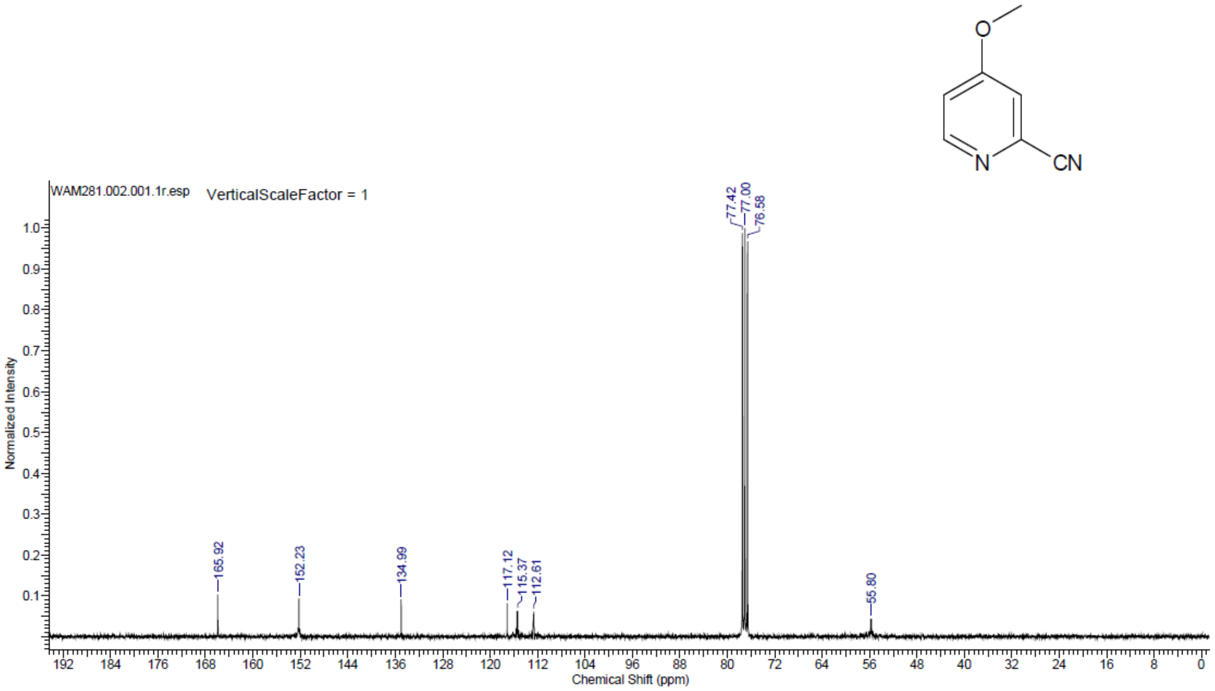

WAM281.002.001.1r.esp
VerticalScaleFactor = 1
Normalized Intensity
Chemical Shift (ppm)
77.42
77.00
76.58
165.92
152.23
134.99
117.12
115.37
112.61
55.80
O
N
CN

Compound **49**

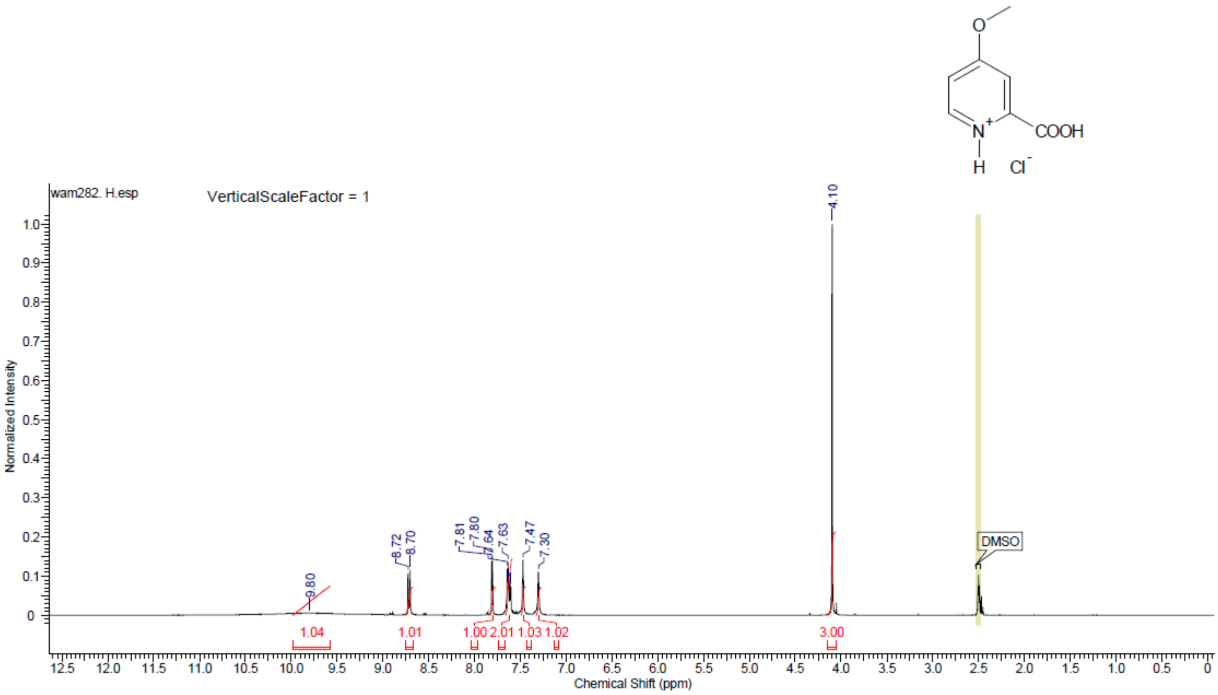

O
N+
COOH
H
Cl-
wam282. H.esp
VerticalScaleFactor = 1
Normalized Intensity
Chemical Shift (ppm)
DMSO
4.10
9.80
8.72
8.70
7.81
7.80
7.64
7.63
7.47
7.30
1.04
1.01
1.00
2.01
1.03
1.02
3.00

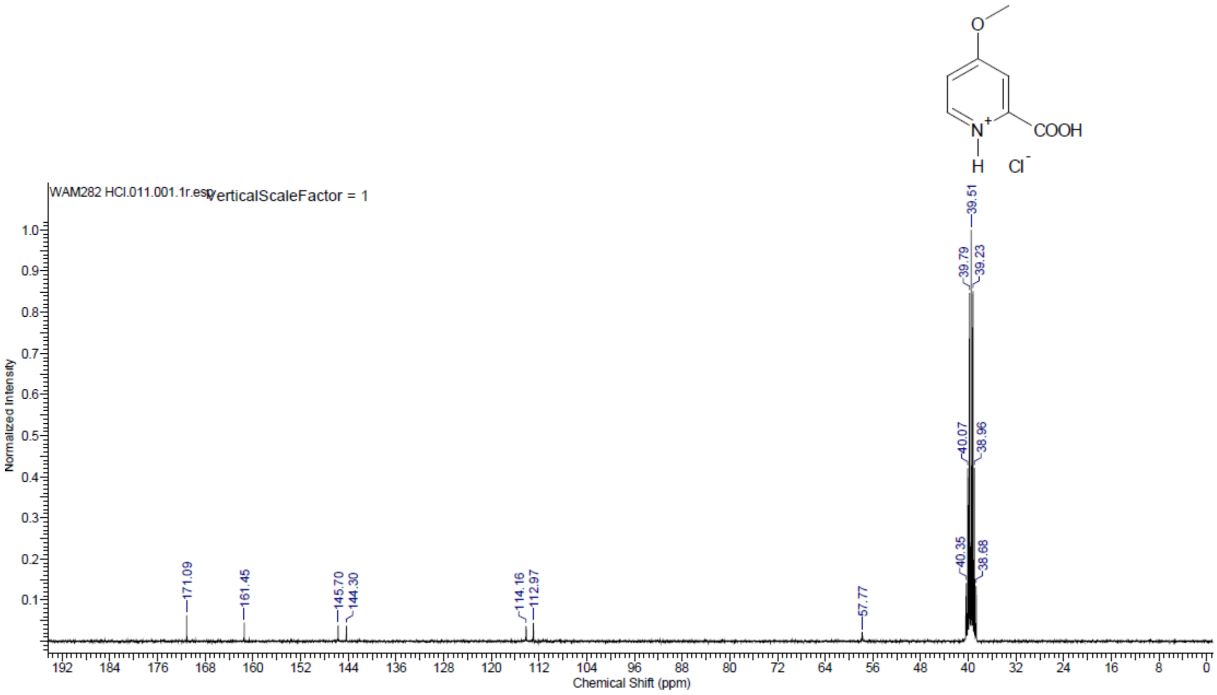

O
N+
COOH
H
Cl-
WAM282 HCl.011.001.1r.esp
VerticalScaleFactor = 1
Normalized Intensity
Chemical Shift (ppm)
171.09
161.45
145.70
144.30
114.16
112.97
57.77
40.35
40.07
39.79
39.51
39.23
38.96
38.68

Compound **50**

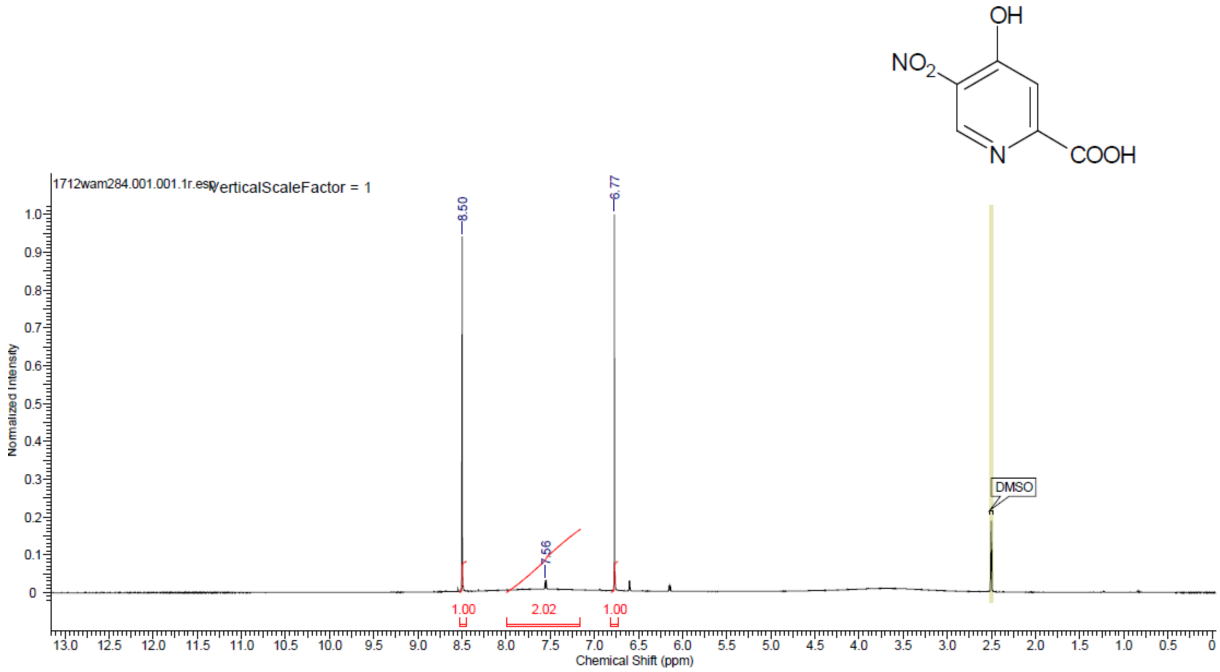
OH
NO_2
N
COOH
1712wam284.001.001.1r.esp
VerticalScaleFactor = 1
8.50
6.77
7.56
DMSO
1.00
2.02
1.00
Normalized Intensity
Chemical Shift (ppm)

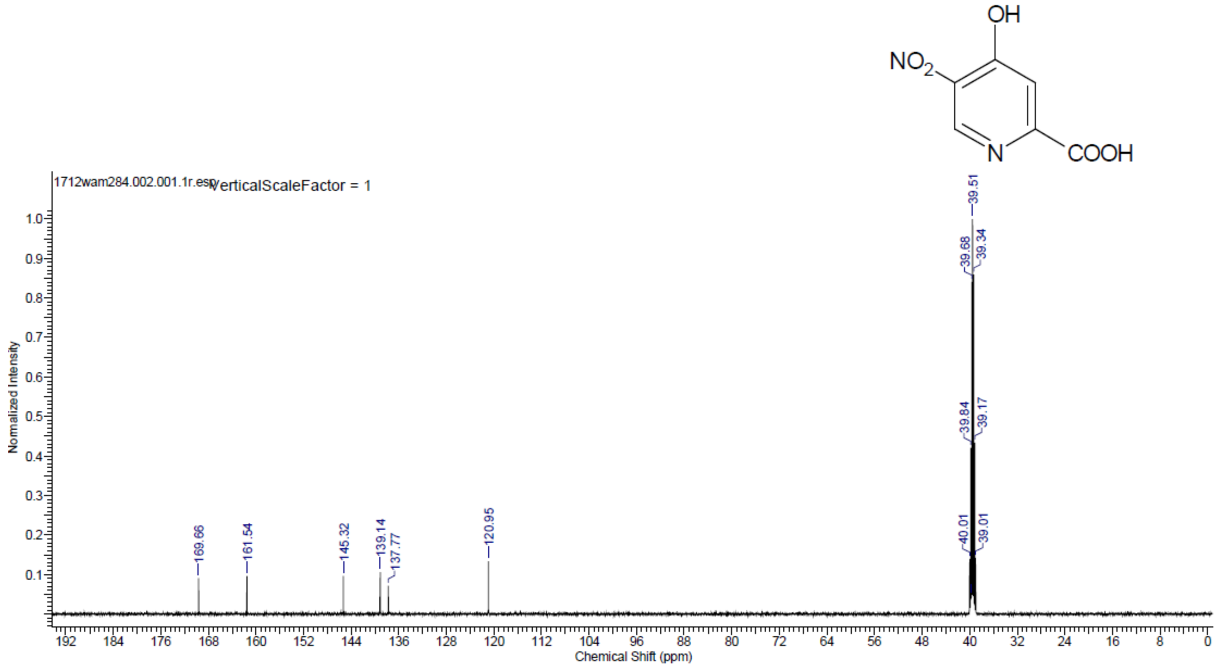
OH
NO_2
N
COOH
1712wam284.002.001.1r.esp
VerticalScaleFactor = 1
169.66
161.54
145.32
139.14
137.77
120.95
39.51
39.68
39.34
39.84
39.17
40.01
39.01
Normalized Intensity
Chemical Shift (ppm)

Compound **51**

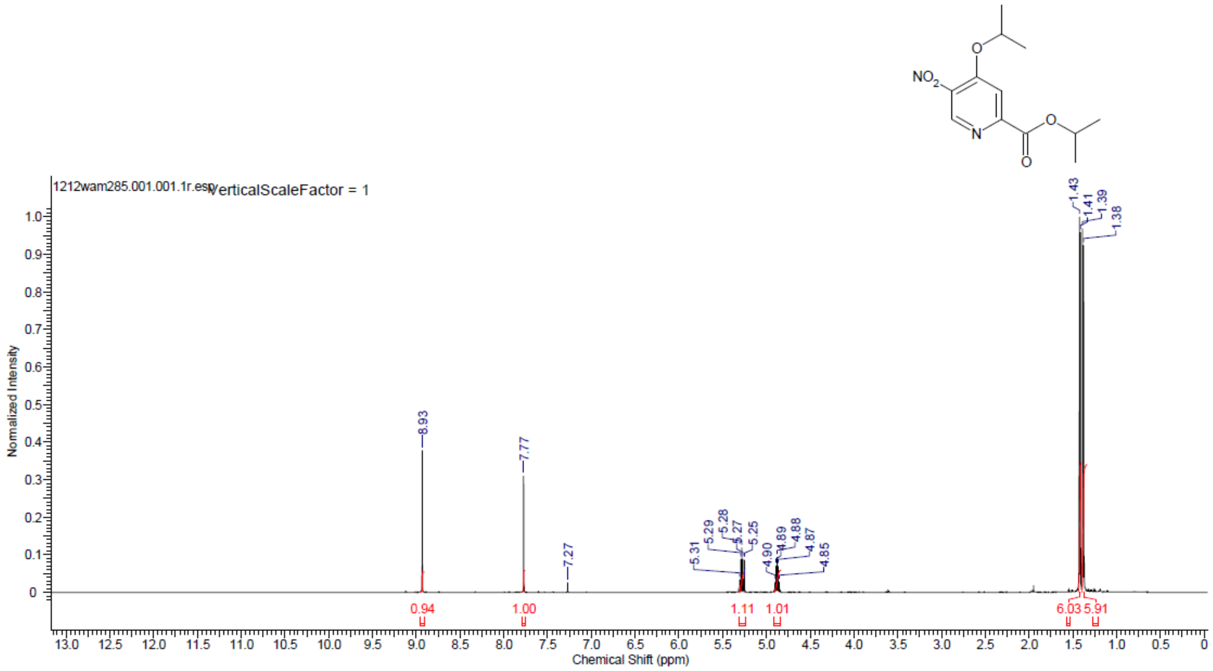
NO2
O
N
O
O
1212wam285.001.001.1r.esp
VerticalScaleFactor = 1
Normalized Intensity
Chemical Shift (ppm)
8.93
7.77
7.27
5.31
5.29
5.28
5.27
5.25
4.90
4.89
4.88
4.87
4.85
1.43
1.41
1.39
1.38
0.94
1.00
1.11
1.01
6.03
5.91

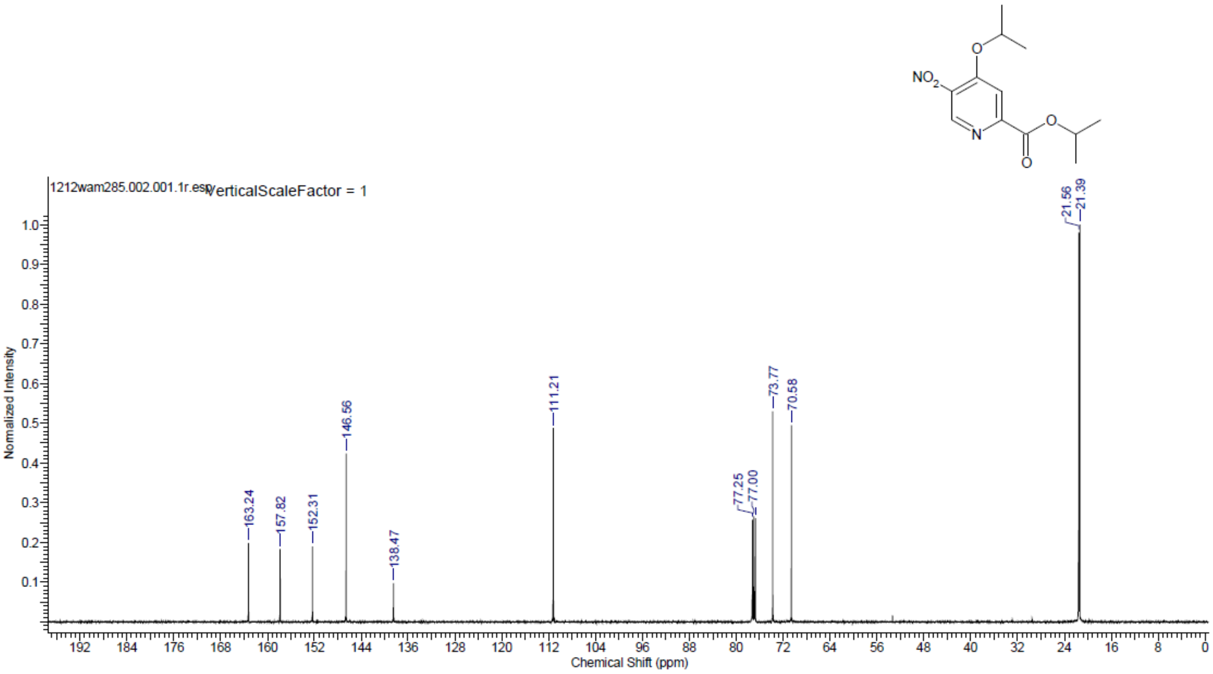
NO2
O
N
O
O
1212wam285.002.001.1r.esp
VerticalScaleFactor = 1
Normalized Intensity
Chemical Shift (ppm)
163.24
157.82
152.31
146.56
138.47
111.21
77.25
77.00
73.77
70.58
21.56
21.39

Compound **52**

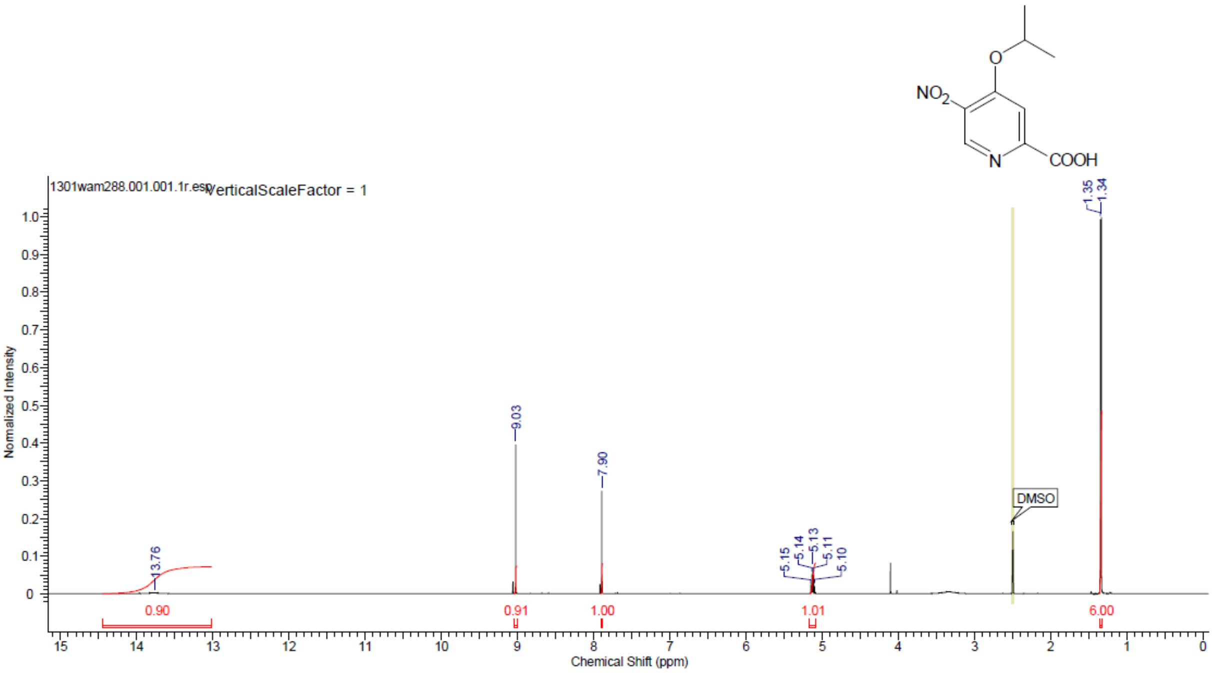
NO2
O
N
COOH
1301wam288.001.001.1r.esp
VerticalScaleFactor = 1
Normalized Intensity
Chemical Shift (ppm)
DMSO
13.76
9.03
7.90
5.15
5.14
5.13
5.11
5.10
1.35
1.34
0.90
0.91
1.00
1.01
6.00

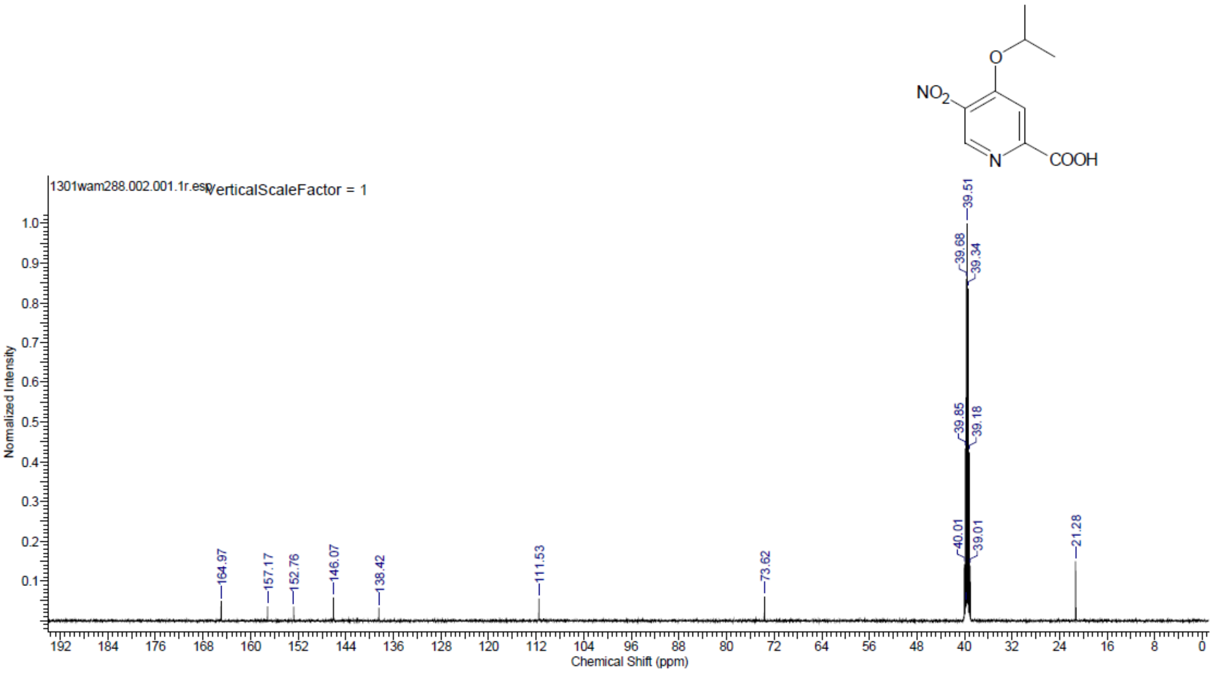
NO2
O
N
COOH
1301wam288.002.001.1r.esp
VerticalScaleFactor = 1
Normalized Intensity
Chemical Shift (ppm)
164.97
157.17
152.76
146.07
138.42
111.53
73.62
40.01
39.85
39.68
39.51
39.34
39.18
39.01
21.28

Compound **53**

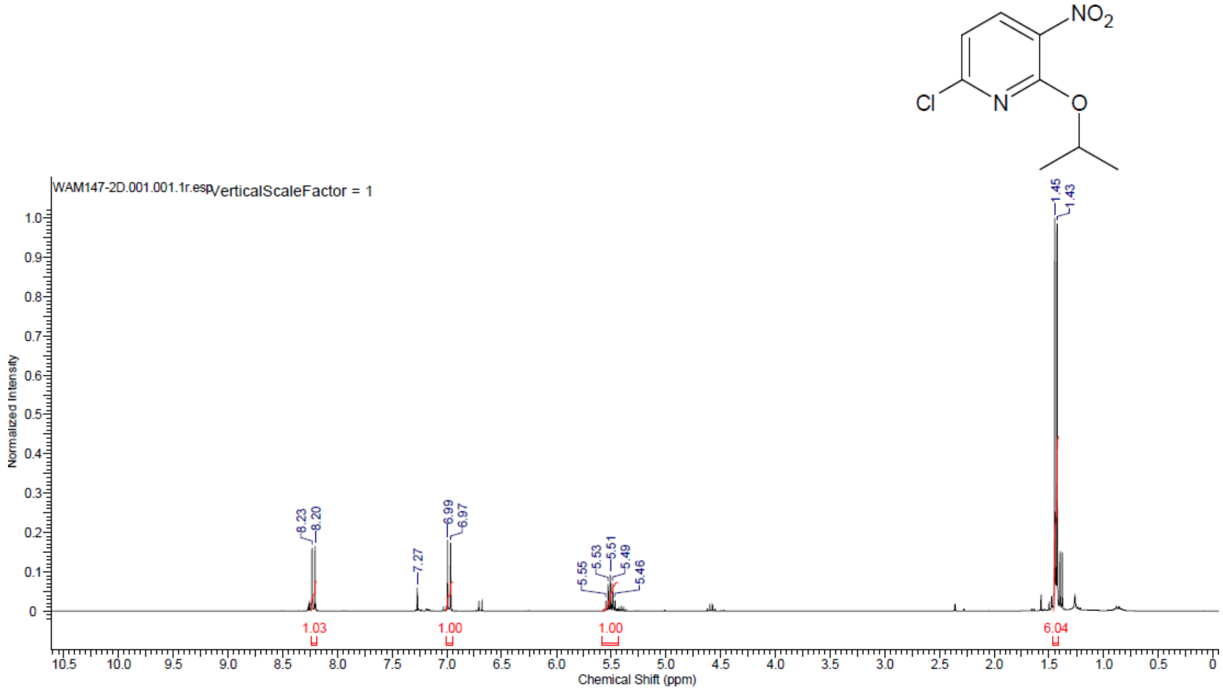
NO2
Cl
N
O
WAM147-2D.001.001.1r.esp VerticalScaleFactor = 1
Normalized Intensity
Chemical Shift (ppm)
8.23
8.20
7.27
6.99
6.97
5.55
5.53
5.51
5.49
5.46
1.45
1.43
1.03
1.00
1.00
6.04

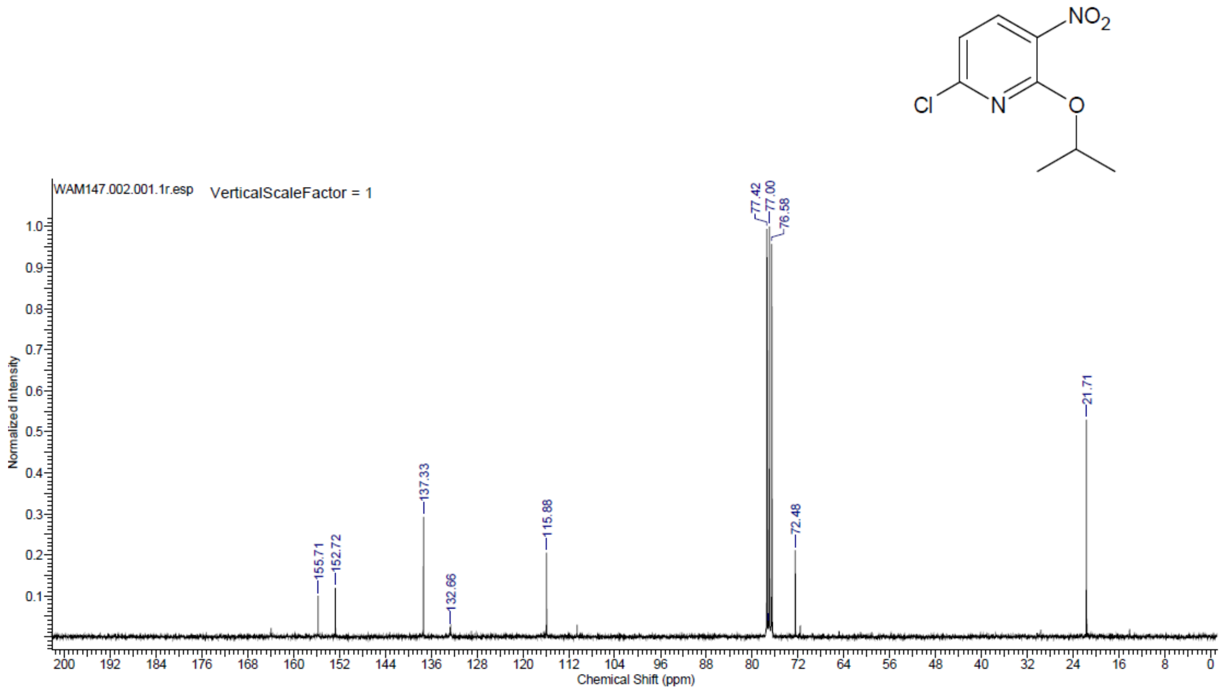
NO2
Cl
N
O
WAM147.002.001.1r.esp VerticalScaleFactor = 1
Normalized Intensity
Chemical Shift (ppm)
155.71
152.72
137.33
132.66
115.88
77.42
77.00
76.58
72.48
21.71

Compound **54**

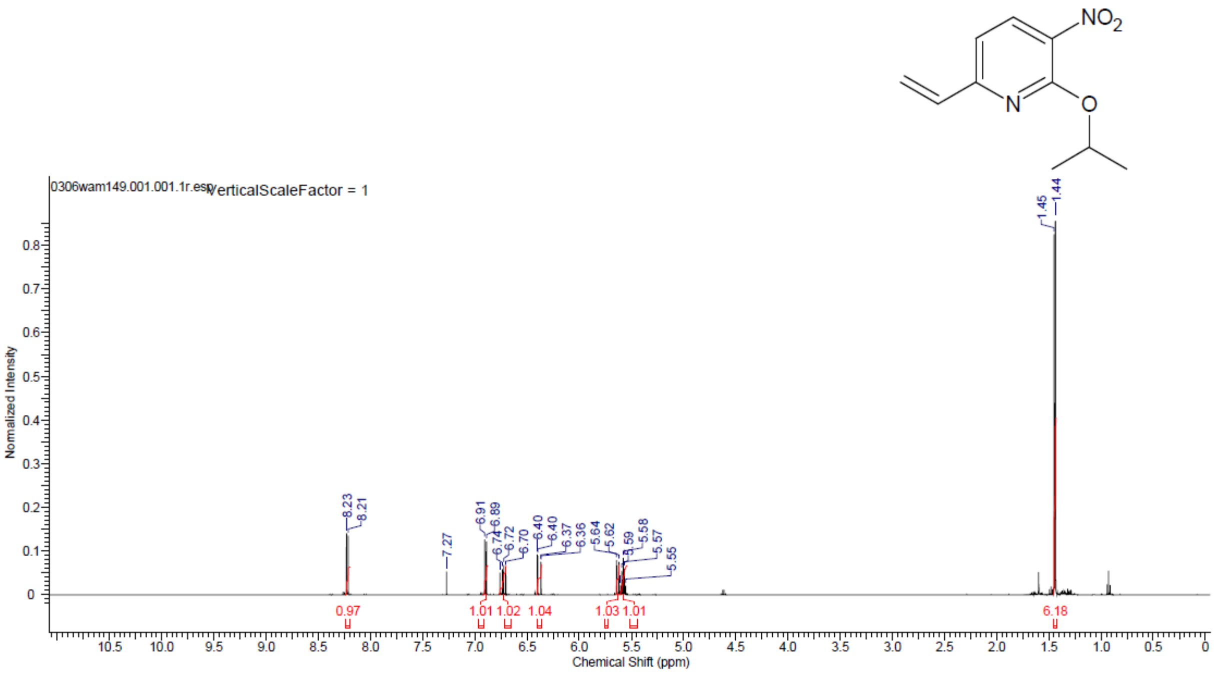
NO2
N
O
0306wam149.001.001.1r.esp
VerticalScaleFactor = 1
Normalized Intensity
Chemical Shift (ppm)
8.23
8.21
7.27
6.91
6.89
6.74
6.72
6.70
6.40
6.40
6.37
6.36
5.64
5.62
5.59
5.58
5.57
5.55
1.45
1.44
0.97
1.01
1.02
1.04
1.03
1.01
6.18

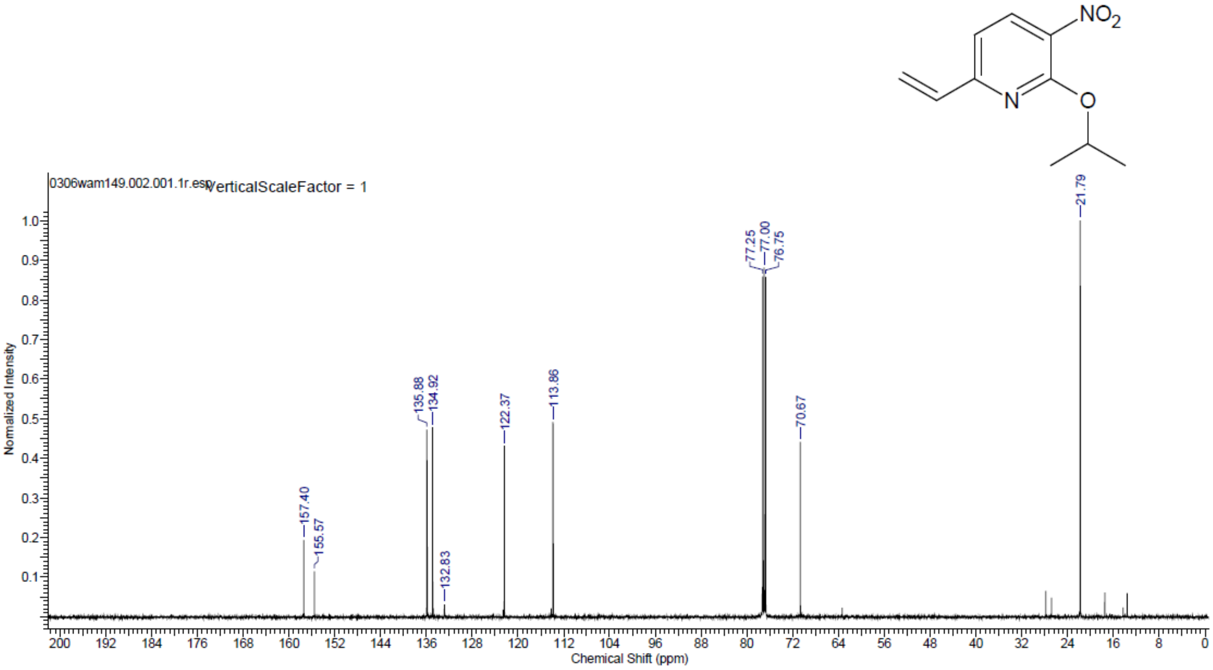
NO2
N
O
0306wam149.002.001.1r.esp
VerticalScaleFactor = 1
Normalized Intensity
Chemical Shift (ppm)
157.40
155.57
135.88
134.92
132.83
122.37
113.86
77.25
77.00
76.75
70.67
21.79

Compound **55**

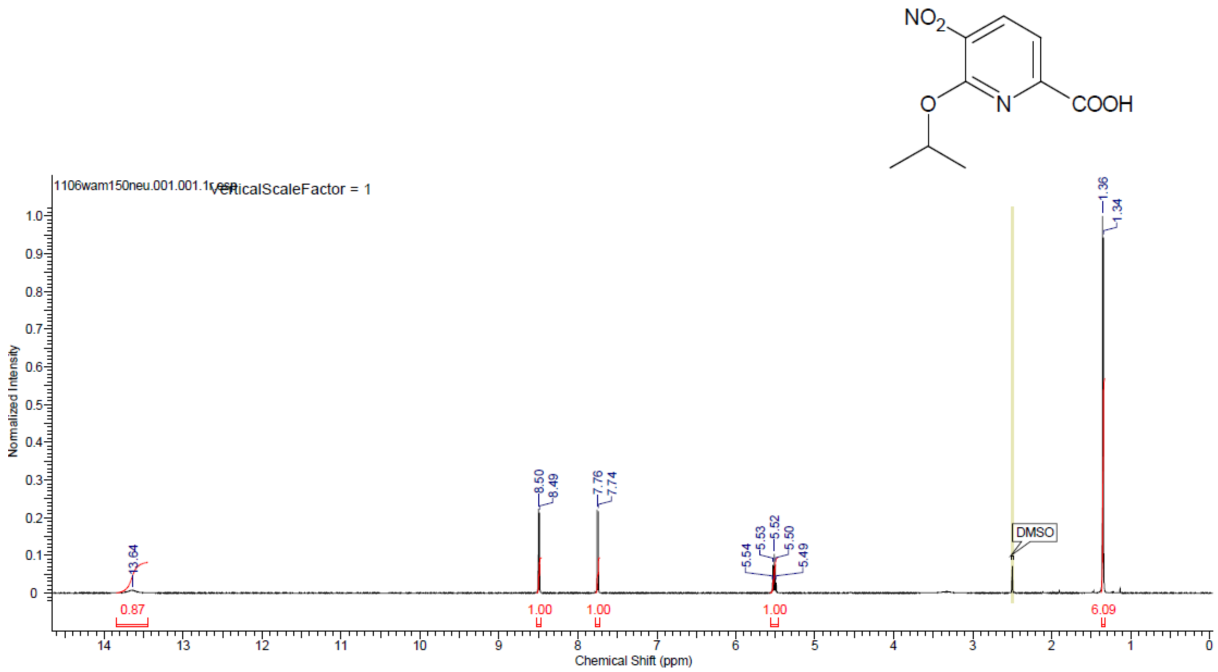

NO2
N
COOH
O
1106wam150neu.001.001.1r.esp
verticalScaleFactor = 1
Normalized Intensity
Chemical Shift (ppm)
13.64
8.50
8.49
7.76
7.74
5.54
5.53
5.52
5.50
5.49
DMSO
1.36
1.34
0.87
1.00
1.00
1.00
6.09

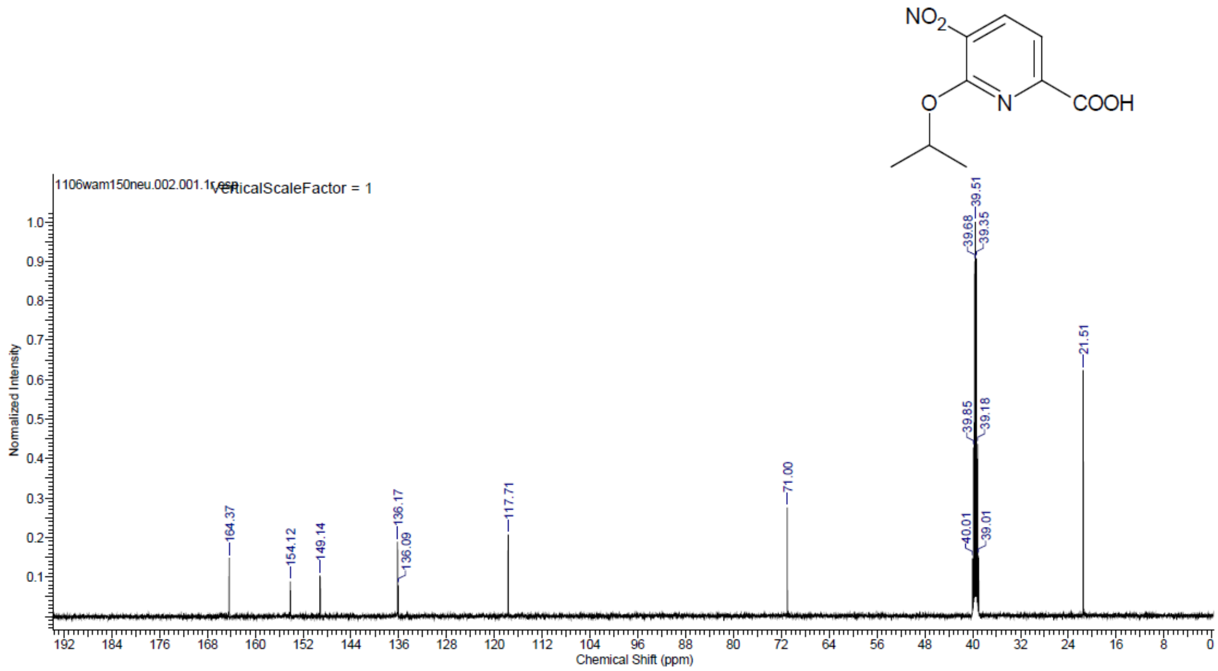

NO2
N
COOH
O
1106wam150neu.002.001.1r.esp
verticalScaleFactor = 1
Normalized Intensity
Chemical Shift (ppm)
164.37
154.12
149.14
136.17
136.09
117.71
71.00
40.01
39.85
39.68
39.51
39.35
39.18
39.01
21.51

Compound **21**

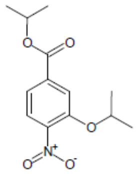

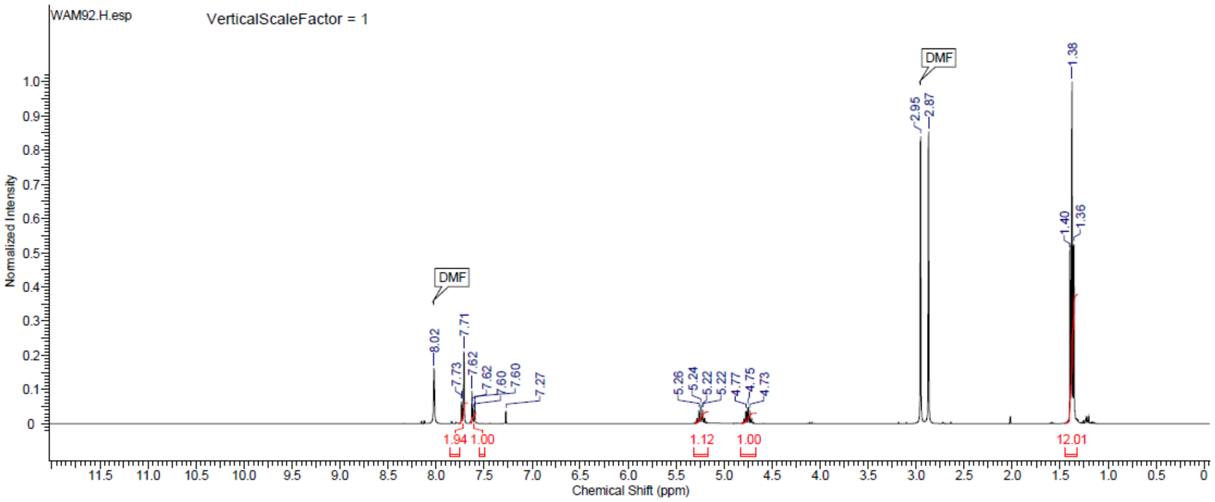

WAM92.H.esp
VerticalScaleFactor = 1
DMF
8.02
7.73
7.71
7.62
7.60
7.60
7.27
5.26
5.24
5.22
5.22
4.77
4.75
4.73
2.95
2.87
1.40
1.38
1.36
1.94
1.00
1.12
1.00
12.01
Normalized Intensity
Chemical Shift (ppm)

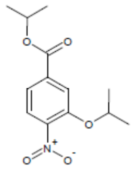

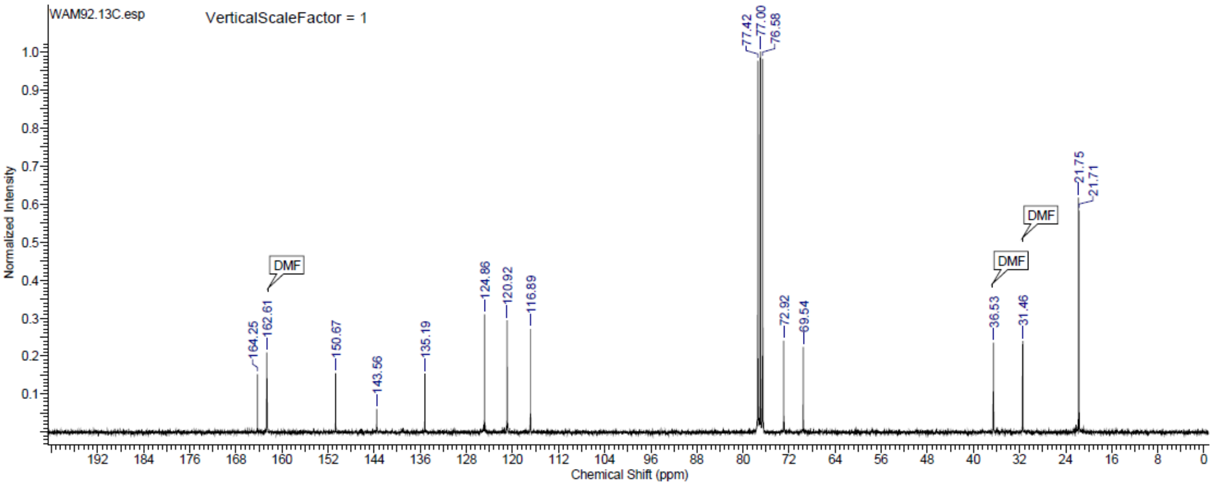

WAM92.13C.esp
VerticalScaleFactor = 1
DMF
164.25
162.61
150.67
143.56
135.19
124.86
120.92
116.89
77.42
77.00
76.58
72.92
69.54
36.53
31.46
21.75
21.71
Normalized Intensity
Chemical Shift (ppm)

Compound **27**

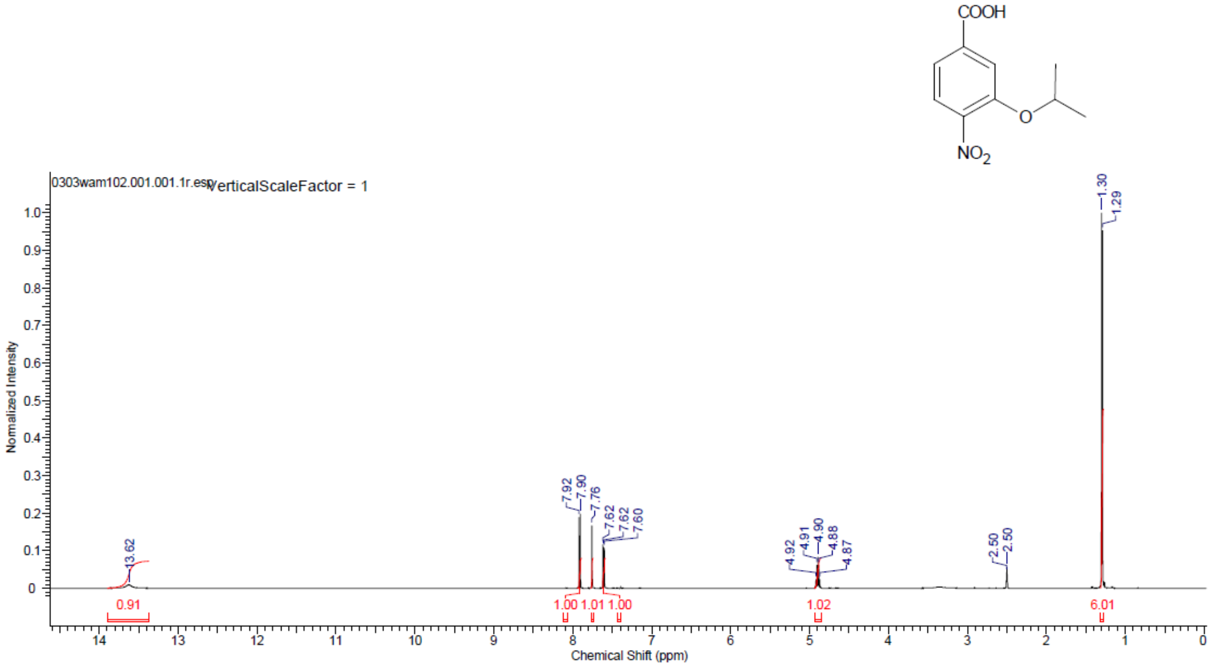

COOH
O
NO_2
0303wam102.001.001.1r.esp
VerticalScaleFactor = 1
Normalized Intensity
Chemical Shift (ppm)
13.62
7.92
7.90
7.76
7.62
7.62
7.60
4.92
4.91
4.90
4.88
4.87
2.50
2.50
1.30
1.29
0.91
1.00
1.01
1.00
1.02
6.01

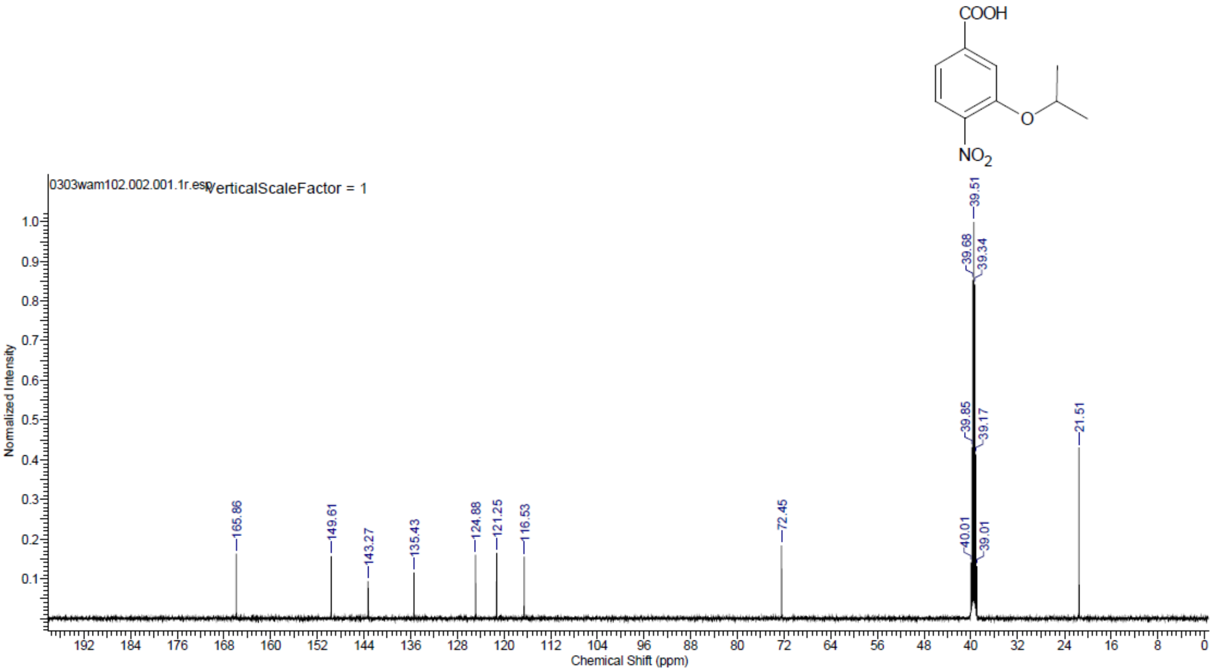

COOH
O
NO_2
0303wam102.002.001.1r.esp
VerticalScaleFactor = 1
Normalized Intensity
Chemical Shift (ppm)
165.86
149.61
143.27
135.43
124.88
121.25
116.53
72.45
40.01
39.85
39.68
39.51
39.34
39.17
39.01
21.51

Compound **22**

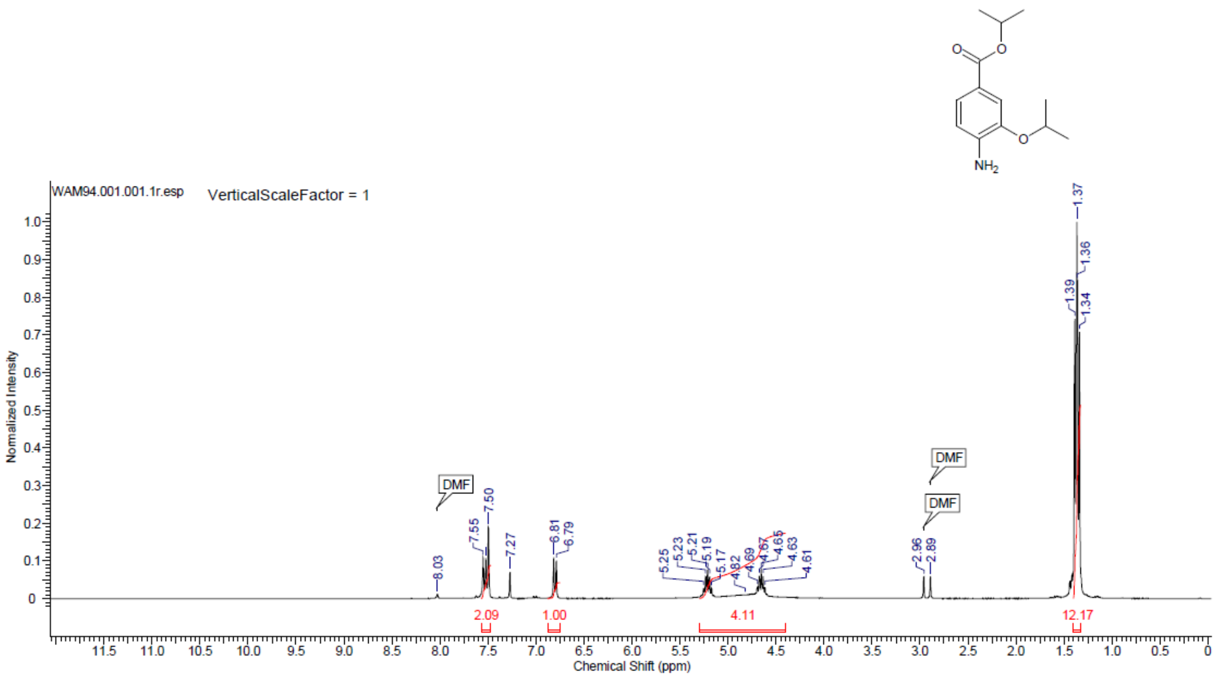
WAM94.001.001.1r.esp
VerticalScaleFactor = 1
Normalized Intensity
Chemical Shift (ppm)
DMF
DMF
DMF
NH2
O
O
O
2.09
1.00
4.11
12.17

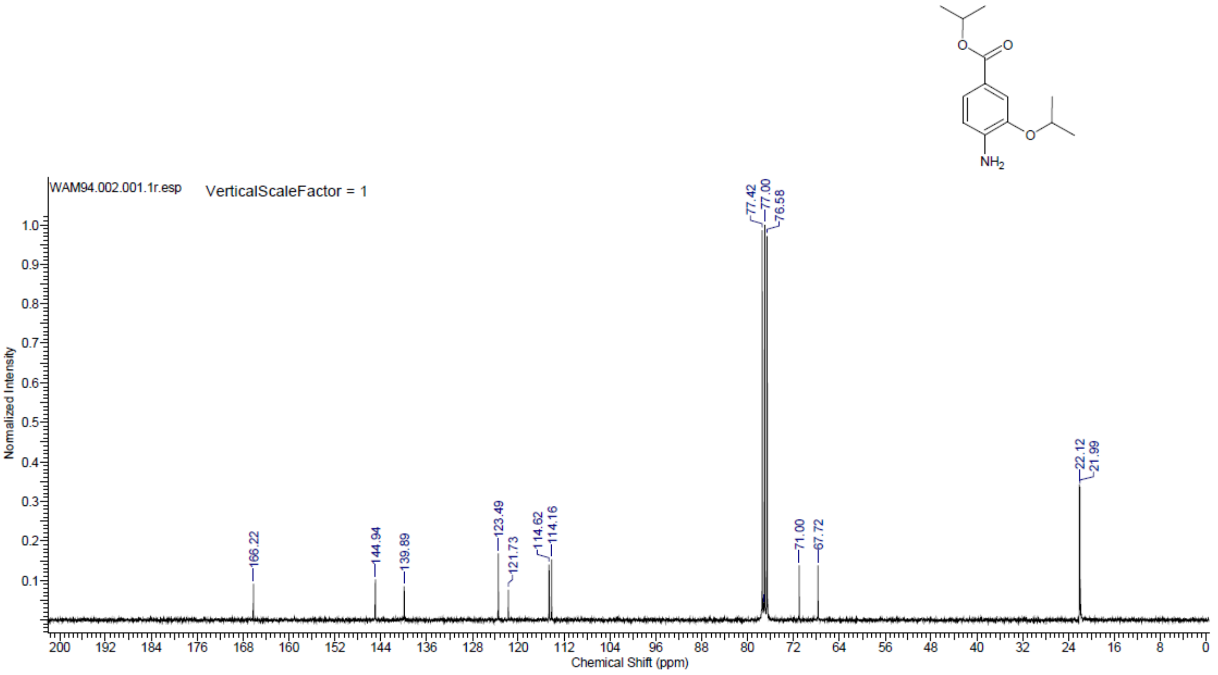
WAM94.002.001.1r.esp
VerticalScaleFactor = 1
Normalized Intensity
Chemical Shift (ppm)
NH2
O
O
O

Compound **23**

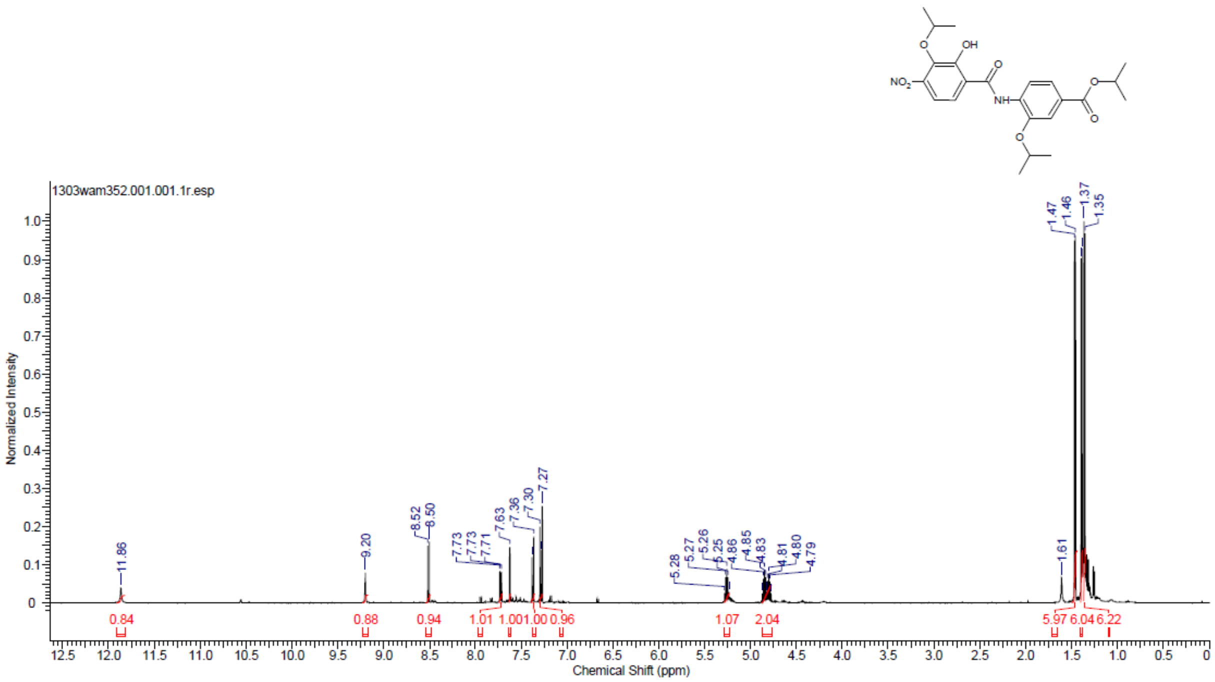

OH
O
NO2
NH
O
O
O
1303wam352.001.001.1r.esp
Normalized Intensity
Chemical Shift (ppm)
11.86
9.20
8.52
8.50
7.73
7.73
7.71
7.63
7.36
7.30
7.27
5.28
5.27
5.26
5.25
4.86
4.85
4.83
4.81
4.80
4.79
1.61
1.47
1.46
1.37
1.35
0.84
0.88
0.94
1.01
1.00
1.00
0.96
1.07
2.04
5.97
6.04
6.22

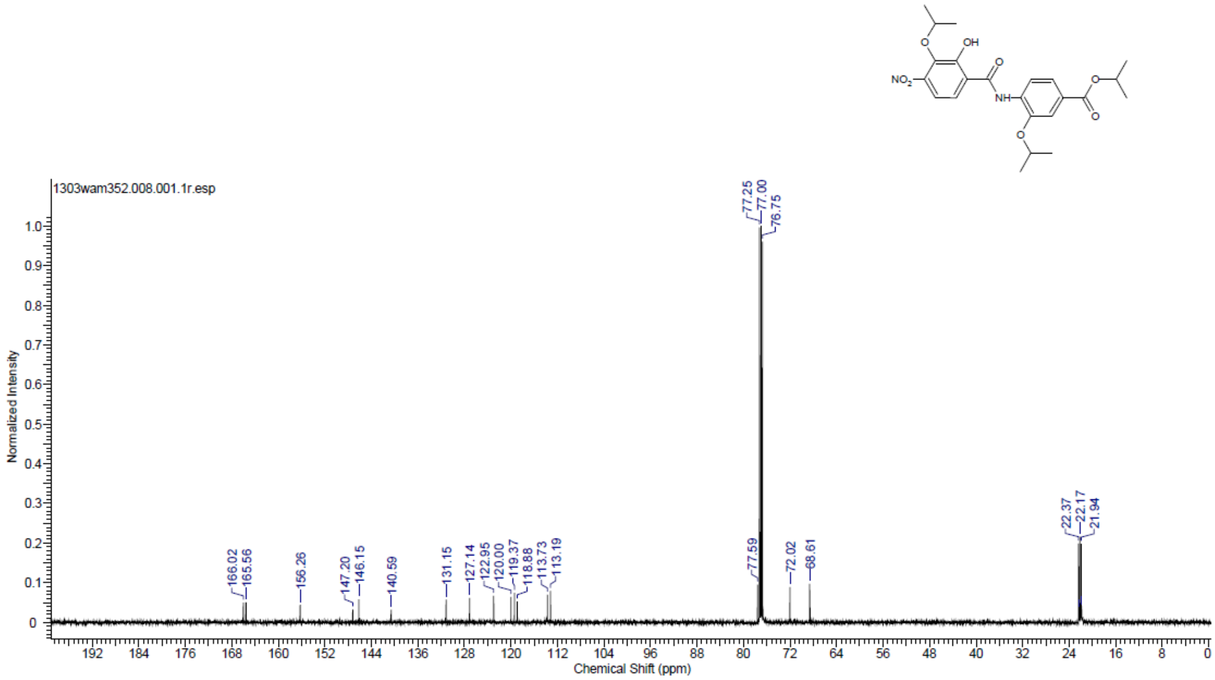

OH
O
NO2
NH
O
O
O
1303wam352.008.001.1r.esp
Normalized Intensity
Chemical Shift (ppm)
166.02
165.56
156.26
147.20
146.15
140.59
131.15
127.14
122.95
120.00
119.37
118.88
113.73
113.19
77.59
77.25
77.00
76.75
72.02
68.61
22.37
22.17
21.94

Compound **58**

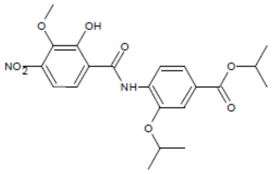

OH
O
NO2
NH
O
O
O

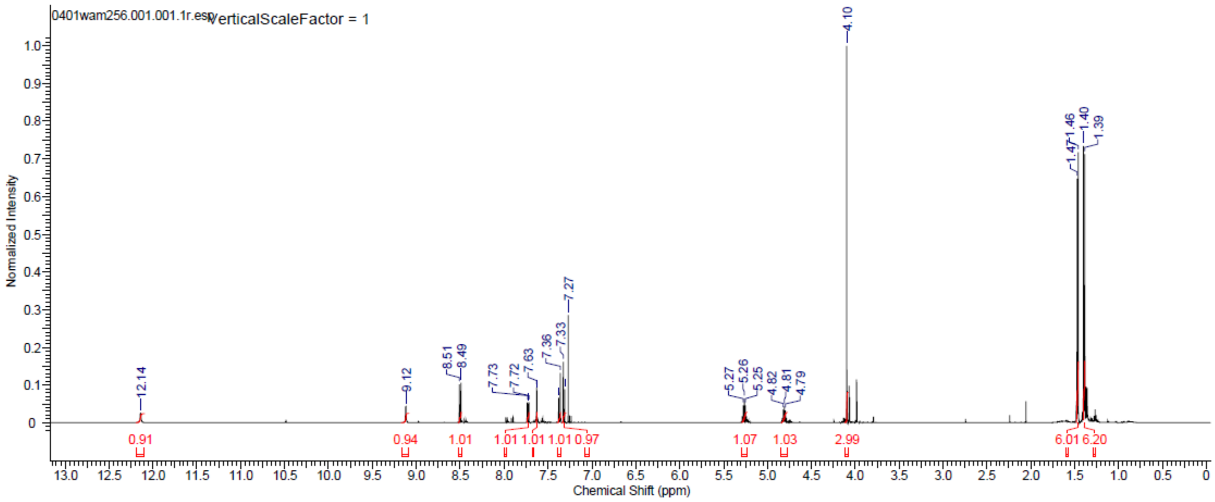

0401wam256.001.001.1r.esp
VerticalScaleFactor = 1
Normalized Intensity
12.14
9.12
8.51
8.49
7.73
7.72
7.63
7.36
7.33
7.27
5.27
5.26
5.25
4.82
4.81
4.79
4.10
1.47
1.46
1.40
1.39
0.91
0.94
1.01
1.01
1.01
1.01
0.97
1.07
1.03
2.99
6.01
6.20
Chemical Shift (ppm)

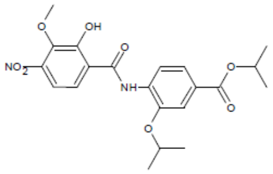

OH
O
NO2
NH
O
O
O

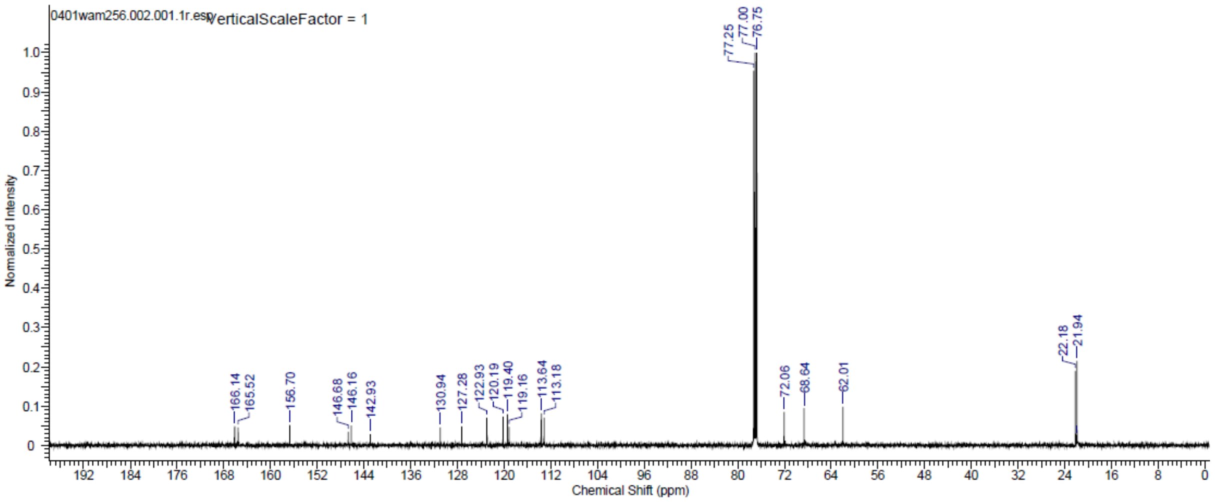

0401wam256.002.001.1r.esp
VerticalScaleFactor = 1
Normalized Intensity
166.14
165.52
156.70
146.68
146.16
142.93
130.94
127.28
122.93
120.19
119.40
119.16
113.64
113.18
77.25
77.00
76.75
72.06
68.64
62.01
22.18
21.94
Chemical Shift (ppm)

Compound **59**

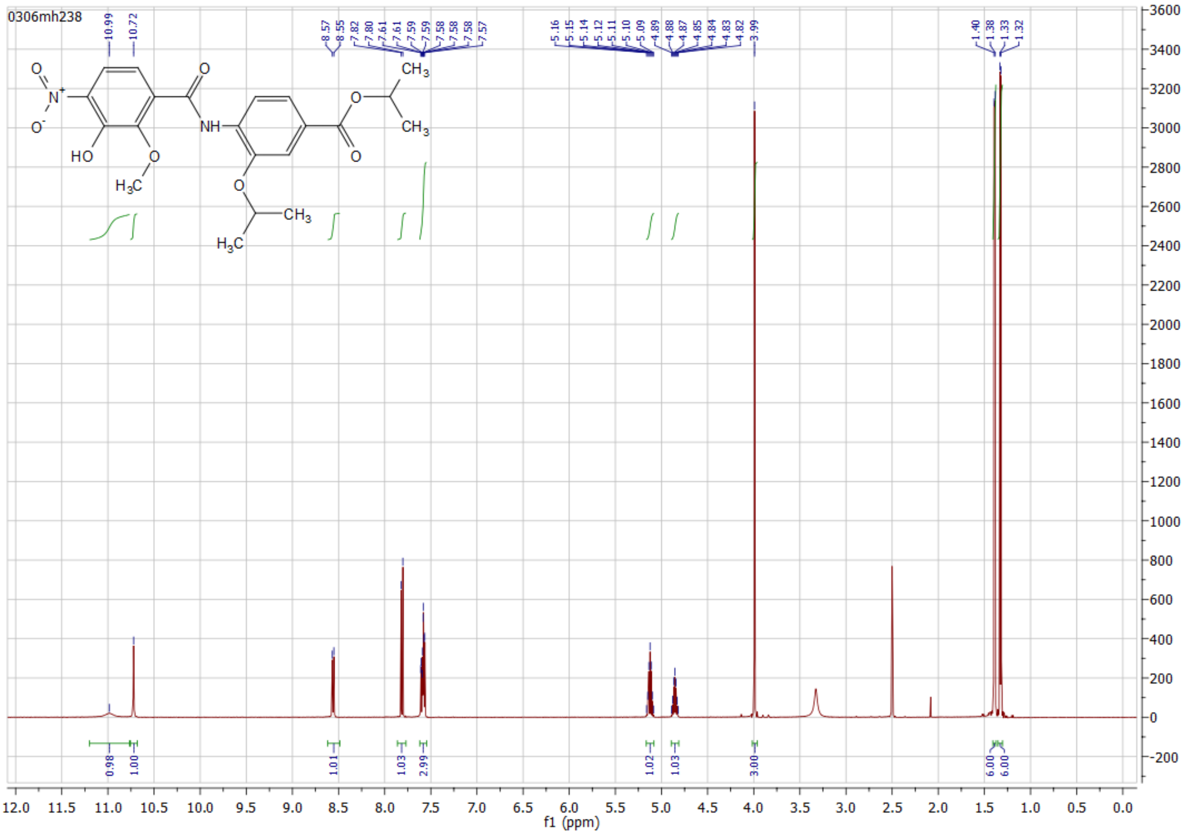

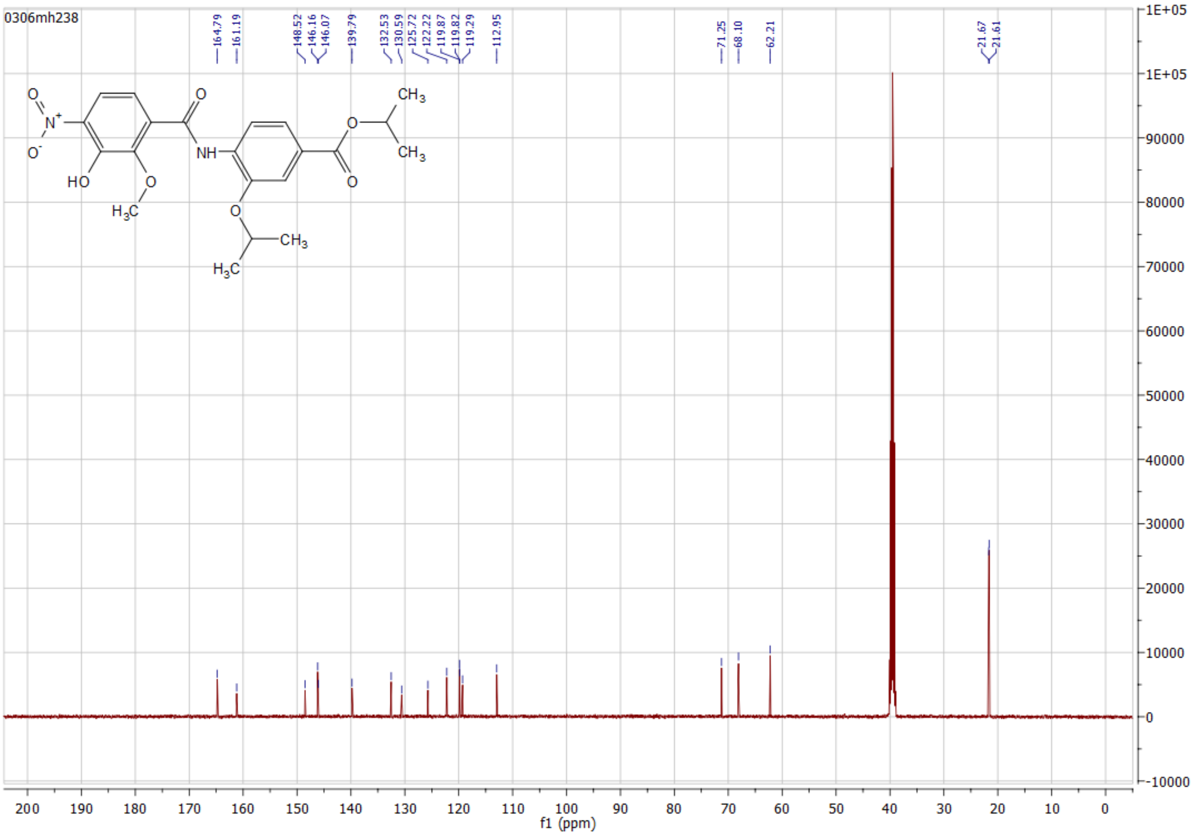

Compound **60**

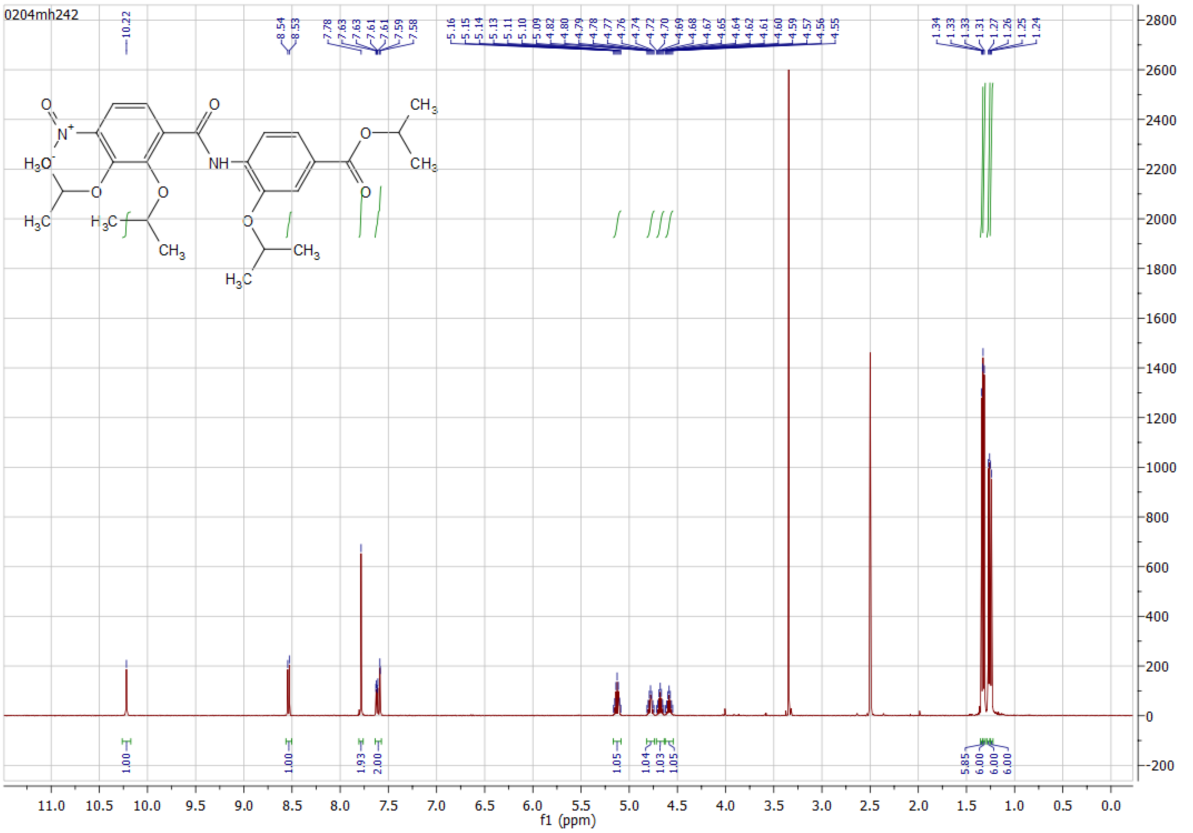

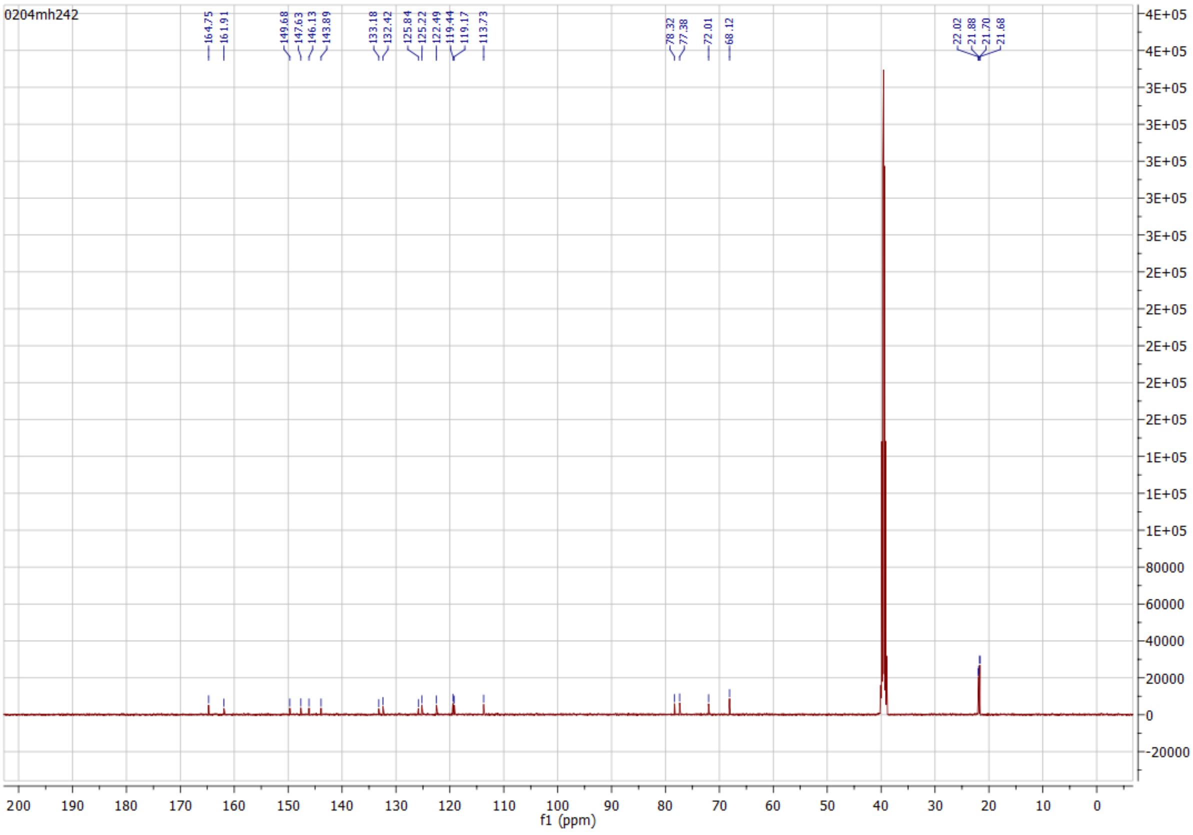

Compound **61**

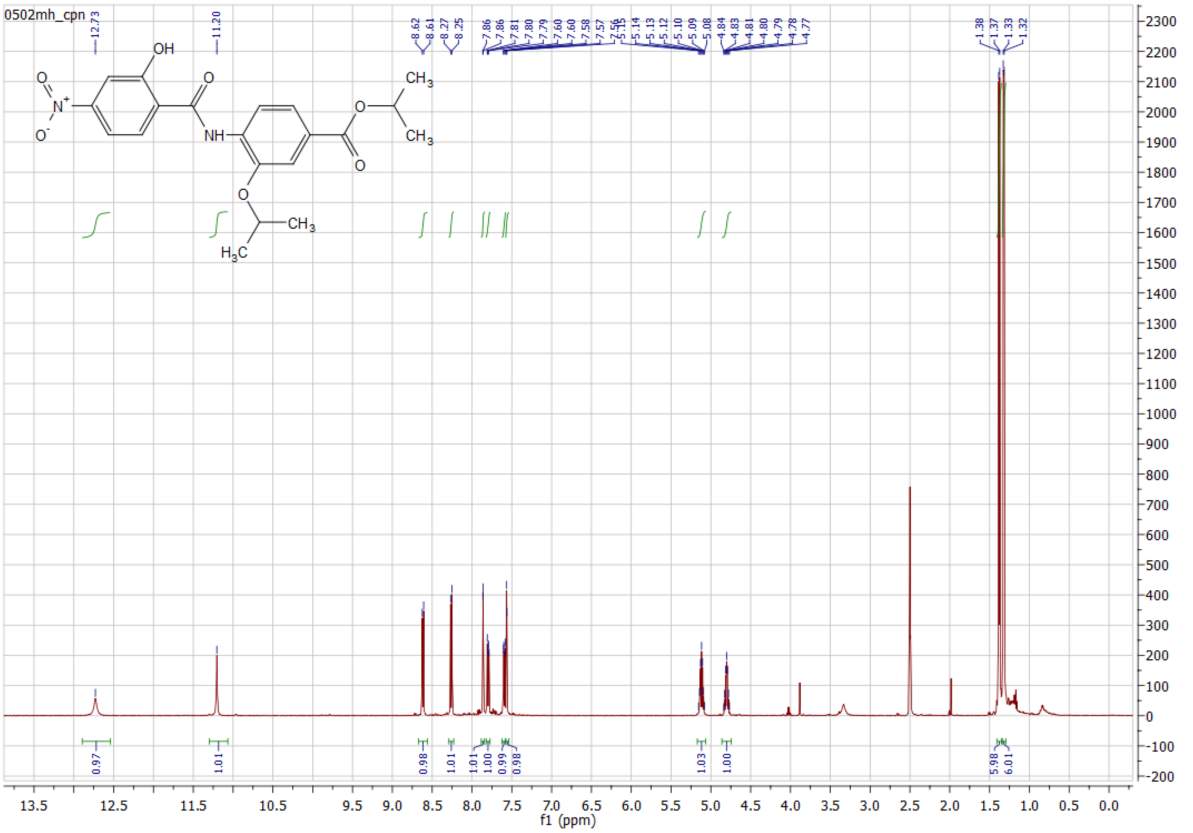

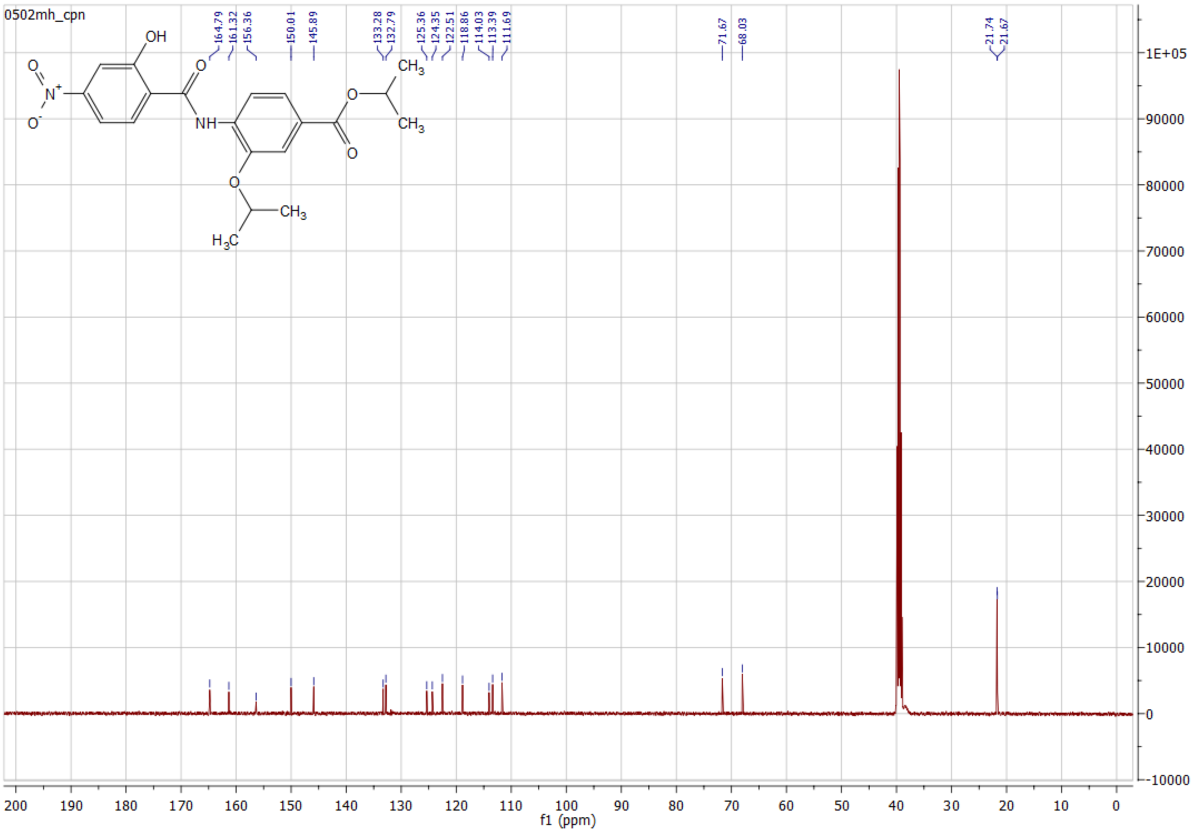

Compound **62**

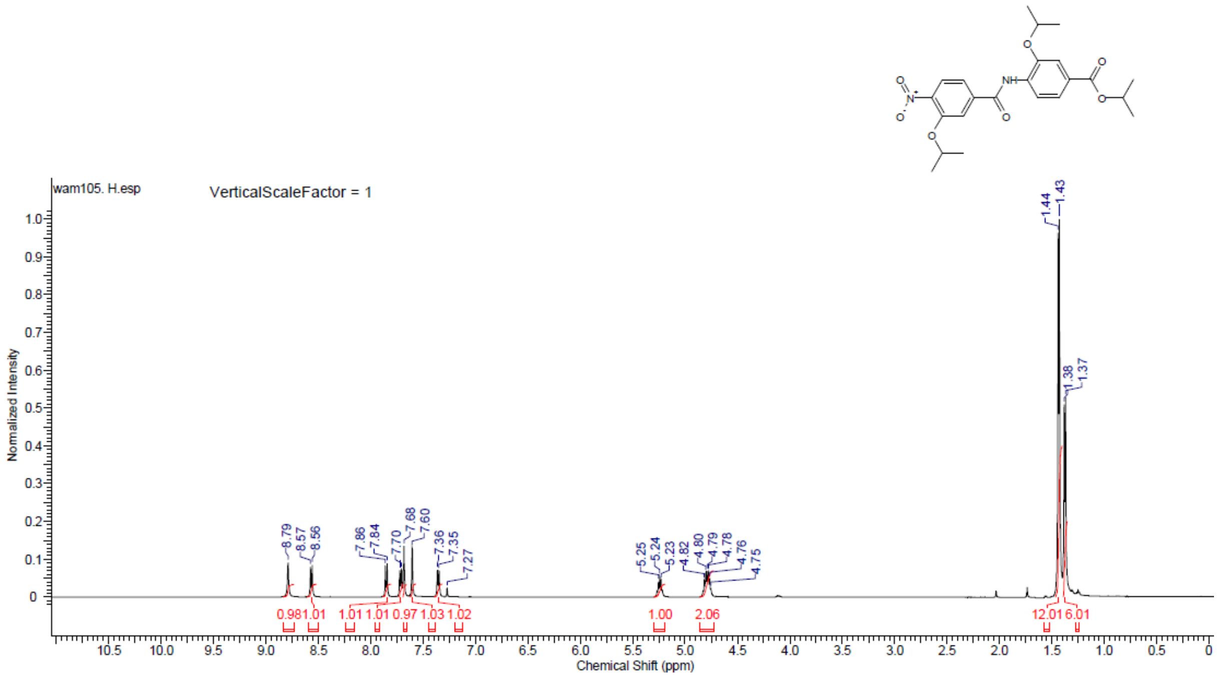

wam105. H.esp
VerticalScaleFactor = 1
8.79 8.57 8.56 7.86 7.84 7.70 7.68 7.60 7.36 7.35 7.27
5.25 5.24 5.23 4.82 4.80 4.79 4.78 4.76 4.75
1.44 1.43 1.38 1.37
0.98 1.01 1.01 1.01 0.97 1.03 1.02 1.00 2.06 12.01 6.01
Normalized Intensity
Chemical Shift (ppm)

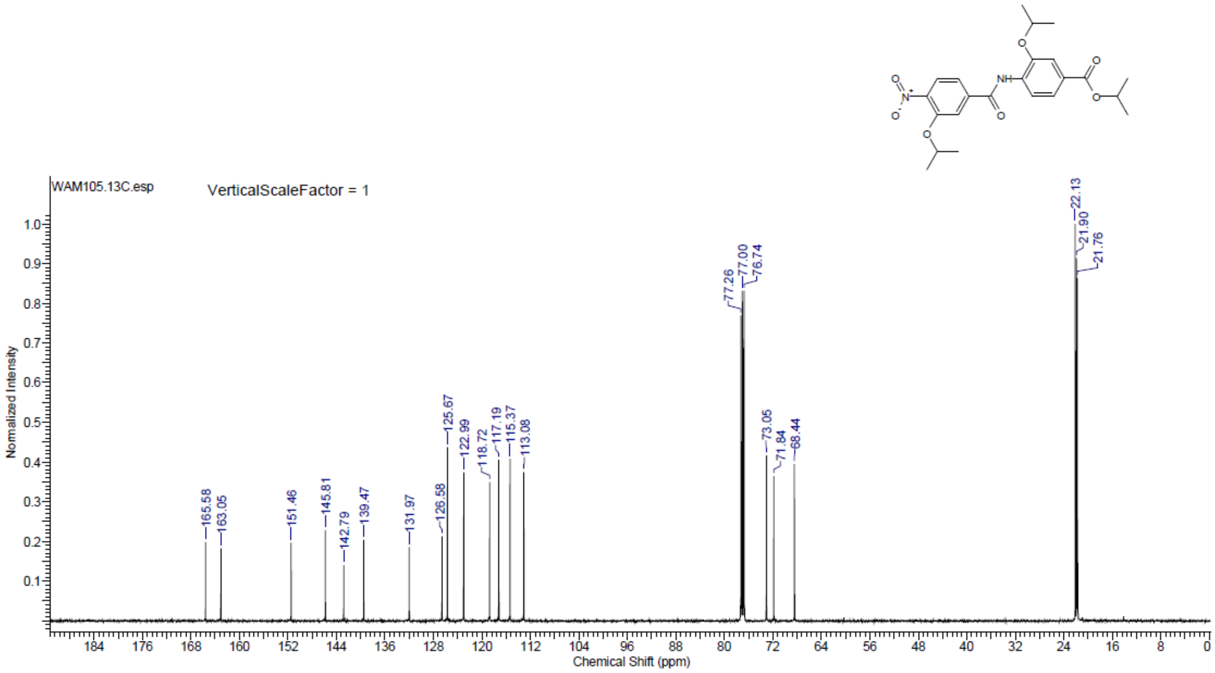

WAM105.13C.esp
VerticalScaleFactor = 1
165.58 163.05 151.46 145.81 142.79 139.47 131.97 126.58 125.67 122.99 118.72 117.19 115.37 113.08
77.26 77.00 76.74 73.05 71.84 68.44
22.13 21.90 21.76
Normalized Intensity
Chemical Shift (ppm)

Compound **24**

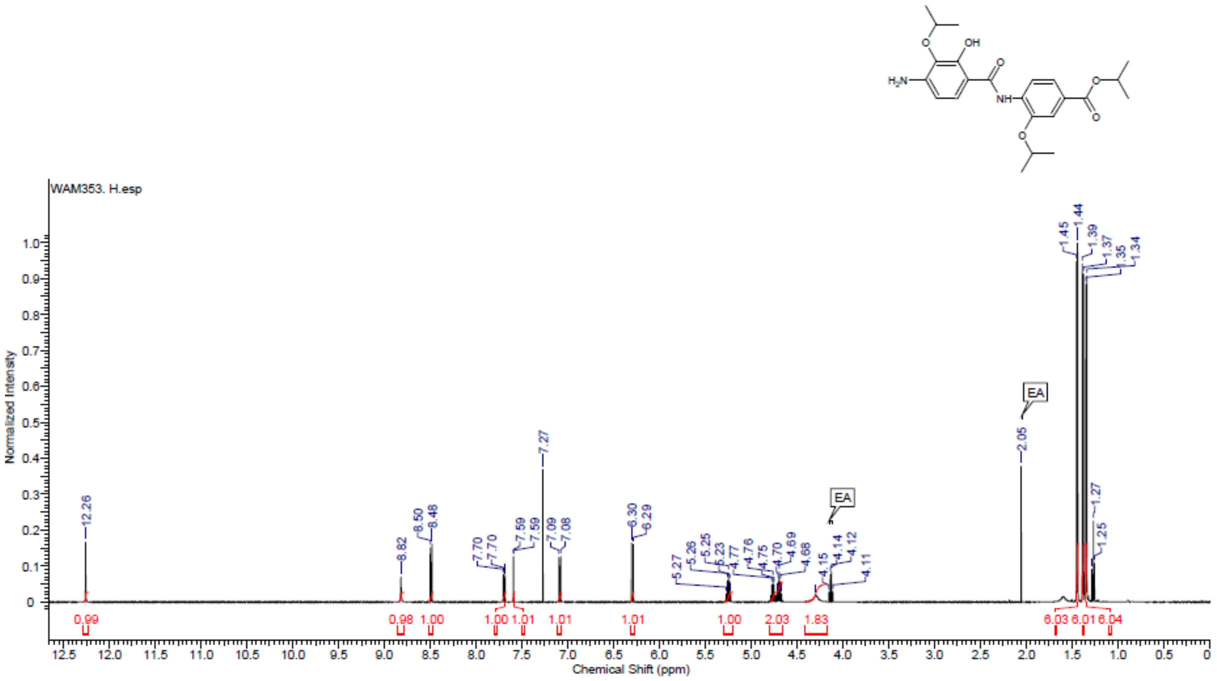

OH
H2N
NH
O
WAM353. H.esp
Normalized Intensity
Chemical Shift (ppm)
EA
12.26
8.82
8.50
8.48
7.70
7.70
7.59
7.59
7.27
7.09
7.08
6.30
6.29
5.27
5.26
5.25
5.23
4.77
4.76
4.75
4.70
4.69
4.68
4.15
4.14
4.12
4.11
2.05
1.45
1.44
1.39
1.37
1.35
1.34
1.27
1.25
0.99
0.98
1.00
1.00
1.01
1.01
1.01
1.00
2.03
1.83
6.03
6.01
6.04

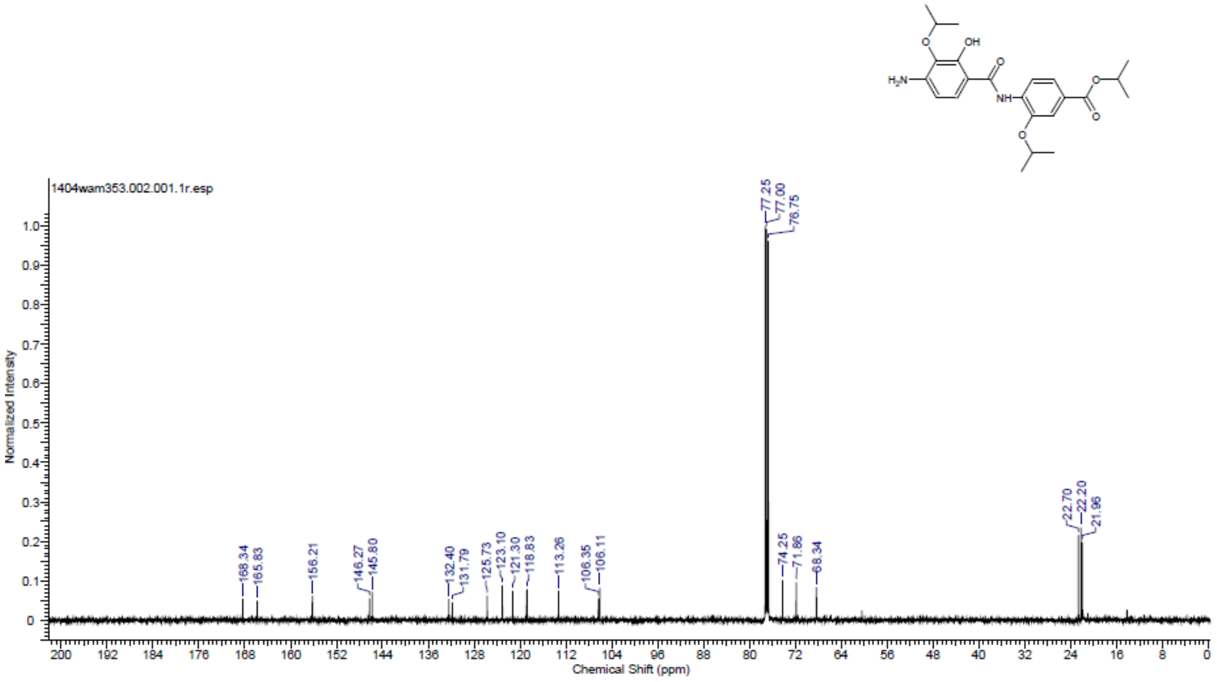

OH
H2N
NH
O
1404wam353.002.001.1r.esp
Normalized Intensity
Chemical Shift (ppm)
168.34
165.83
156.21
146.27
145.80
132.40
131.79
125.73
123.10
121.30
118.83
113.26
106.35
106.11
77.25
77.00
76.75
74.25
71.86
68.34
22.70
22.20
21.96

Compound **63**

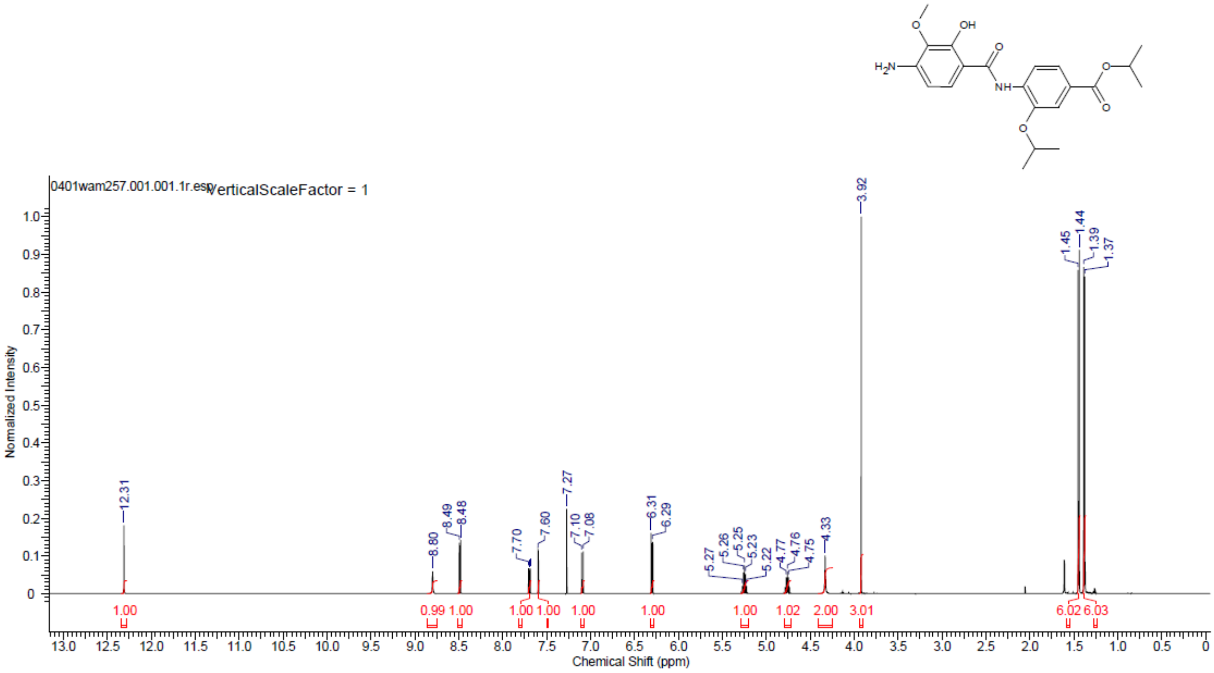
OH
O
H2N
NH
O
O
O
0401wam257.001.001.1r.esp
VerticalScaleFactor = 1
Normalized Intensity
Chemical Shift (ppm)
12.31
8.80
8.49
8.48
7.70
7.60
7.27
7.10
7.08
6.31
6.29
5.27
5.26
5.25
5.23
5.22
4.77
4.76
4.75
4.33
3.92
1.45
1.44
1.39
1.37
1.00
0.99
1.00
1.00
1.00
1.00
1.00
1.00
1.02
2.00
3.01
6.02
6.03

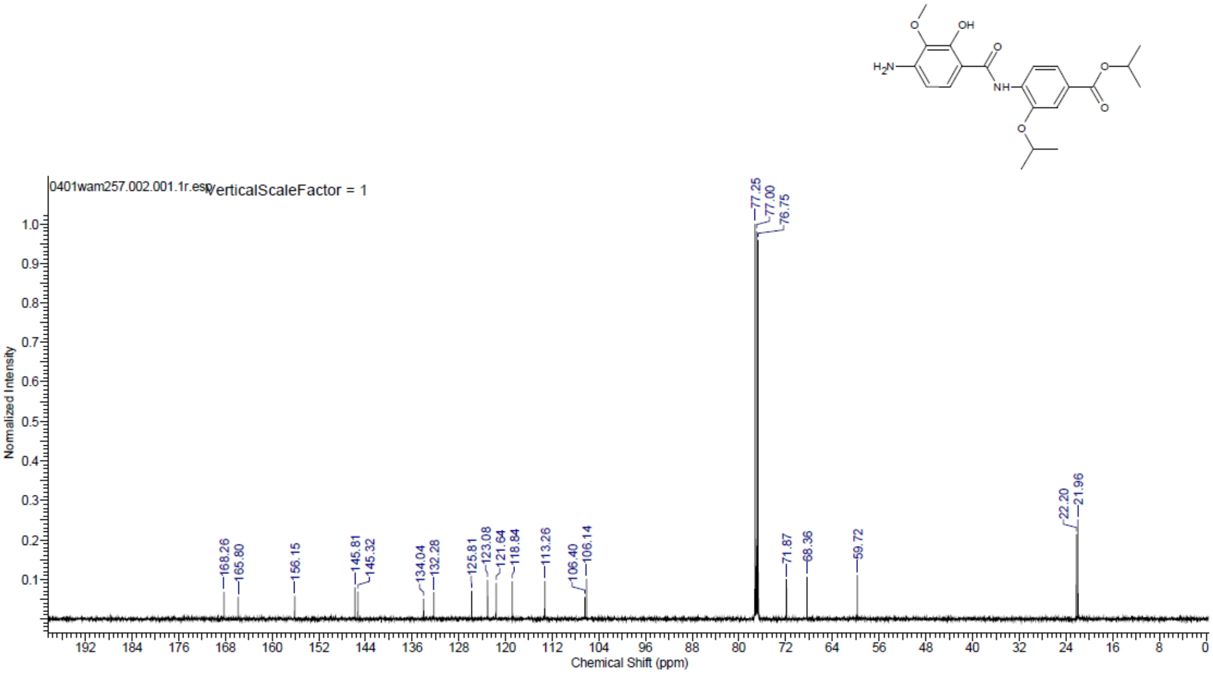
OH
O
H2N
NH
O
O
O
0401wam257.002.001.1r.esp
VerticalScaleFactor = 1
Normalized Intensity
Chemical Shift (ppm)
168.26
165.80
156.15
145.81
145.32
134.04
132.28
125.81
123.08
121.64
118.84
113.26
106.40
106.14
77.25
77.00
76.75
71.87
68.36
59.72
22.20
21.96

Compound **64**

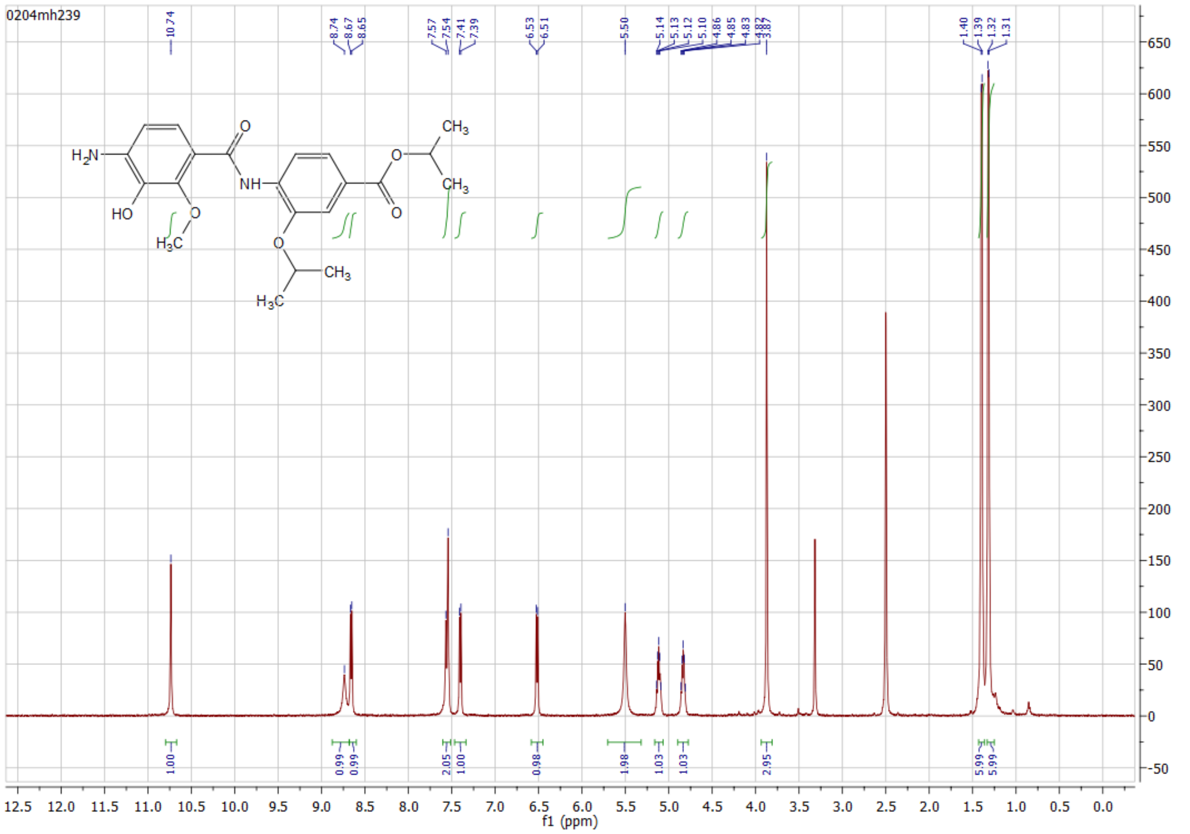
0204mh239
10.74
8.74
8.67
8.65
7.57
7.54
7.41
7.39
6.53
6.51
5.50
5.14
5.13
5.12
5.10
4.86
4.85
4.83
4.82
3.87
1.40
1.39
1.32
1.31
1.00
0.99
0.99
2.05
1.00
0.98
1.98
1.03
1.03
2.95
5.99
5.99
f1 (ppm)

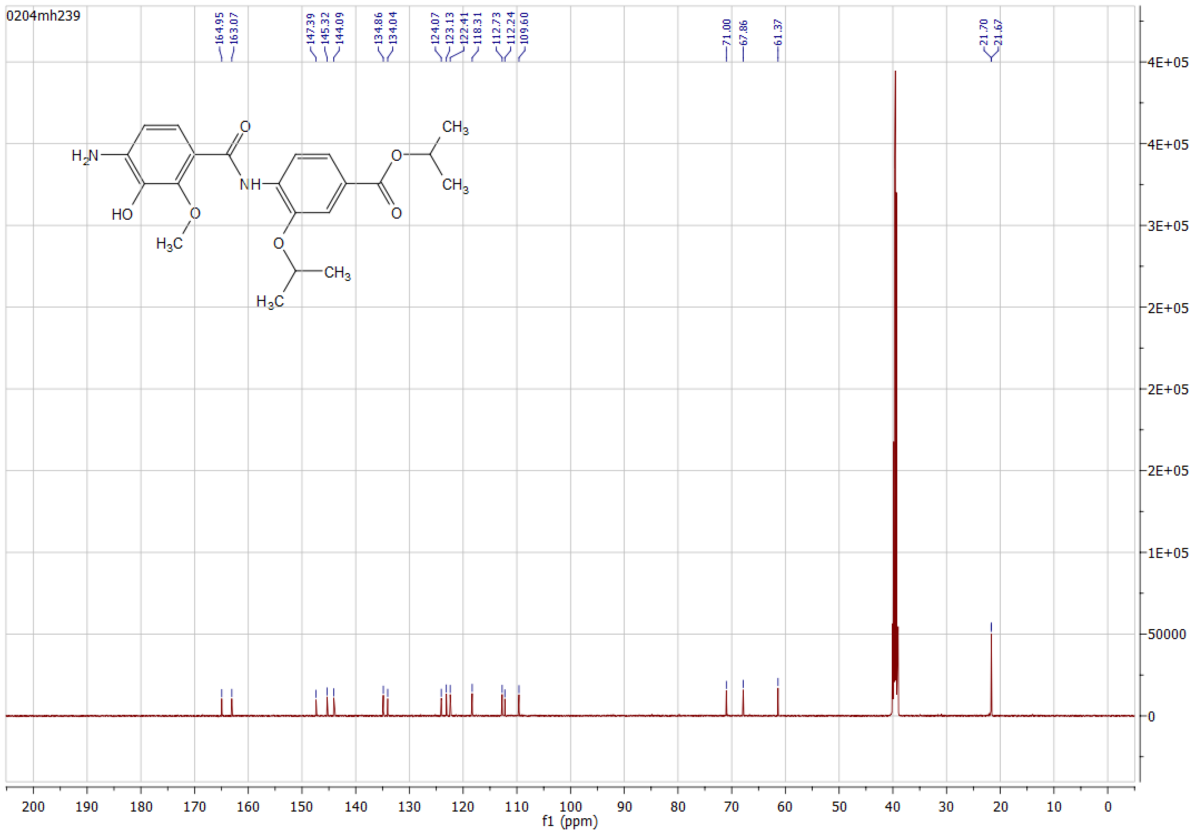
0204mh239
164.95
163.07
147.39
145.32
144.09
134.86
134.04
124.07
123.13
122.41
118.31
112.73
112.24
109.60
71.00
67.86
61.37
21.70
21.67
f1 (ppm)

Compound **65**

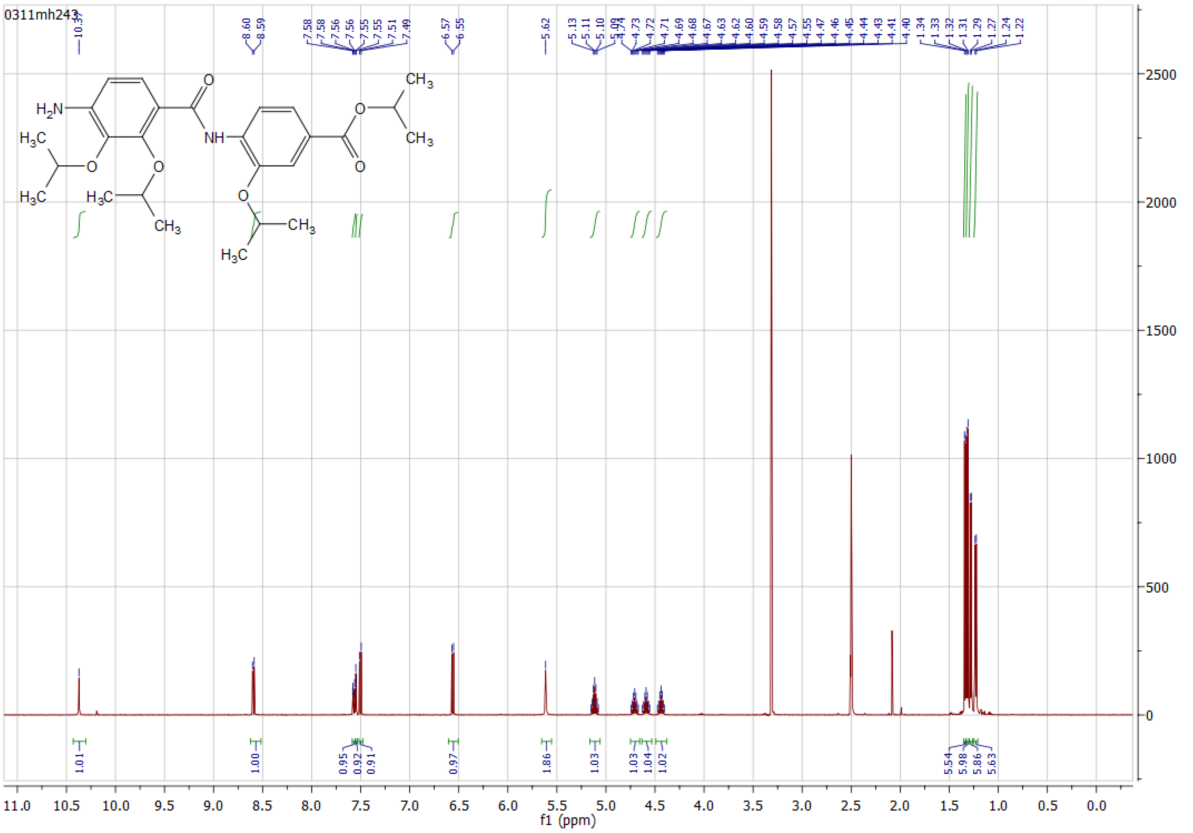

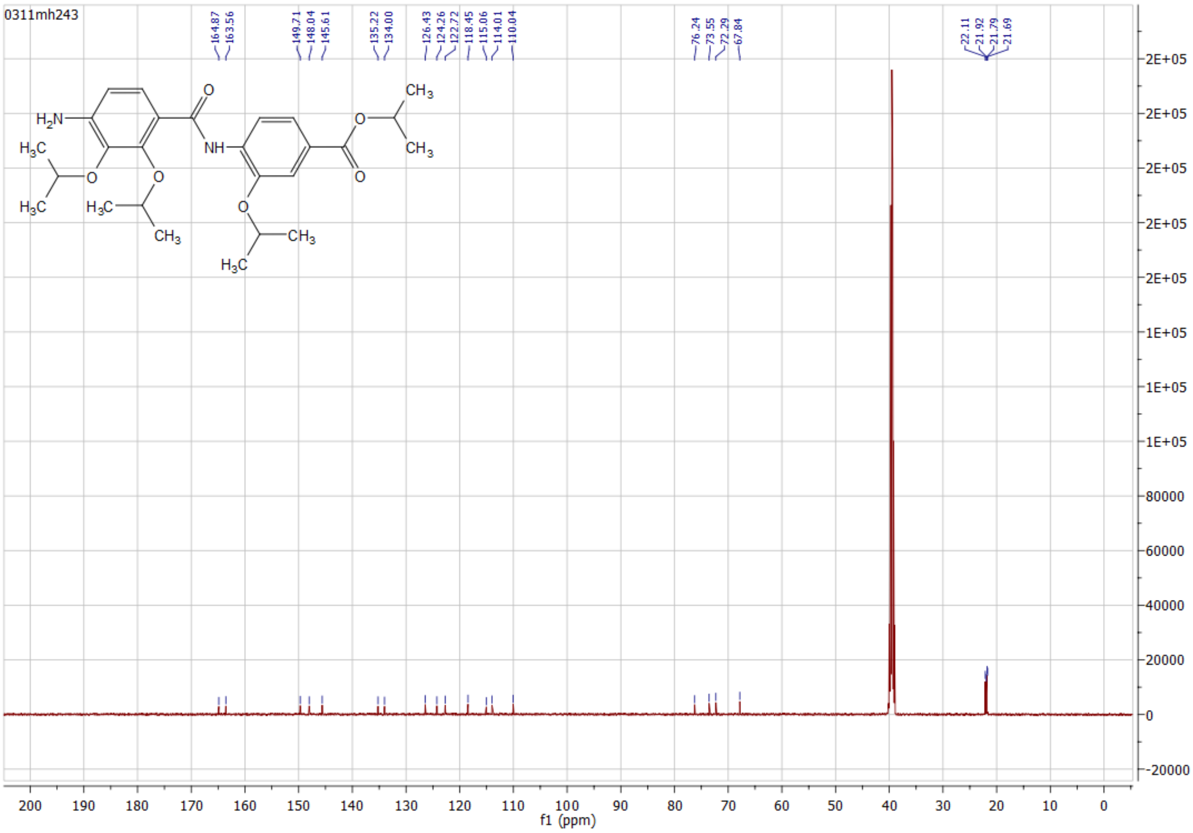

Compound **66**

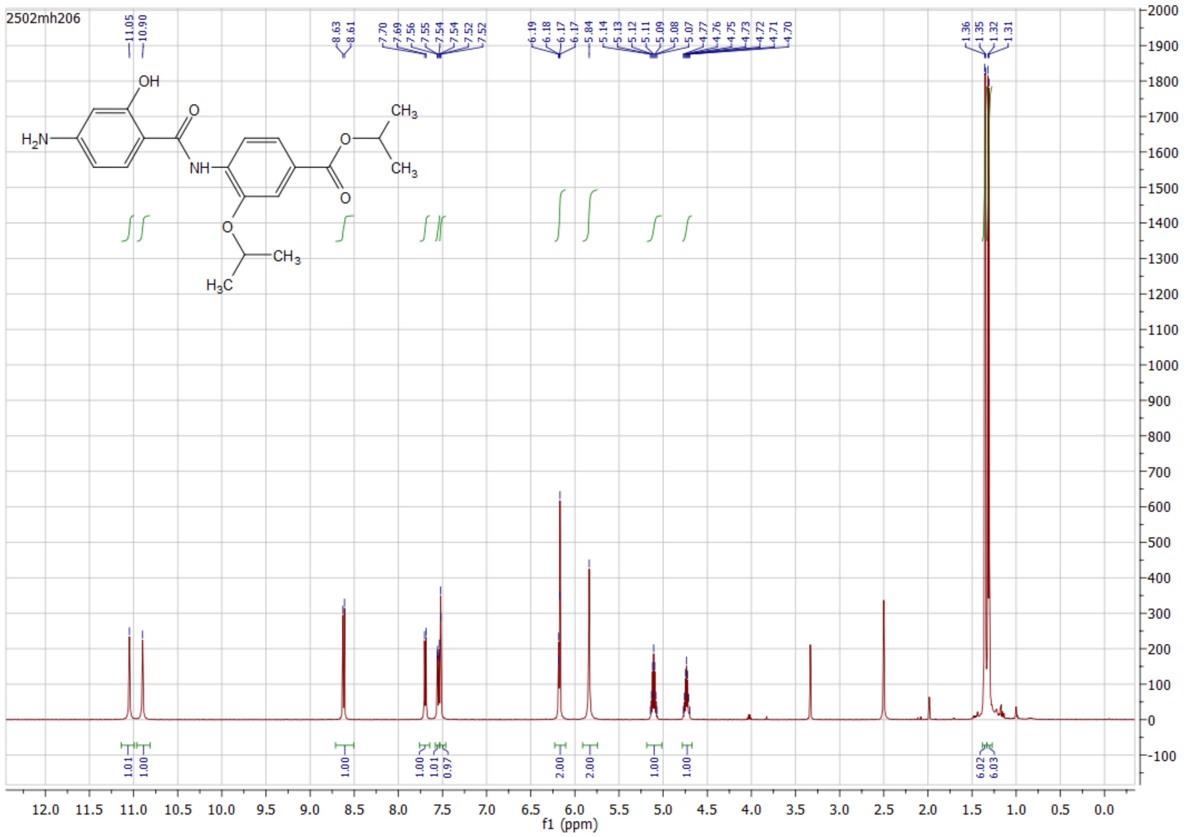

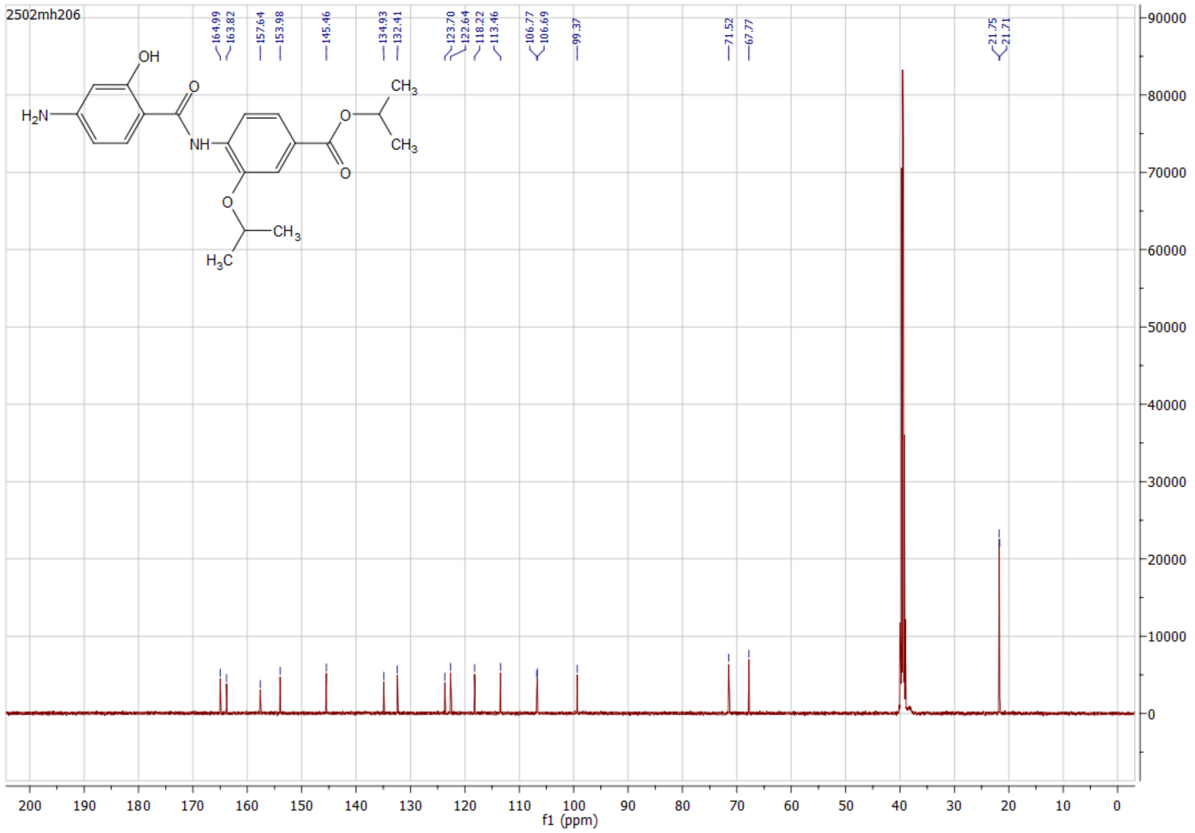

Compound **67**

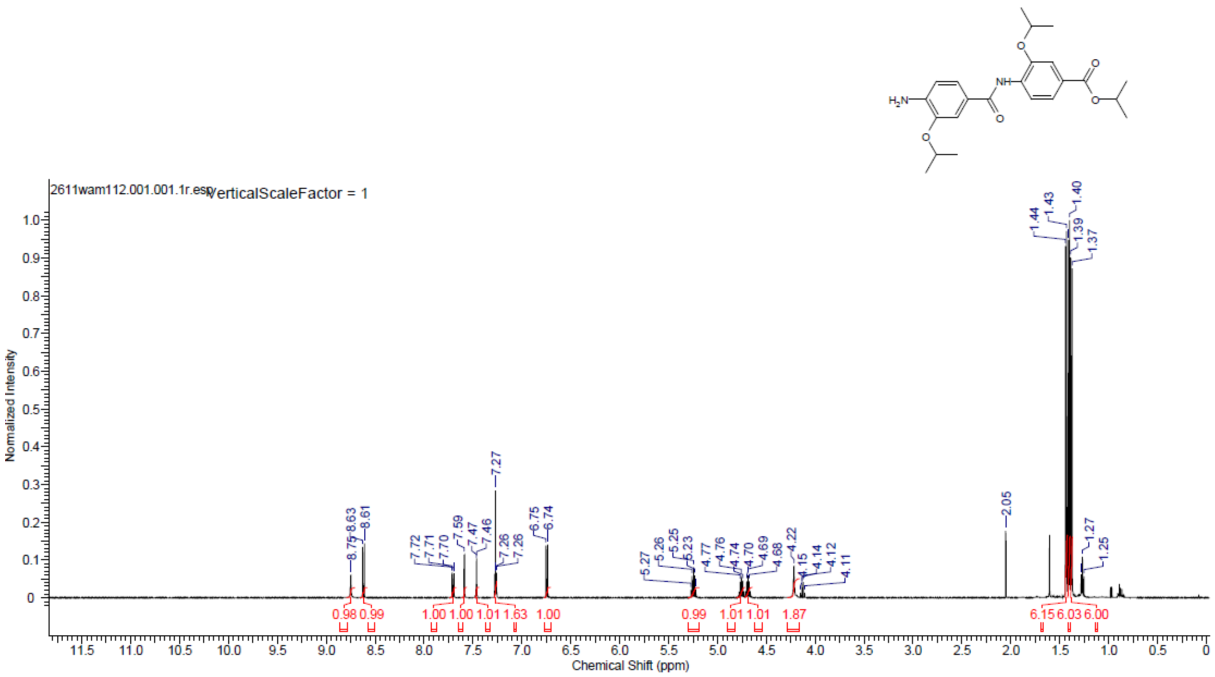

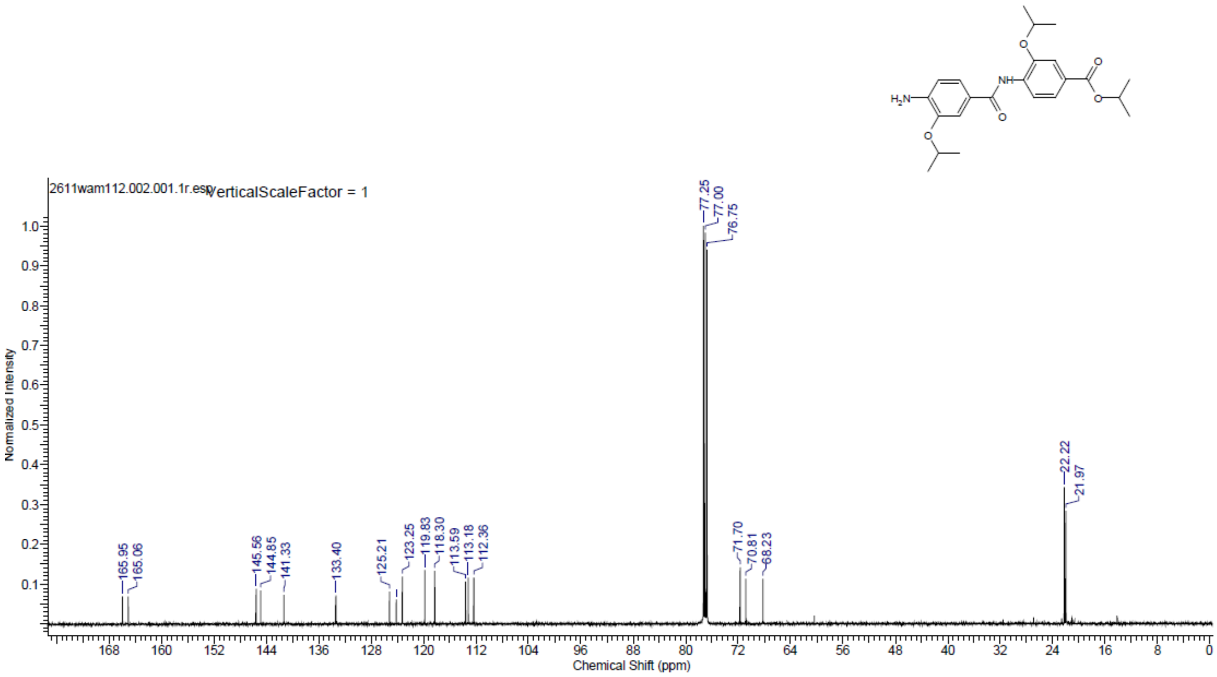

Compound **69**

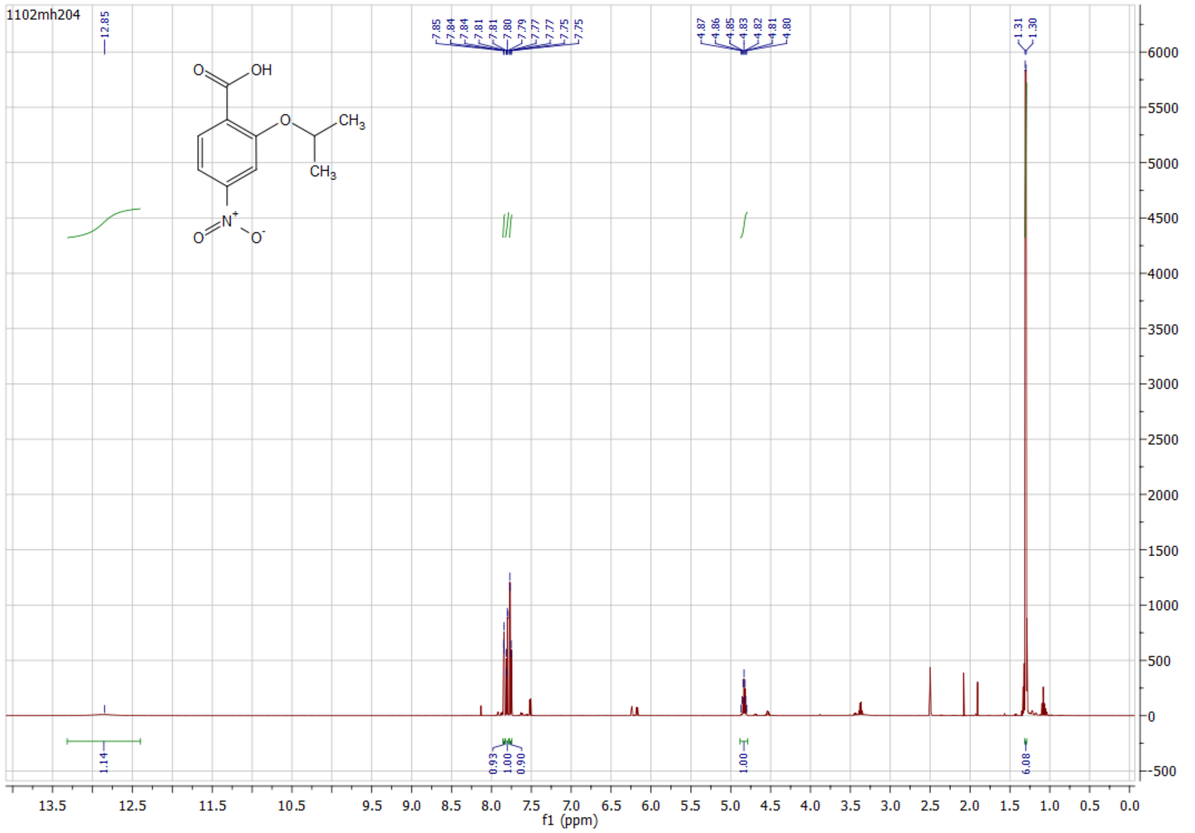
1102mh204
12.85
7.85
7.84
7.84
7.81
7.81
7.80
7.79
7.77
7.77
7.75
7.75
4.87
4.86
4.85
4.83
4.82
4.81
4.80
1.31
1.30
1.14
0.93
1.00
0.90
1.00
6.08
f1 (ppm)

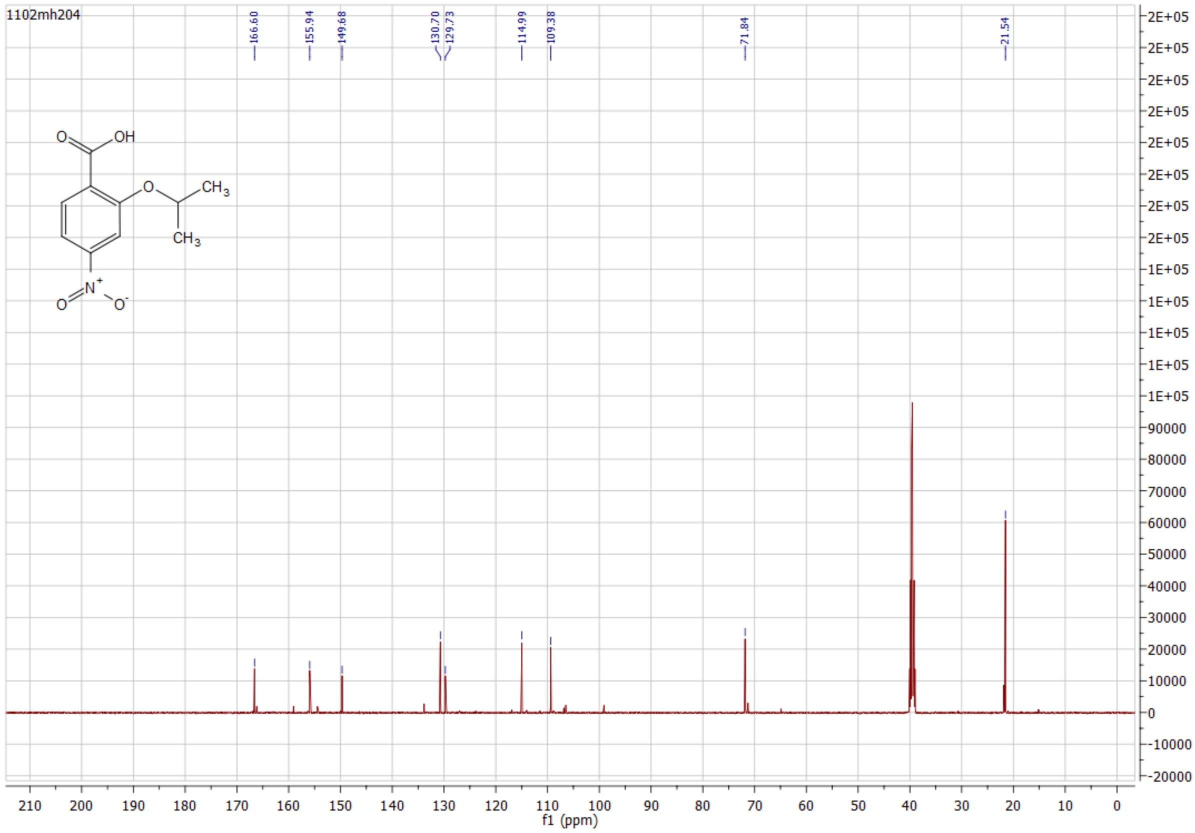
1102mh204
166.60
155.94
149.68
130.70
129.73
114.99
109.38
71.84
21.54
f1 (ppm)

Compound **25**

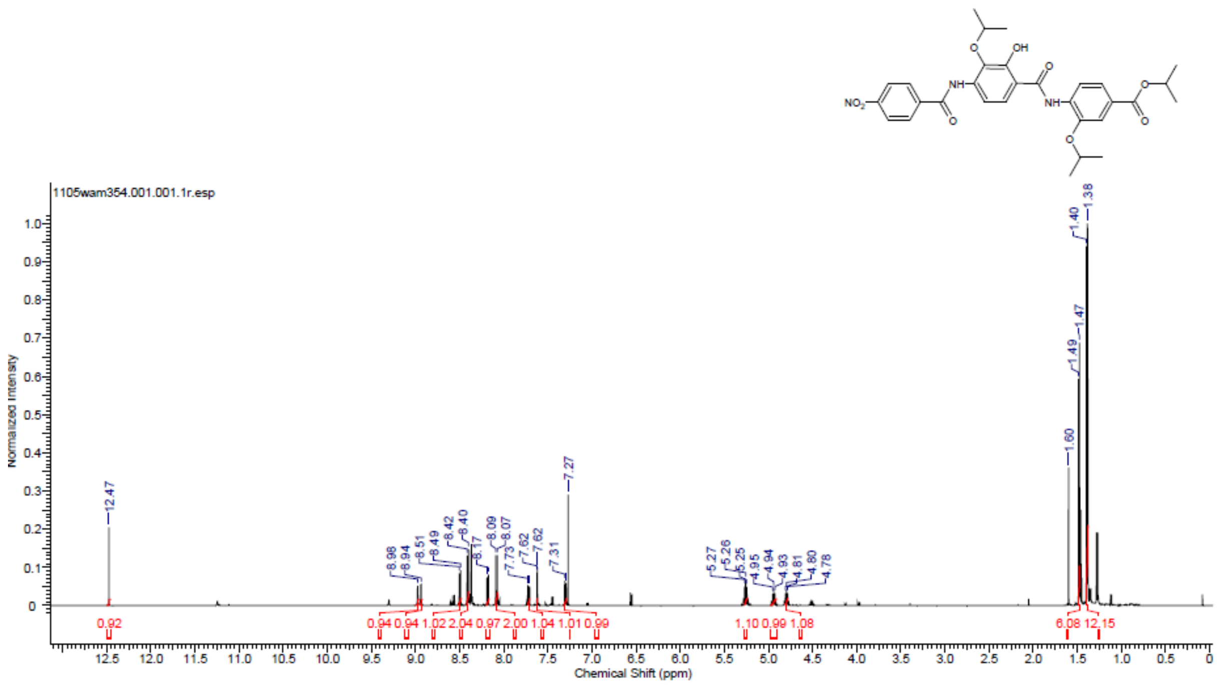

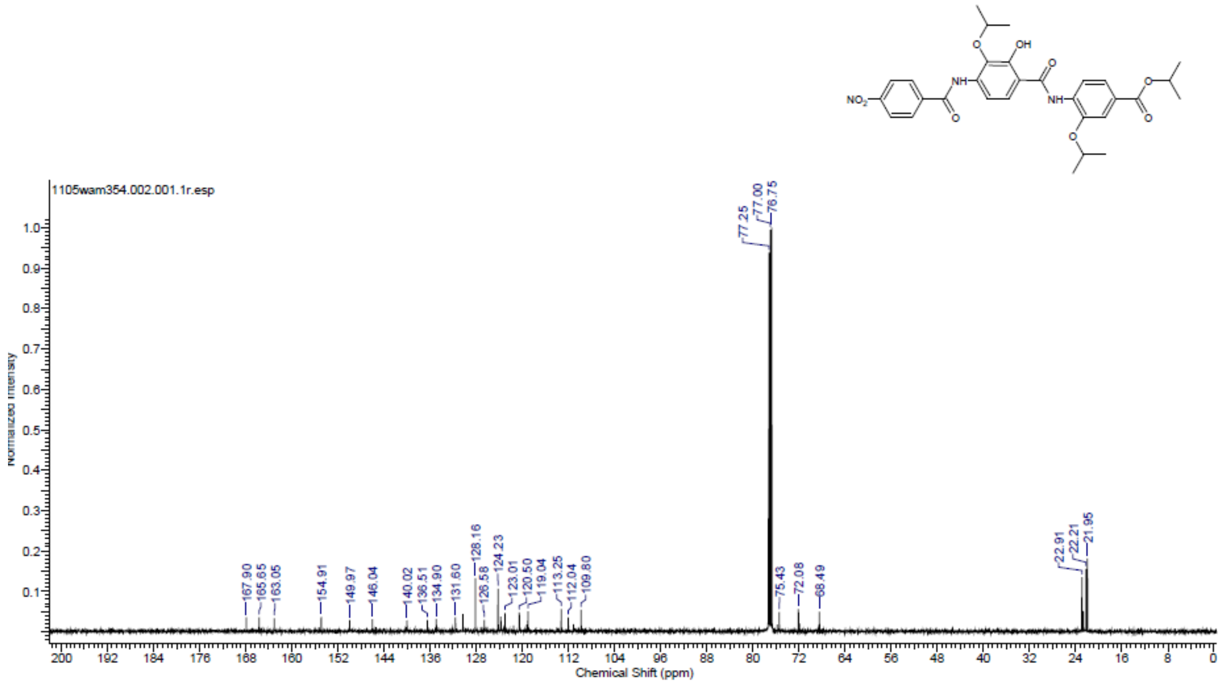

Compound **70** ($CDCl_3$)

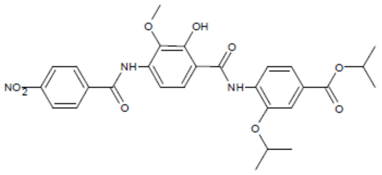

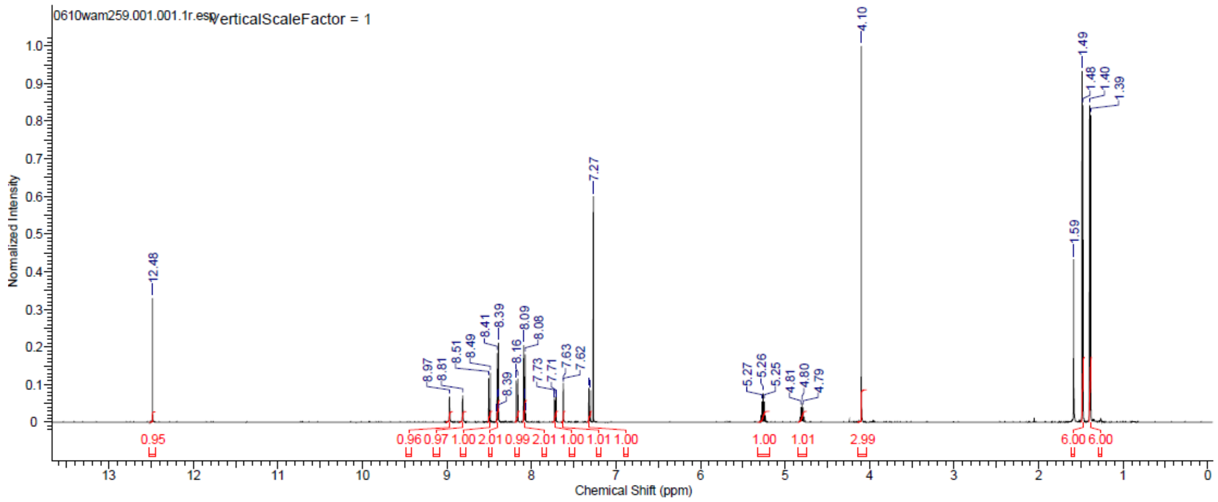

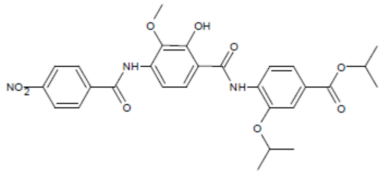

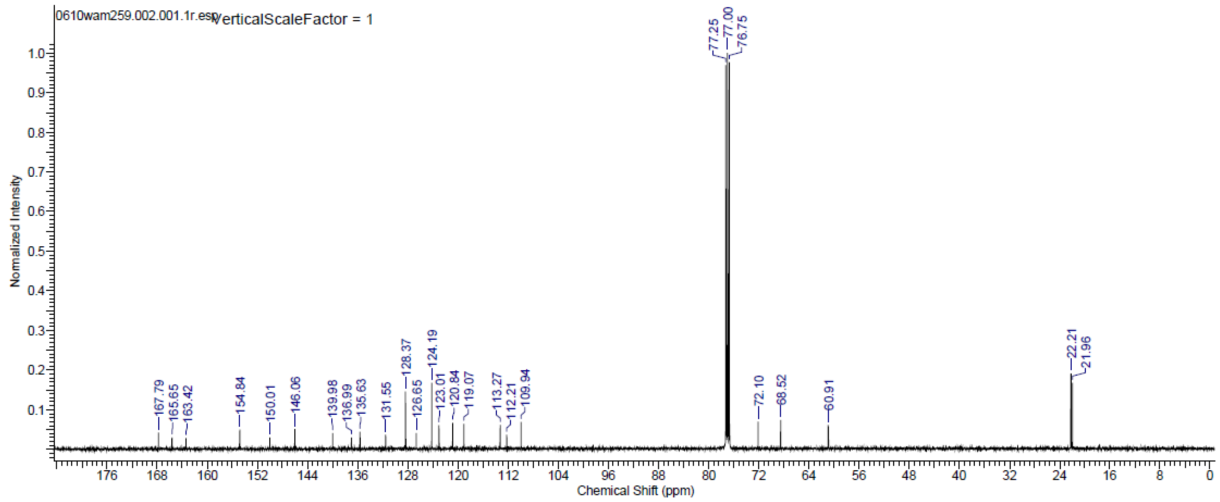

Compound **70** (DMSO-d_6)

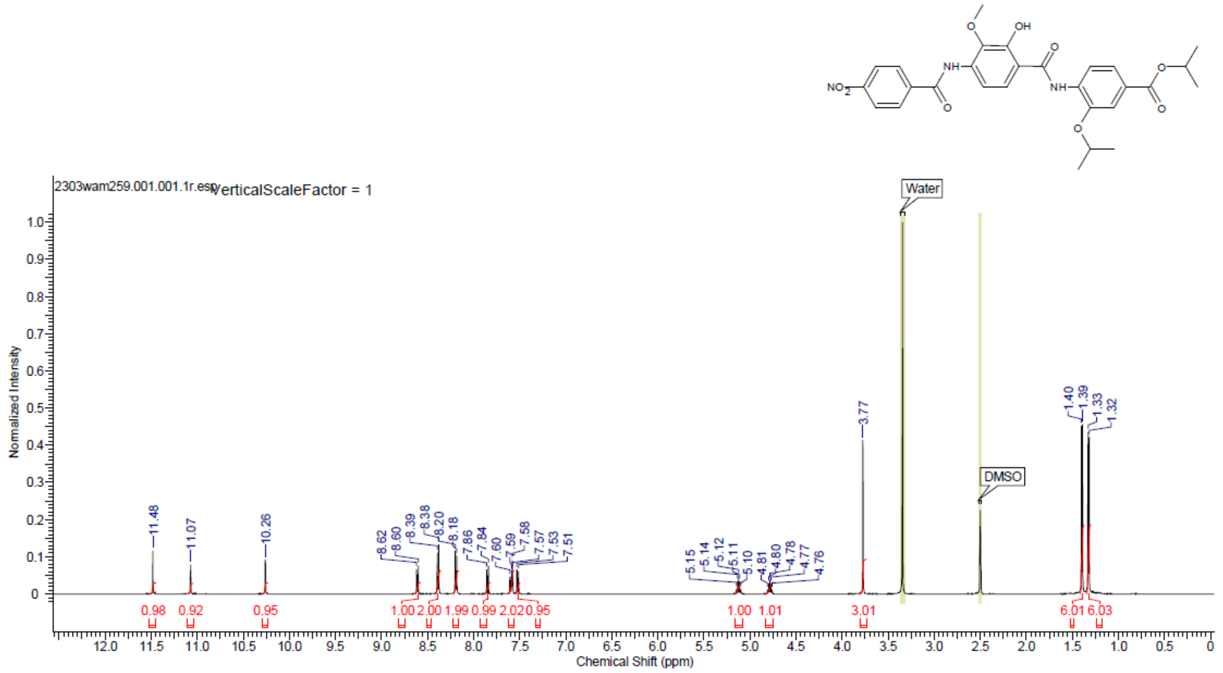

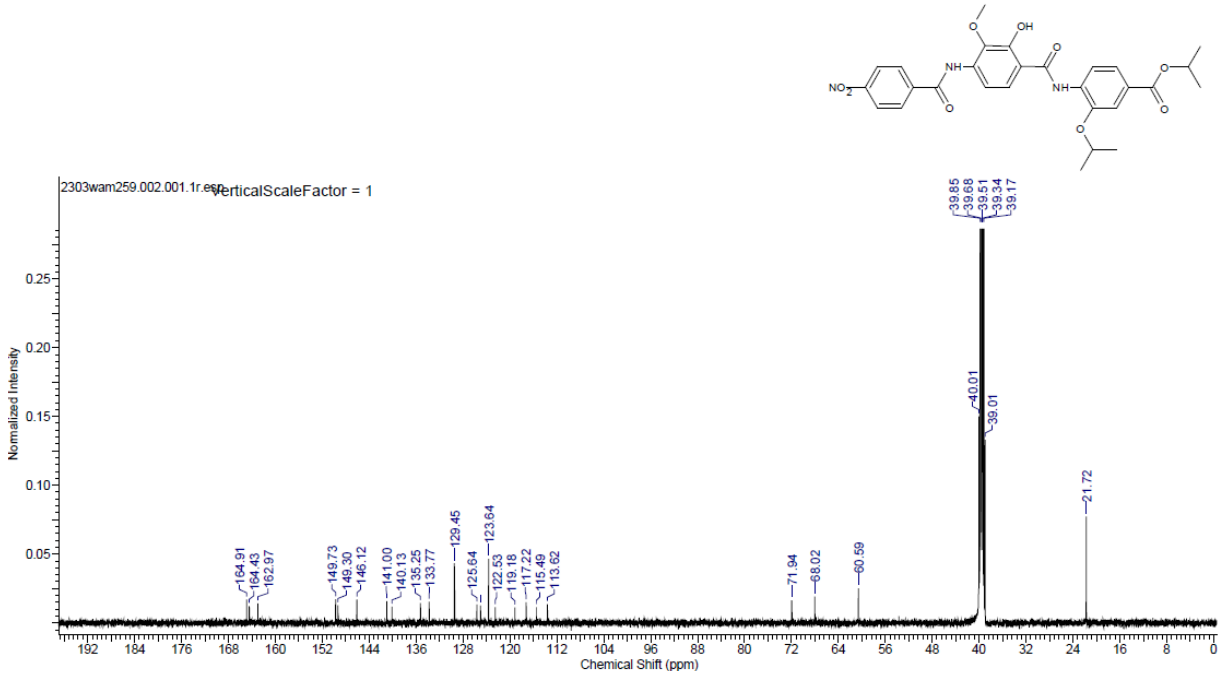

Compound **71**

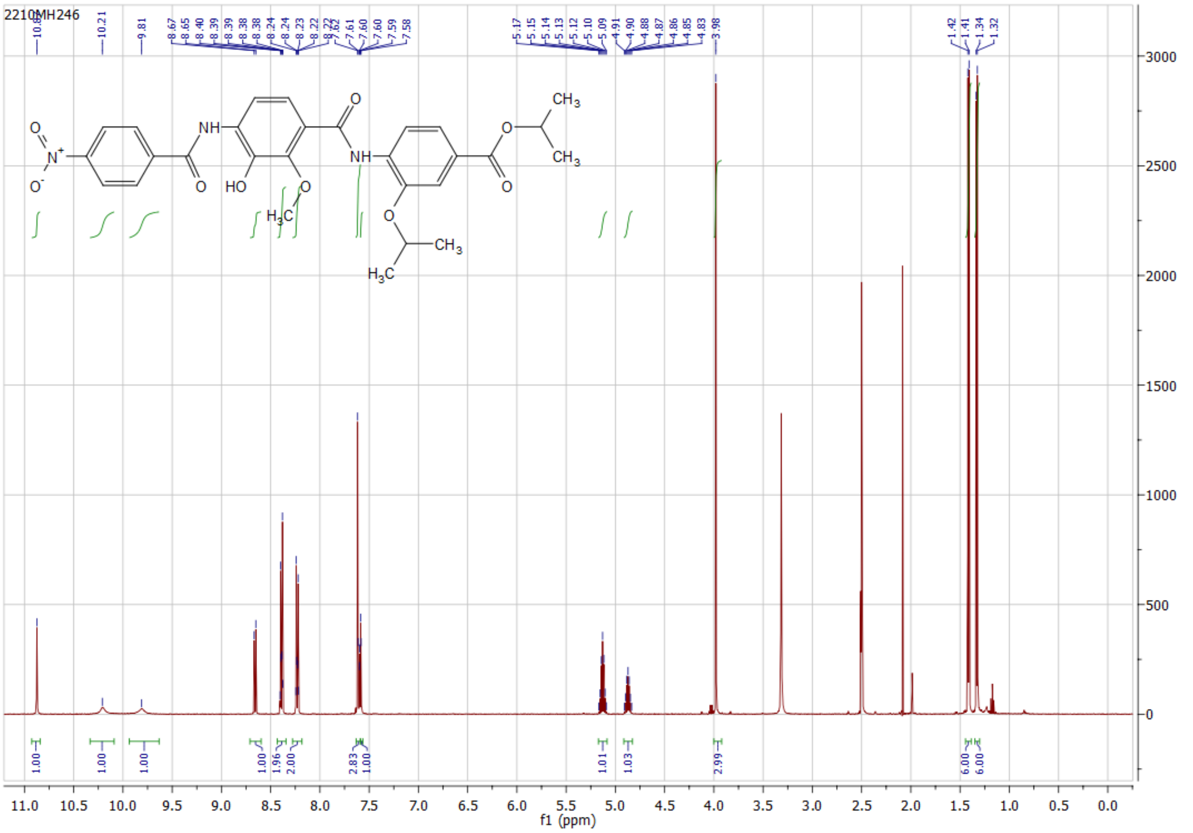

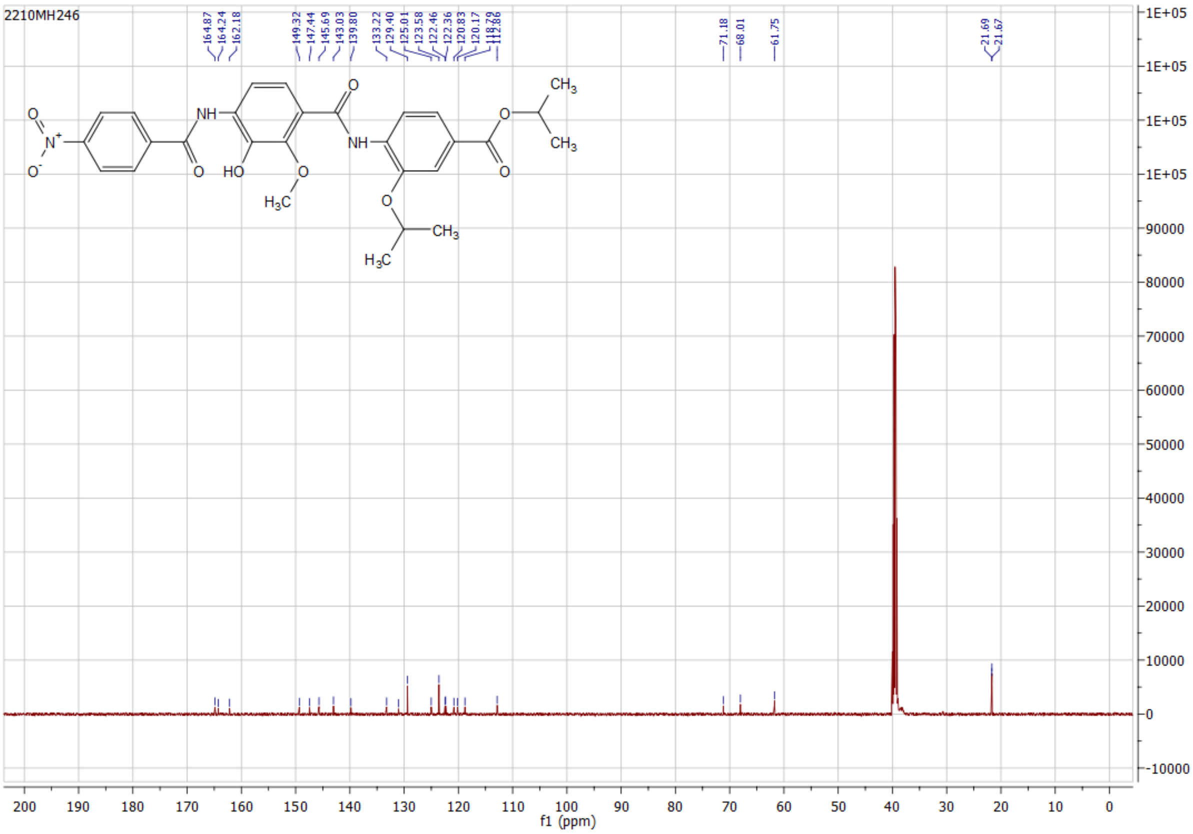

Compound **72**

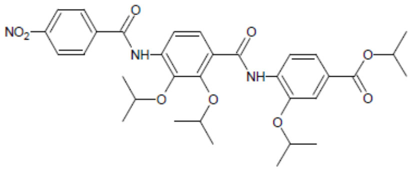

NO_2
NH
NH
O
O
O
O
O
O

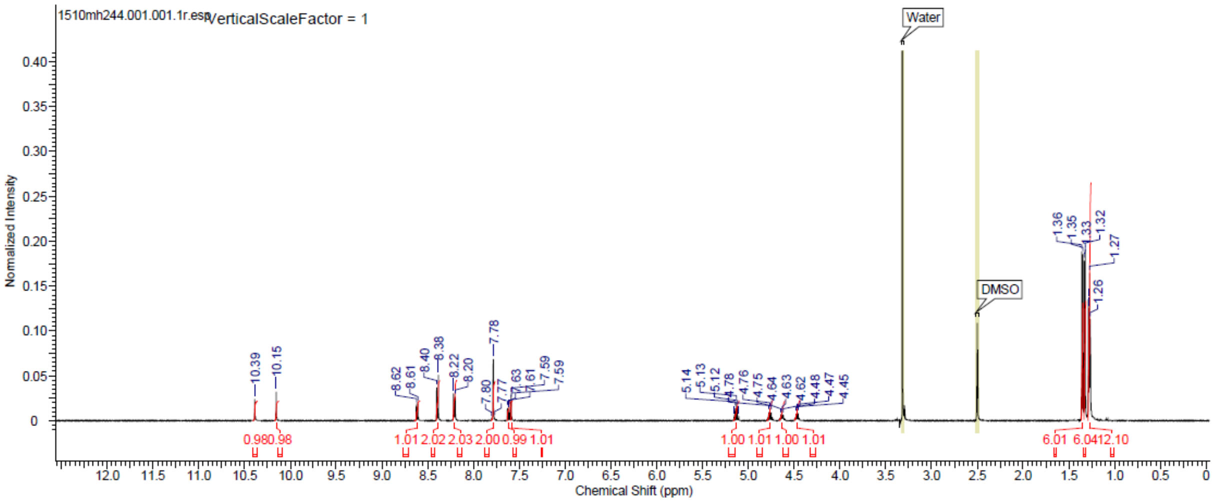

1510mh244.001.001.1r.esp
VerticalScaleFactor = 1
Water
DMSO
Normalized Intensity
Chemical Shift (ppm)
10.39
10.15
8.62
8.61
8.40
8.38
8.22
8.20
7.80
7.78
7.77
7.63
7.61
7.59
7.59
5.14
5.13
5.12
4.78
4.76
4.75
4.64
4.63
4.62
4.48
4.47
4.45
1.36
1.35
1.33
1.32
1.27
1.26
0.98
0.98
1.01
2.02
2.03
2.00
0.99
1.01
1.00
1.01
1.00
1.01
6.01
6.04
12.10

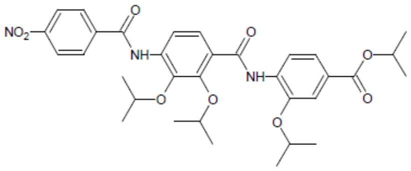

NO_2
NH
NH
O
O
O
O
O
O

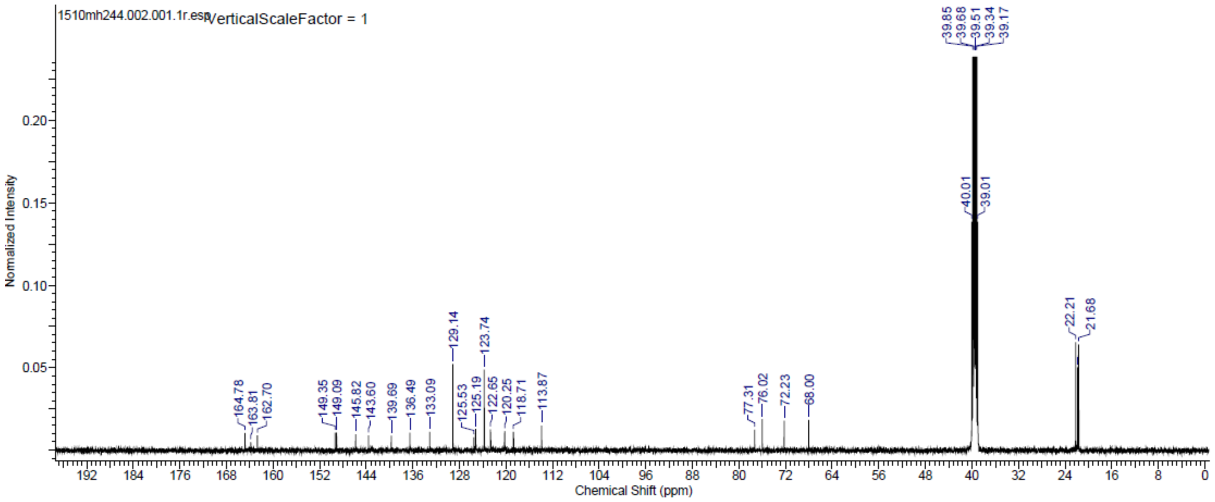

1510mh244.002.001.1r.esp
VerticalScaleFactor = 1
Normalized Intensity
Chemical Shift (ppm)
164.78
163.81
162.70
149.35
149.09
145.82
143.60
139.69
136.49
133.09
129.14
125.53
125.19
123.74
122.65
120.25
118.71
113.87
77.31
76.02
72.23
68.00
39.85
39.68
39.51
39.34
39.17
40.01
39.01
22.21
21.68

Compound **73**

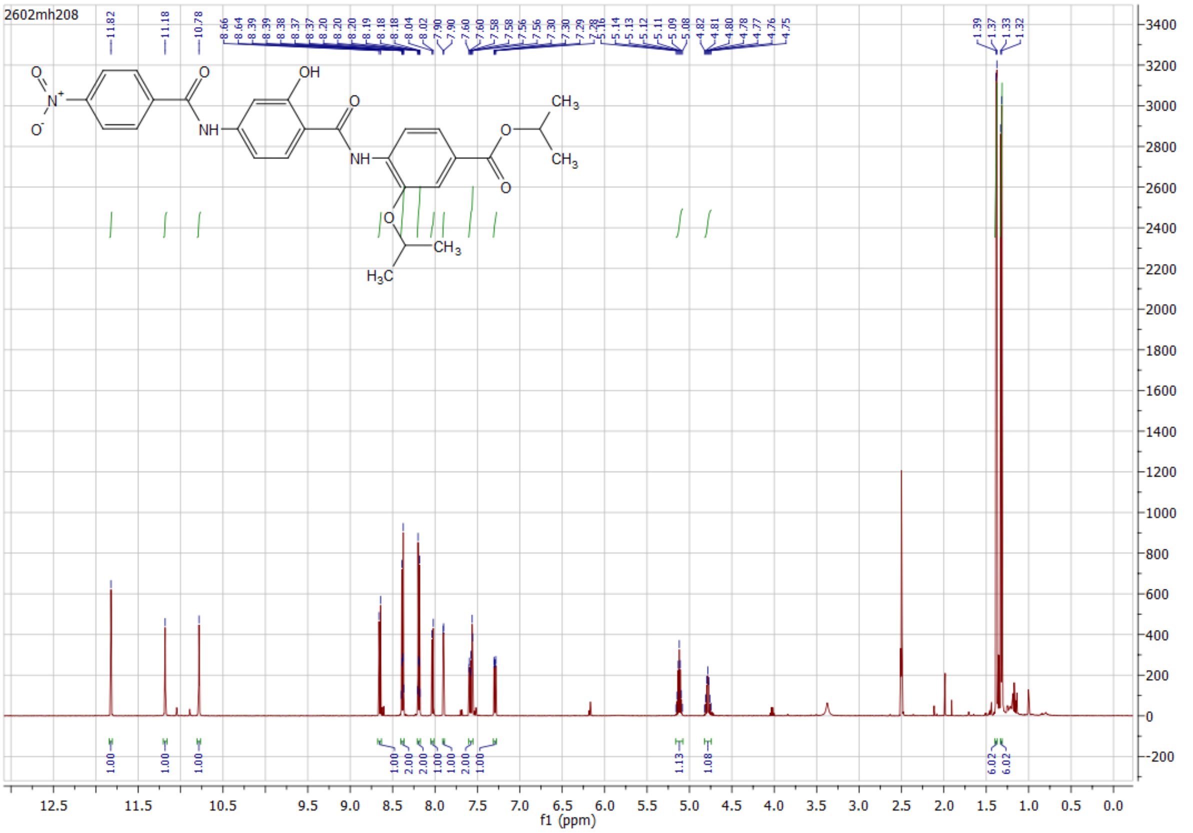

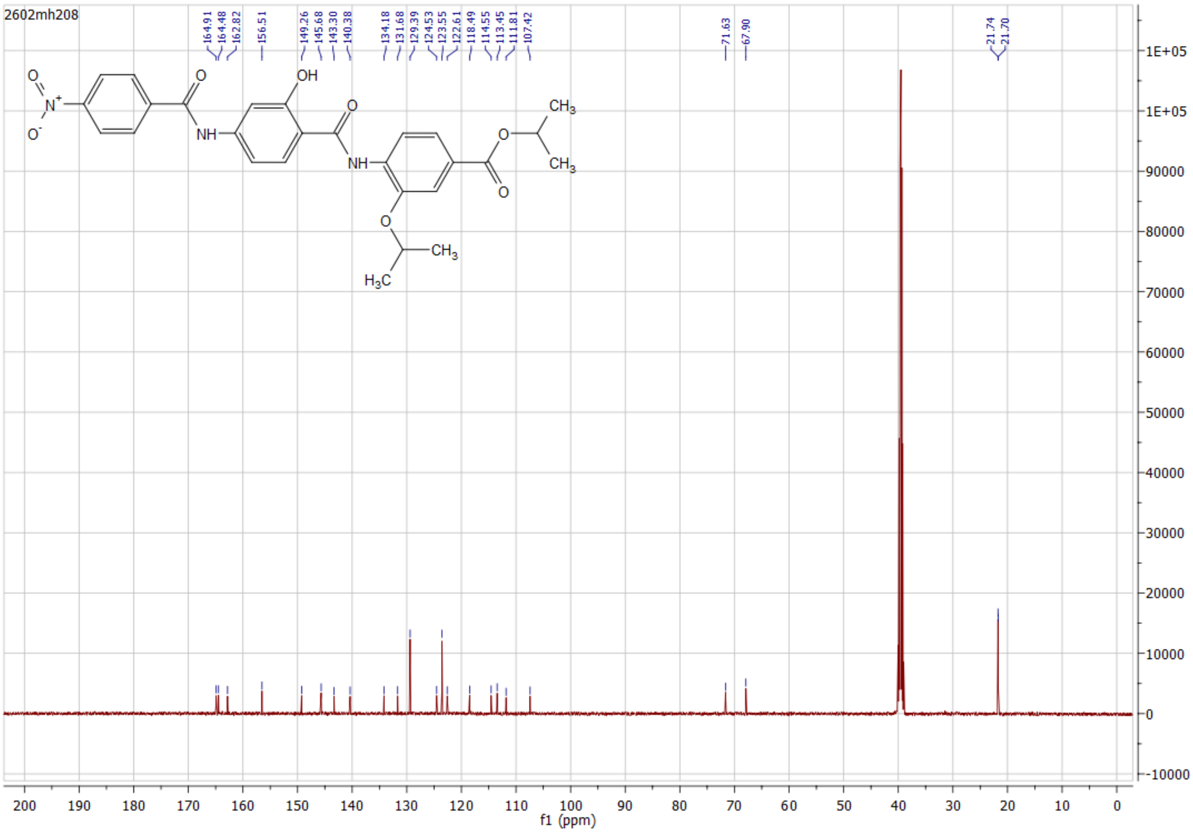

Compound **74**

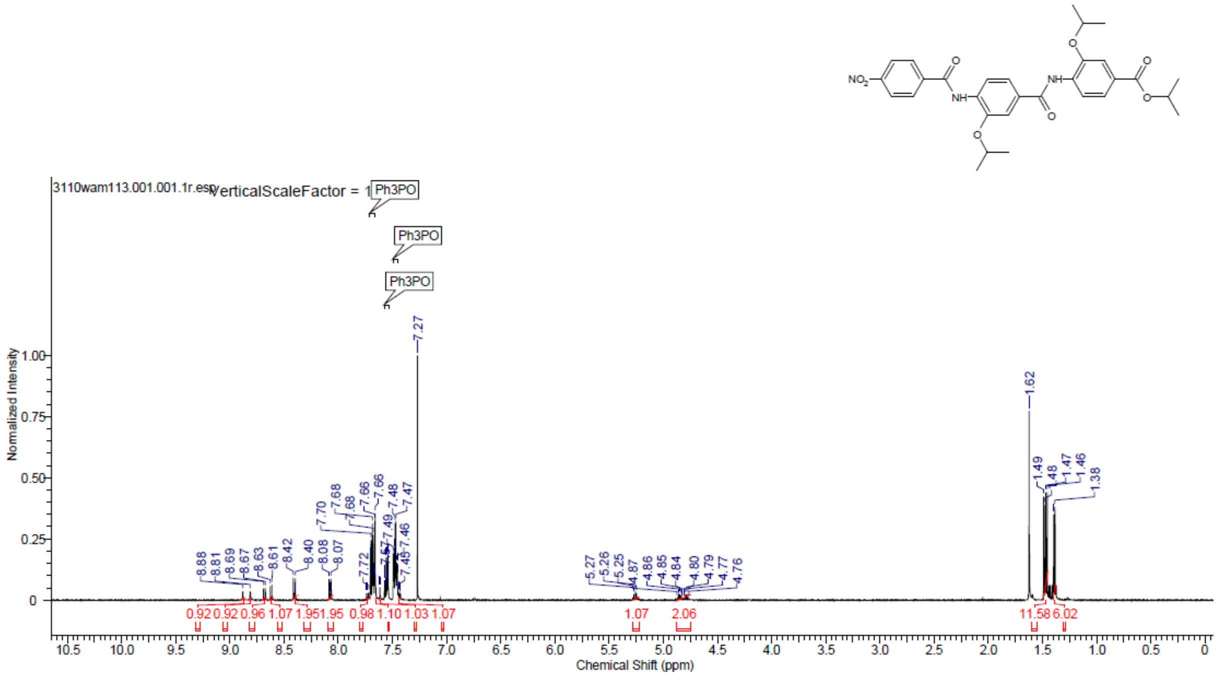
NO2
NH
NH
O
3110wam113.001.001.1r.esp VerticalScaleFactor = 1
Ph3PO
Ph3PO
Ph3PO
Normalized Intensity
Chemical Shift (ppm)
7.27
1.62

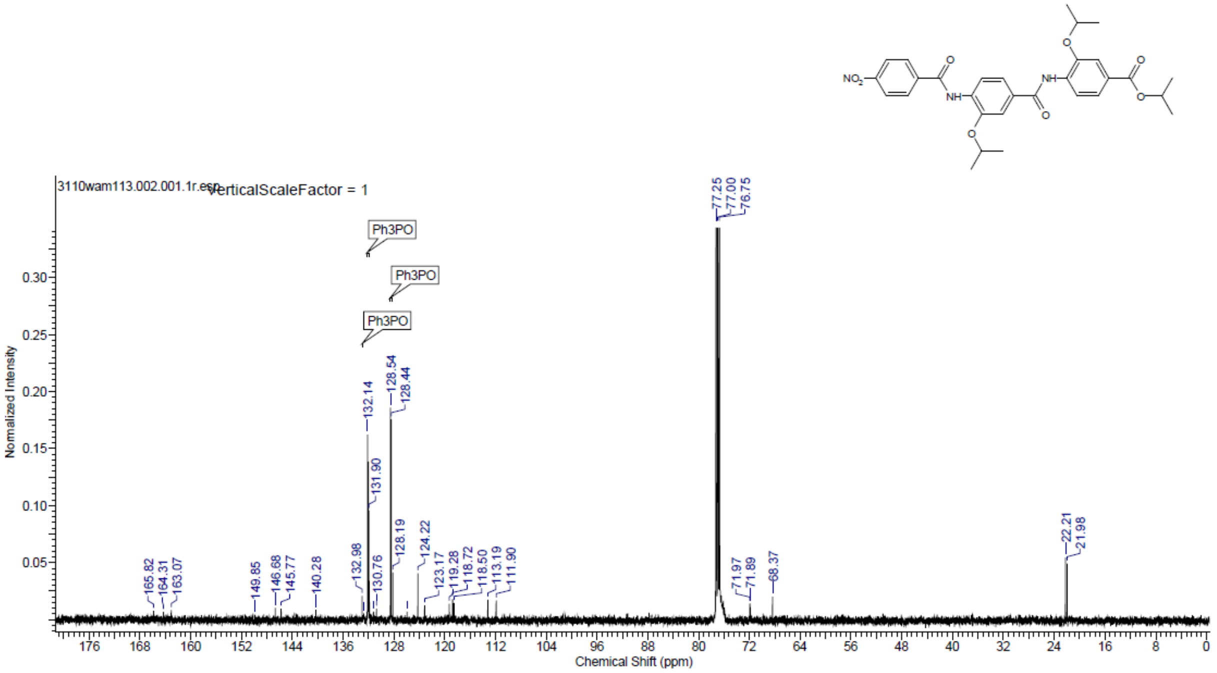
NO2
NH
NH
O
3110wam113.002.001.1r.esp VerticalScaleFactor = 1
Ph3PO
Ph3PO
Ph3PO
Normalized Intensity
Chemical Shift (ppm)
77.25
77.00
76.75
132.14
128.54
128.44
22.21
21.98

Compound **75**

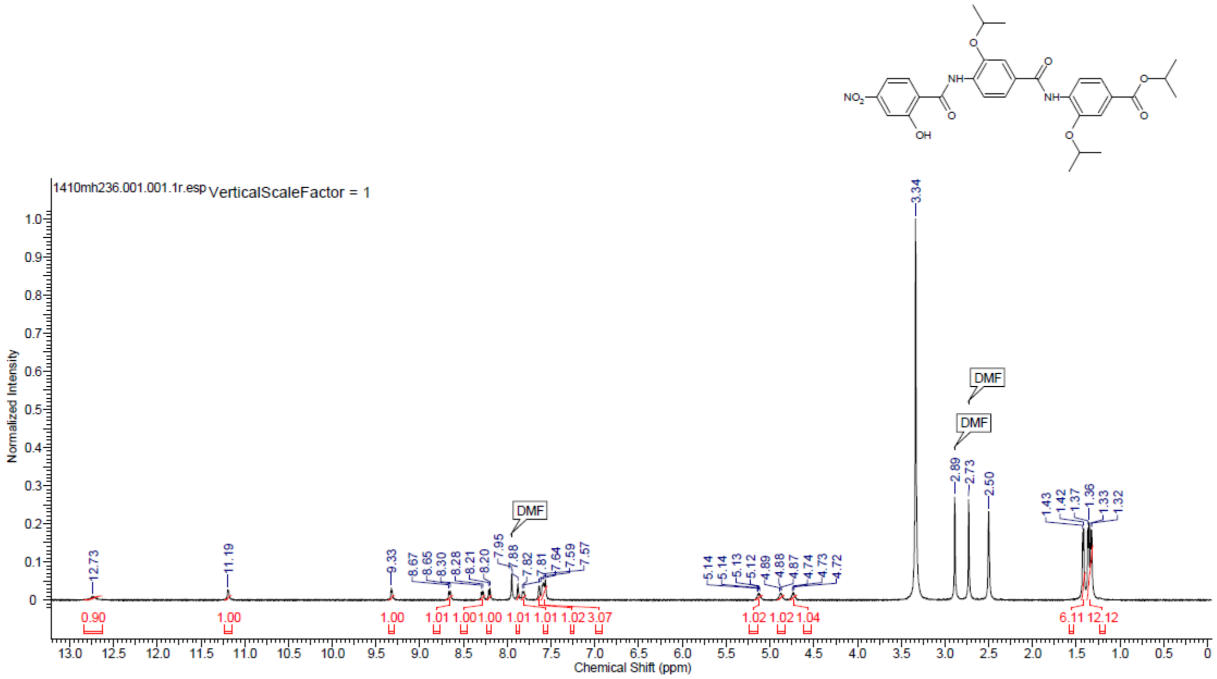

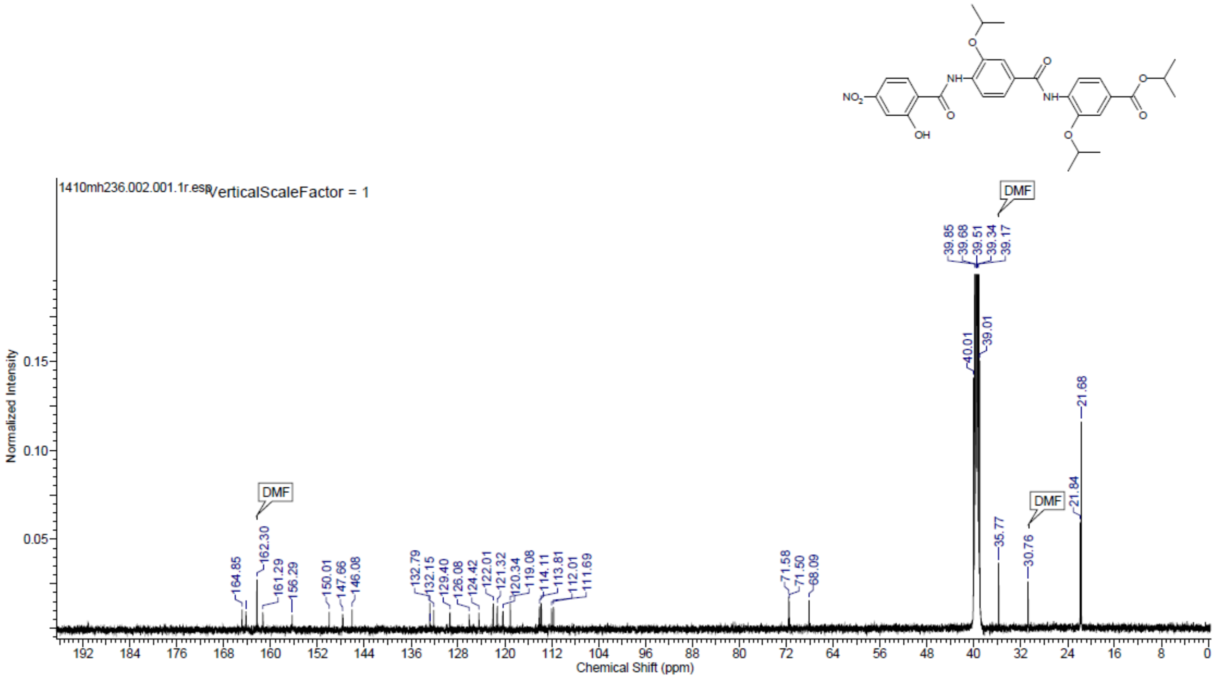

Compound **76**

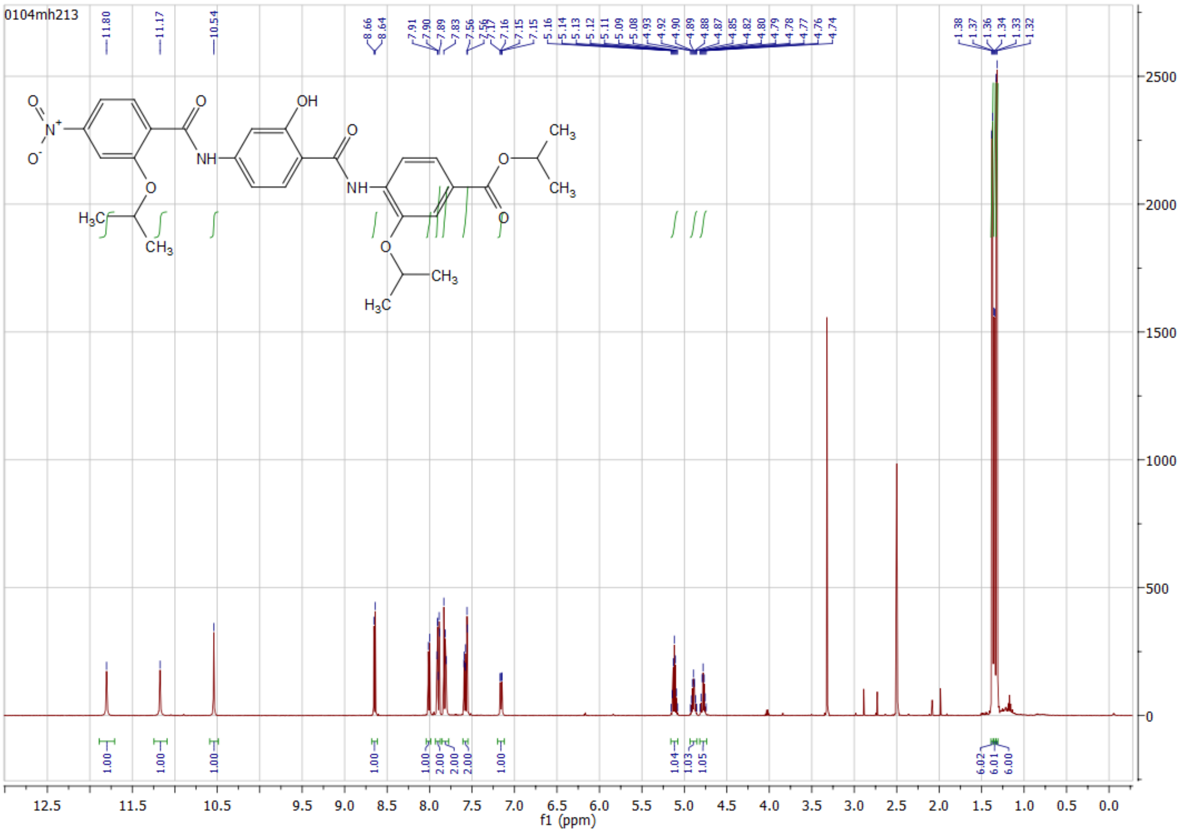

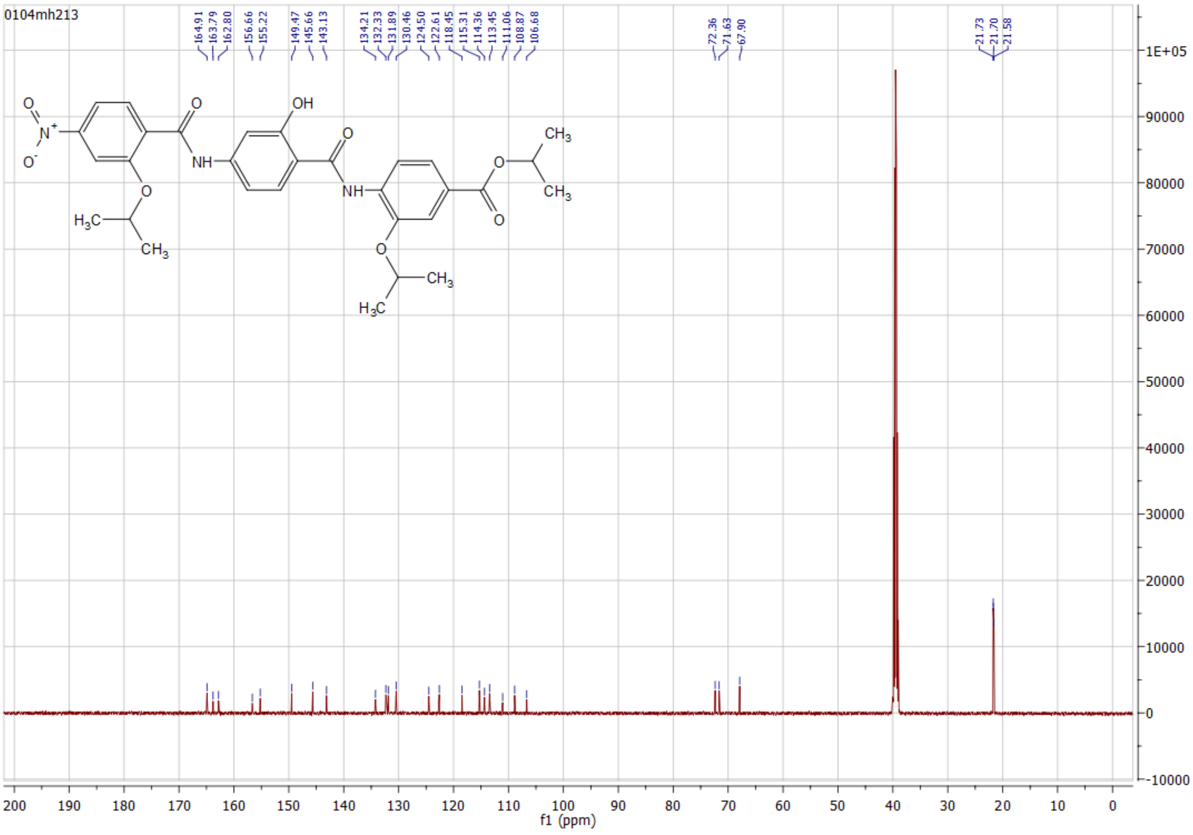

Compound **77**

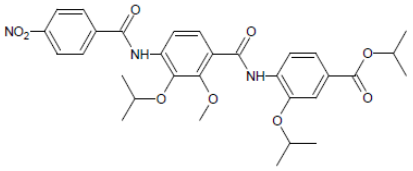
NO2
NH
O
NH
O
O
O
O
O
O

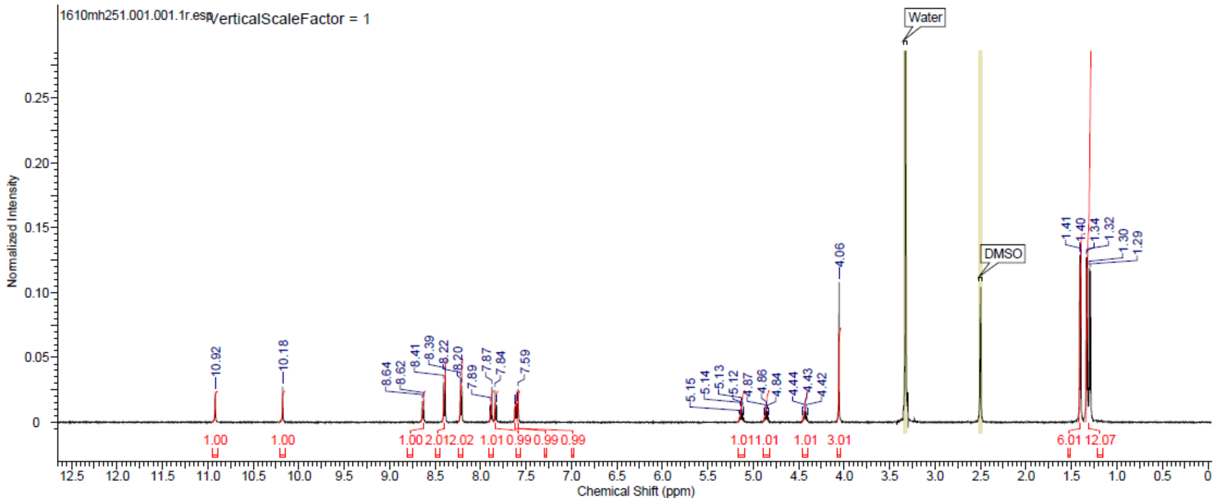
1610mh251.001.001.1r.esp
VerticalScaleFactor = 1
Water
DMSO
Normalized Intensity
10.92
10.18
8.64
8.62
8.41
8.39
8.22
8.20
7.89
7.87
7.84
7.59
5.15
5.14
5.13
5.12
4.87
4.86
4.84
4.44
4.43
4.42
4.06
1.41
1.40
1.34
1.32
1.30
1.29
1.00
1.00
1.00
2.01
2.02
1.01
0.99
0.99
0.99
1.01
1.01
1.01
3.01
6.01
12.07
Chemical Shift (ppm)

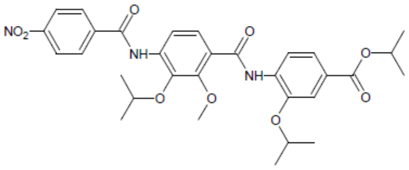
NO2
NH
O
NH
O
O
O
O
O
O

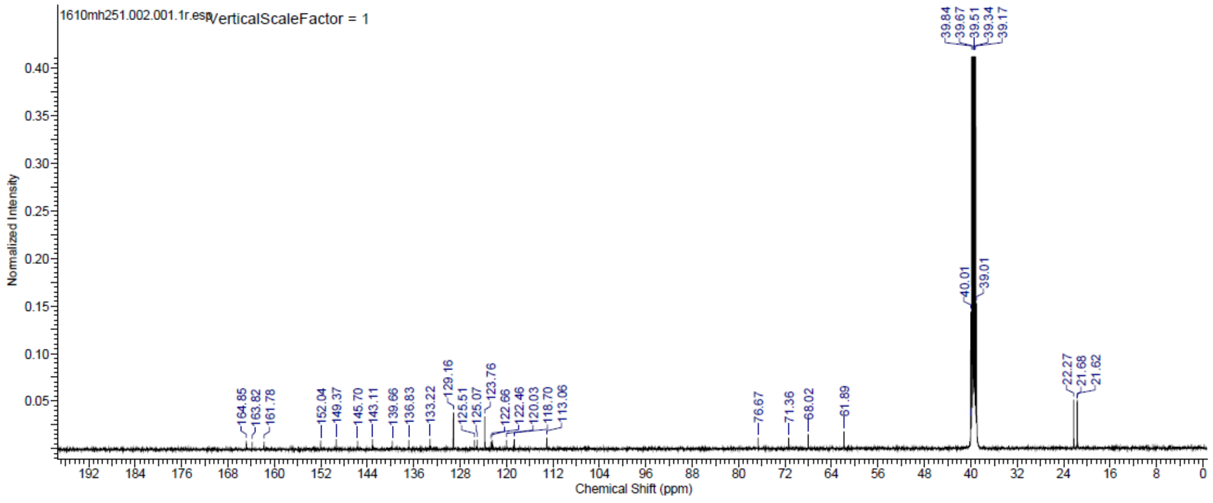
1610mh251.002.001.1r.esp
VerticalScaleFactor = 1
Normalized Intensity
164.85
163.82
161.78
152.04
149.37
145.70
143.11
139.66
136.83
133.22
129.16
125.51
125.07
123.76
122.66
122.46
120.03
118.70
113.06
76.67
71.36
68.02
61.89
39.84
39.67
39.51
39.34
39.17
40.01
39.01
22.27
21.68
21.62
Chemical Shift (ppm)

Compound **26**

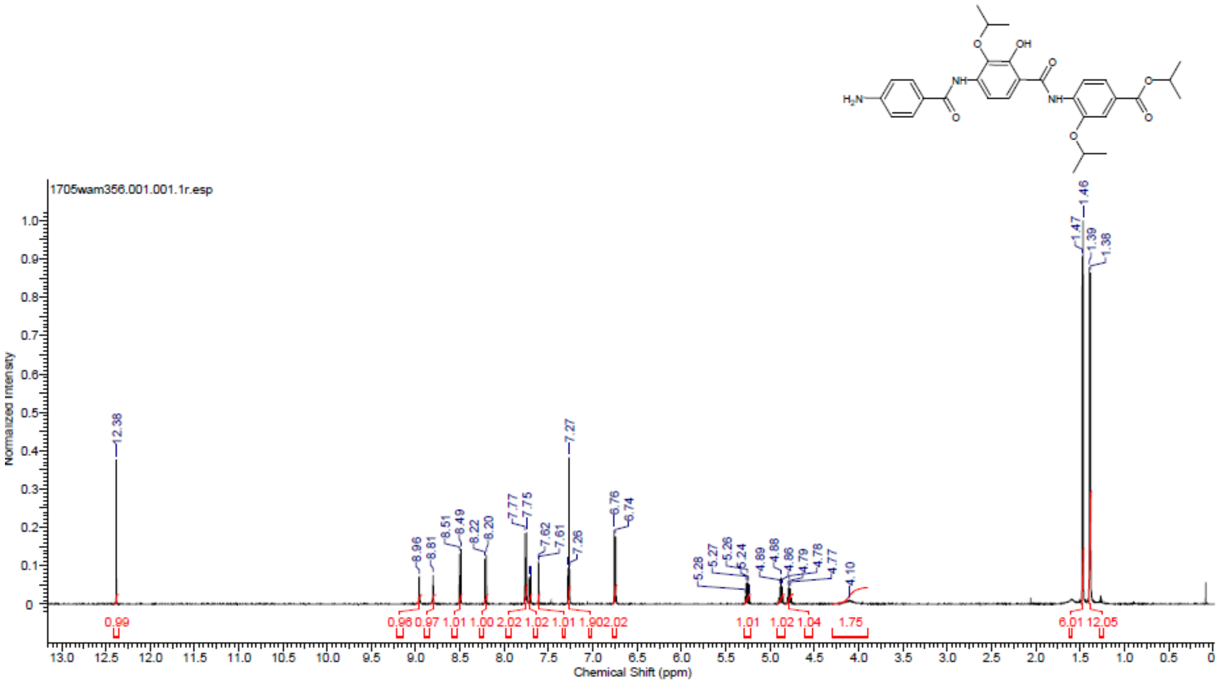

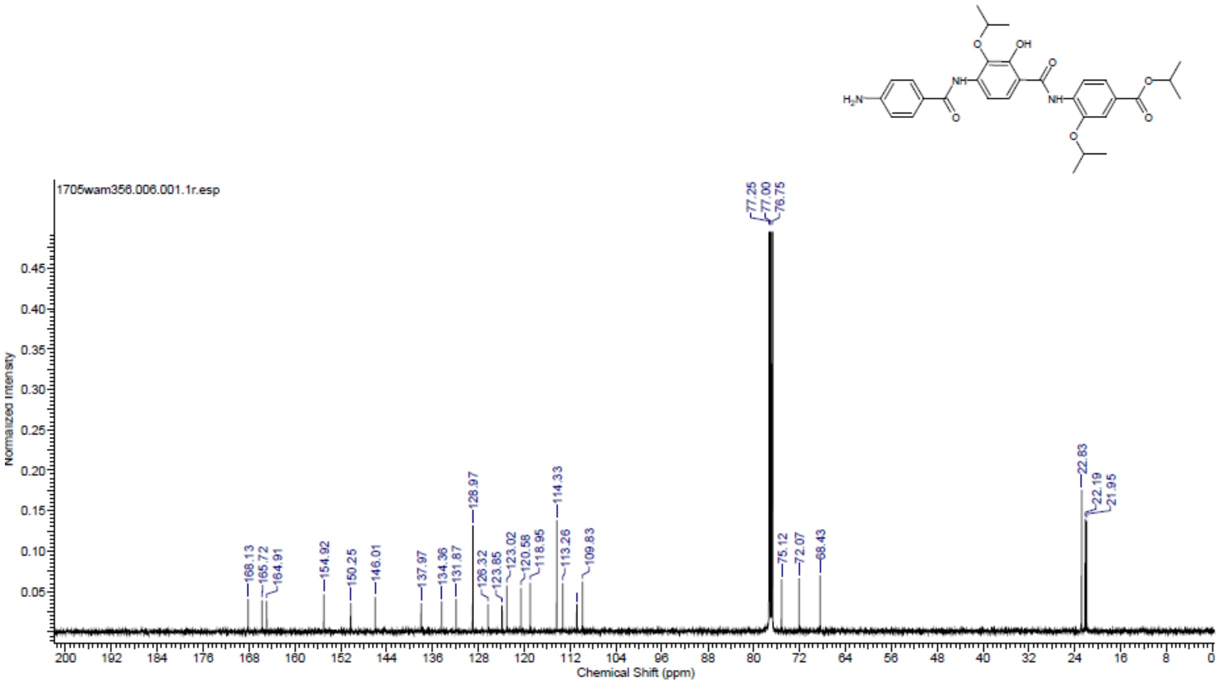

Compound **78**

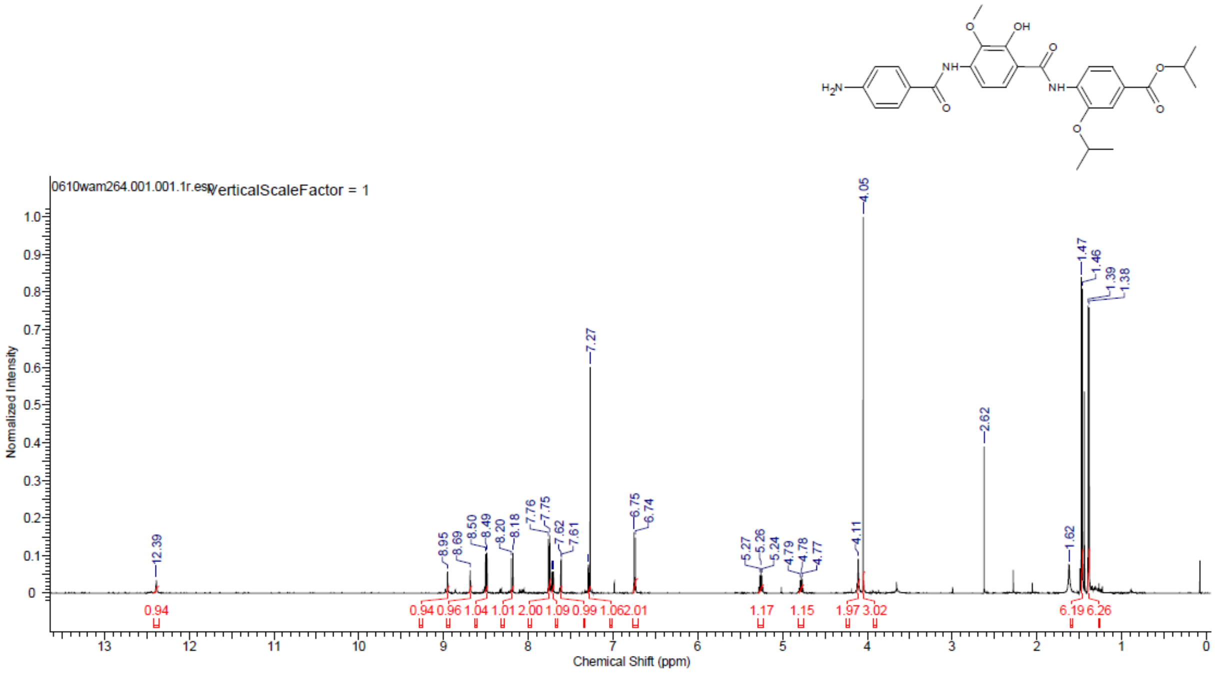

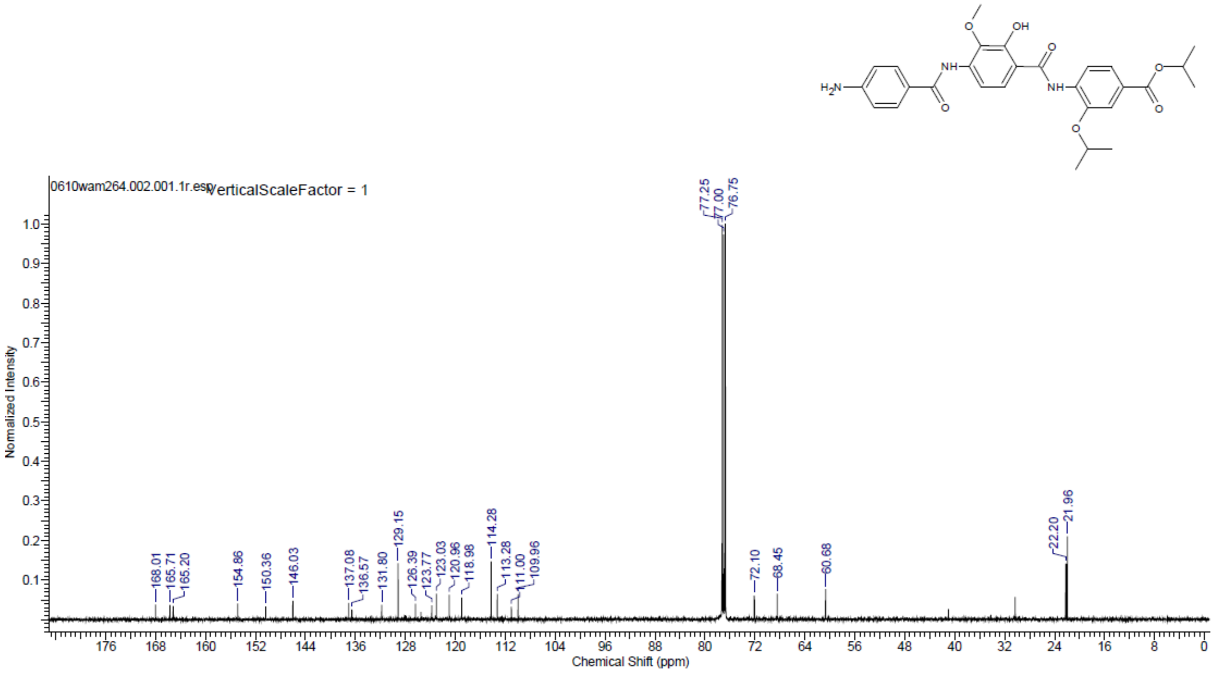

Compound **79**

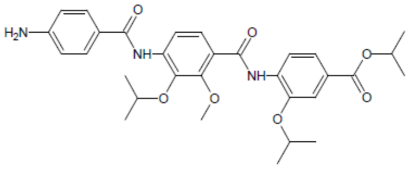

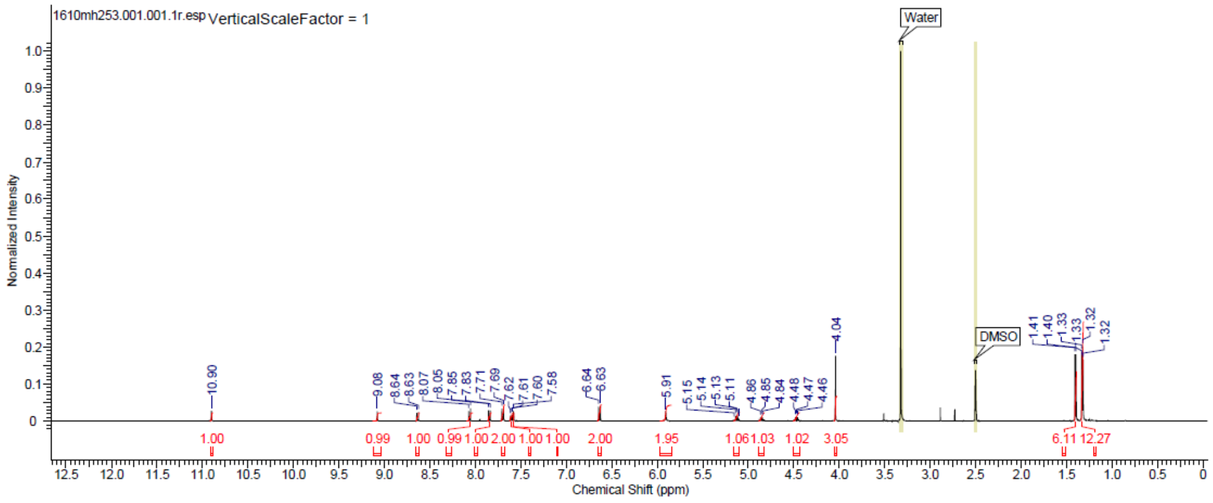

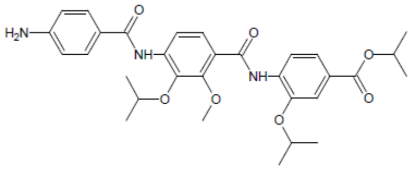

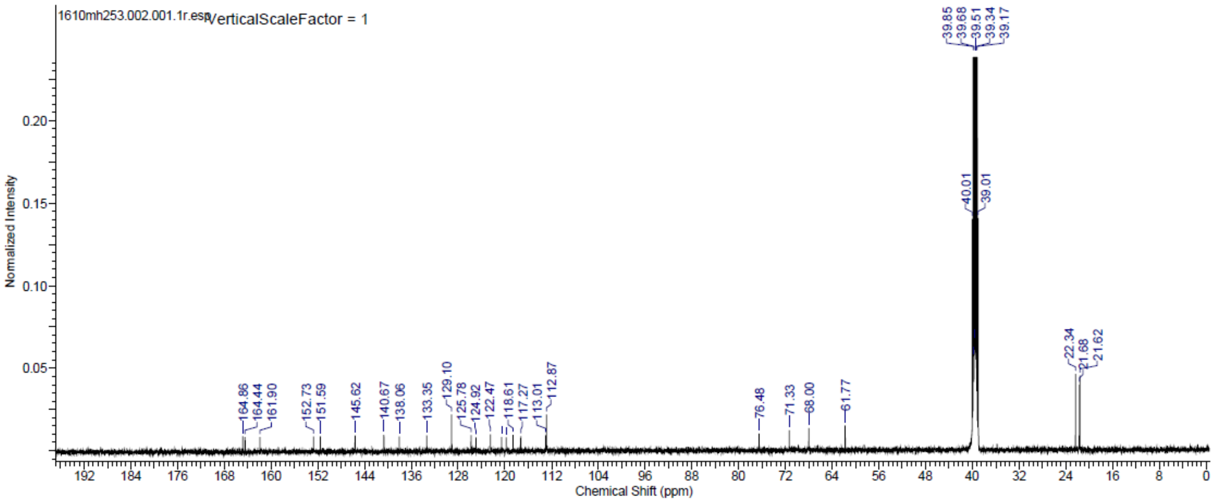

Compound **80**

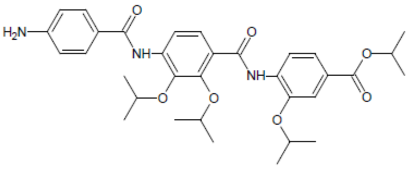

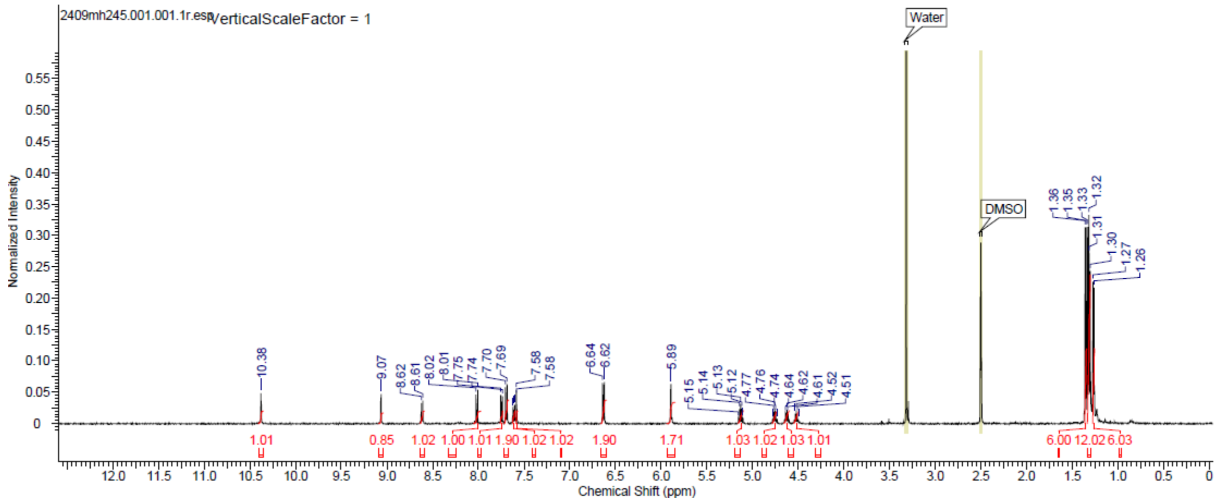

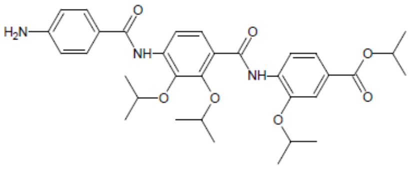

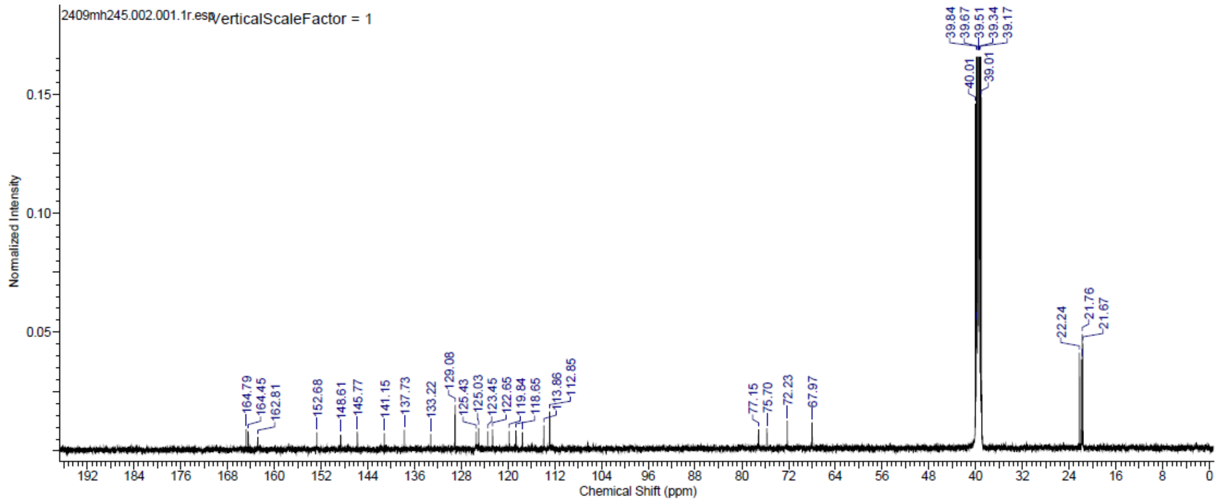

Compound **81**

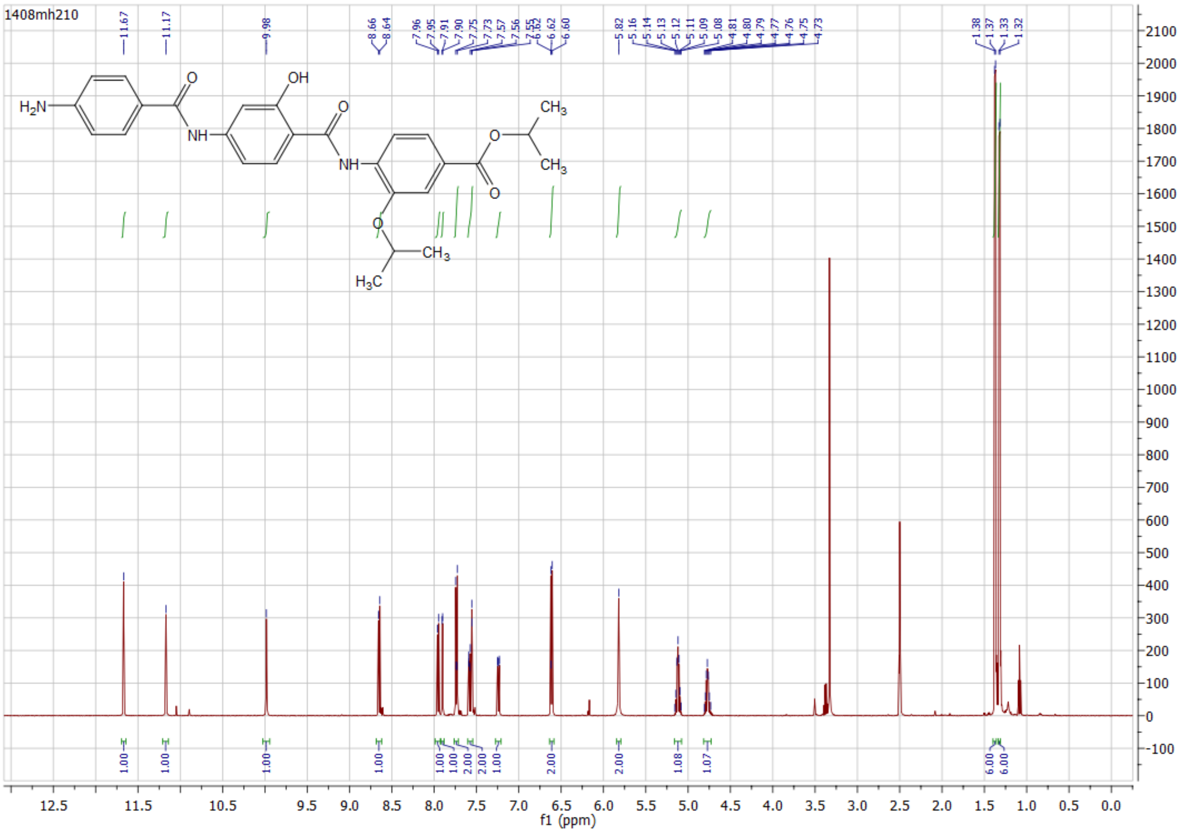

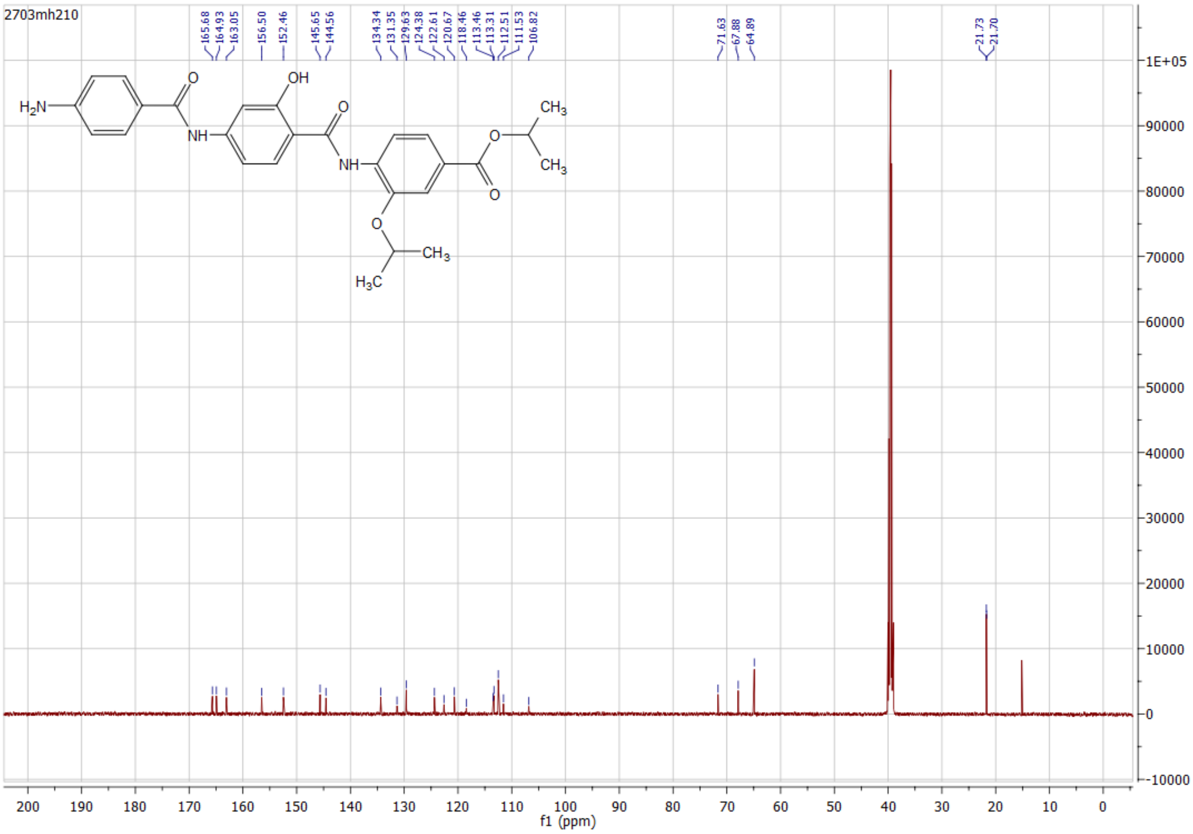

Compound **82**

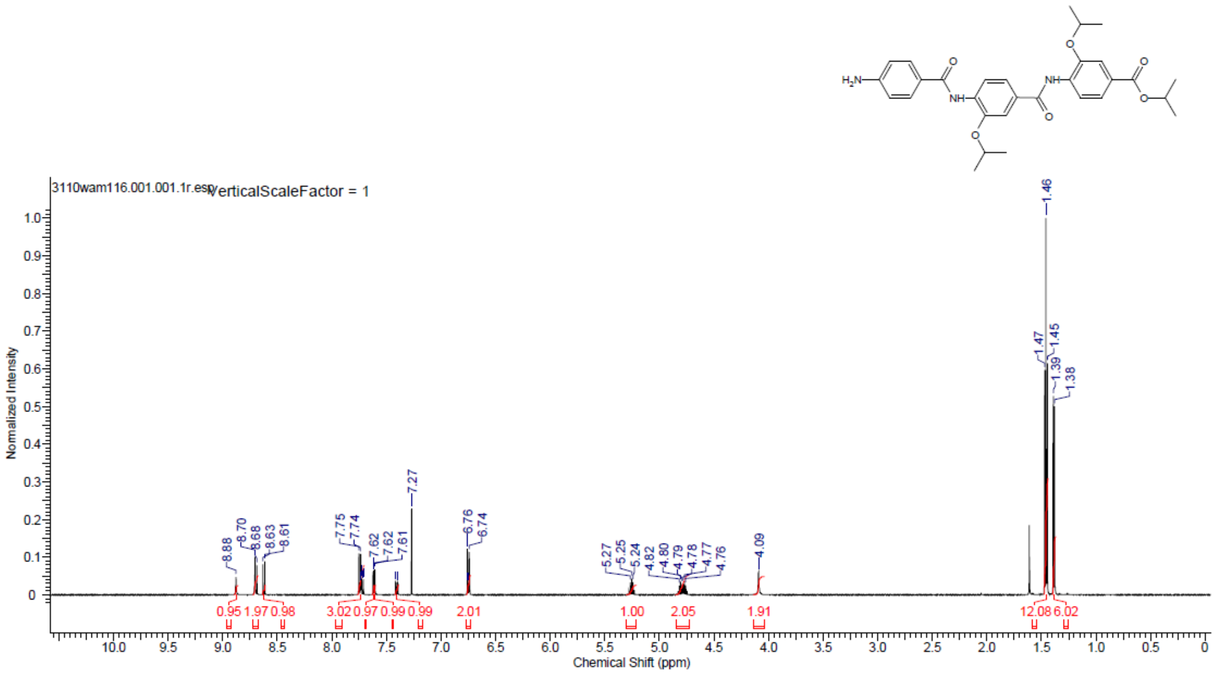

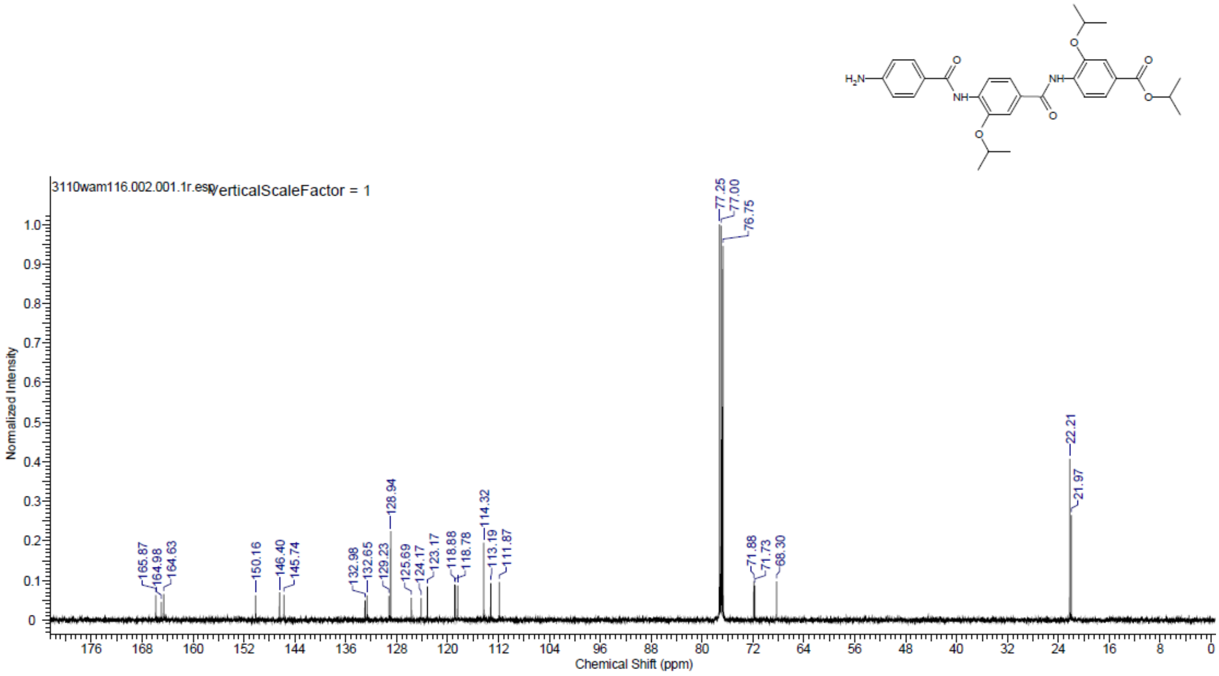

Compound **83**

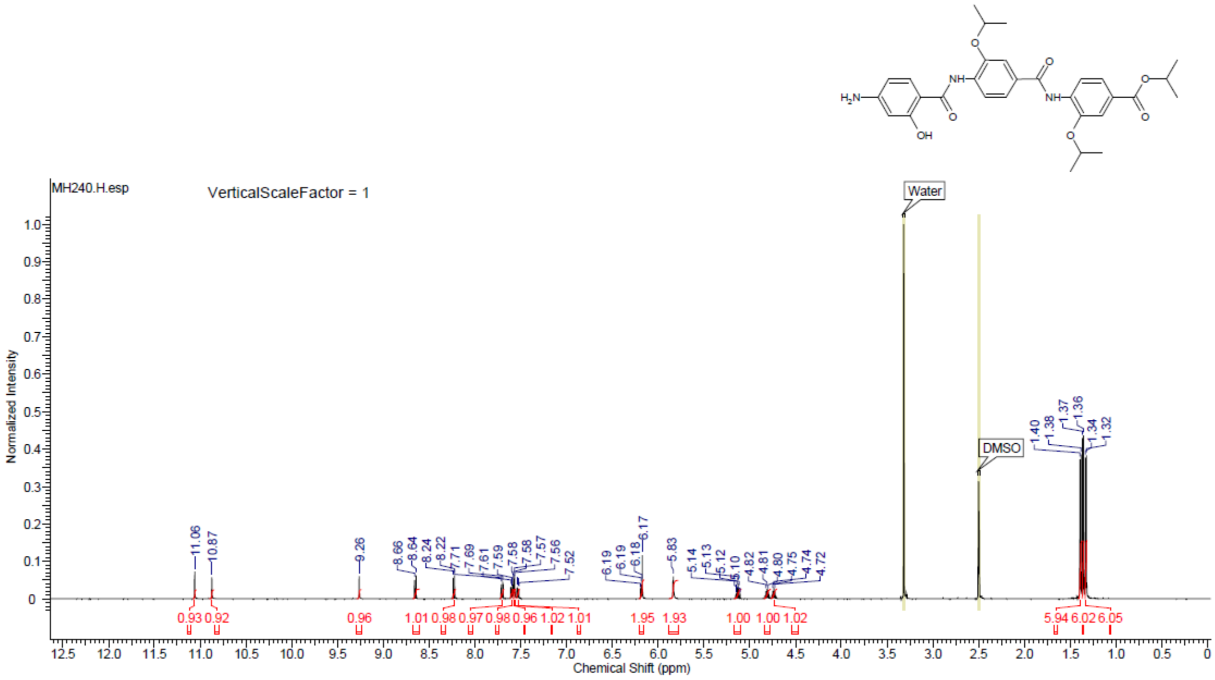
MH240.H.esp
VerticalScaleFactor = 1
Water
DMSO
Normalized Intensity
Chemical Shift (ppm)

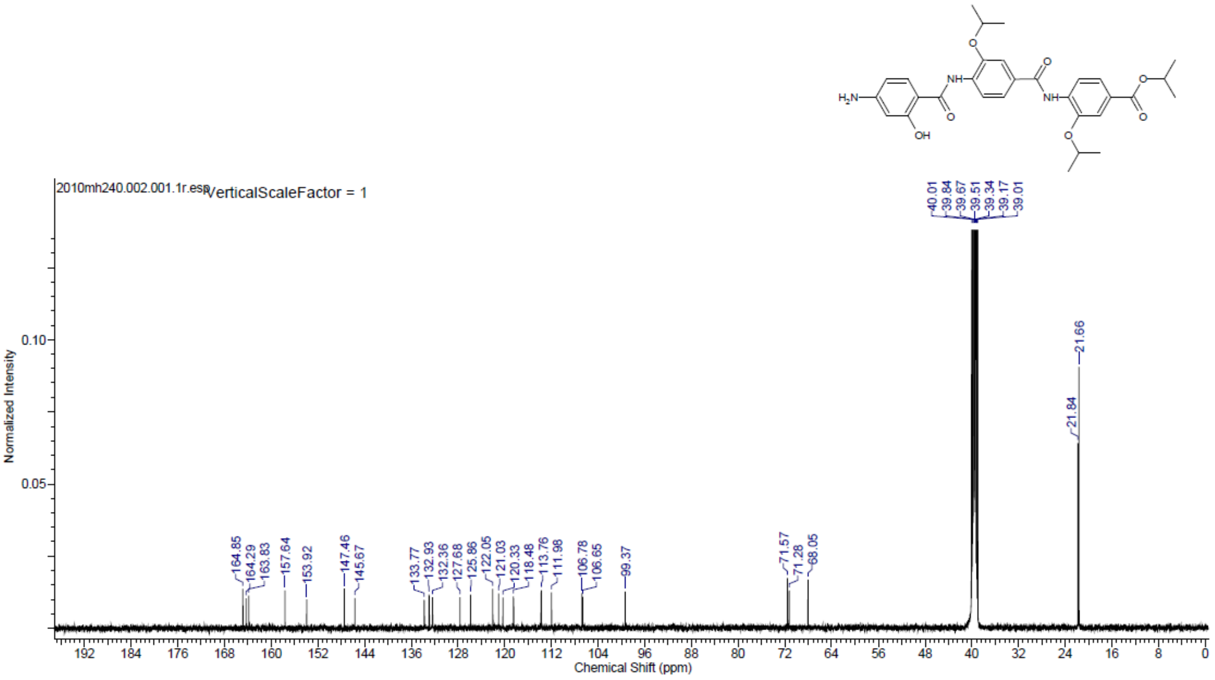
2010mh240.002.001.1r.esp
VerticalScaleFactor = 1
Normalized Intensity
Chemical Shift (ppm)

Compound **84**

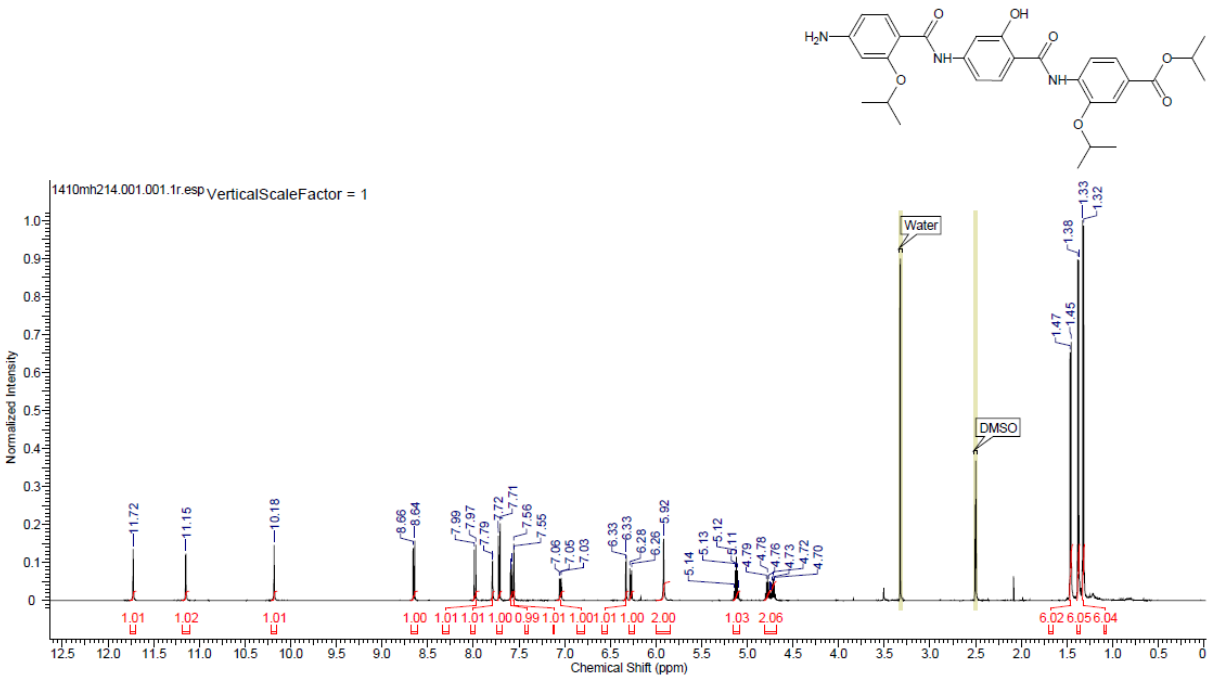
H2N
O
OH
NH
O
NH
O
O
O
O
1410mh214.001.001.1r.esp VerticalScaleFactor = 1
Water
DMSO
Normalized Intensity
Chemical Shift (ppm)
11.72
11.15
10.18
8.66
8.64
7.99
7.97
7.79
7.72
7.71
7.56
7.55
7.06
7.05
7.03
6.33
6.33
6.28
6.26
5.92
5.14
5.13
5.12
5.11
4.79
4.78
4.76
4.73
4.72
4.70
1.47
1.45
1.38
1.33
1.32
1.01
1.02
1.01
1.00
1.01
1.01
1.00
0.99
1.01
1.00
1.01
1.00
2.00
1.03
2.06
6.02
6.05
6.04

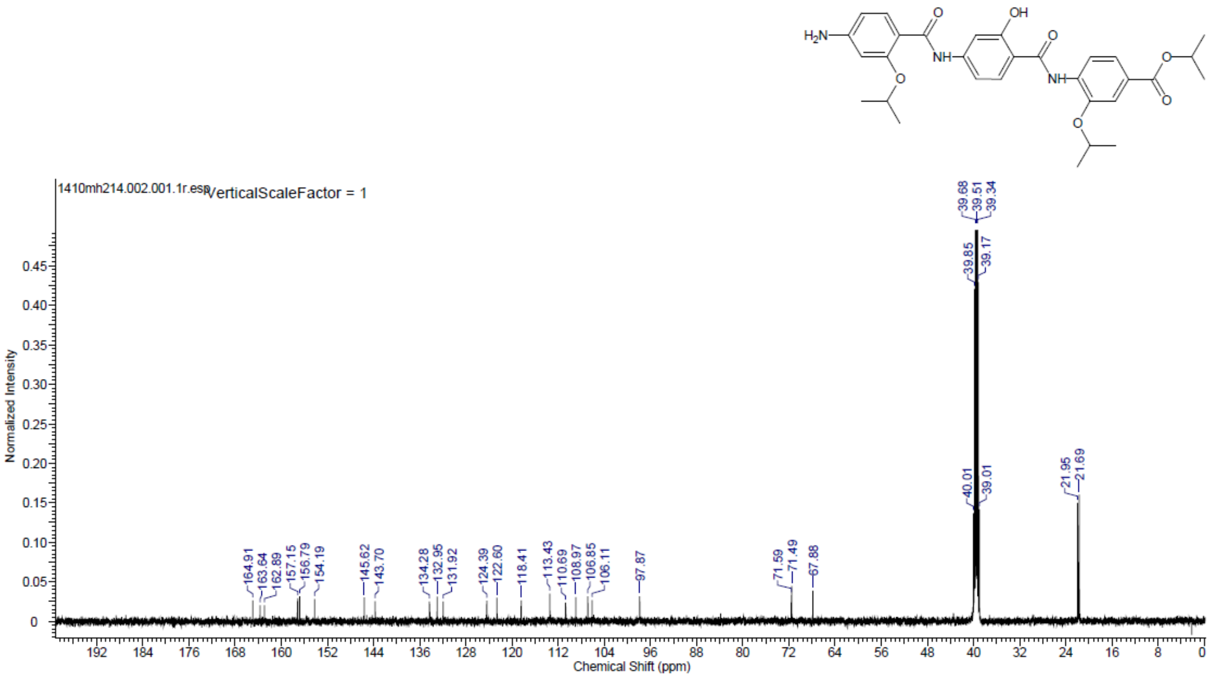
H2N
O
OH
NH
O
NH
O
O
O
O
1410mh214.002.001.1r.esp VerticalScaleFactor = 1
Normalized Intensity
Chemical Shift (ppm)
164.91
163.64
162.89
157.15
156.79
154.19
145.62
143.70
134.28
132.95
131.92
124.39
122.60
118.41
113.43
110.69
108.97
106.85
106.11
97.87
71.59
71.49
67.88
39.68
39.51
39.34
39.85
39.17
40.01
39.01
21.95
21.69

Compound **85**

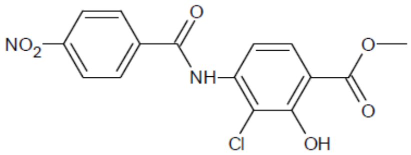
NO2
O
NH
O
O
Cl
OH

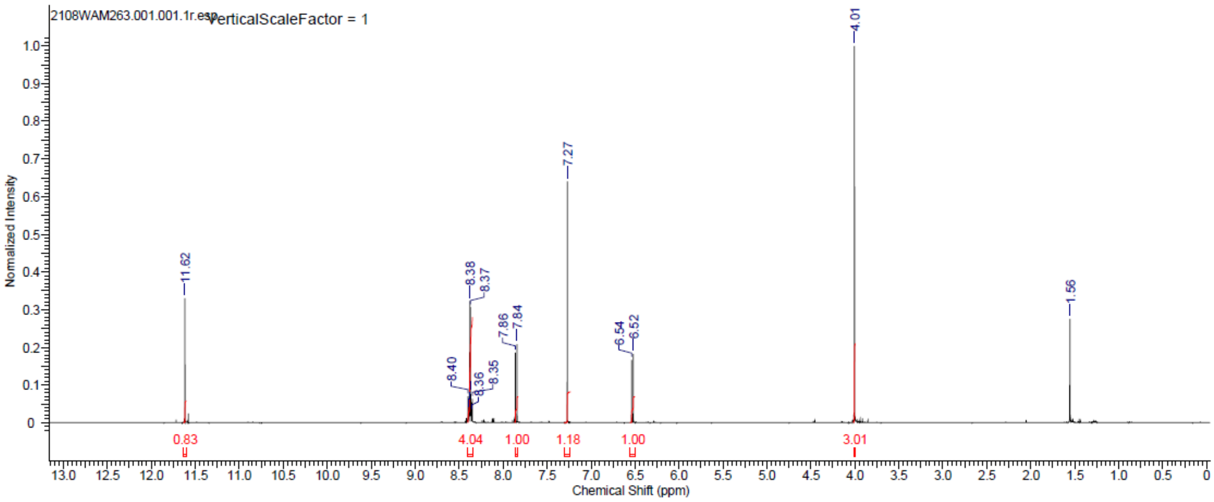
2108WAM263.001.001.1r.esp
VerticalScaleFactor = 1
11.62
8.40
8.38
8.37
8.36
8.35
7.86
7.84
7.27
6.54
6.52
4.01
1.56
0.83
4.04
1.00
1.18
1.00
3.01
Normalized Intensity
Chemical Shift (ppm)

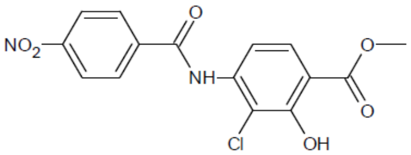
NO2
O
NH
O
O
Cl
OH

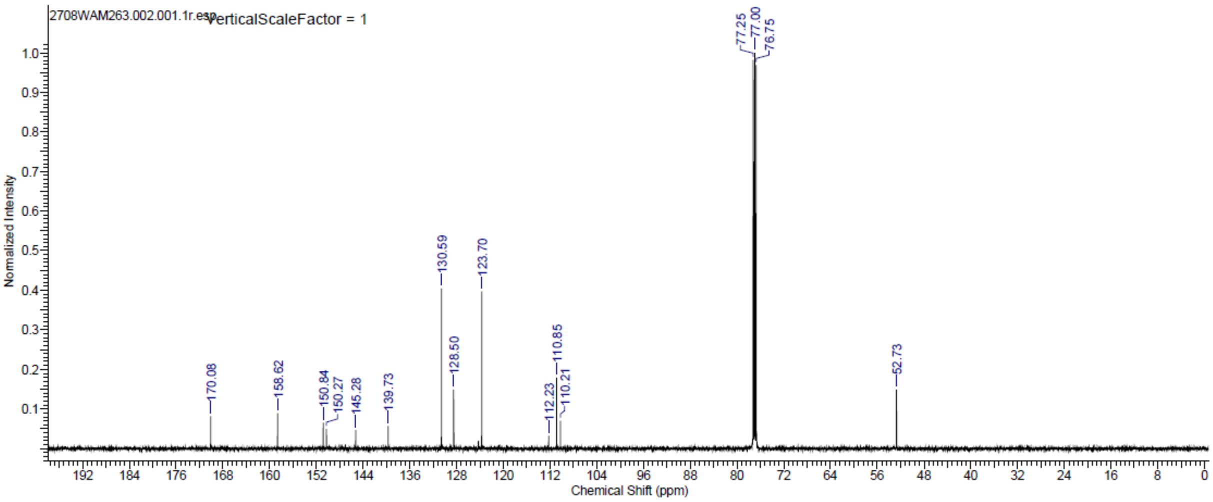
2708WAM263.002.001.1r.esp
VerticalScaleFactor = 1
77.25
77.00
76.75
170.08
158.62
150.84
150.27
145.28
139.73
130.59
128.50
123.70
112.23
110.85
110.21
52.73
Normalized Intensity
Chemical Shift (ppm)

Compound **87**

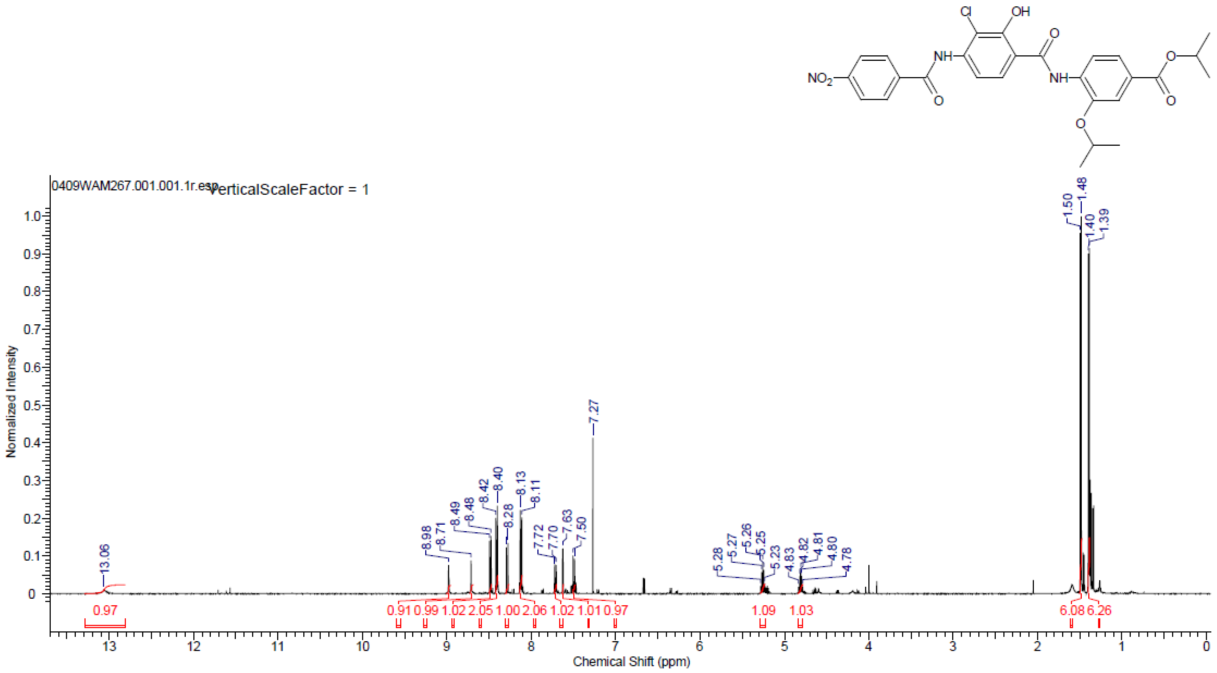

Cl
OH
NH
O
NO2
NH
O
O
O
O
0409WAM267.001.001.1r.esp
VerticalScaleFactor = 1
Normalized Intensity
13.06
8.98
8.71
8.49
8.48
8.42
8.40
8.28
8.13
8.11
7.72
7.70
7.63
7.50
7.27
5.28
5.27
5.26
5.25
5.23
4.83
4.82
4.81
4.80
4.78
1.50
1.48
1.40
1.39
0.97
0.91
0.99
1.02
2.05
1.00
2.06
1.02
1.01
0.97
1.09
1.03
6.08
6.26
Chemical Shift (ppm)

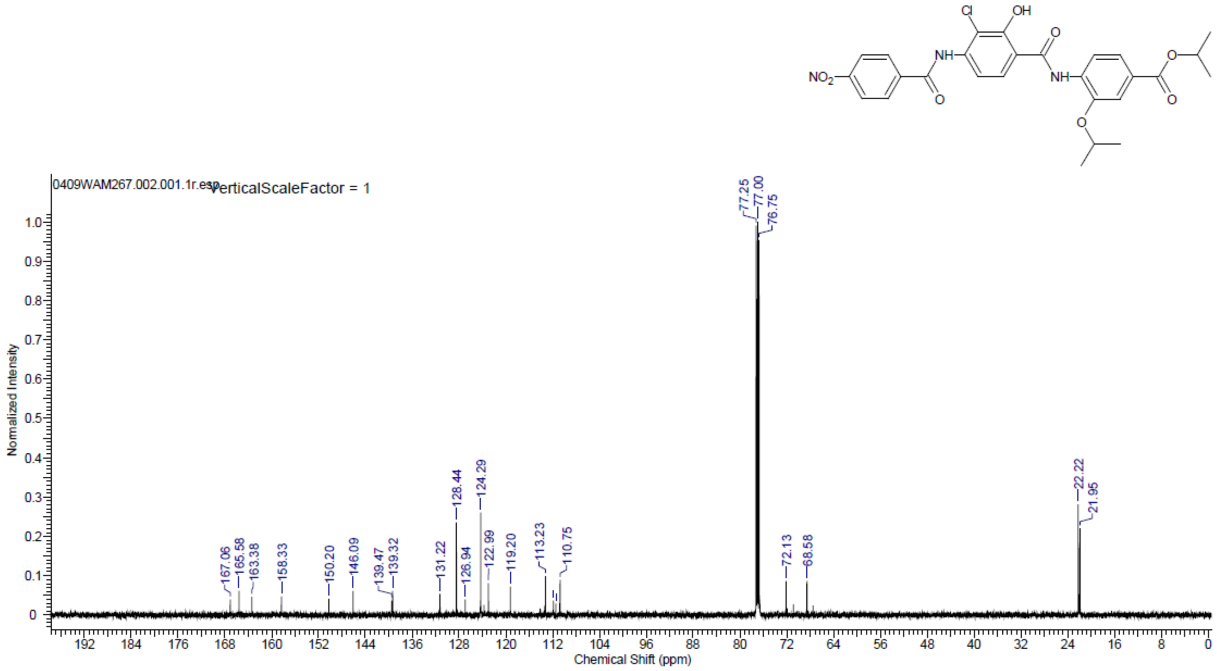

Cl
OH
NH
O
NO2
NH
O
O
O
O
0409WAM267.002.001.1r.esp
VerticalScaleFactor = 1
Normalized Intensity
167.06
165.58
163.38
158.33
150.20
146.09
139.47
139.32
131.22
128.44
126.94
124.29
122.99
119.20
113.23
110.75
77.25
77.00
76.75
72.13
68.58
22.22
21.95
Chemical Shift (ppm)

Compound **88**

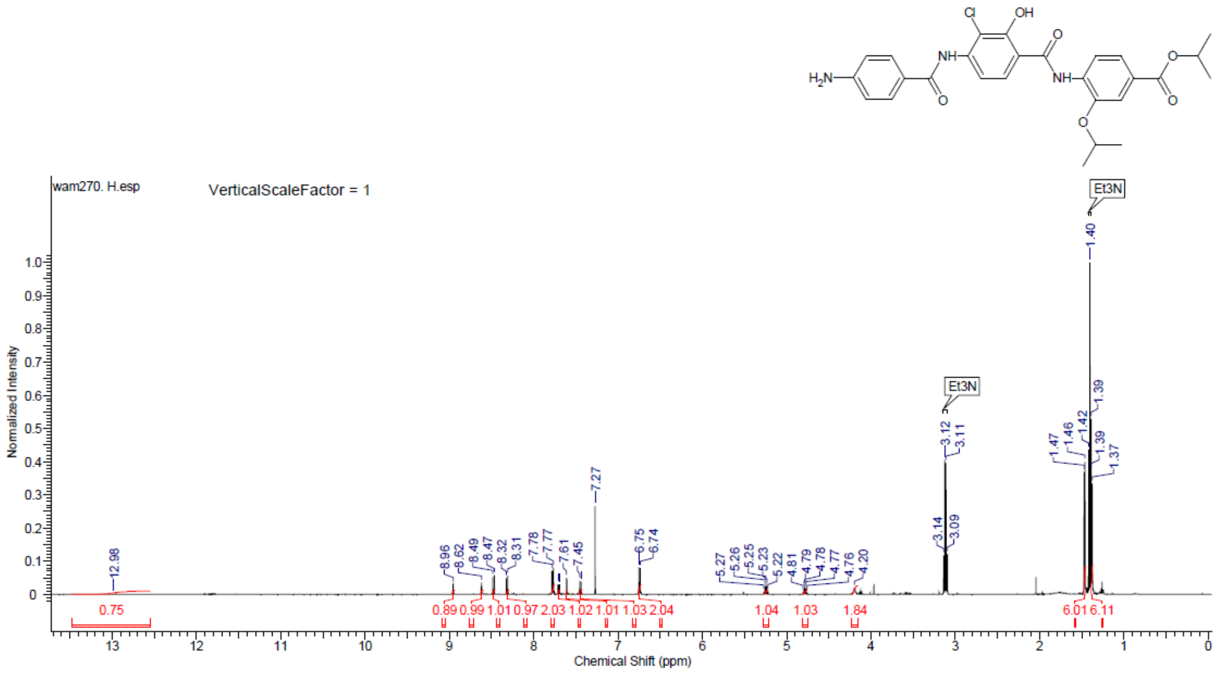

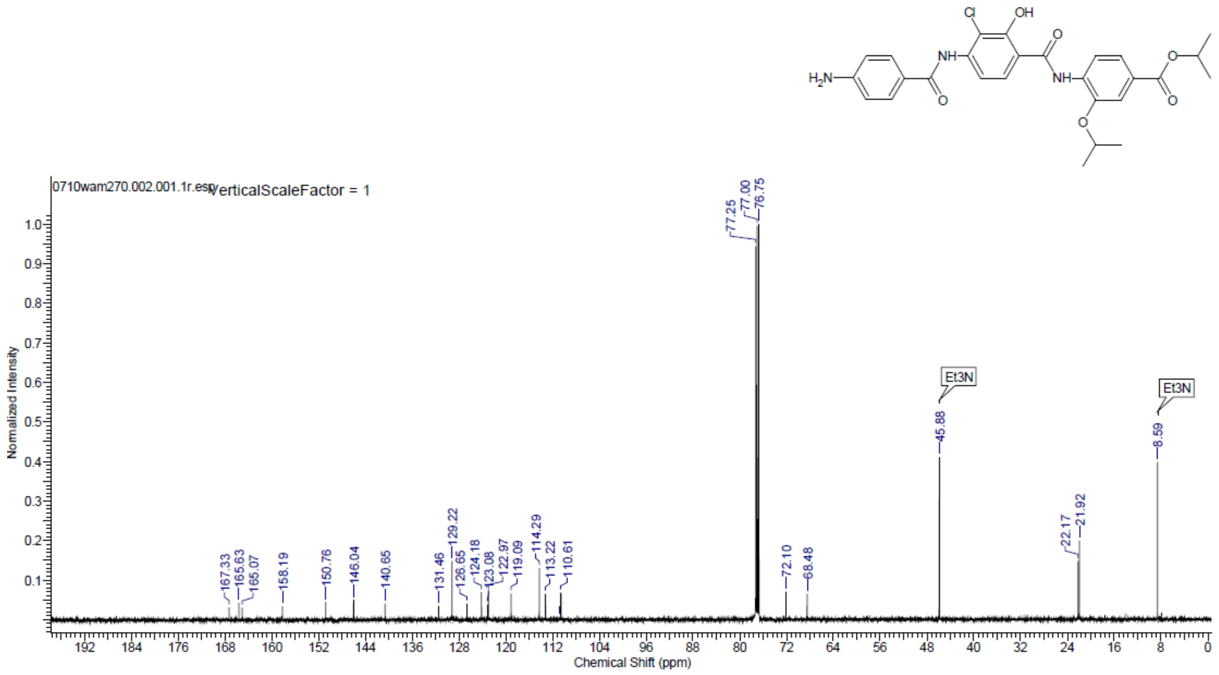

Compound **89**

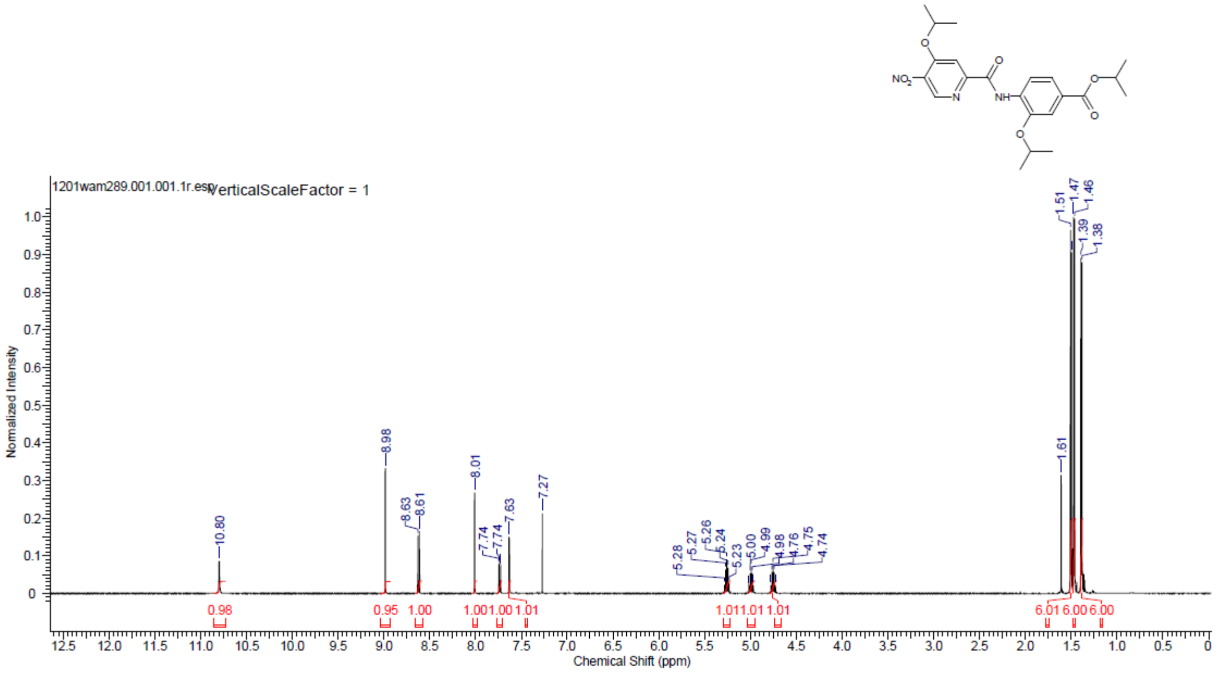

1201wam289.001.001.1r.esp
VerticalScaleFactor = 1
Normalized Intensity
Chemical Shift (ppm)
10.80
8.98
8.63
8.61
8.01
7.74
7.74
7.63
7.27
5.28
5.27
5.26
5.24
5.23
5.00
4.99
4.98
4.76
4.75
4.74
1.61
1.51
1.47
1.46
1.39
1.38
0.98
0.95
1.00
1.00
1.00
1.01
1.01
1.01
1.01
6.01
6.00
6.00
NO2
NH
N
O

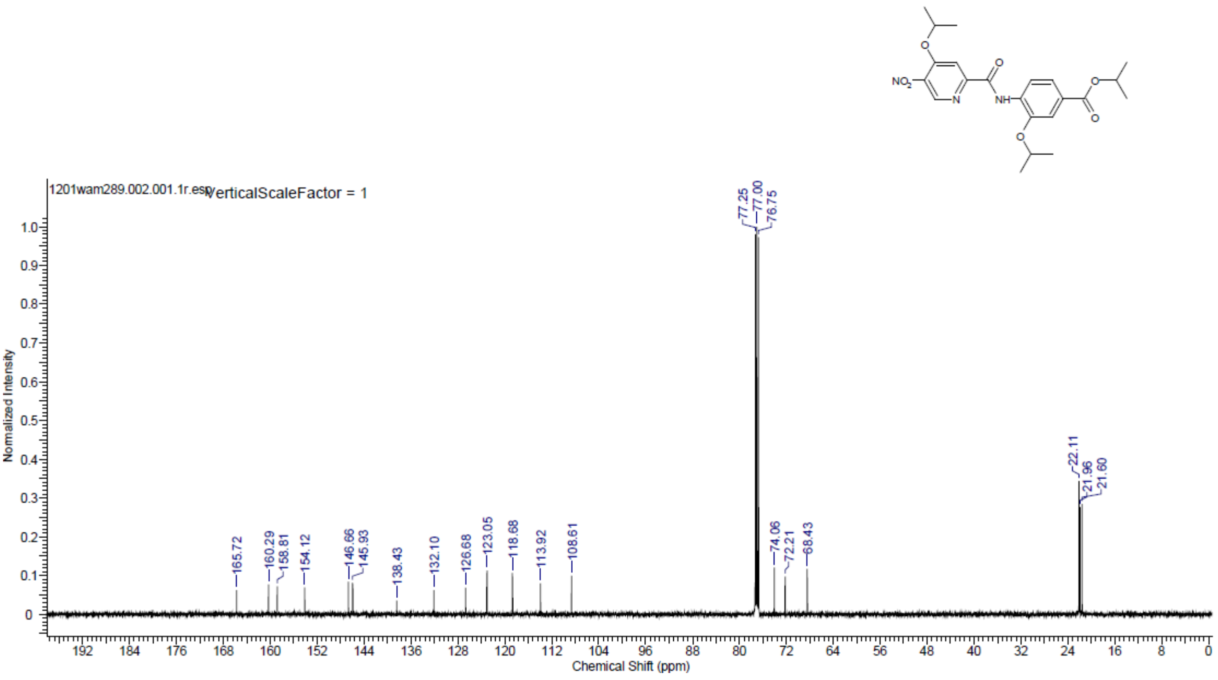

1201wam289.002.001.1r.esp
VerticalScaleFactor = 1
Normalized Intensity
Chemical Shift (ppm)
165.72
160.29
158.81
154.12
146.66
145.93
138.43
132.10
126.68
123.05
118.68
113.92
108.61
77.25
77.00
76.75
74.06
72.21
68.43
22.11
21.96
21.60
NO2
NH
N
O

Compound **90**

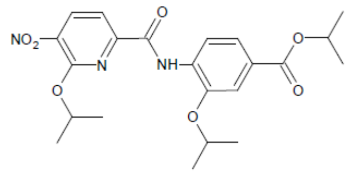
NO2
N
O
NH
O
O
O
O

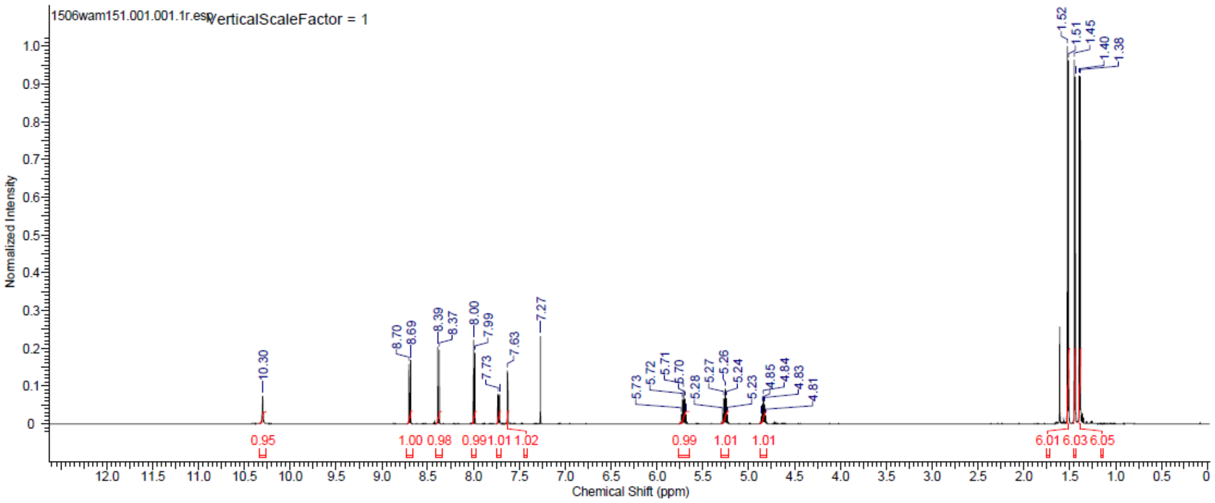
1506wam151.001.001.1r.esp
VerticalScaleFactor = 1
Normalized Intensity
Chemical Shift (ppm)
10.30
8.70
8.69
8.39
8.37
8.00
7.99
7.73
7.63
7.27
5.73
5.72
5.71
5.70
5.28
5.27
5.26
5.24
5.23
4.85
4.84
4.83
4.81
1.52
1.51
1.45
1.40
1.38
0.95
1.00
0.98
0.99
1.01
1.02
0.99
1.01
1.01
6.01
6.03
6.05

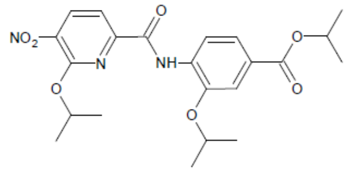
NO2
N
O
NH
O
O
O
O

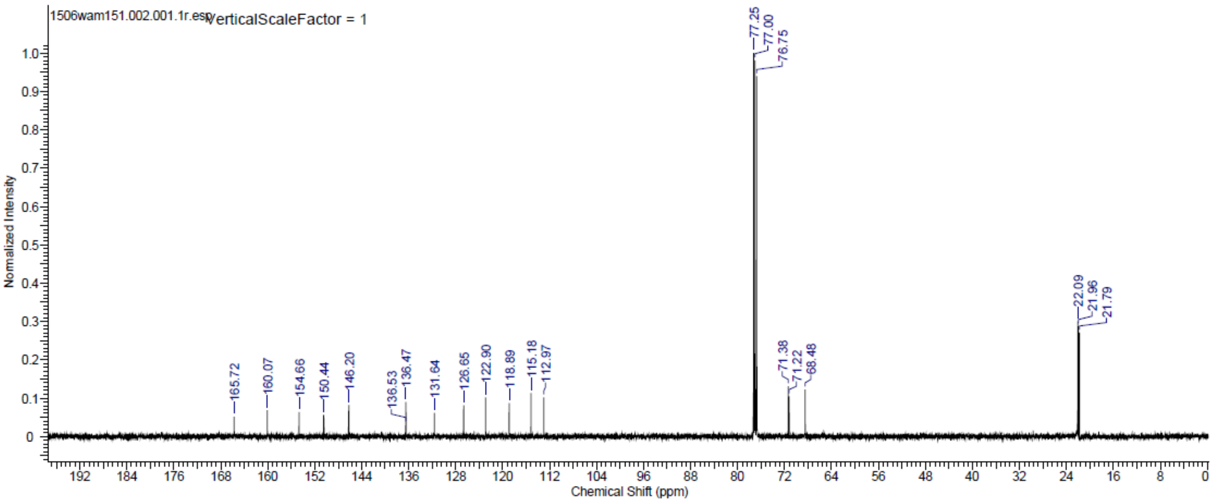
1506wam151.002.001.1r.esp
VerticalScaleFactor = 1
Normalized Intensity
Chemical Shift (ppm)
165.72
160.07
154.66
150.44
146.20
136.53
136.47
131.64
126.65
122.90
118.89
115.18
112.97
77.25
77.00
76.75
71.38
71.22
68.48
22.09
21.96
21.79

Compound **91**

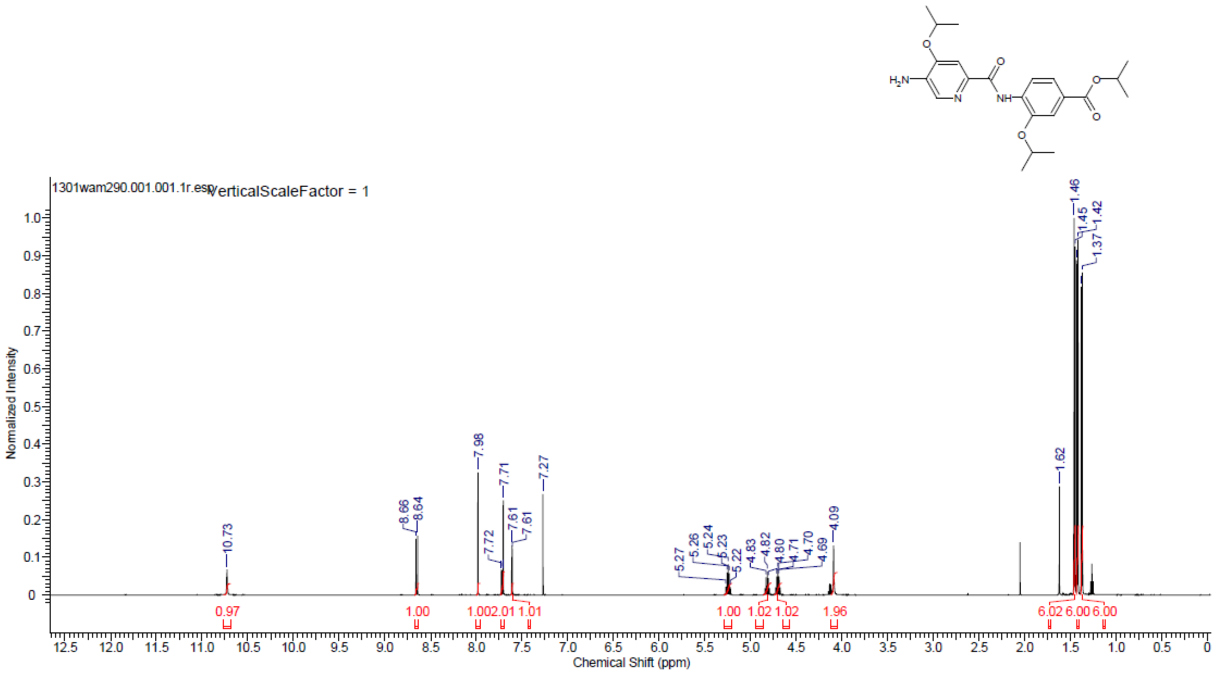
1301wam290.001.001.1r.esp
VerticalScaleFactor = 1
Normalized Intensity
Chemical Shift (ppm)
H_2N
N
NH
O

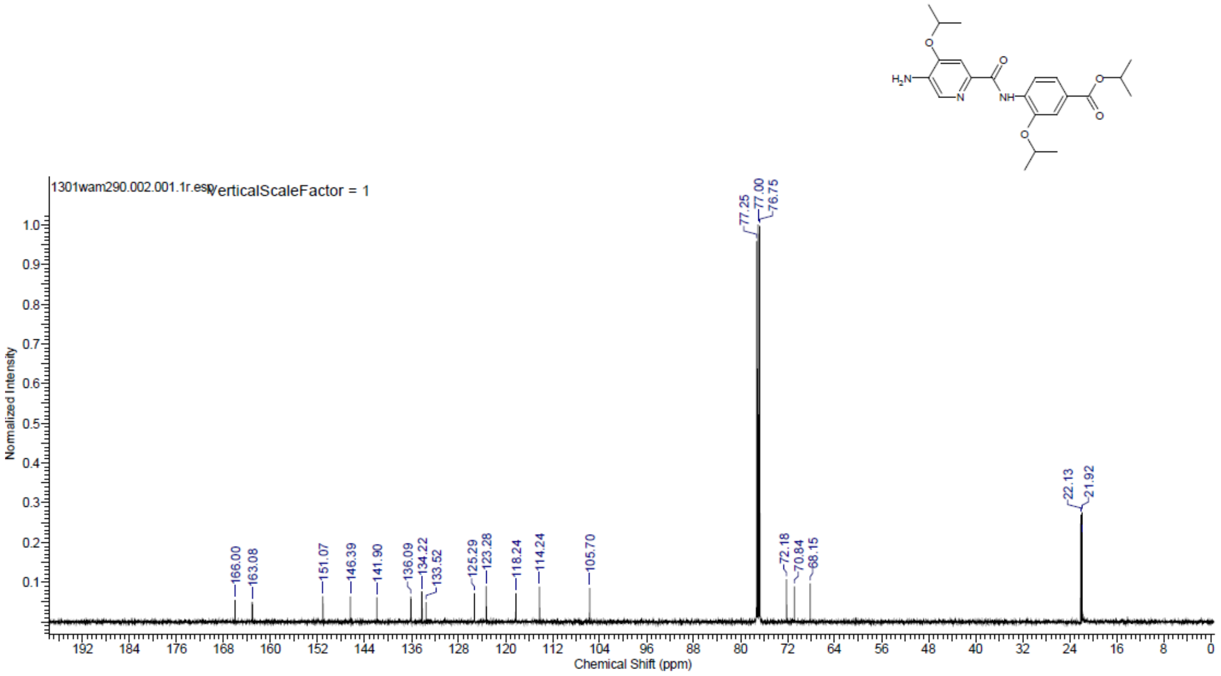
1301wam290.002.001.1r.esp
VerticalScaleFactor = 1
Normalized Intensity
Chemical Shift (ppm)
H_2N
N
NH
O

Compound **92**

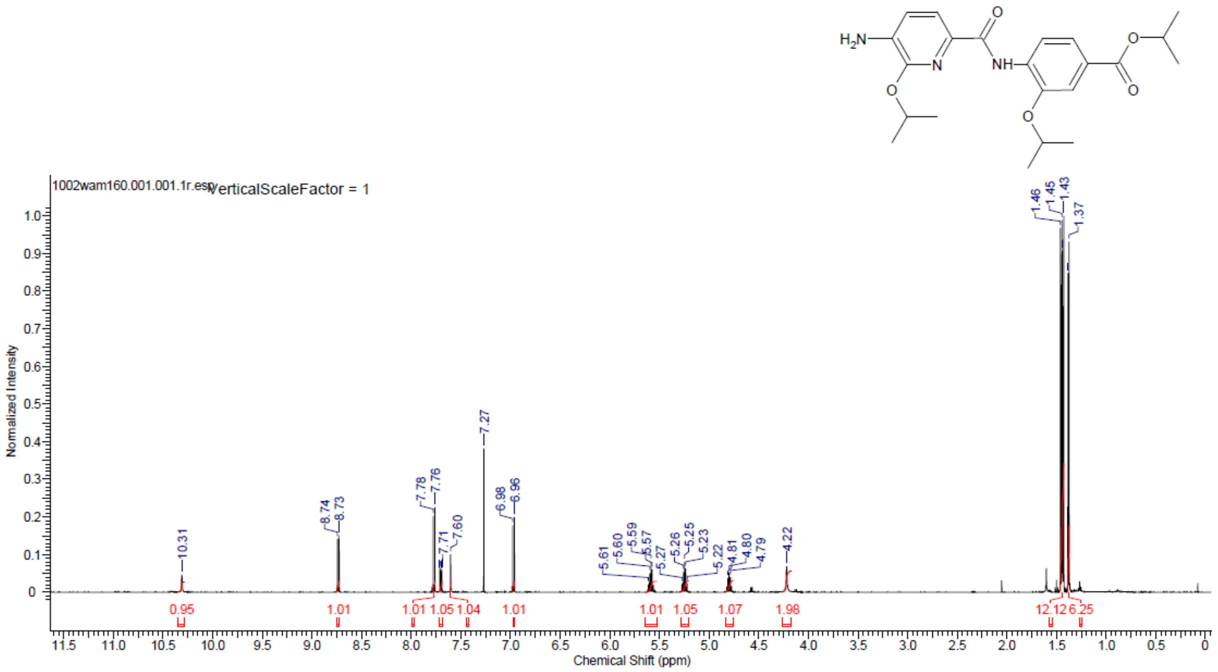
1002wam160.001.001.1r.esp
VerticalScaleFactor = 1
Normalized Intensity
Chemical Shift (ppm)
10.31
8.74
8.73
7.78
7.76
7.71
7.60
7.27
6.98
6.96
5.61
5.60
5.59
5.57
5.27
5.26
5.25
5.23
5.22
4.81
4.80
4.79
4.22
1.46
1.45
1.43
1.37
0.95
1.01
1.01
1.05
1.04
1.01
1.01
1.05
1.07
1.98
12.12
6.25

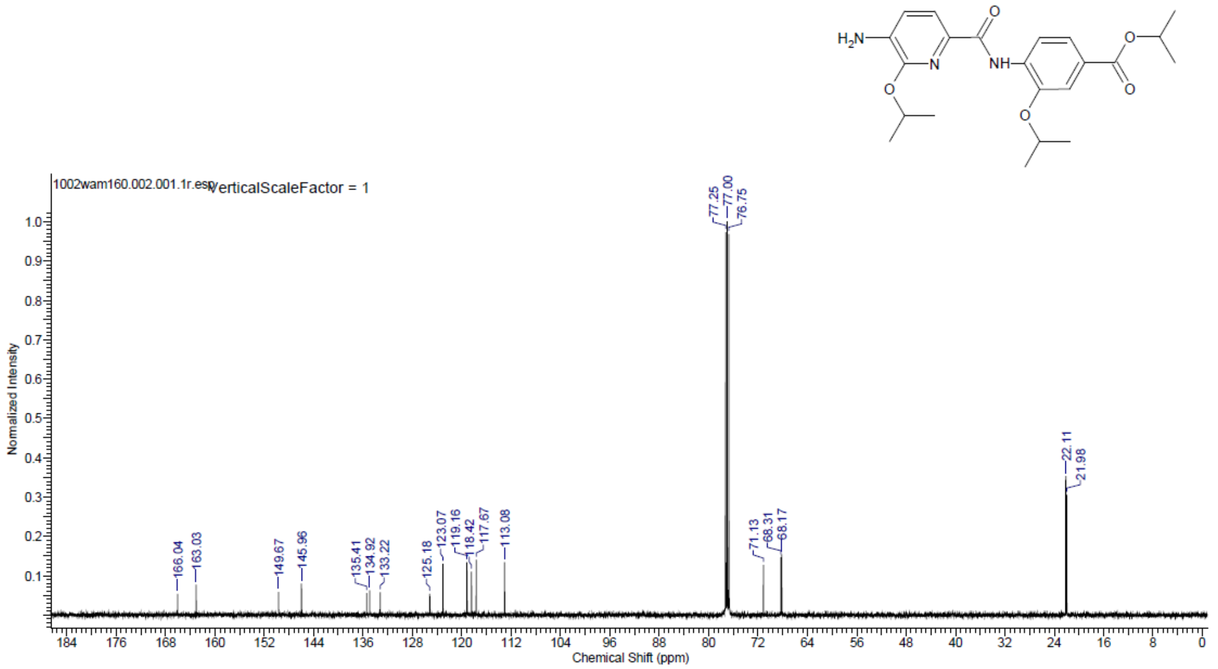
1002wam160.002.001.1r.esp
VerticalScaleFactor = 1
Normalized Intensity
Chemical Shift (ppm)
166.04
163.03
149.67
145.96
135.41
134.92
133.22
125.18
123.07
119.16
118.42
117.67
113.08
77.25
77.00
76.75
71.13
68.31
68.17
22.11
21.98

Compound **93**

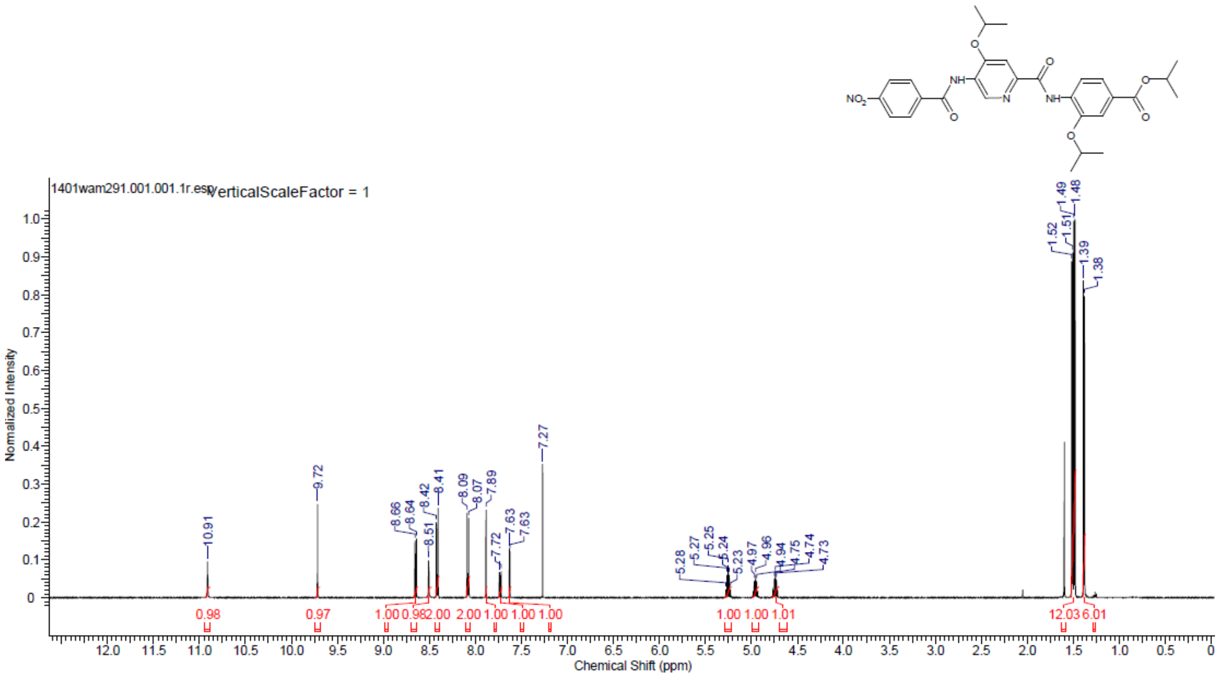

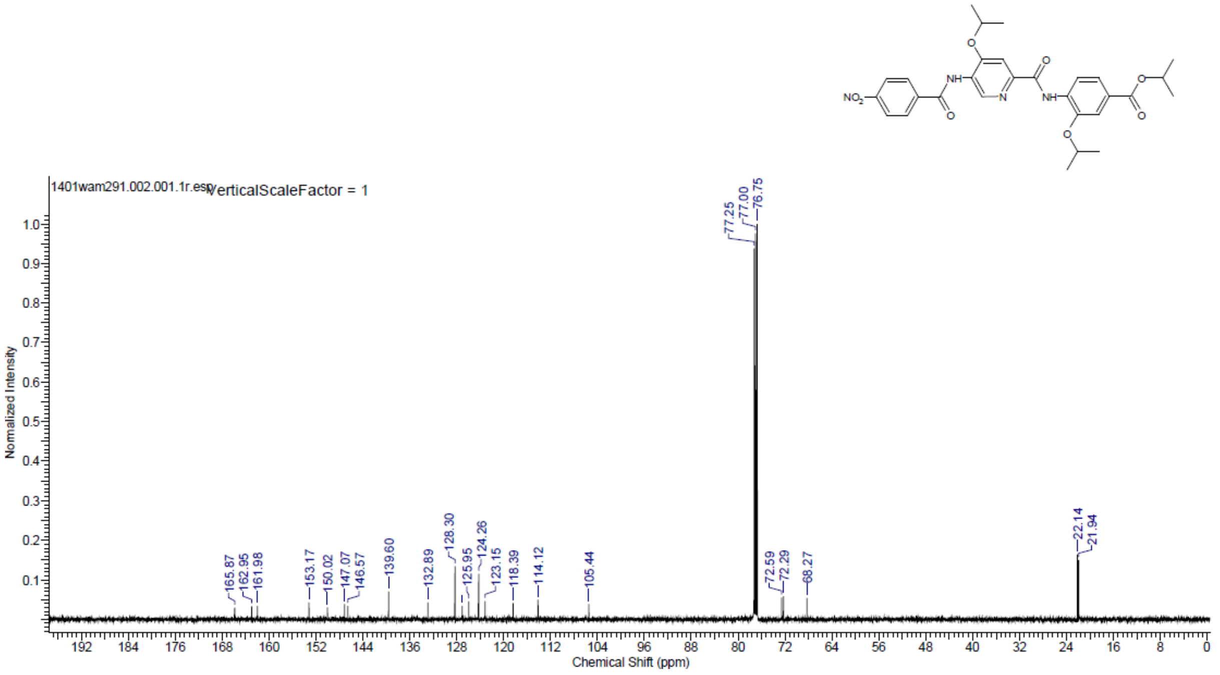

Compound **94**

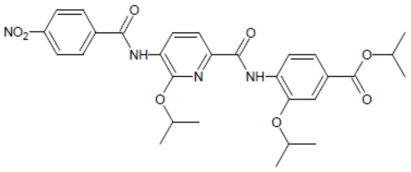
NO2
NH
N
O
NH
O
O
O
O
O

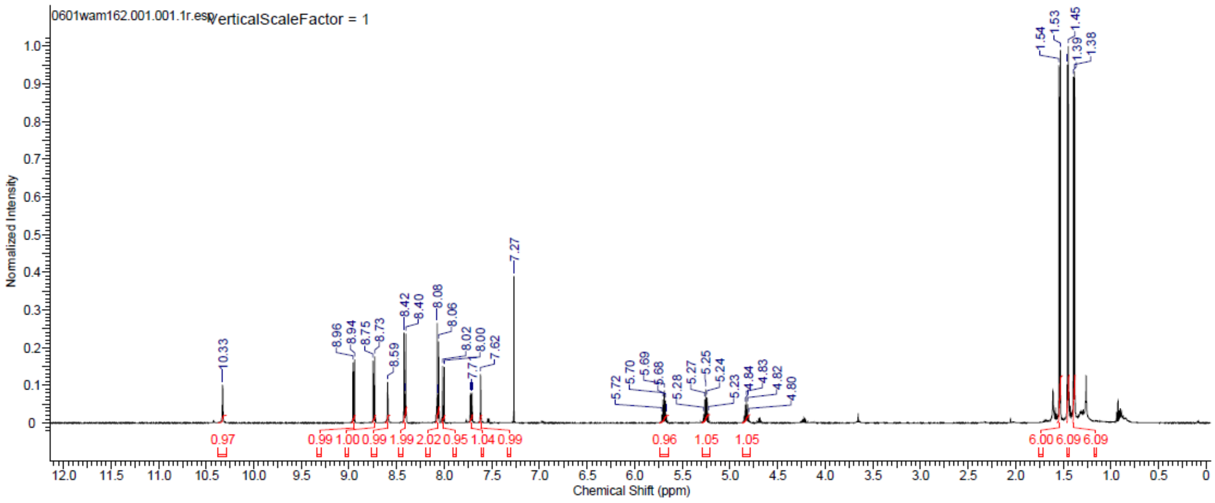
0601wam162.001.001.1r.esp
VerticalScaleFactor = 1
Normalized Intensity
Chemical Shift (ppm)
10.33
8.96
8.94
8.75
8.73
8.59
8.42
8.40
8.08
8.06
8.02
8.00
7.71
7.62
7.27
5.72
5.70
5.69
5.68
5.28
5.27
5.25
5.24
5.23
4.84
4.83
4.82
4.80
1.54
1.53
1.45
1.39
1.38
0.97
0.99
1.00
0.99
1.99
2.02
0.95
1.04
0.99
0.96
1.05
1.05
6.00
6.09
6.09

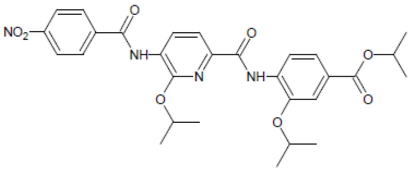
NO2
NH
N
O
NH
O
O
O
O
O

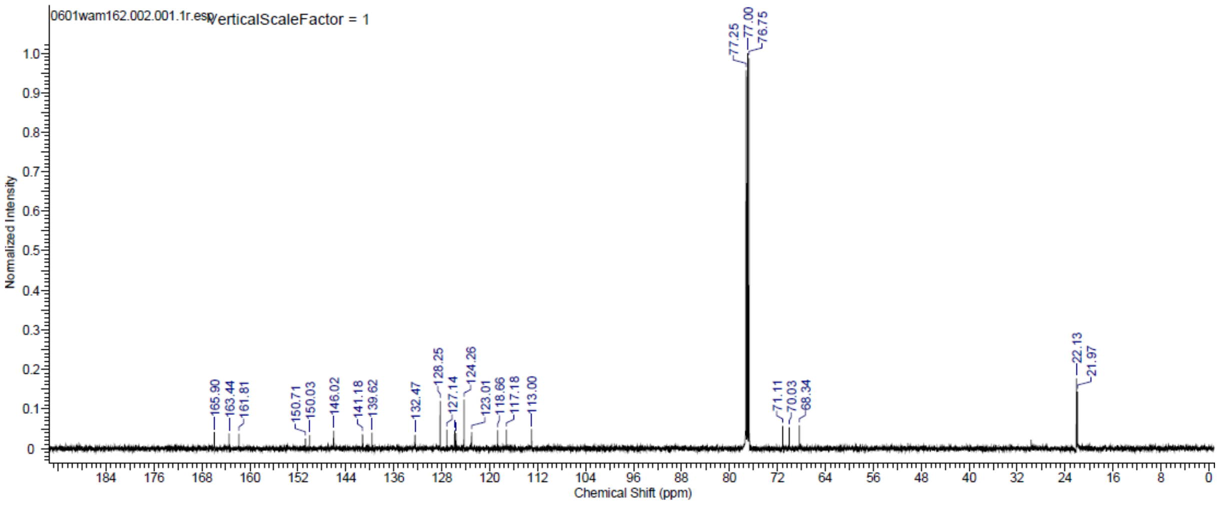
0601wam162.002.001.1r.esp
VerticalScaleFactor = 1
Normalized Intensity
Chemical Shift (ppm)
165.90
163.44
161.81
150.71
150.03
146.02
141.18
139.62
132.47
128.25
127.14
124.26
123.01
118.66
117.18
113.00
77.25
77.00
76.75
71.11
70.03
68.34
22.13
21.97

Compound **95**

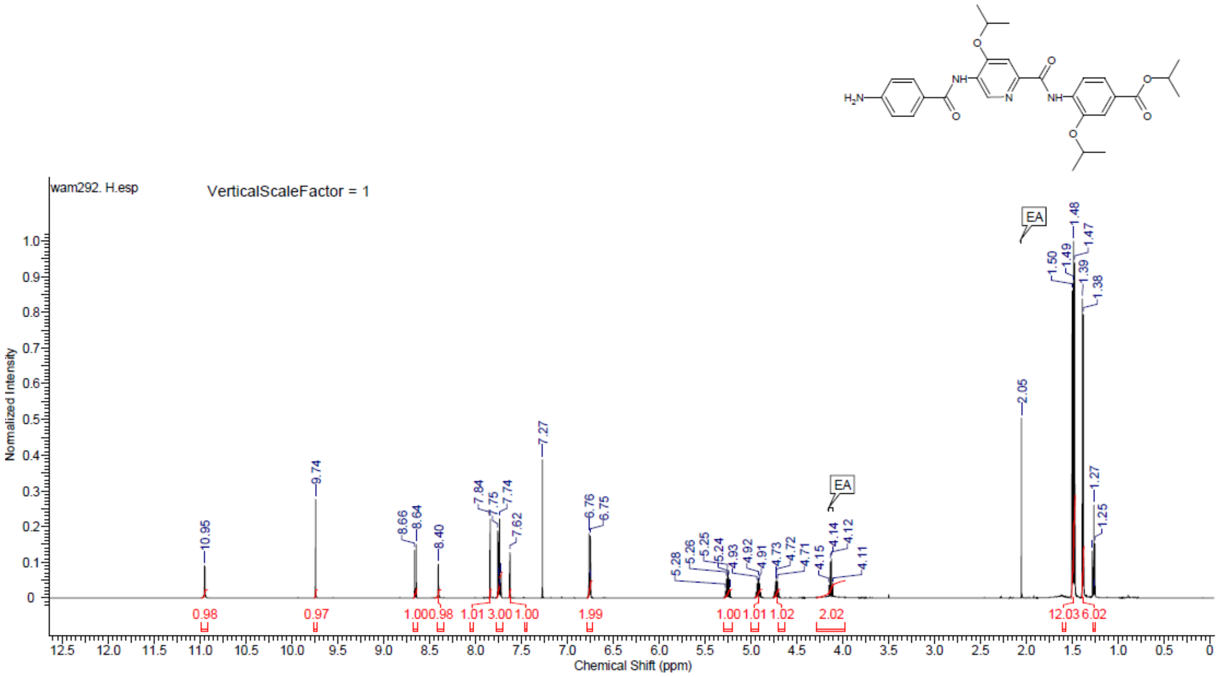

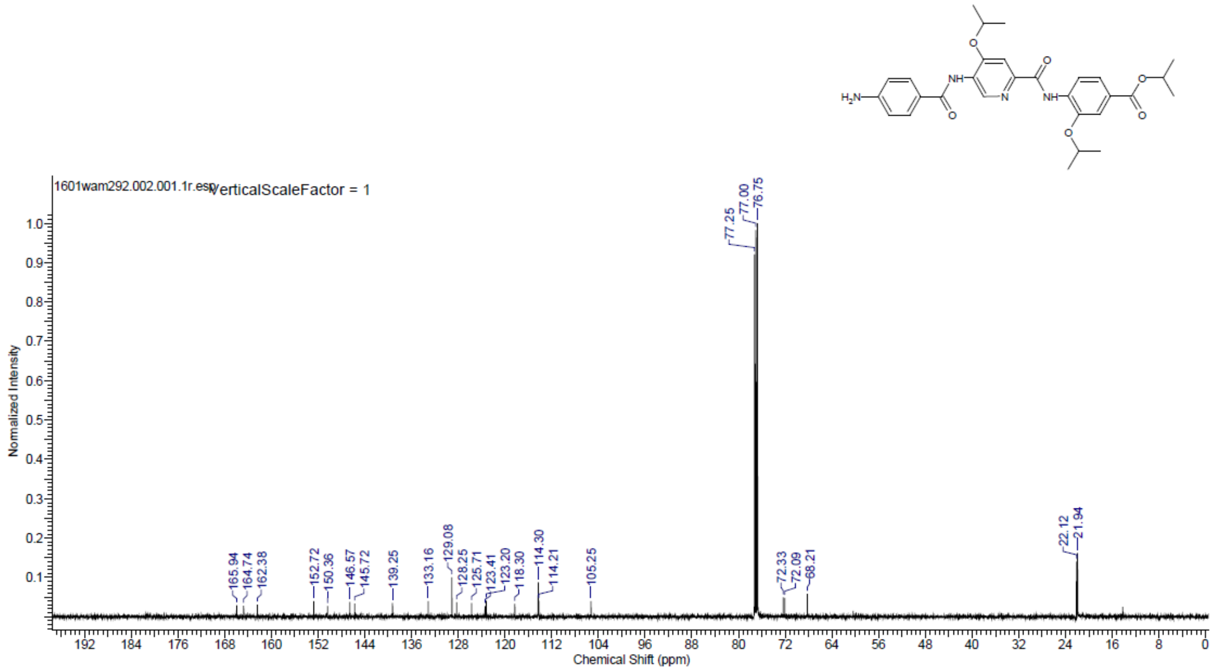

Compound **96**

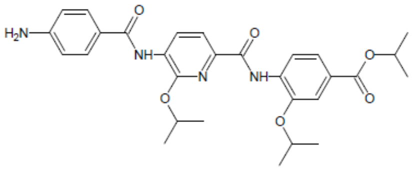
H2N
O
NH
N
O
NH
O
O
O

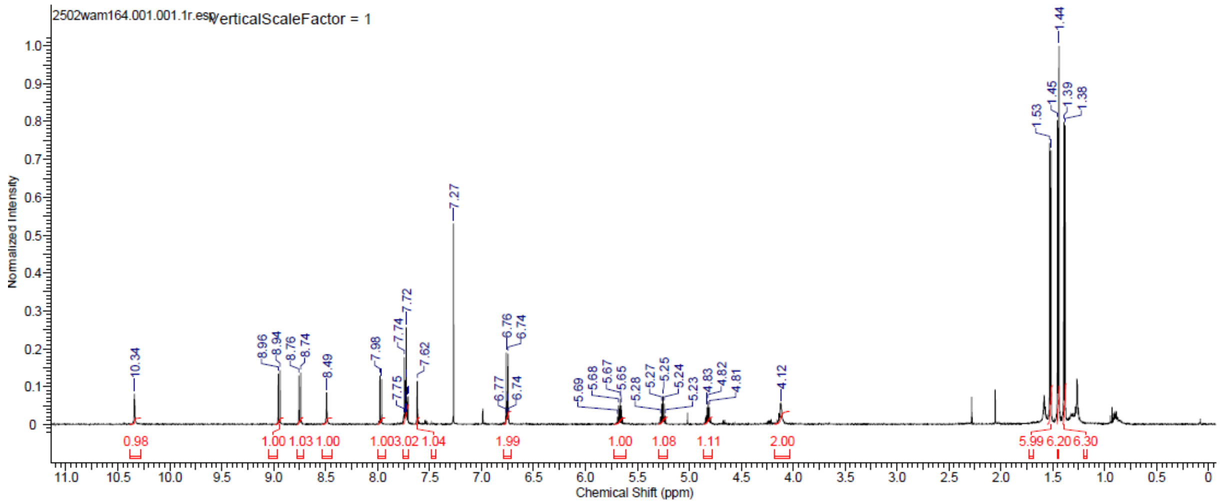
2502wam164.001.001.1r.esp VerticalScaleFactor = 1
Normalized Intensity
Chemical Shift (ppm)
10.34
8.96 8.94 8.76 8.74 8.49
7.98 7.75 7.74 7.72 7.62 7.27
6.77 6.76 6.74 6.74
5.69 5.68 5.67 5.65 5.28 5.27 5.25 5.24 5.23
4.83 4.82 4.81 4.12
1.53 1.45 1.44 1.39 1.38
0.98 1.00 1.03 1.00 1.00 3.02 1.04 1.99 1.00 1.08 1.11 2.00 5.99 6.20 6.30

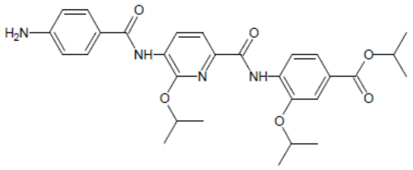
H2N
O
NH
N
O
NH
O
O
O

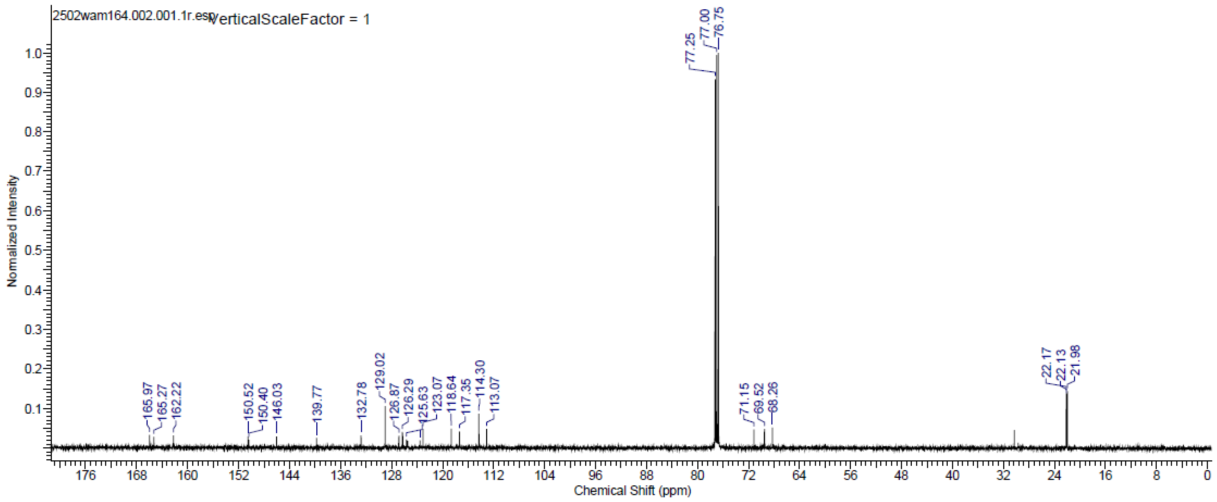
2502wam164.002.001.1r.esp VerticalScaleFactor = 1
Normalized Intensity
Chemical Shift (ppm)
165.97 165.27 162.22
150.52 150.40 146.03 139.77
132.78 129.02 126.87 126.29 125.63 123.07 118.64 117.35 114.30 113.07
77.25 77.00 76.75
71.15 69.52 68.26
22.17 22.13 21.98

Compound **2** (cystobactamid 507) (DMSO-d$_6$)

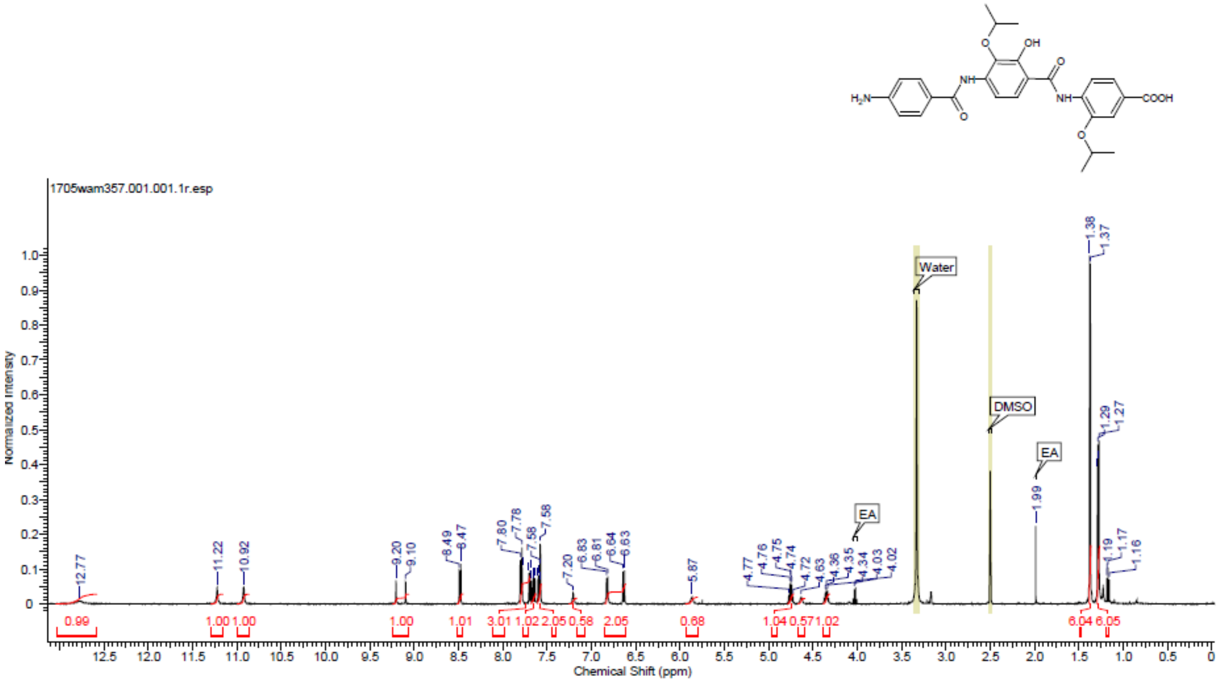

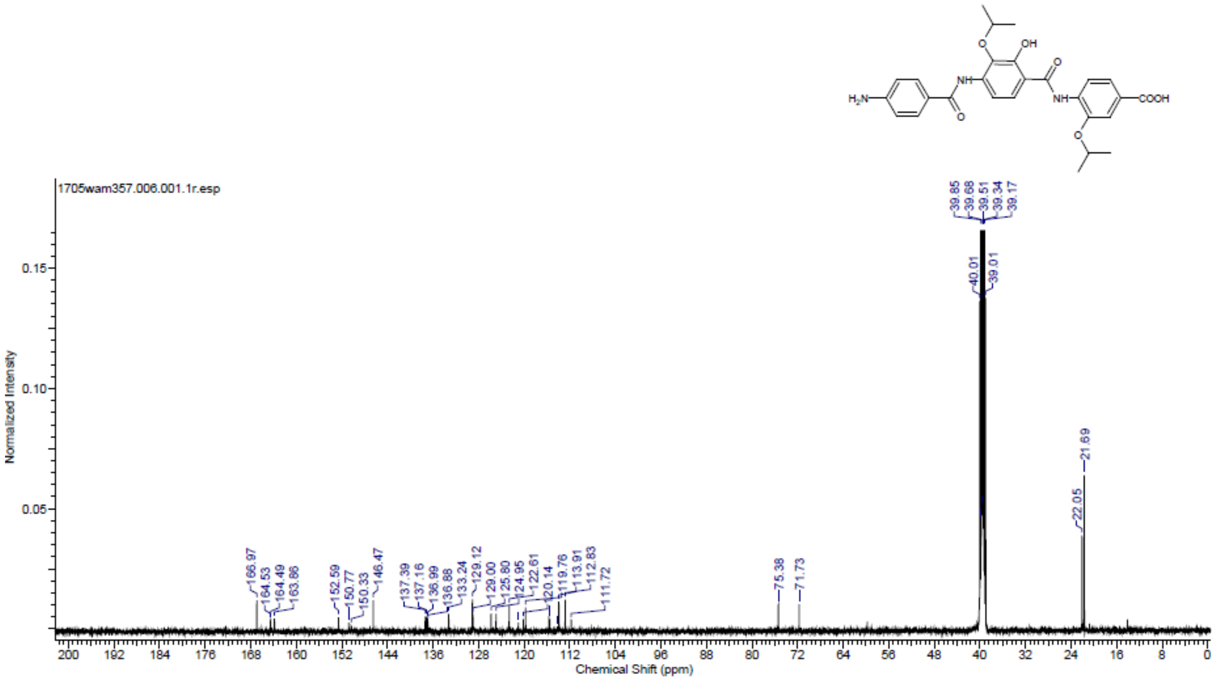

Compound **2** (cystobactamid 507) (acetone-d_6)

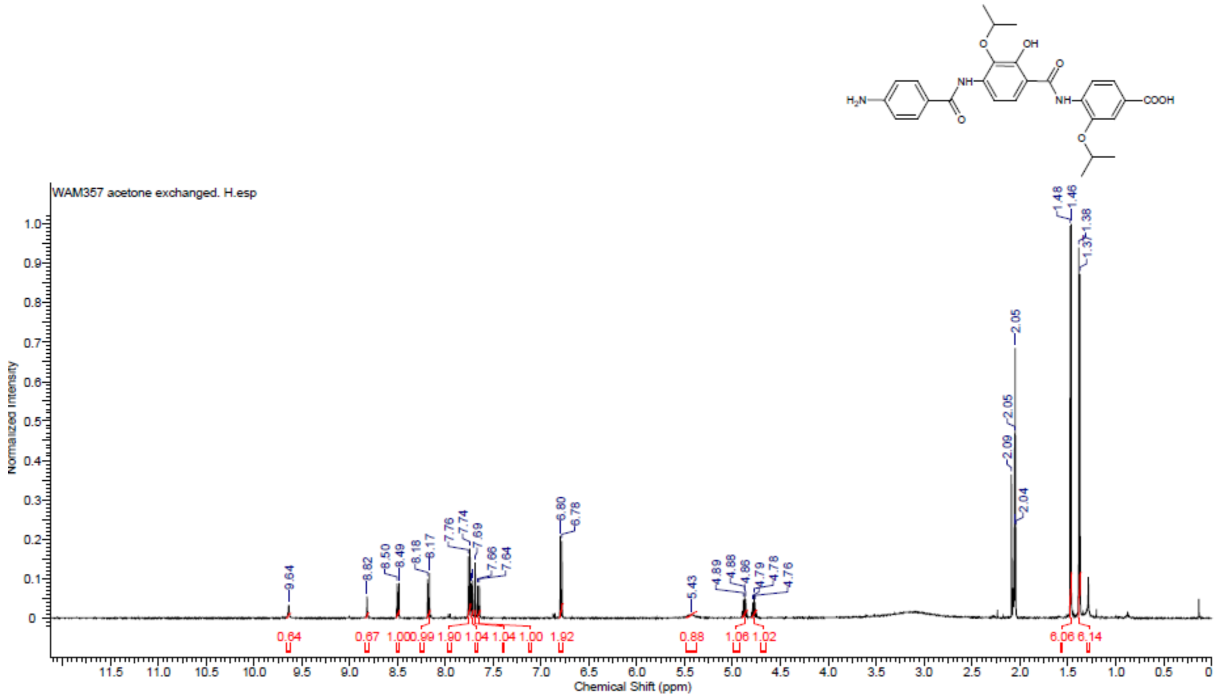

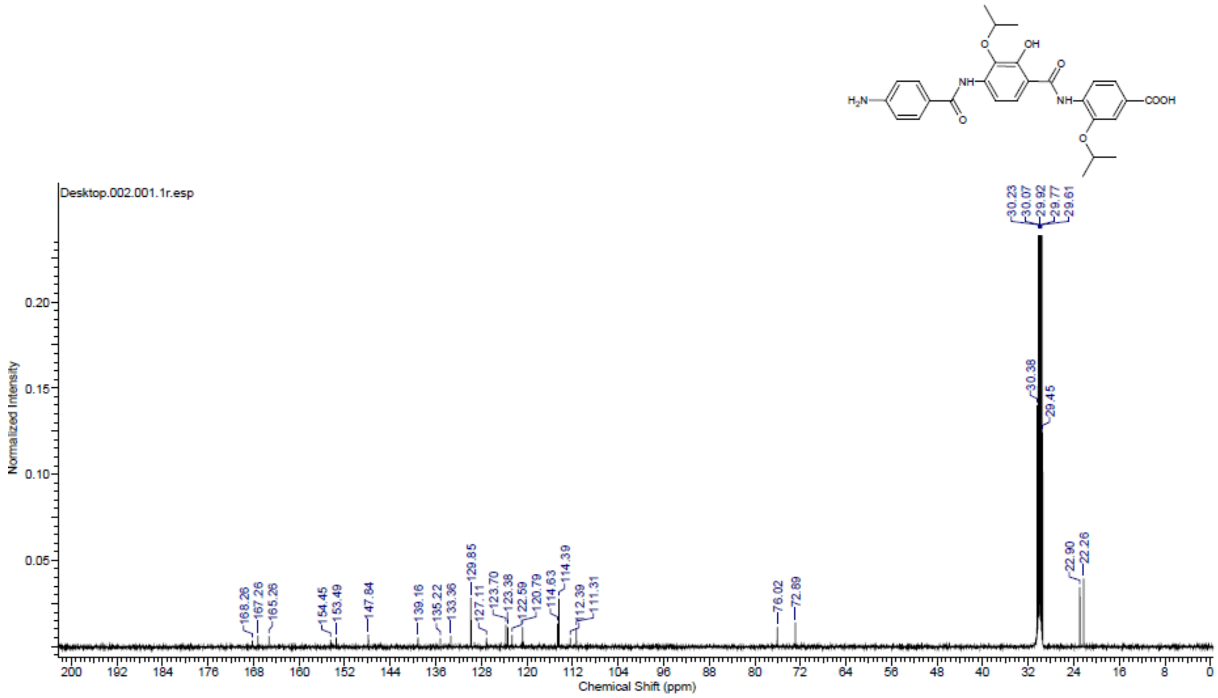

Compound **4**

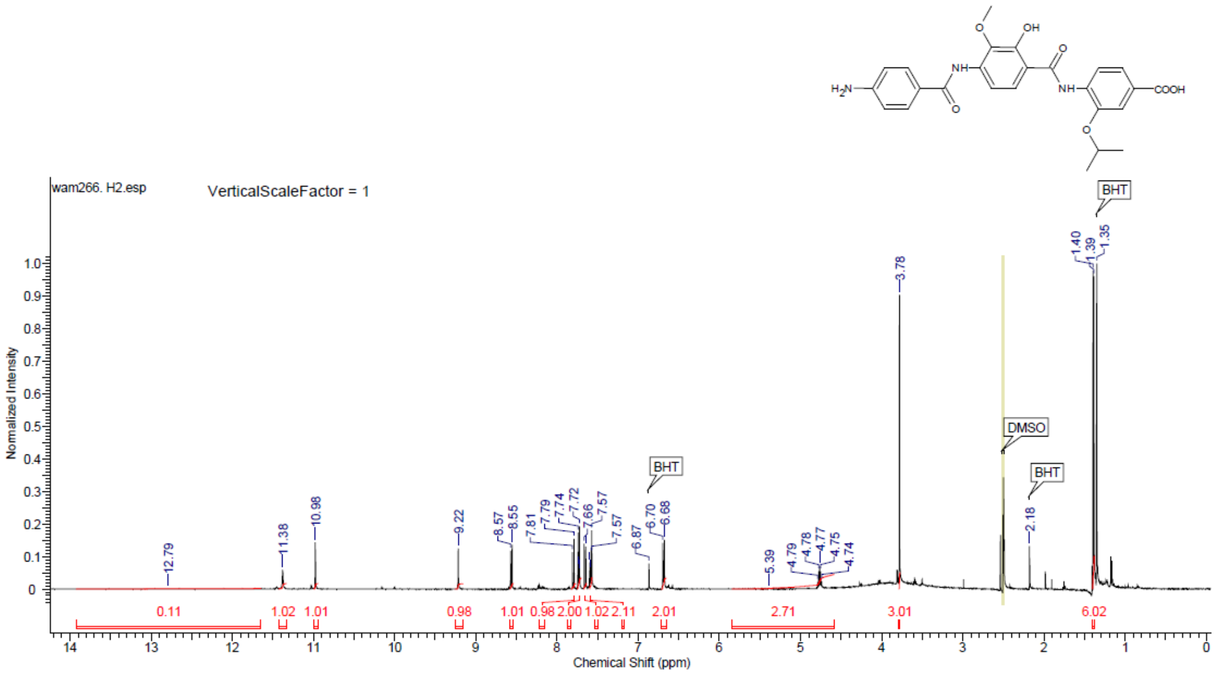

H₂N
NH
O
OH
NH
COOH
wam266. H2.esp
VerticalScaleFactor = 1
BHT
DMSO
12.79
11.38
10.98
9.22
8.57
8.55
7.81
7.79
7.74
7.72
7.66
7.57
7.57
6.87
6.70
6.68
5.39
4.79
4.78
4.77
4.75
4.74
3.78
2.18
1.40
1.39
1.35
0.11
1.02
1.01
0.98
1.01
0.98
2.00
1.02
2.11
2.01
2.71
3.01
6.02
Normalized Intensity
Chemical Shift (ppm)

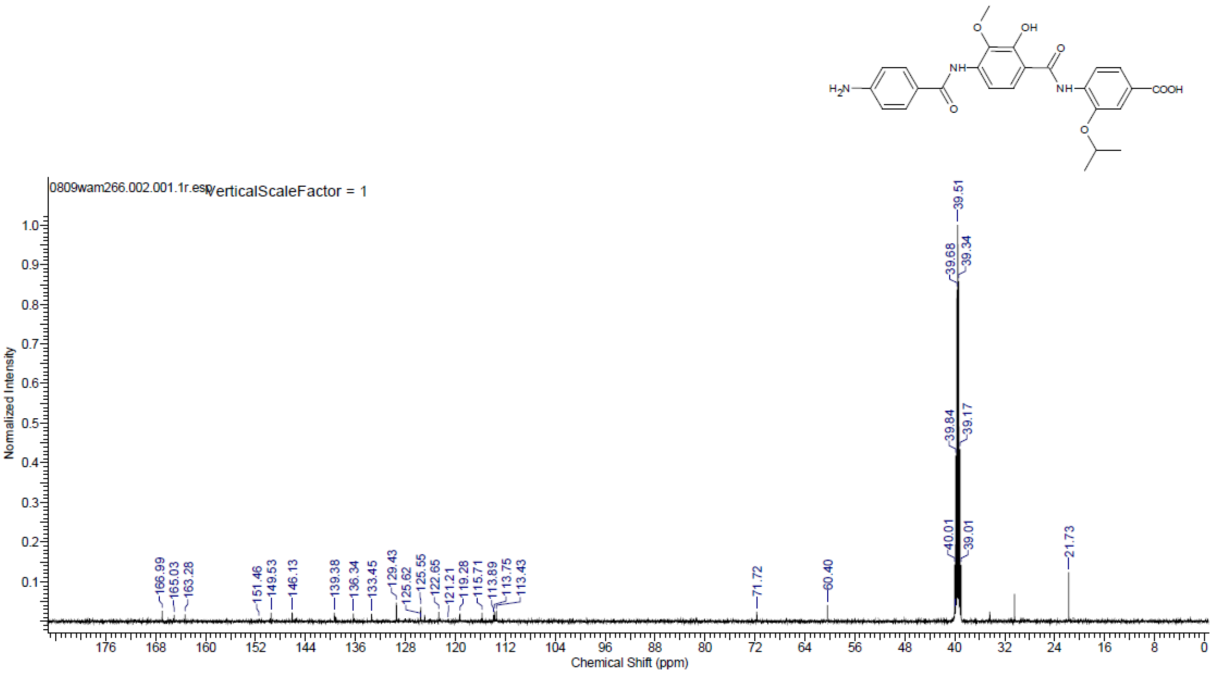

H₂N
NH
O
OH
NH
COOH
0809wam266.002.001.1r.esp
VerticalScaleFactor = 1
166.99
165.03
163.28
151.46
149.53
146.13
139.38
136.34
133.45
129.43
125.62
125.55
122.65
121.21
119.28
115.71
113.89
113.75
113.43
71.72
60.40
40.01
39.84
39.68
39.51
39.34
39.17
39.01
21.73
Normalized Intensity
Chemical Shift (ppm)

Compound **5**

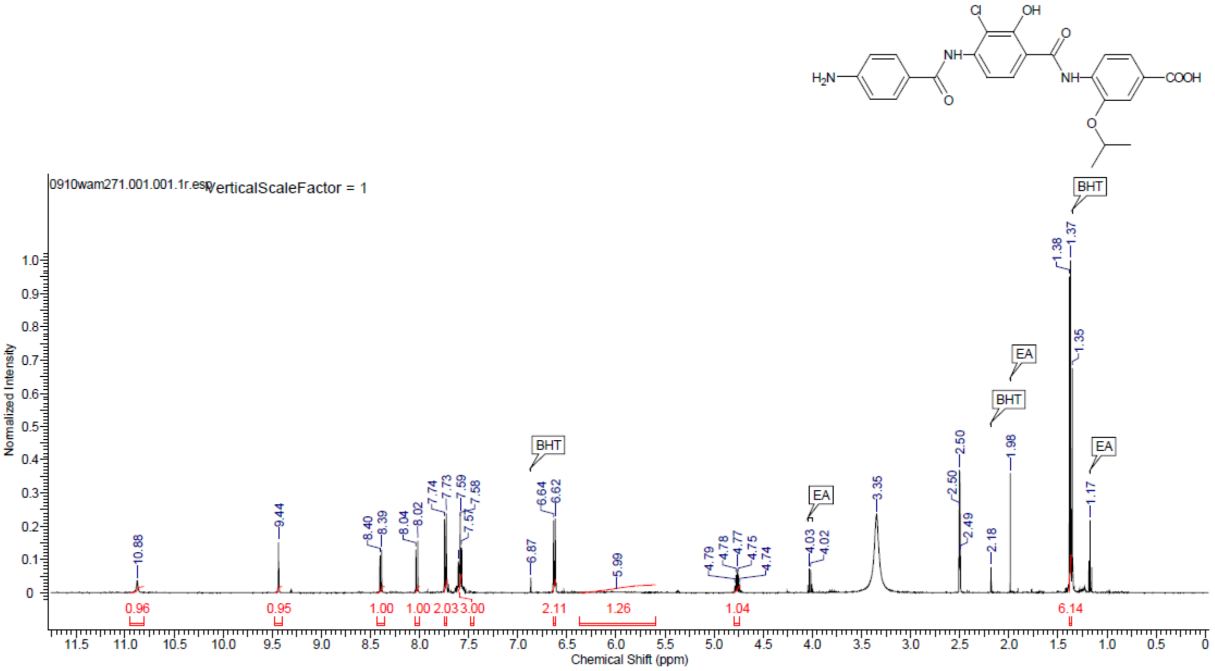
Cl
OH
O
NH
H2N
O
NH
COOH
O
0910wam271.001.001.1r.esp
VerticalScaleFactor = 1
Normalized Intensity
Chemical Shift (ppm)
BHT
EA
10.88
9.44
8.40
8.39
8.04
8.02
7.74
7.73
7.59
7.58
7.57
6.87
6.64
6.62
5.99
4.79
4.78
4.77
4.75
4.74
4.03
4.02
3.35
2.50
2.50
2.49
2.18
1.98
1.38
1.37
1.35
1.17
0.96
0.95
1.00
1.00
2.03
3.00
2.11
1.26
1.04
6.14

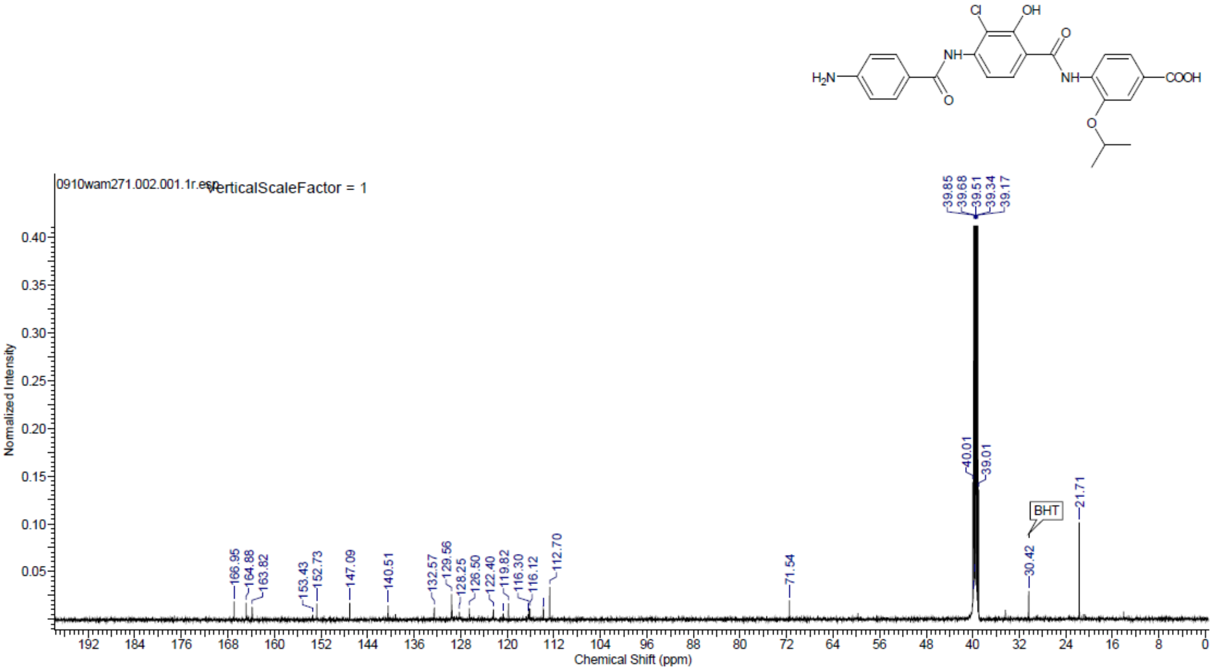
Cl
OH
O
NH
H2N
O
NH
COOH
O
0910wam271.002.001.1r.esp
VerticalScaleFactor = 1
Normalized Intensity
Chemical Shift (ppm)
BHT
166.95
164.88
163.82
153.43
152.73
147.09
140.51
132.57
129.56
128.25
126.50
122.40
119.82
116.30
116.12
112.70
71.54
39.85
39.68
39.51
39.34
39.17
40.01
39.01
30.42
21.71

Compound **6**

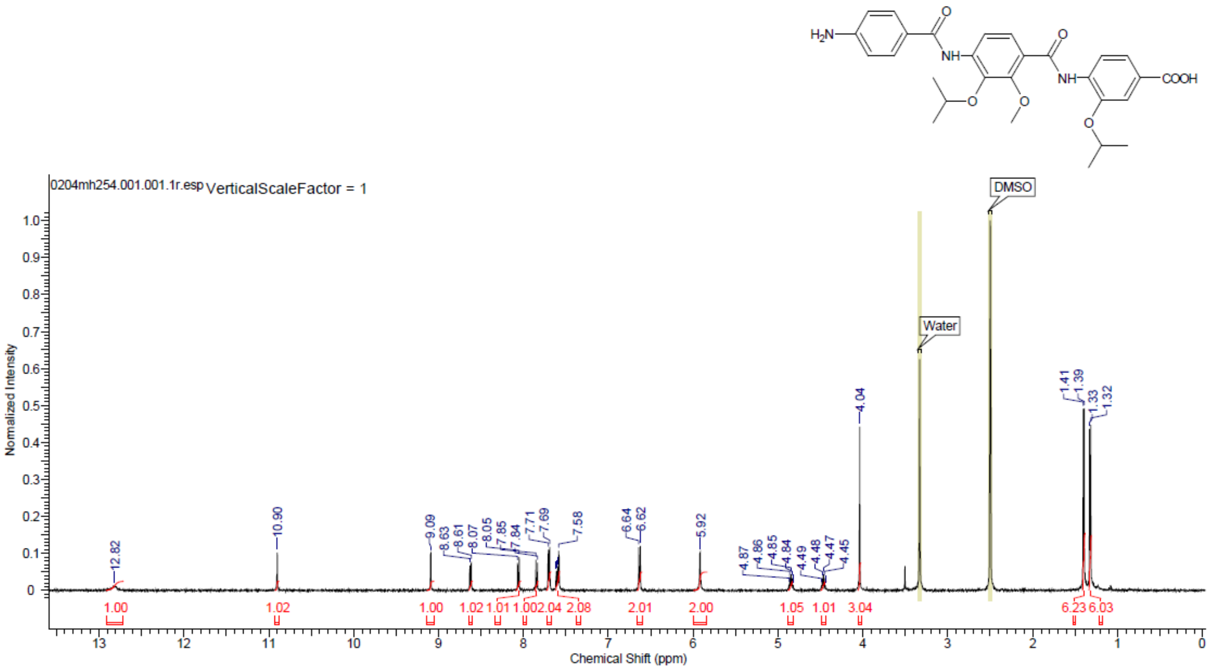
H2N
NH
O
COOH
0204mh254.001.001.1r.esp VerticalScaleFactor = 1
DMSO
Water
Normalized Intensity
Chemical Shift (ppm)
12.82
10.90
9.09
8.63
8.61
8.07
8.05
7.85
7.84
7.71
7.69
7.58
6.64
6.62
5.92
4.87
4.86
4.85
4.84
4.49
4.48
4.47
4.45
4.04
1.41
1.39
1.33
1.32
1.00
1.02
1.00
1.02
1.01
1.00
2.04
2.08
2.01
2.00
1.05
1.01
3.04
6.23
6.03

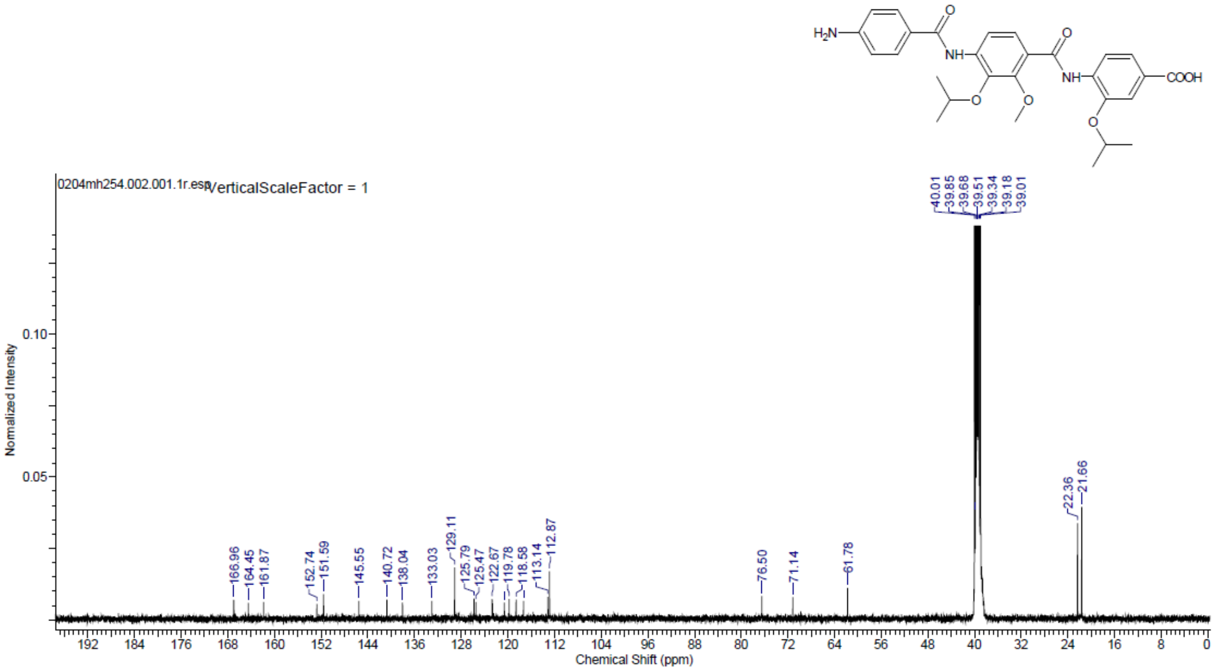
H2N
NH
O
COOH
0204mh254.002.001.1r.esp VerticalScaleFactor = 1
Normalized Intensity
Chemical Shift (ppm)
166.96
164.45
161.87
152.74
151.59
145.55
140.72
138.04
133.03
129.11
125.79
125.47
122.67
119.78
118.58
113.14
112.87
76.50
71.14
61.78
40.01
39.85
39.68
39.51
39.34
39.18
39.01
22.36
21.66

Compound **7**

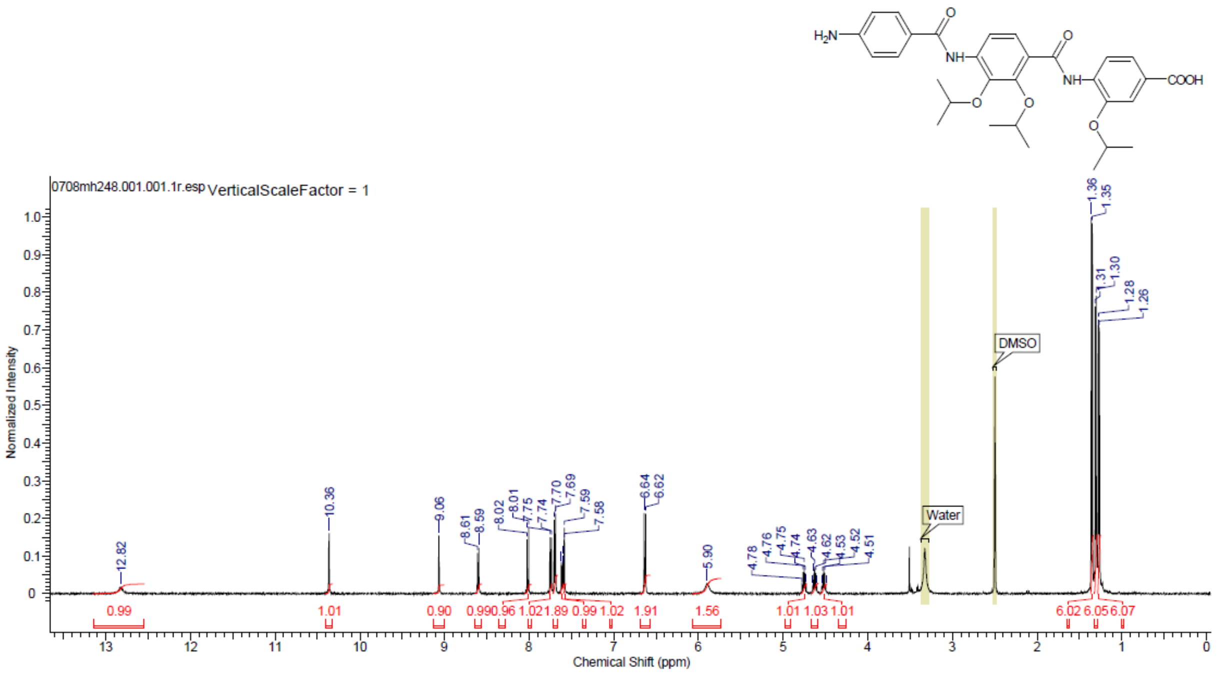

0708mh248.001.001.1r.esp VerticalScaleFactor = 1
DMSO
Water
Normalized Intensity
Chemical Shift (ppm)

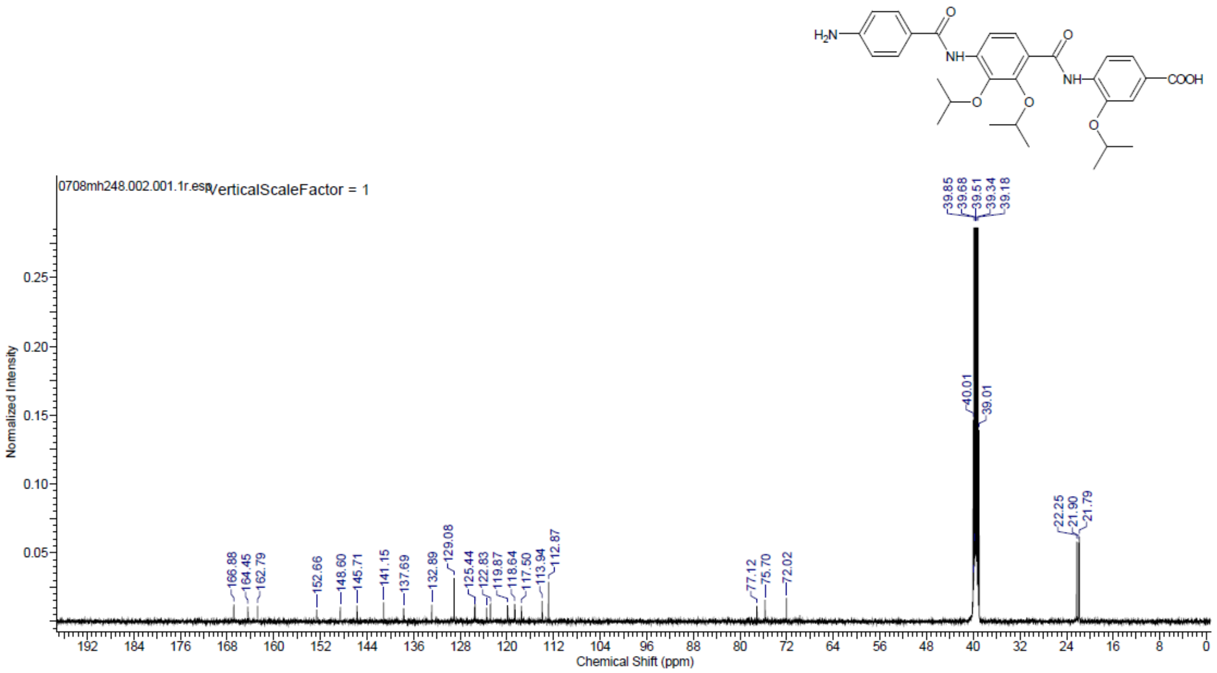

0708mh248.002.001.1r.esp VerticalScaleFactor = 1
Normalized Intensity
Chemical Shift (ppm)

Compound **8**

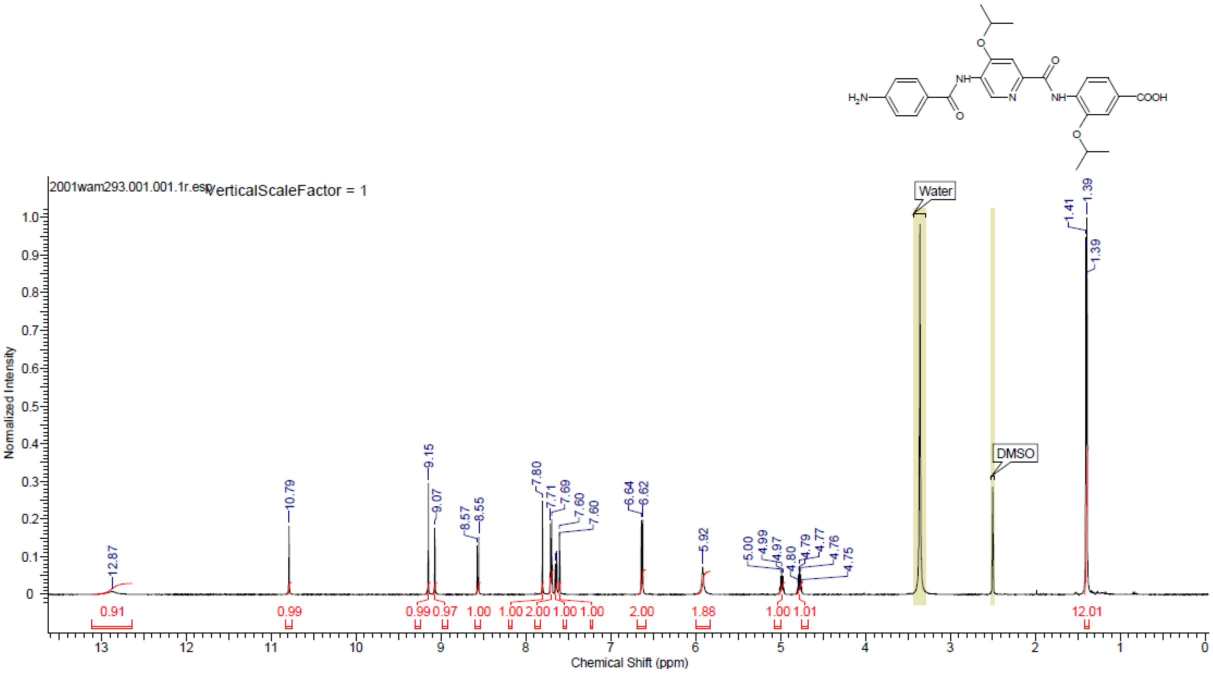

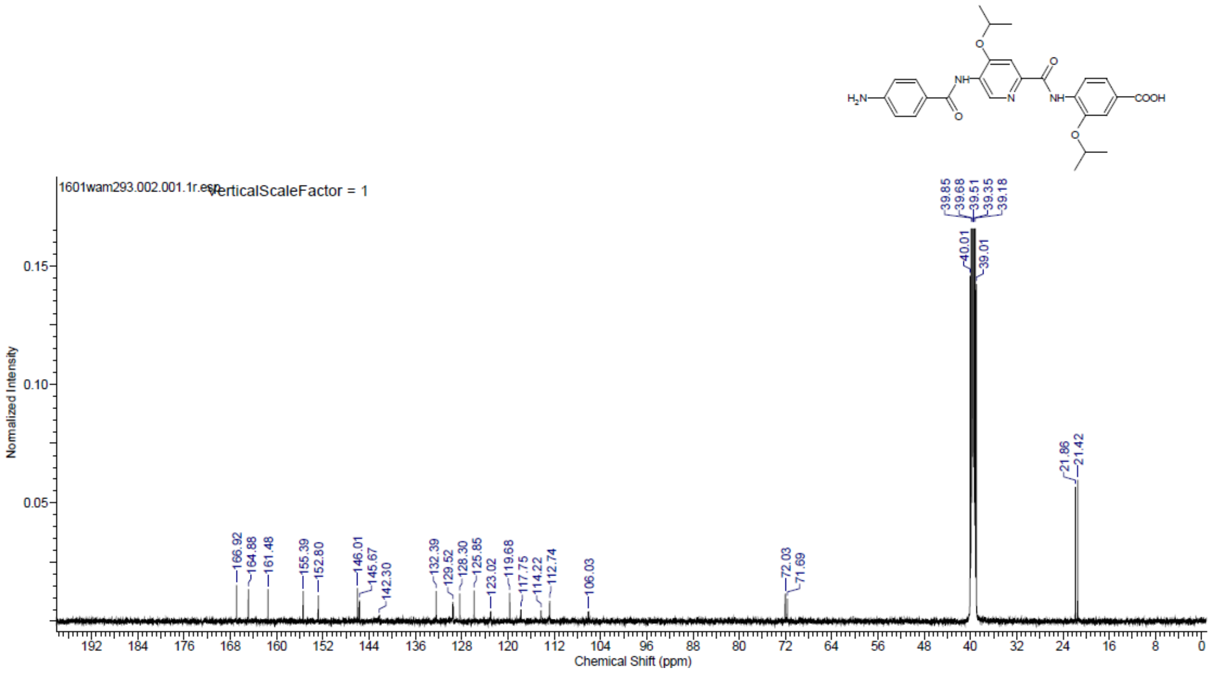

Compound **9**

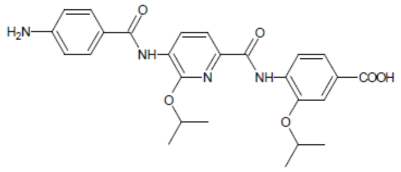
H2N
O
NH
N
O
NH
COOH
O
O

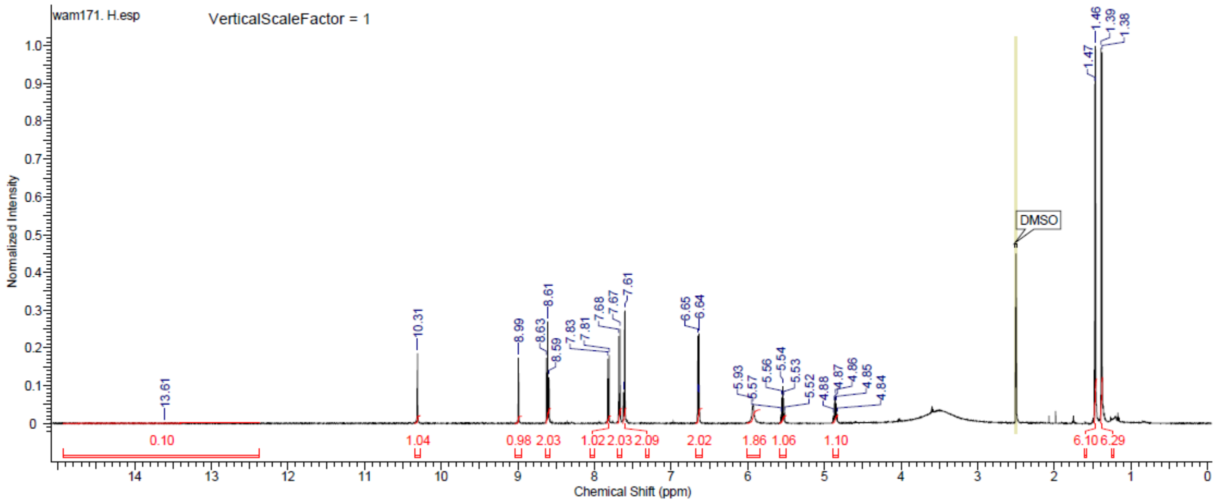
wam171. H.esp
VerticalScaleFactor = 1
Normalized Intensity
Chemical Shift (ppm)
DMSO
13.61
10.31
8.99
8.63
8.61
8.59
7.83
7.81
7.68
7.67
7.61
6.65
6.64
5.93
5.57
5.56
5.54
5.53
5.52
4.88
4.87
4.86
4.85
4.84
1.47
1.46
1.39
1.38
0.10
1.04
0.98
2.03
1.02
2.03
2.09
2.02
1.86
1.06
1.10
6.10
6.29

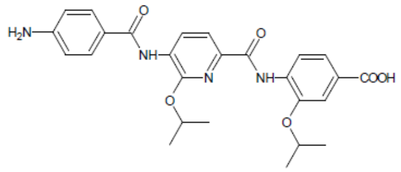
H2N
O
NH
N
O
NH
COOH
O
O

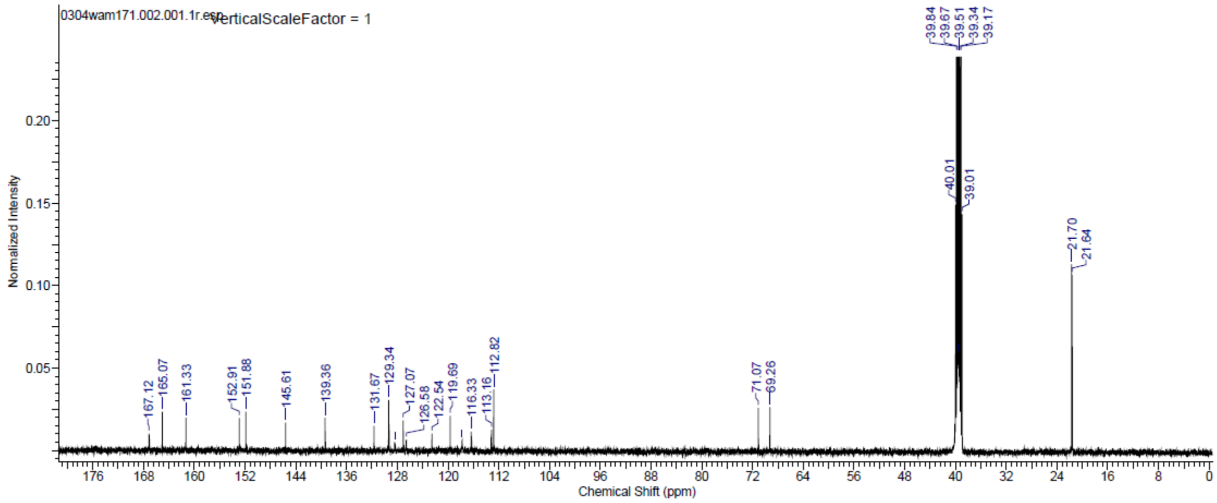
0304wam171.002.001.1r.esp
VerticalScaleFactor = 1
Normalized Intensity
Chemical Shift (ppm)
167.12
165.07
161.33
152.91
151.88
145.61
139.36
131.67
129.34
127.07
126.58
122.54
119.69
116.33
113.16
112.82
71.07
69.26
40.01
39.84
39.67
39.51
39.34
39.17
39.01
21.70
21.64

Compound **10**

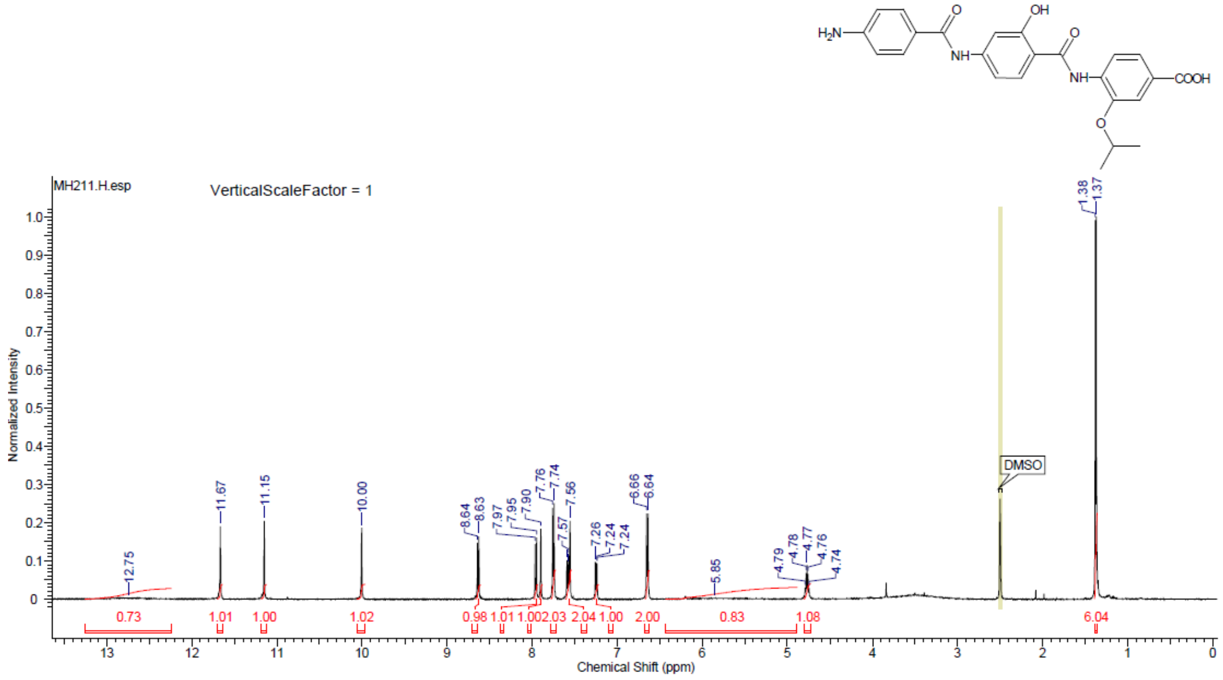
H2N
O
NH
OH
O
NH
COOH
O
MH211.H.esp
VerticalScaleFactor = 1
Normalized Intensity
Chemical Shift (ppm)
DMSO
12.75
11.67
11.15
10.00
8.64
8.63
7.97
7.95
7.90
7.76
7.74
7.57
7.56
7.26
7.24
7.24
6.66
6.64
5.85
4.79
4.78
4.77
4.76
4.74
1.38
1.37
0.73
1.01
1.00
1.02
0.98
1.01
1.00
2.03
2.04
1.00
2.00
0.83
1.08
6.04

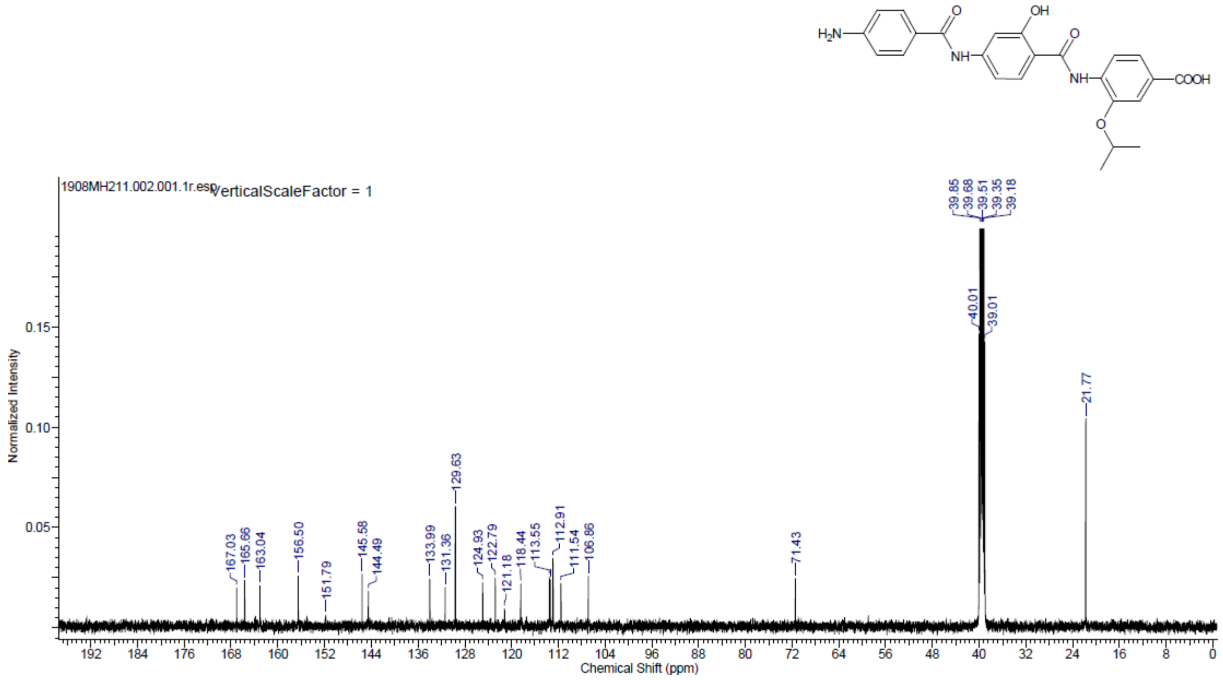
H2N
O
NH
OH
O
NH
COOH
O
1908MH211.002.001.1r.esp
VerticalScaleFactor = 1
Normalized Intensity
Chemical Shift (ppm)
167.03
165.66
163.04
156.50
151.79
145.58
144.49
133.99
131.36
129.63
124.93
122.79
121.18
118.44
113.55
112.91
111.54
106.86
71.43
40.01
39.85
39.68
39.51
39.35
39.18
39.01
21.77

Compound **11**

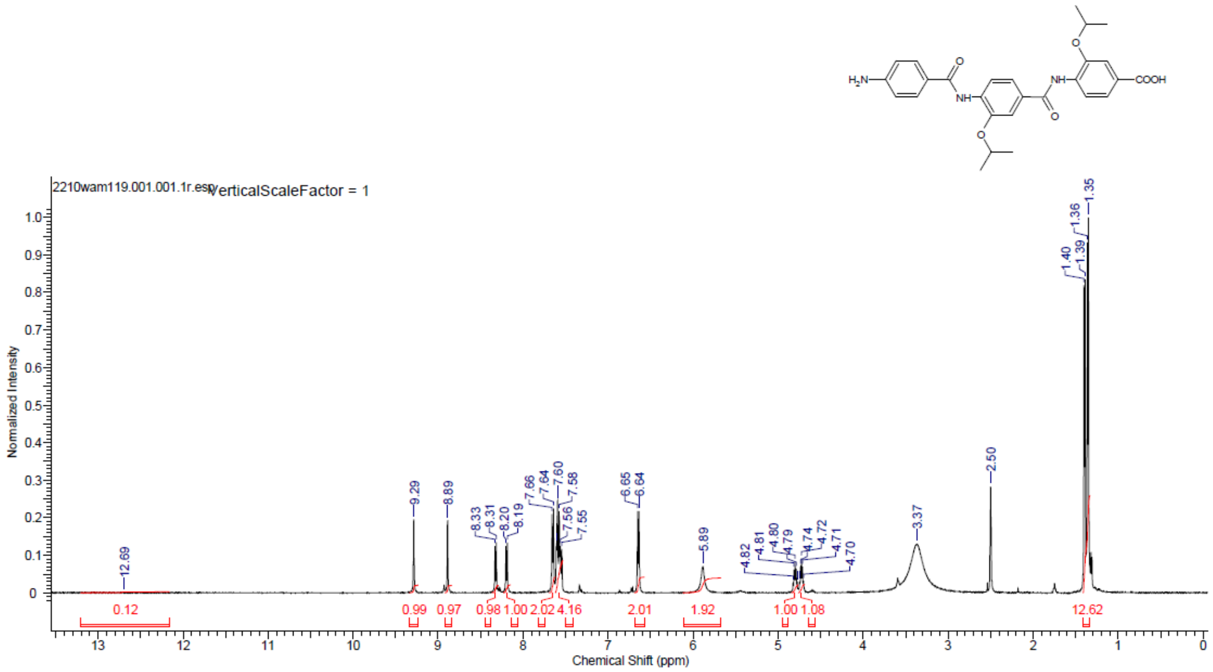

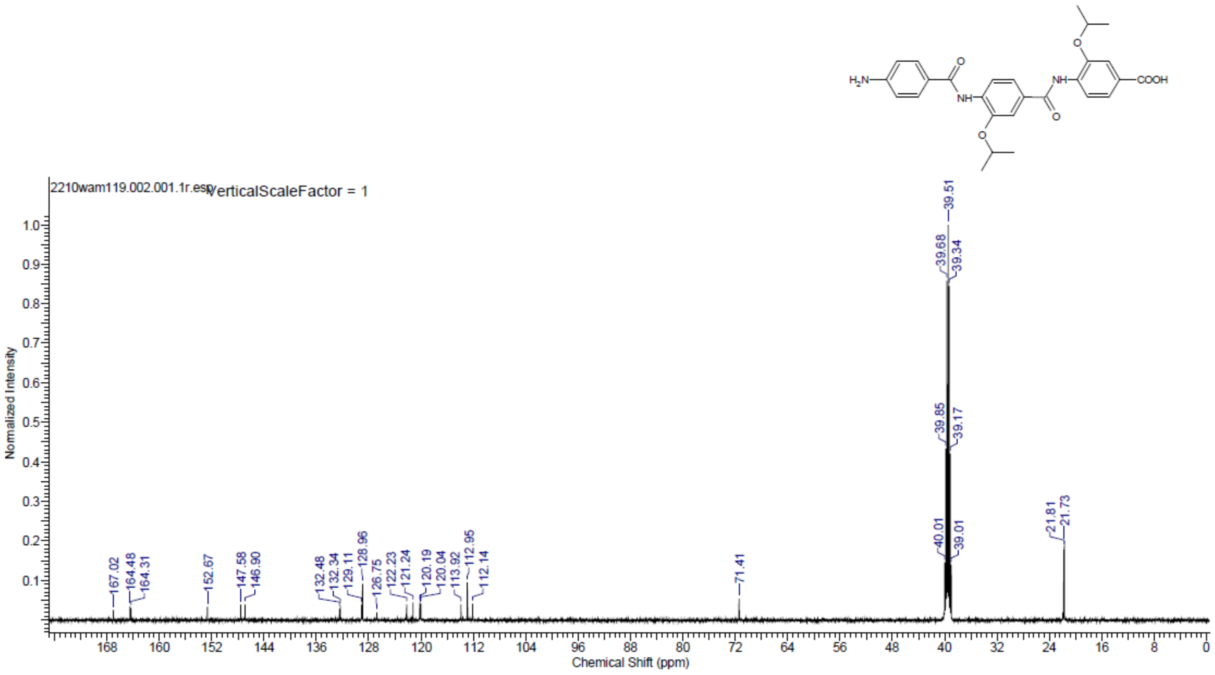

Compound **12**

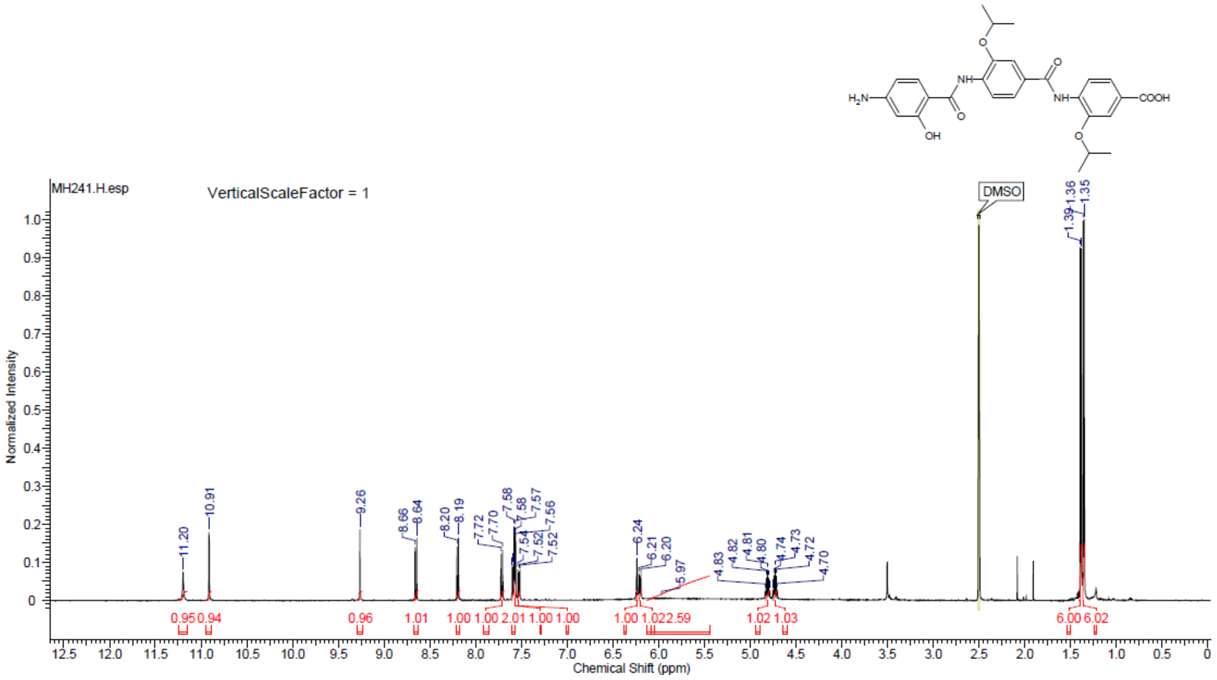
H2N
NH
O
OH
COOH
MH241.H.esp
VerticalScaleFactor = 1
DMSO
Normalized Intensity
Chemical Shift (ppm)
11.20
10.91
9.26
8.66
8.64
8.20
8.19
7.72
7.70
7.58
7.58
7.57
7.56
7.54
7.52
7.52
6.24
6.21
6.20
5.97
4.83
4.82
4.81
4.80
4.74
4.73
4.72
4.70
1.39
1.36
1.35
0.95
0.94
0.96
1.01
1.00
1.00
2.01
1.00
1.00
1.00
1.02
2.59
1.02
1.03
6.00
6.02

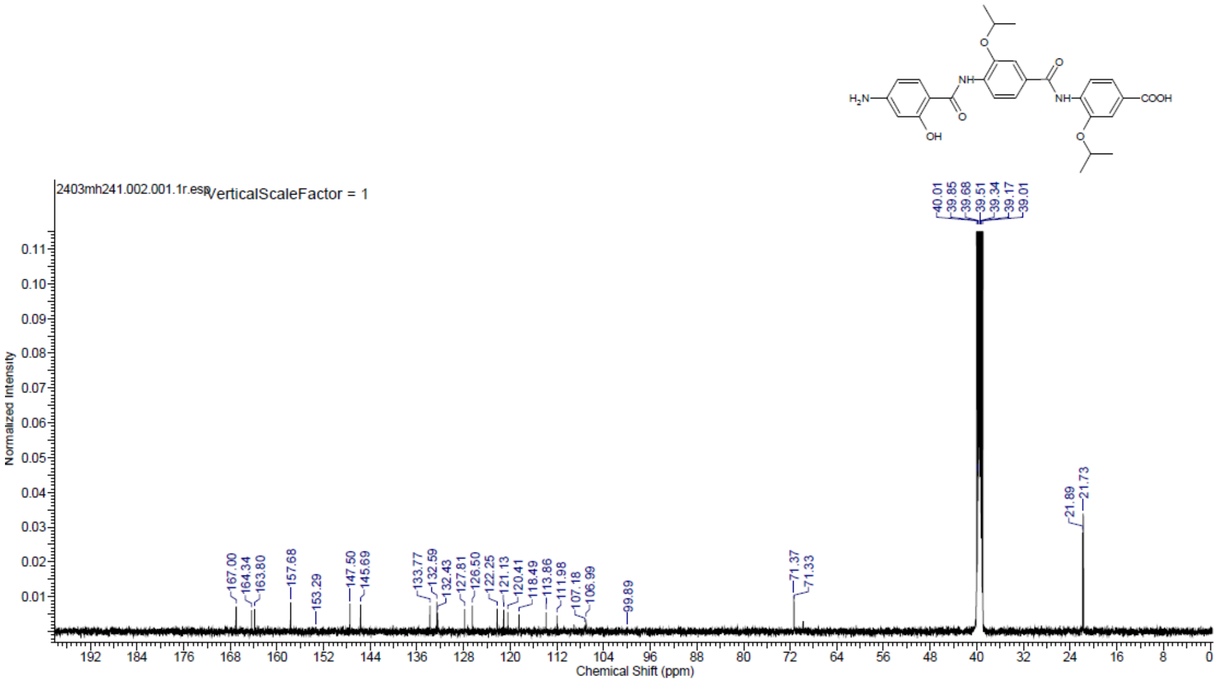
H2N
NH
O
OH
COOH
2403mh241.002.001.1r.esp
VerticalScaleFactor = 1
Normalized Intensity
Chemical Shift (ppm)
167.00
164.34
163.80
157.68
153.29
147.50
145.69
133.77
132.59
132.43
127.81
126.50
122.25
121.13
120.41
118.49
113.86
111.98
107.18
106.99
99.89
71.37
71.33
40.01
39.85
39.68
39.51
39.34
39.17
39.01
21.89
21.73

Compound **13**

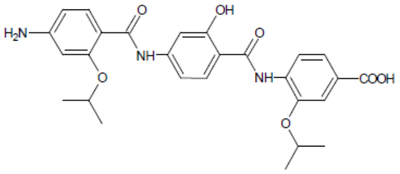
H2N
O
NH
OH
O
NH
COOH
O
O

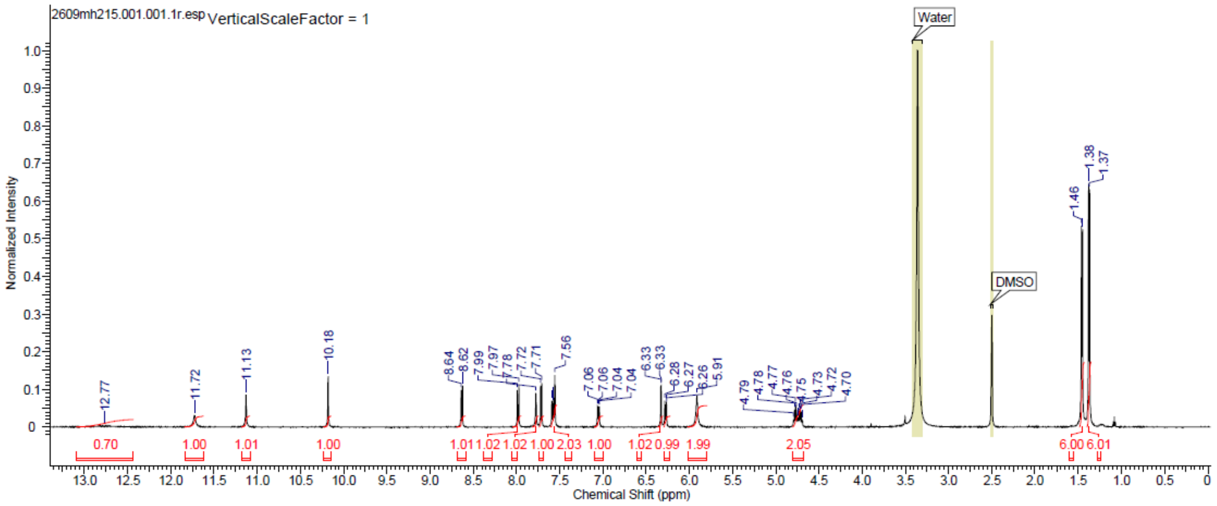
2609mh215.001.001.1r.esp VerticalScaleFactor = 1
Water
DMSO
Normalized Intensity
Chemical Shift (ppm)
12.77
11.72
11.13
10.18
8.64
8.62
7.99
7.97
7.78
7.72
7.71
7.56
7.06
7.06
7.04
7.04
6.33
6.33
6.28
6.27
6.26
5.91
4.79
4.78
4.77
4.76
4.75
4.73
4.72
4.70
1.46
1.38
1.37
0.70
1.00
1.01
1.00
1.01
1.02
1.02
1.00
2.03
1.00
1.02
0.99
1.99
2.05
6.00
6.01

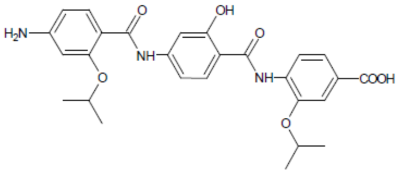
H2N
O
NH
OH
O
NH
COOH
O
O

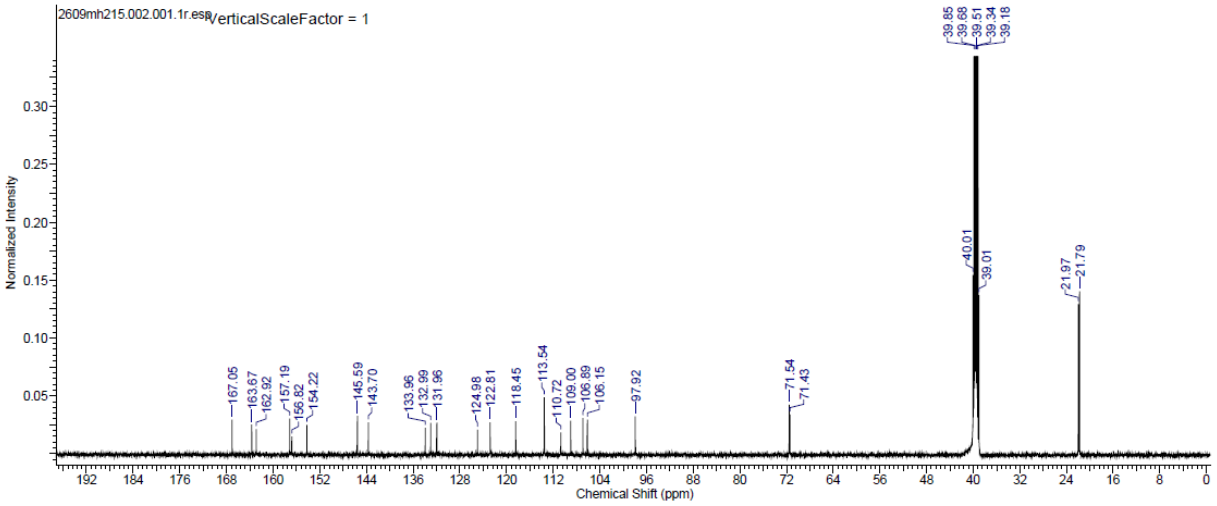
2609mh215.002.001.1r.esp VerticalScaleFactor = 1
Normalized Intensity
Chemical Shift (ppm)
167.05
163.67
162.92
157.19
156.82
154.22
145.59
143.70
133.96
132.99
131.96
124.98
122.81
118.45
113.54
110.72
109.00
106.89
106.15
97.92
71.54
71.43
39.85
39.68
39.51
39.34
39.18
40.01
39.01
21.97
21.79

Compound **14**

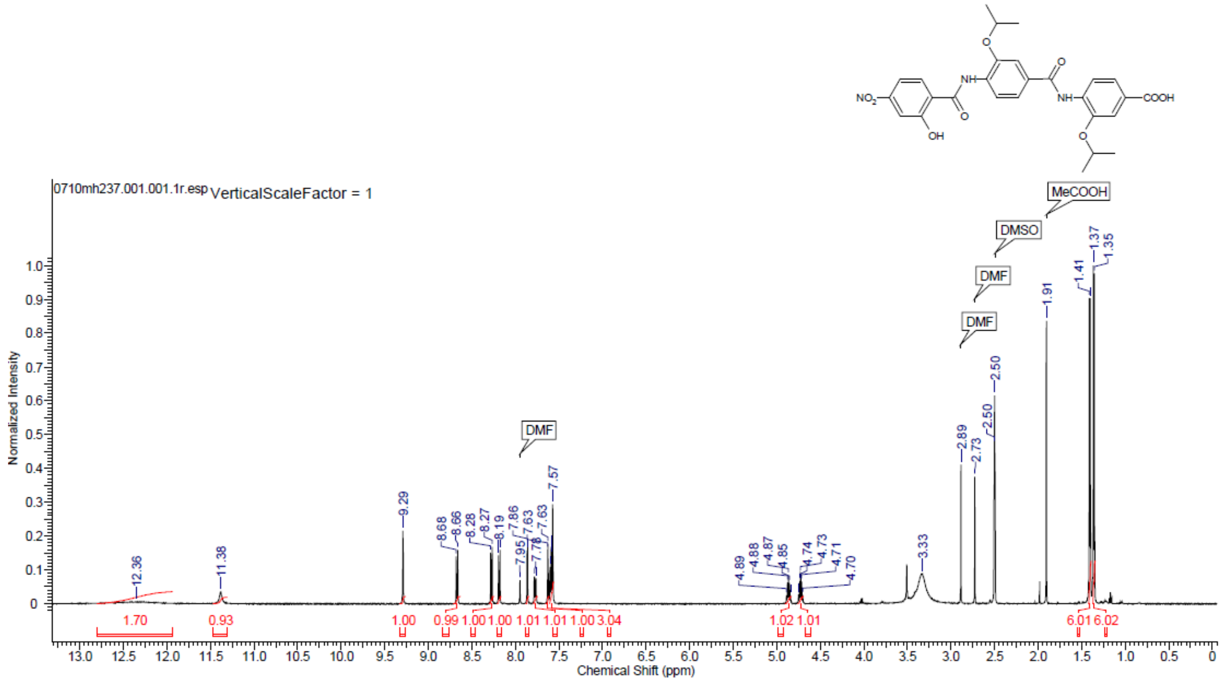

NO2
OH
NH
O
COOH
0710mh237.001.001.1r.esp VerticalScaleFactor = 1
Normalized Intensity
Chemical Shift (ppm)
DMF
DMSO
MeCOOH
12.36
11.38
9.29
8.68
8.66
8.28
8.27
8.19
7.95
7.86
7.78
7.63
7.63
7.57
4.89
4.88
4.87
4.85
4.74
4.73
4.71
4.70
3.33
2.89
2.73
2.50
2.50
1.91
1.41
1.37
1.35
1.70
0.93
1.00
0.99
1.00
1.00
1.01
1.01
1.00
3.04
1.02
1.01
6.01
6.02

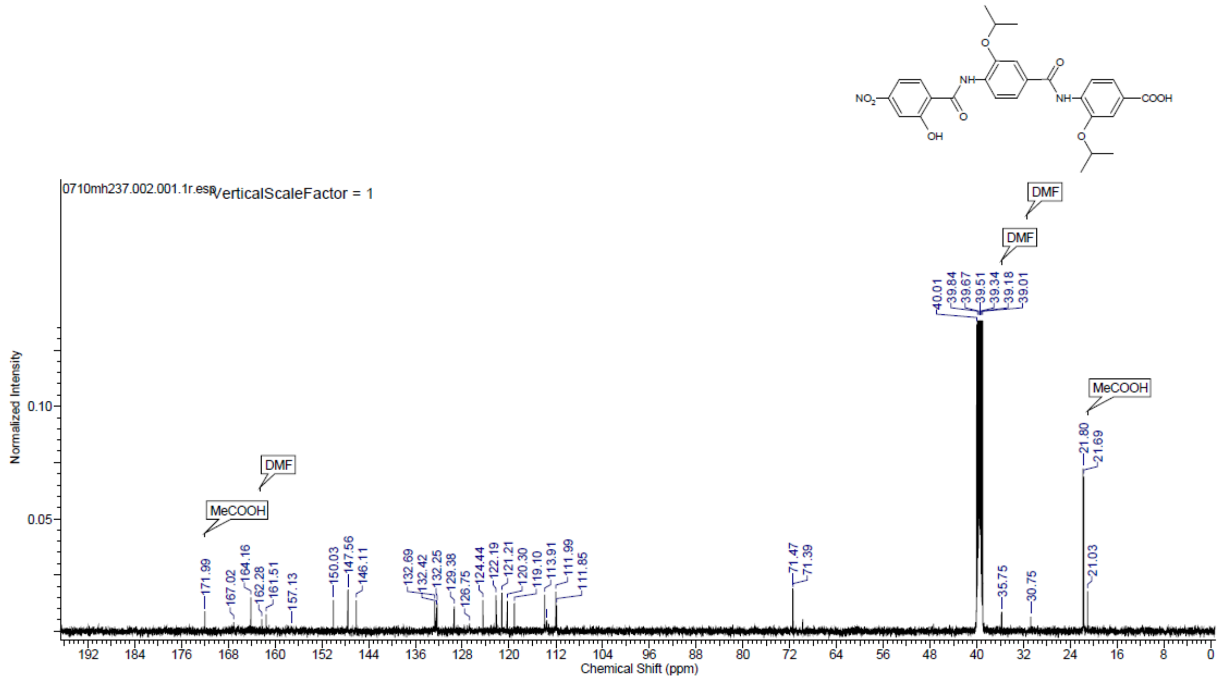

NO2
OH
NH
O
COOH
0710mh237.002.001.1r.esp VerticalScaleFactor = 1
Normalized Intensity
Chemical Shift (ppm)
DMF
MeCOOH
171.99
167.02
164.16
162.28
161.51
157.13
150.03
147.56
146.11
132.69
132.42
132.25
129.38
126.75
124.44
122.19
121.21
120.30
119.10
113.91
111.99
111.85
71.47
71.39
40.01
39.84
39.67
39.51
39.34
39.18
39.01
35.75
30.75
21.80
21.69
21.03

Compound **15**

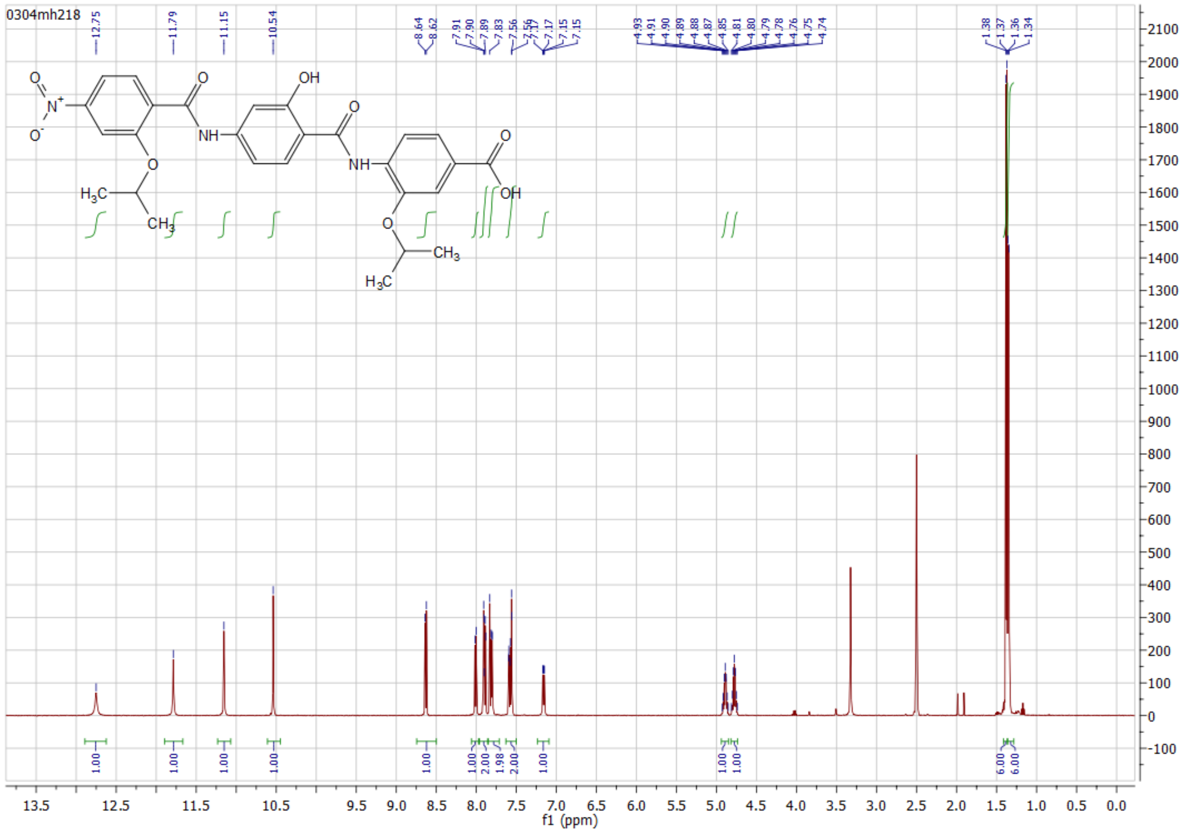

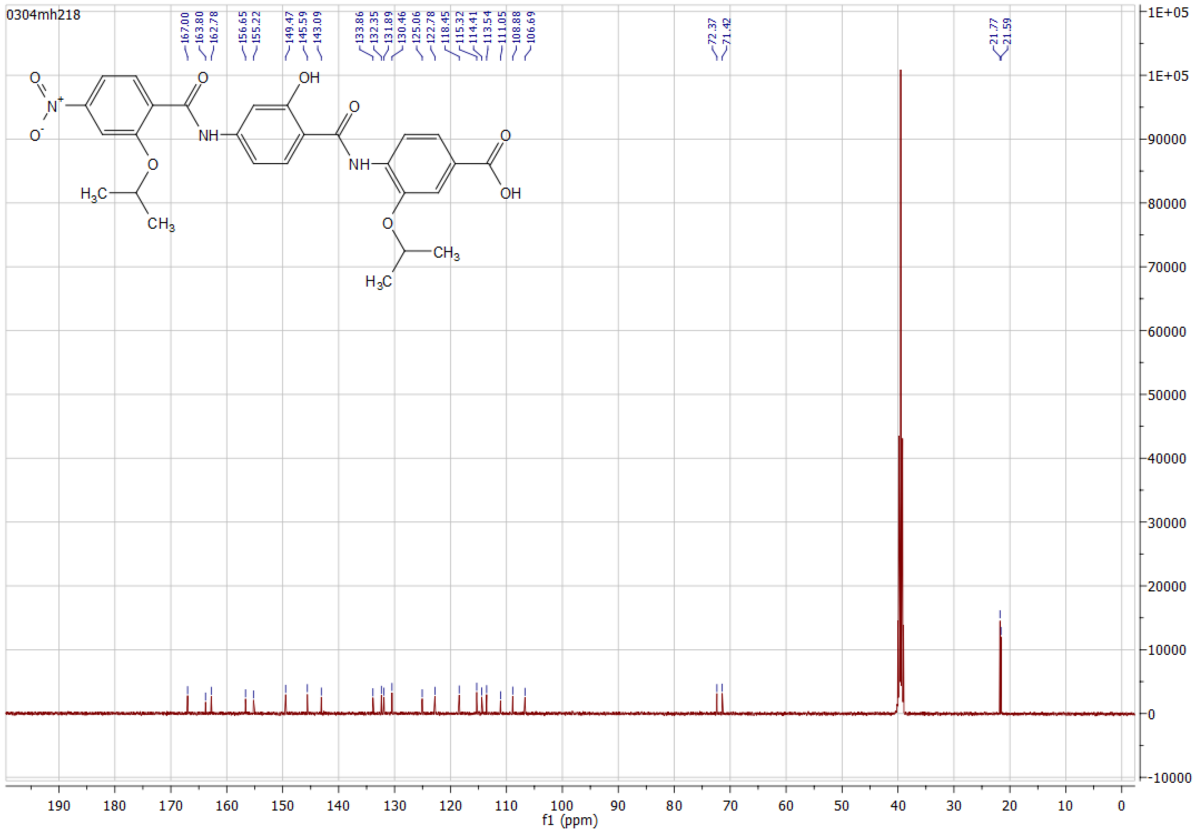

Compound **28**

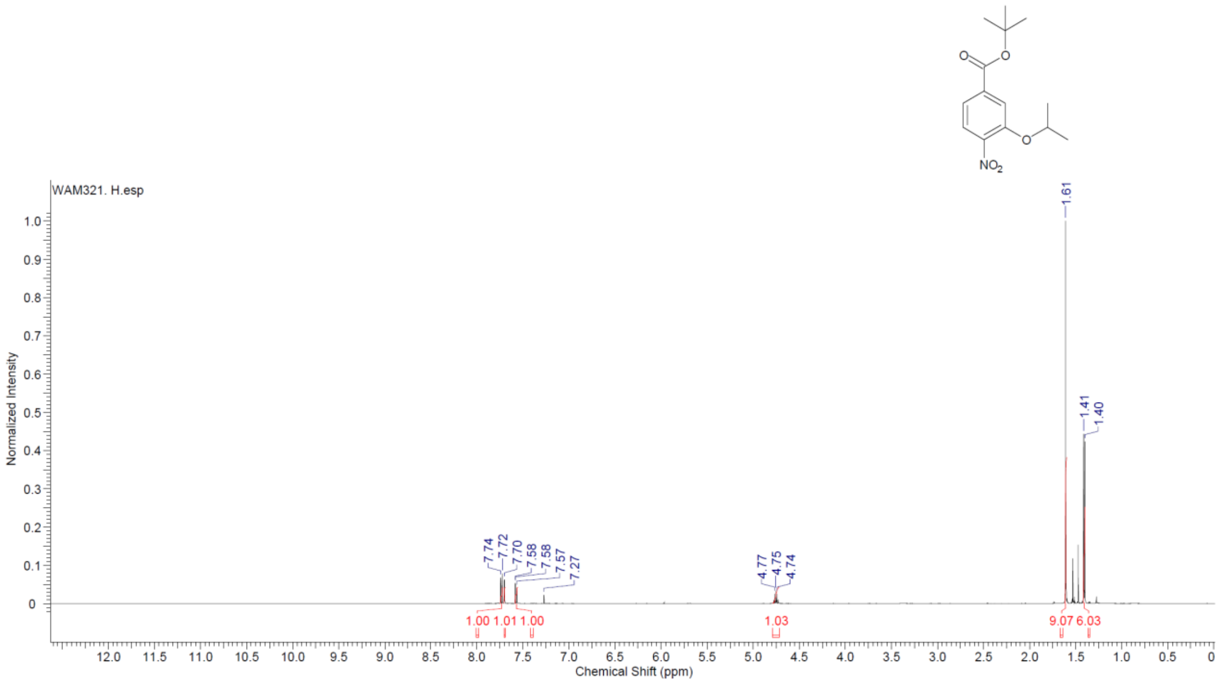

NO2
O
O
O
WAM321. H.esp
Normalized Intensity
Chemical Shift (ppm)
7.74
7.72
7.70
7.58
7.58
7.57
7.27
4.77
4.75
4.74
1.61
1.41
1.40
1.00
1.01
1.00
1.03
9.07
6.03

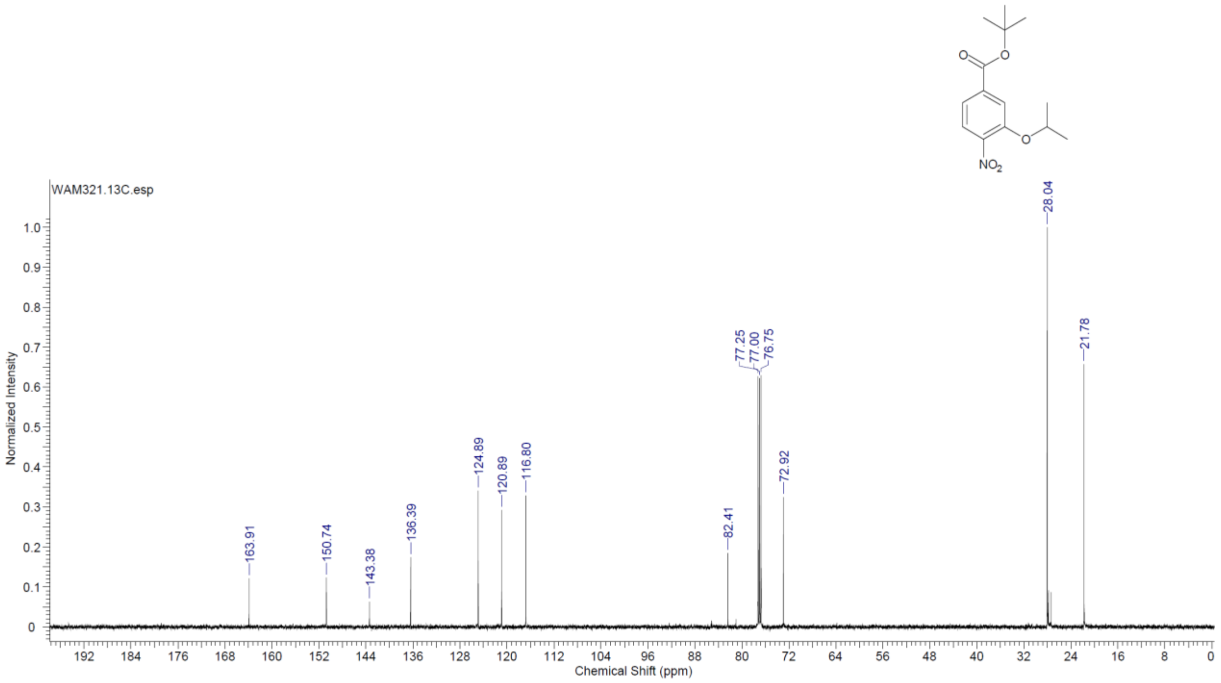

NO2
O
O
O
WAM321.13C.esp
Normalized Intensity
Chemical Shift (ppm)
163.91
150.74
143.38
136.39
124.89
120.89
116.80
82.41
77.25
77.00
76.75
72.92
28.04
21.78

Compound **29**

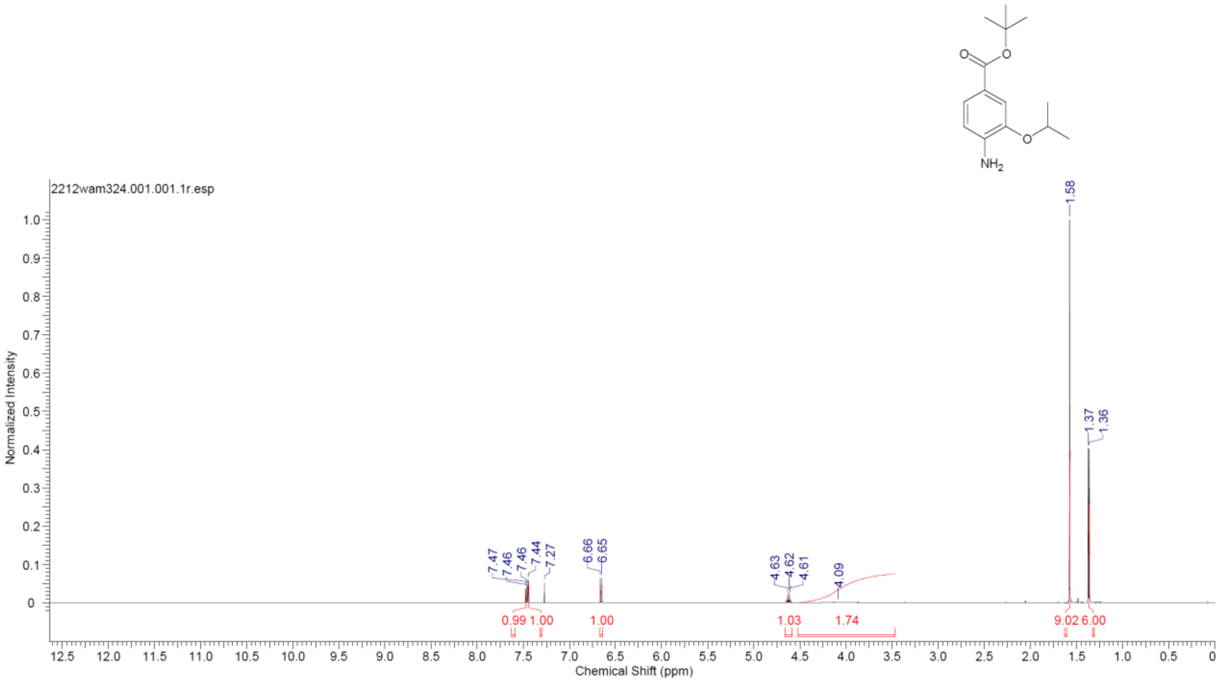

O
O
O
NH_2
2212wam324.001.001.1r.esp
Normalized Intensity
Chemical Shift (ppm)
7.47
7.46
7.46
7.44
7.27
6.66
6.65
4.63
4.62
4.61
4.09
1.58
1.37
1.36
0.99
1.00
1.00
1.03
1.74
9.02
6.00

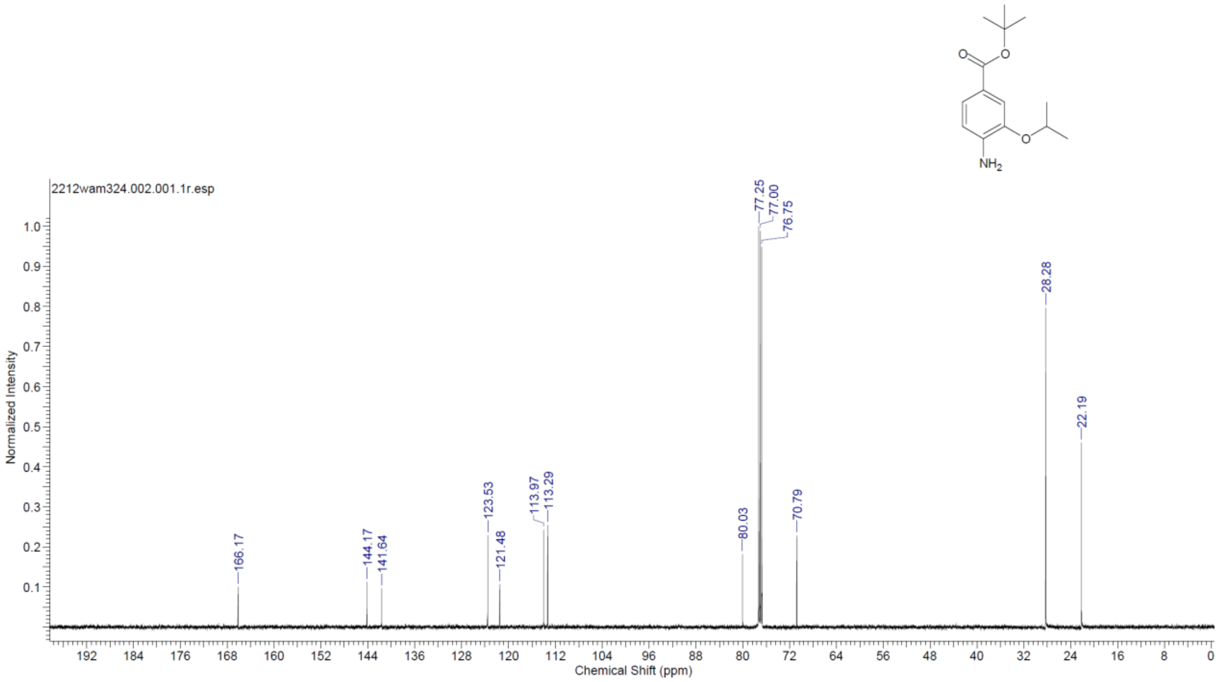

O
O
O
NH_2
2212wam324.002.001.1r.esp
Normalized Intensity
Chemical Shift (ppm)
166.17
144.17
141.64
123.53
121.48
113.97
113.29
80.03
77.25
77.00
76.75
70.79
28.28
22.19

Compound **31**

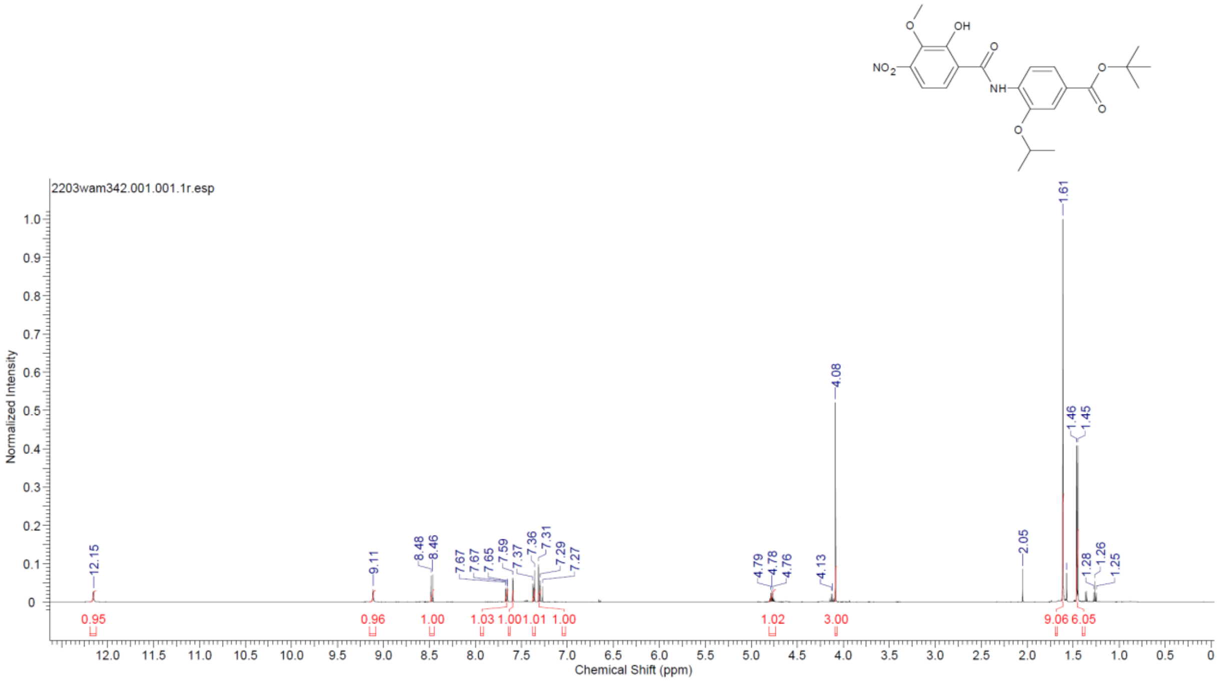

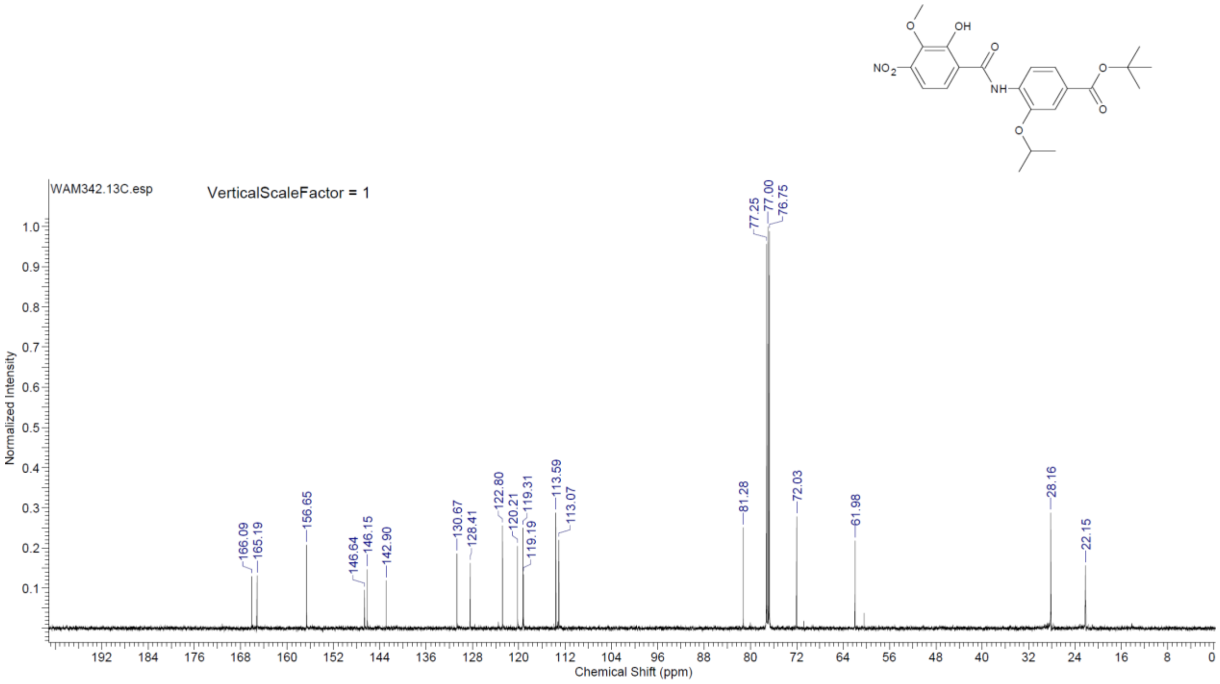

Compound **32**

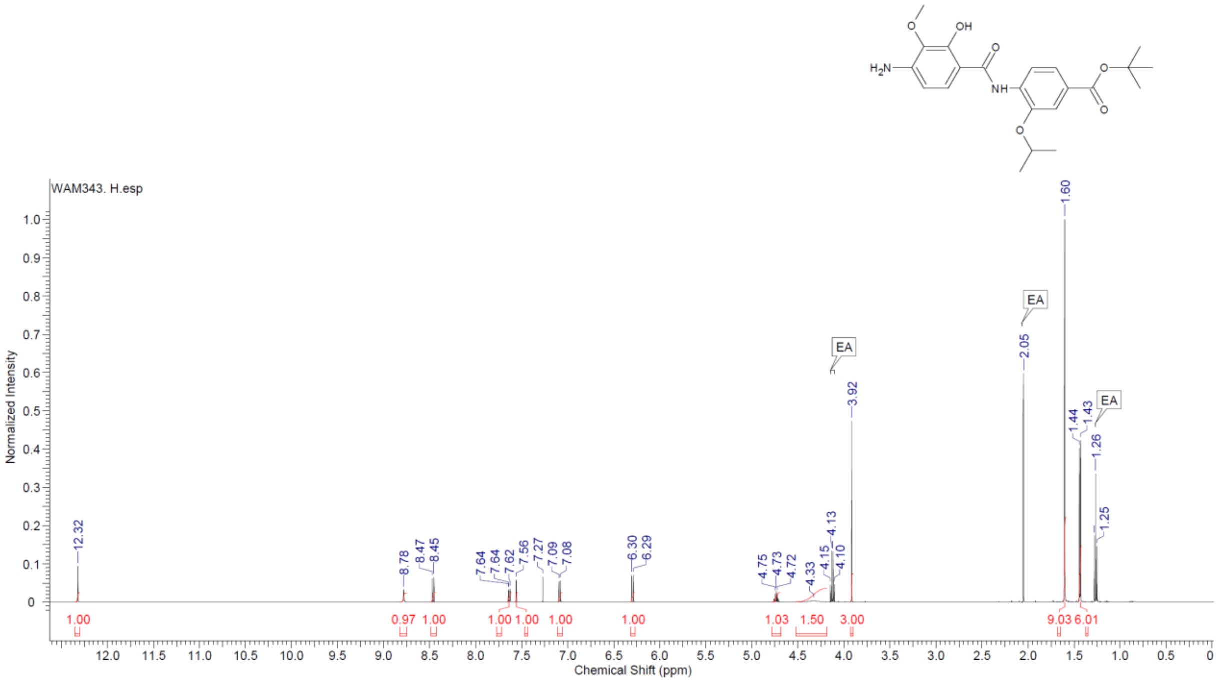

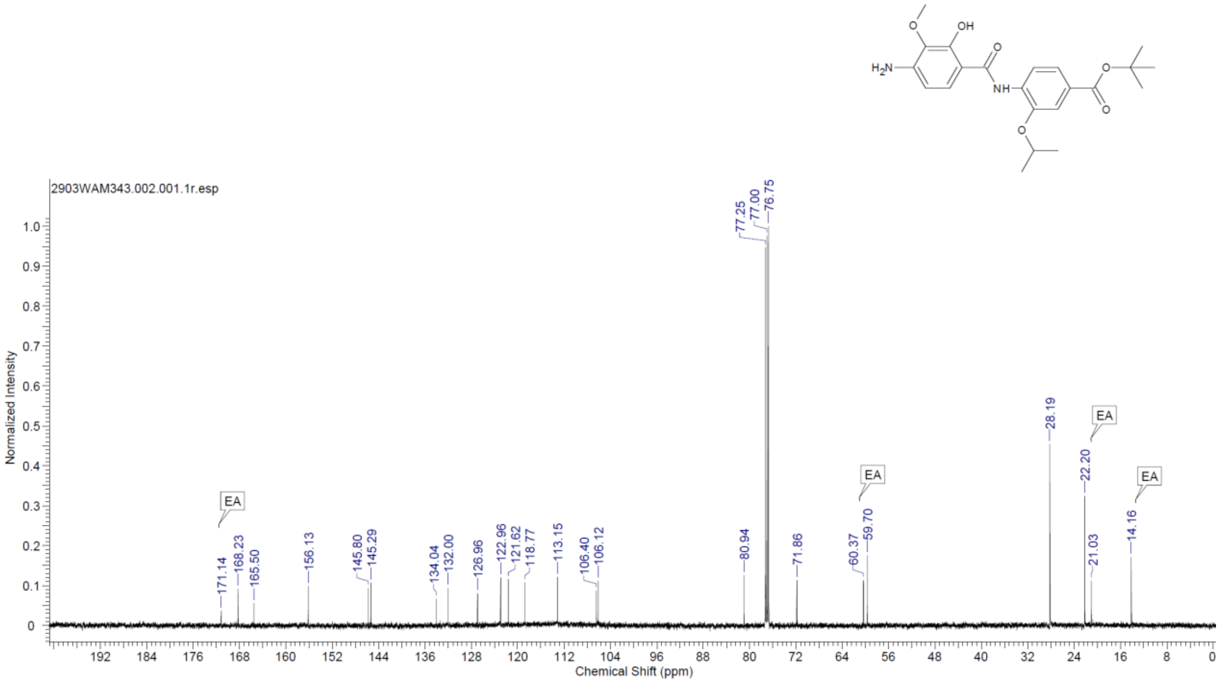

Compound **35**

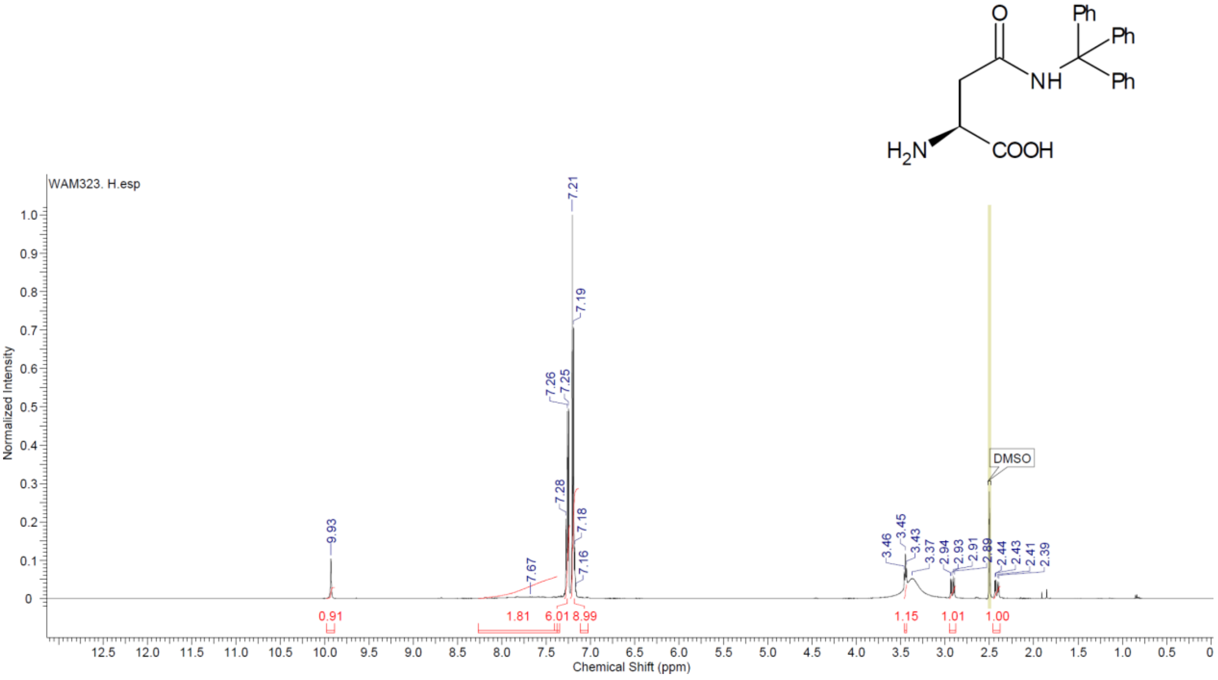

WAM323. H.esp
Normalized Intensity
Chemical Shift (ppm)
DMSO

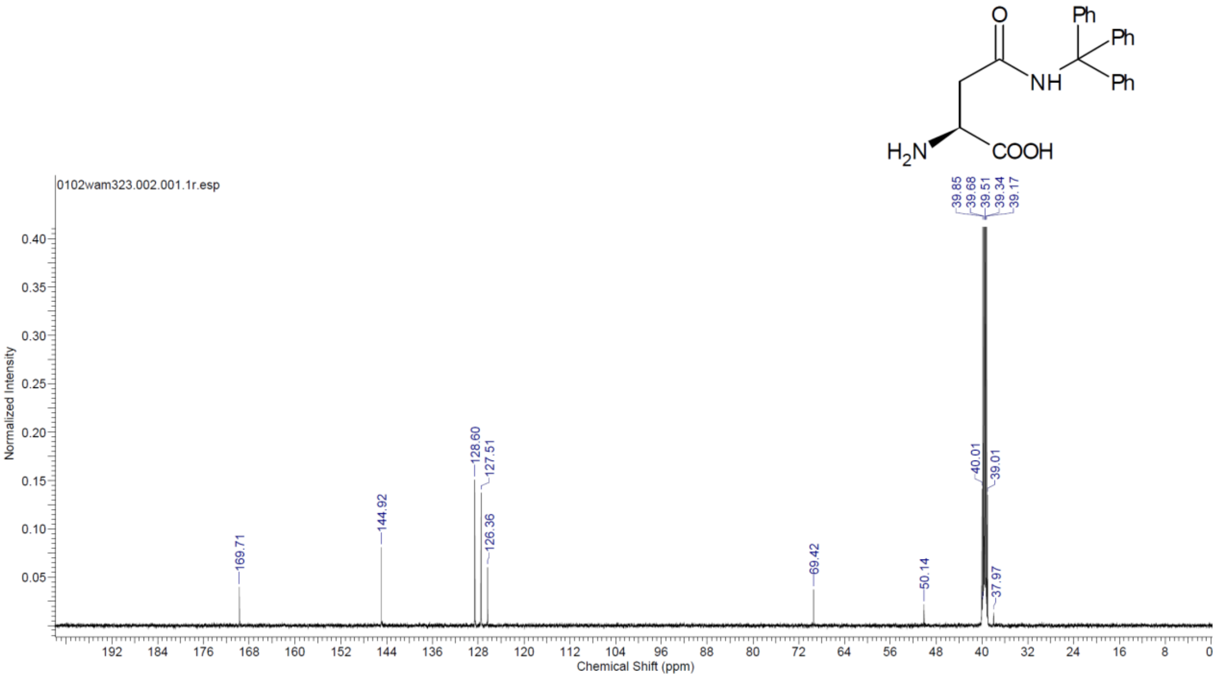

0102wam323.002.001.1r.esp
Normalized Intensity
Chemical Shift (ppm)

Compound **36**

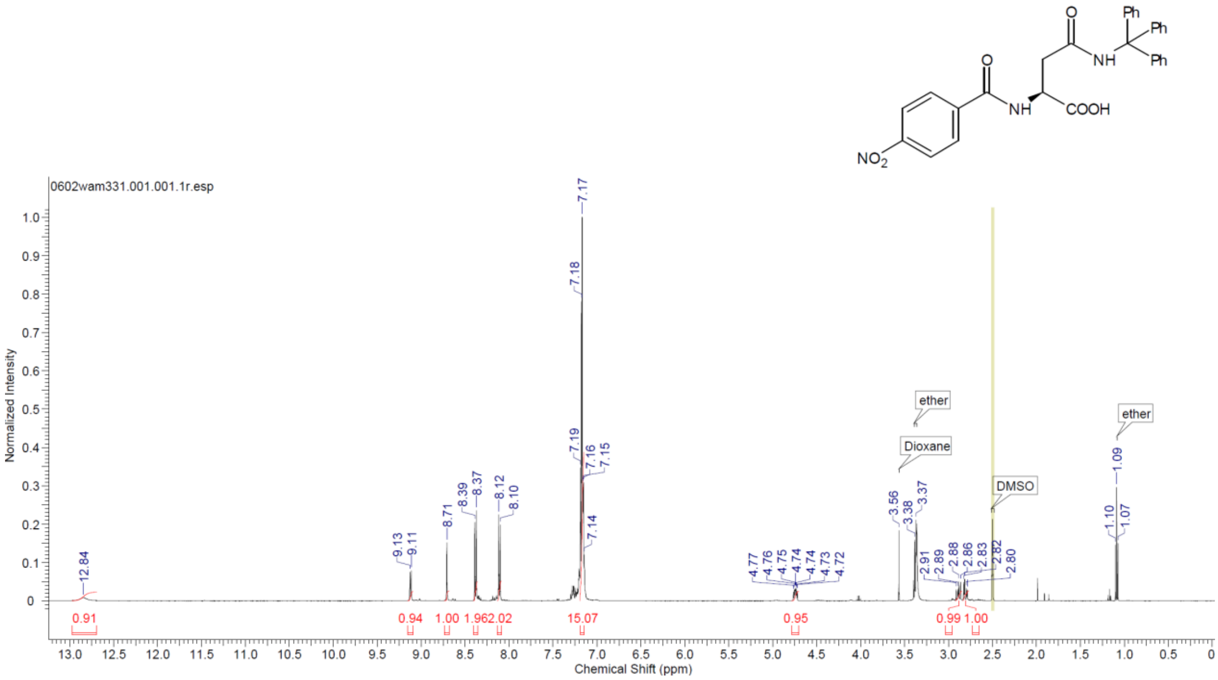
0602wam331.001.001.1r.esp
Normalized Intensity
Chemical Shift (ppm)
ether
Dioxane
DMSO
ether

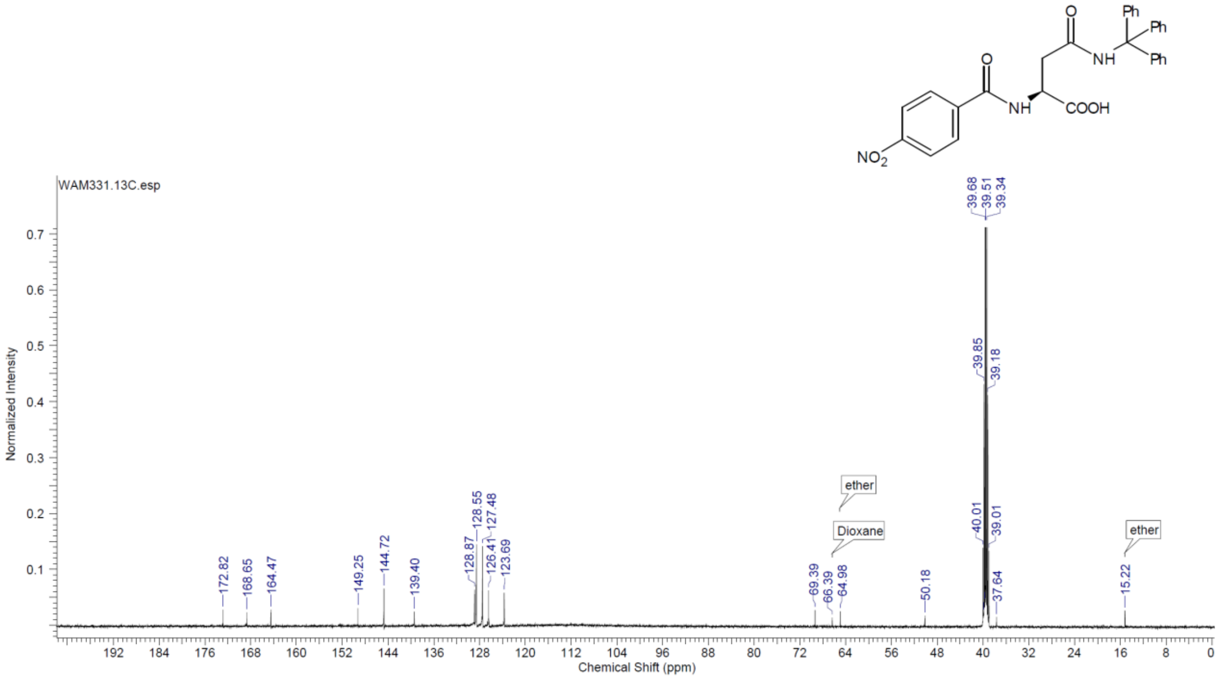
WAM331.13C.esp
Normalized Intensity
Chemical Shift (ppm)
ether
Dioxane
ether

Compound **37**

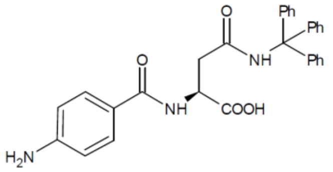

O
Ph
Ph
Ph
NH
O
NH
COOH
H_2N

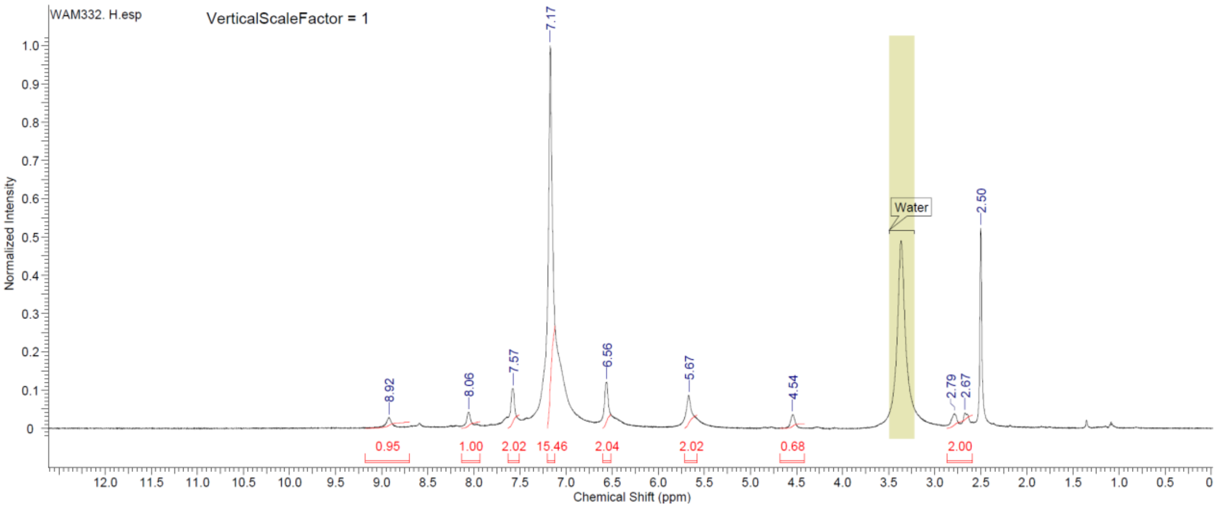

WAM332. H.esp
VerticalScaleFactor = 1
Normalized Intensity
Water
8.92
8.06
7.57
7.17
6.56
5.67
4.54
2.79
2.67
2.50
0.95
1.00
2.02
15.46
2.04
2.02
0.68
2.00
Chemical Shift (ppm)

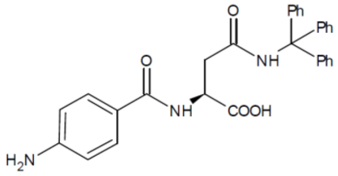

O
Ph
Ph
Ph
NH
O
NH
COOH
H_2N

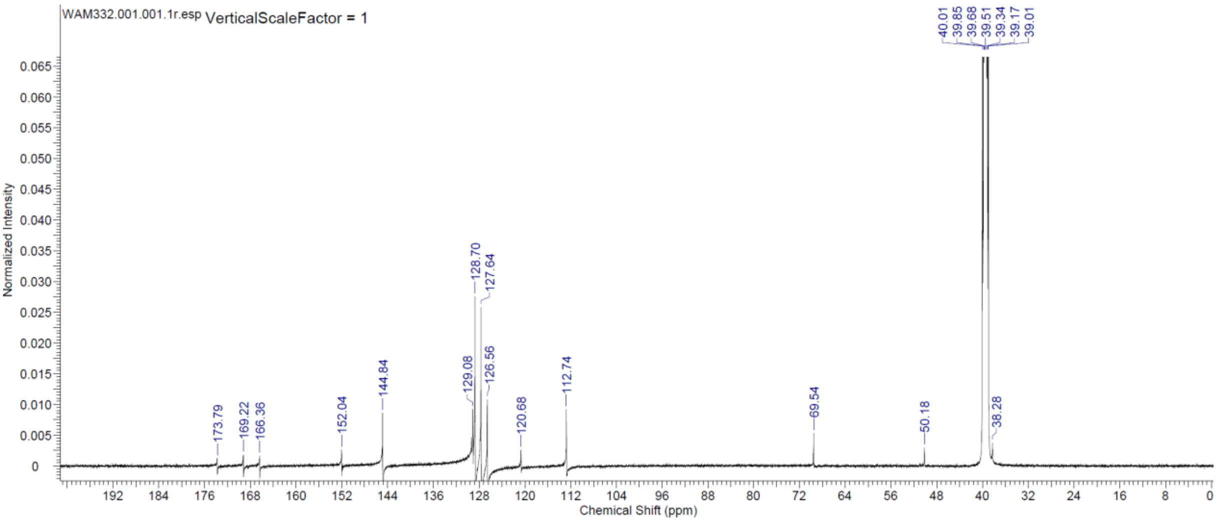

WAM332.001.001.1r.esp
VerticalScaleFactor = 1
Normalized Intensity
173.79
169.22
166.36
152.04
144.84
129.08
128.70
127.64
126.56
120.68
112.74
69.54
50.18
40.01
39.85
39.68
39.51
39.34
39.17
39.01
38.28
Chemical Shift (ppm)

Compound **38**

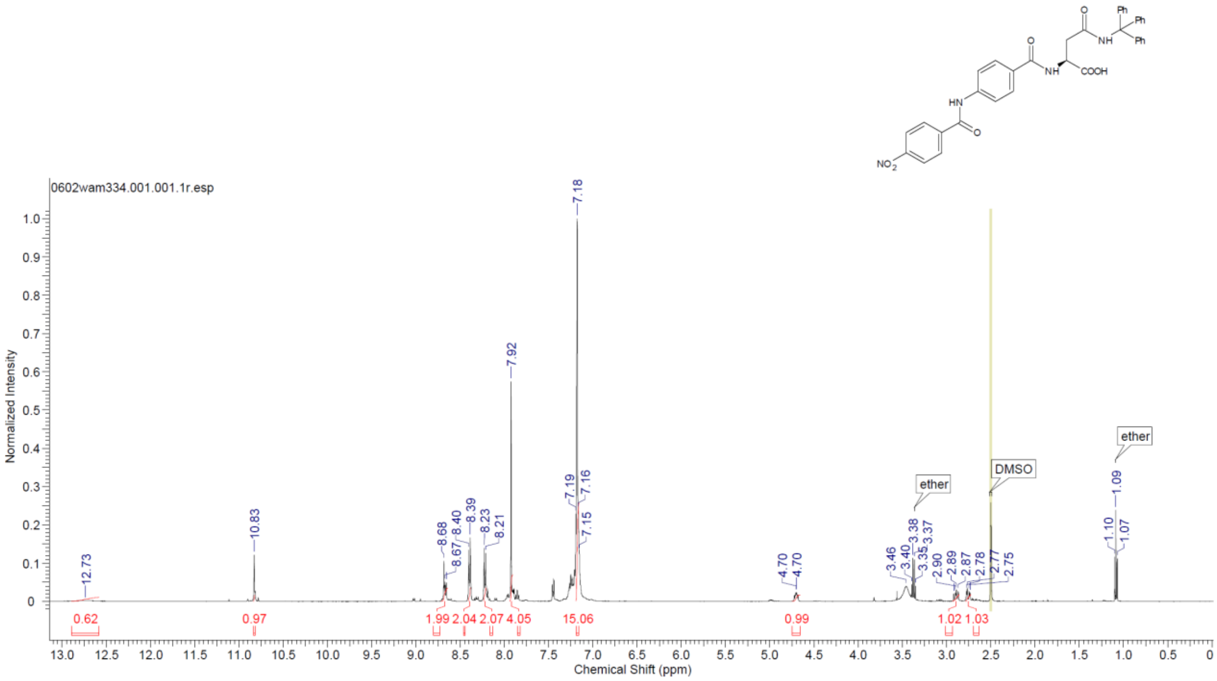

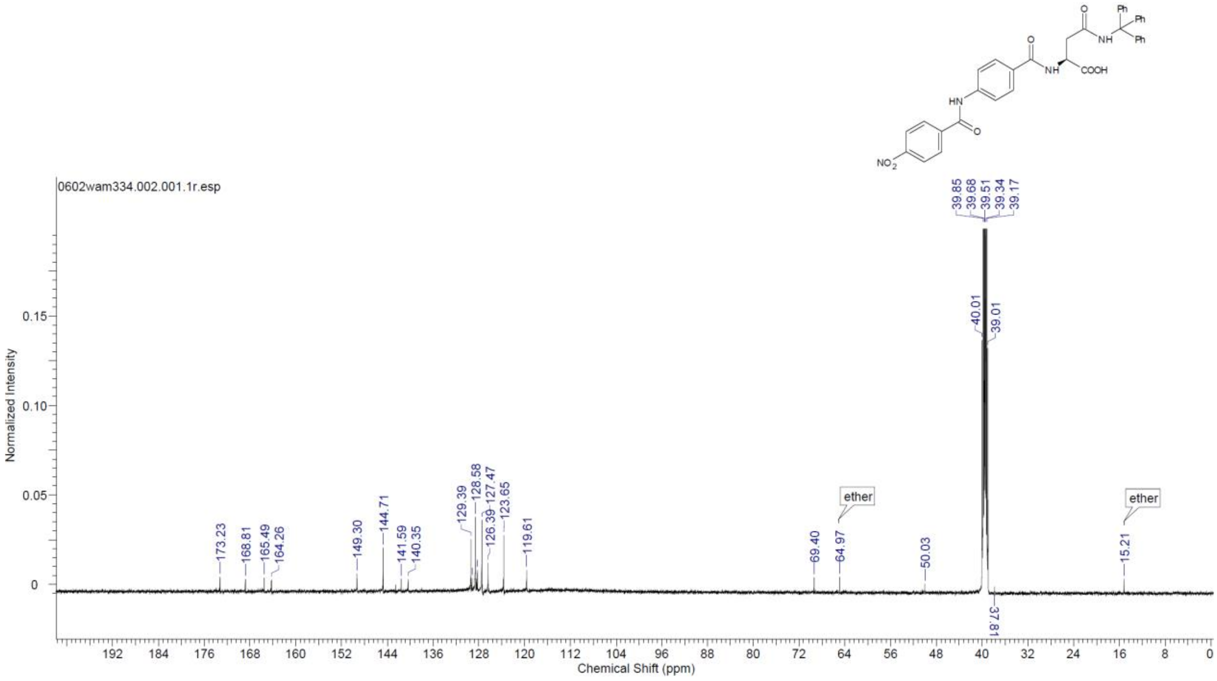

Compound **16**

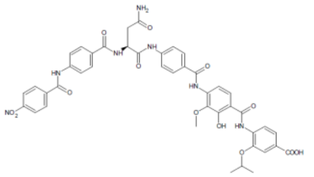

NH2
NH
NH
HN
HN
NO2
OH
HN
COOH

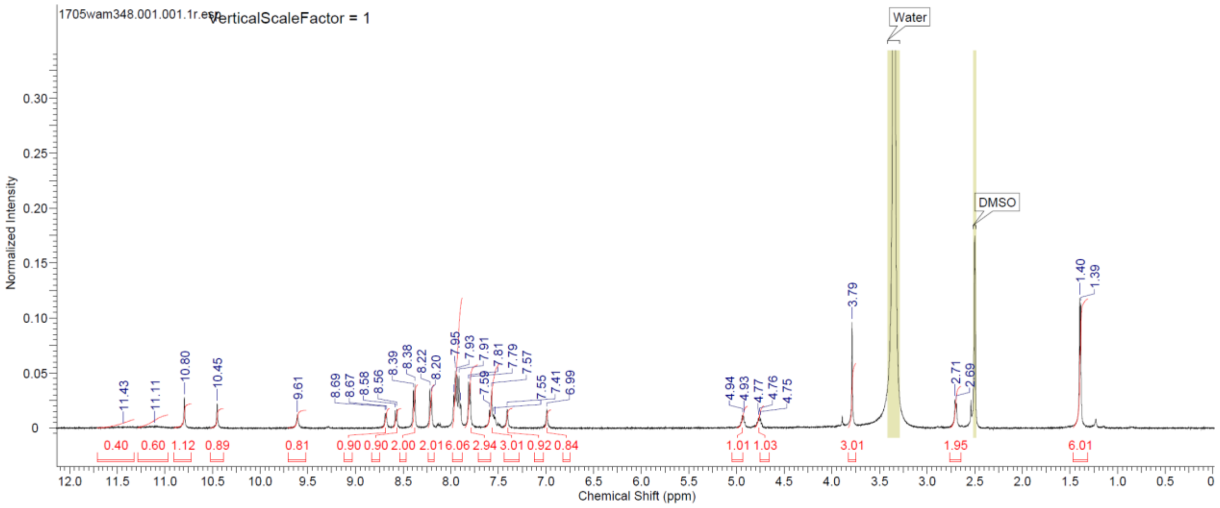

1705wam348.001.001.1r.esp
VerticalScaleFactor = 1
Water
DMSO
Normalized Intensity
Chemical Shift (ppm)
11.43
11.11
10.80
10.45
9.61
8.69
8.67
8.58
8.56
8.39
8.38
8.22
8.20
7.95
7.93
7.91
7.81
7.79
7.59
7.57
7.55
7.41
6.99
4.94
4.93
4.77
4.76
4.75
3.79
2.71
2.69
1.40
1.39
0.40 0.60 1.12 0.89 0.81 0.90 0.90 2.00 2.01 6.06 2.94 3.01 0.92 0.84 1.01 1.03 3.01 1.95 6.01

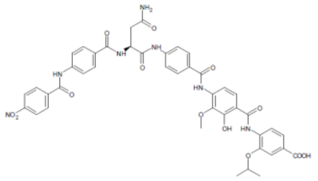

NH2
NH
NH
HN
HN
NO2
OH
HN
COOH

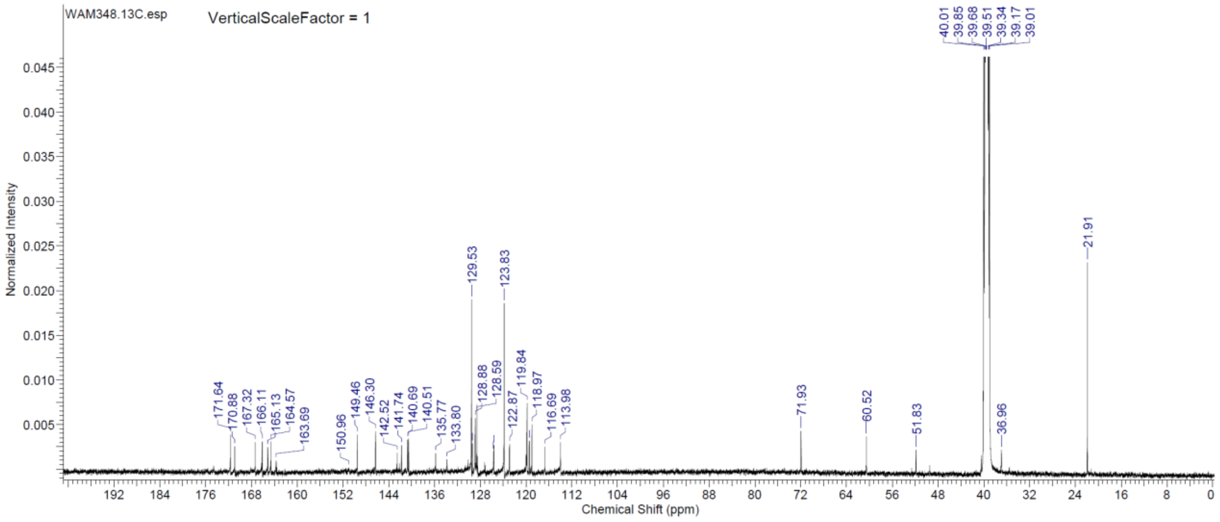

WAM348.13C.esp
VerticalScaleFactor = 1
Normalized Intensity
Chemical Shift (ppm)
40.01
39.85
39.68
39.51
39.34
39.17
39.01
21.91
129.53
123.83
171.64
170.88
167.32
166.11
165.13
164.57
163.69
150.96
149.46
146.30
142.52
141.74
140.69
140.51
135.77
133.80
128.88
128.59
122.87
119.84
118.97
116.69
113.98
71.93
60.52
51.83
36.96
